# Minimization of Back‐Electron Transfer Enables the Elusive sp^3^ C−H Functionalization of Secondary Anilines

**DOI:** 10.1002/anie.202100051

**Published:** 2021-03-03

**Authors:** Huaibo Zhao, Daniele Leonori

**Affiliations:** ^1^ Department of Chemistry University of Manchester Oxford Road Manchester M13 9PL UK

**Keywords:** anilines, C−H functionalization, electron transfer, late-stage modification, photoredox catalysis

## Abstract

Anilines are some of the most used class of substrates for application in photoinduced electron transfer. N,N‐Dialkyl‐derivatives enable radical generation α to the N‐atom by oxidation followed by deprotonation. This approach is however elusive to monosubstituted anilines owing to fast back‐electron transfer (BET). Here we demonstrate that BET can be minimised by using photoredox catalysis in the presence of an exogenous alkylamine. This approach synergistically aids aniline SET oxidation and then accelerates the following deprotonation. In this way, the generation of α‐anilinoalkyl radicals is now possible and these species can be used in a general sense to achieve divergent sp^3^ C−H functionalization.

## Graphical abstract

Divergent radical sp^3^ C−H functionalization of N‐alkyl anilines has been achieved using a photoredox strategy. This approach enables to overcome back‐electron transfer and therefore allows direct access to previously elusive α‐anilinoalkyl radicals.

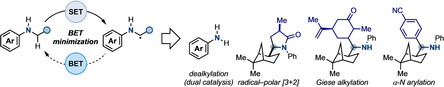

Anilines are one of the most important building blocks in organic chemistry. They are widespread in the core structure of high‐value materials like drugs and agrochemicals, and also have a rich chemistry for utilization in both polar and radical strategies.^[1]^ Indeed, the electron rich nature of the N‐atom, combined with the stabilisation offered by the neighbouring aromatic ring, makes anilines powerful electron donors and this has been pivotal for their exploitation in redox‐based processes.^[2]^ However, N‐substitution is also known to exert a large difference in the way these substrates behave upon single‐electron transfer (SET). Taking *N*,*N*‐Me_2_‐aniline **1** as an example, it is remarkable that while α‐anilinoalkyl radical **B** generation by SET oxidation followed by deprotonation (**1** → **A** → **B**) is a standard process in photocatalysis, the translation of the same elementary steps to *N*‐Me‐aniline **2** (**2** → **C** → **D**) is unprecedented (Scheme 1 A).^[3]^ This lack of reactivity can be exemplified considering the photooxidative demethylation of anilines, one of the most studied and applied reaction in the field of photoinduced SET.[^2c^, ^4^] This transformation can be triggered by a broad range of photocatalysts but it always stops upon the removal of the first Me‐group (**1** → **2**) and **2** never engages in a following demethylation to the free aniline **3**. In the broader sense it is remarkable that the direct conversion of **2** to **3** is currently beyond the scope not just of photoredox catalysis but also of any redox, thermal and transition metal‐mediated approach.^[5]^

**Scheme 1:**
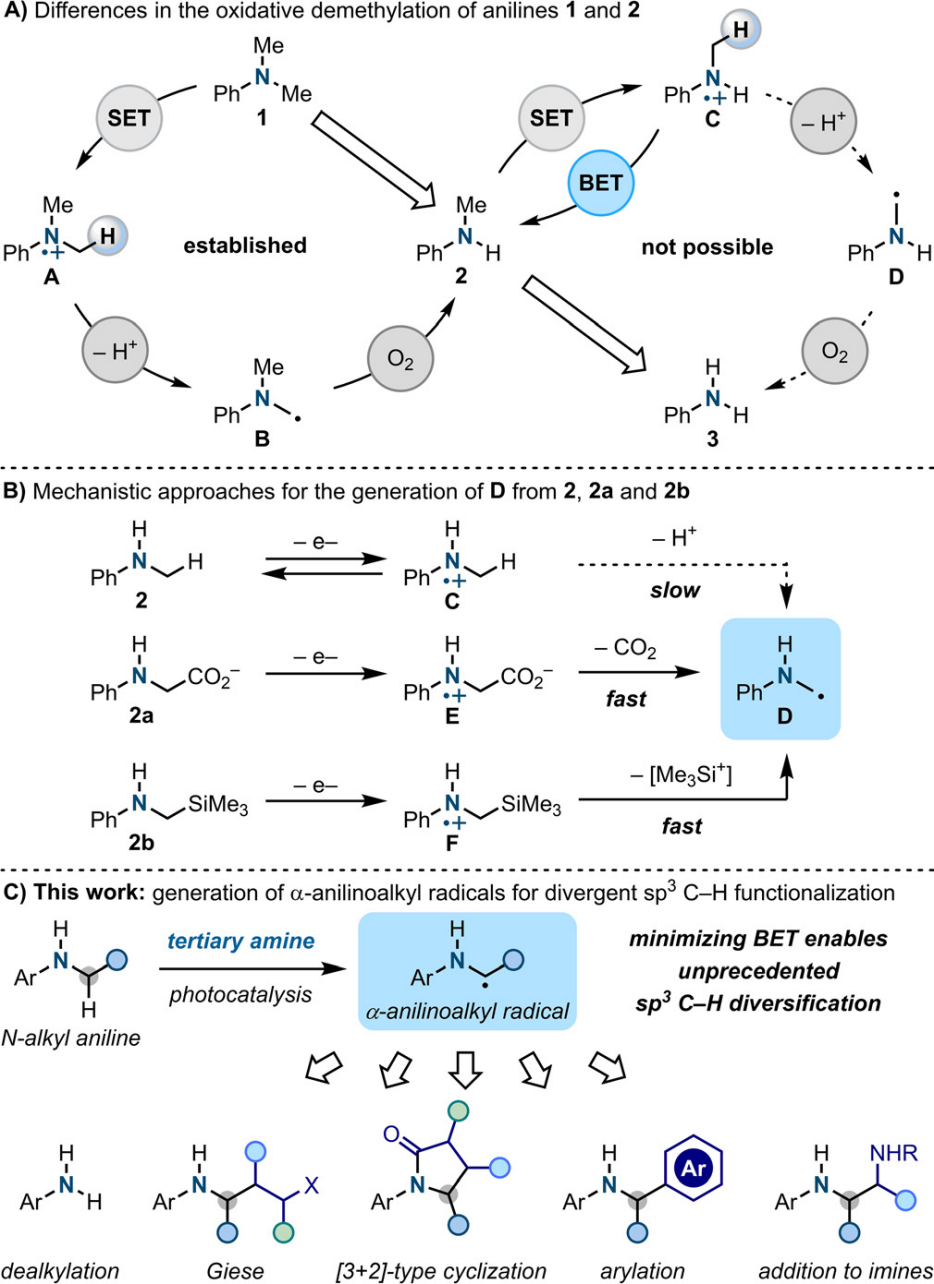
A) Analysis of the reactivity of N,N‐Me_2_ vs. N‐Me anilines in redox chemistry. B) Differences in reactivity of N‐alkyl‐aniline derived aminium radicals. C) This work enables α‐anilinoalkyl radical generation for divergent α‐N sp^3^ C−H functionalization.

Since the redox properties of **1** and **2** are relatively similar and well within the range accessible by excited photocatalysts, the ability of **2** to resist photooxidative demethylation is generally rationalised on the basis of back‐electron transfer (BET).^[6]^ Indeed, pioneering studies from Dinnocenzo and Mariano demonstrated that, upon oxidation, the deprotonation of aromatic aminium radicals **C** is slow (*k*_obs_≈10^5^  s^−1^) which leads to re‐generation of **2** by BET (Scheme 1 B).^[7]^ The kinetics of deprotonation can be favoured by installing electron withdrawing groups onto the aniline aromatic ring but this has the detrimental effect of making the initial SET more difficult therefore accelerating BET.^[8]^ Overall, the subtle interplay of the elementary steps of electron transfer and deprotonation has, so far, thwarted the development of methodologies for direct sp^3^ C−H functionalization of secondary N‐alkyl‐anilines. Currently, the only feasible way to access and exploit the reactivity of radical **D** is to use either *N*‐Ph‐glycine **2 a** or the α‐silylated derivative **2 b**. These two systems have the intrinsic advantage the once SET oxidation occurs, subsequent and diffusion‐controlled decarboxylation (**E** → **D**) or desilylation (**F** → **D**) take place (*k*_obs_≈10^10^  s^−1^).^[9]^

Here we demonstrate that BET can be minimised by simply using basic alkylamines to synergistically facilitate aniline SET oxidation and the following deprotonation (Scheme 1 C). This approach has enabled effective access to α‐anilinoalkyl radicals which can now be used for the elusive α‐N sp^3^ C−H functionalisation of monosubstituted anilines. Most notably, this strategy has also provided the first catalytic approach to achieve unprecedented N‐dealkylation.

This work originated by a serendipitous observation made during routine cyclic voltammetry experiments. Specifically, we noticed that upon addition of Et_3_N or piperidine to **2**, new peaks appeared in the voltammogram with, crucially, oxidation potentials lower than then the ones of the isolated species (Scheme 2 A).^[10]^ A similar outcome, albeit less dramatic, was also observed upon performing Stern–Volmer luminescence quenching studies using the photoredox catalyst Ir(dtbbpy)(ppy)_2_PF_6_.^[10]^ These unforeseen results led us to consider the formation of a H‐bonded complex between **2** and the two amines (e.g. Ph(Me)N−H⋅⋅⋅NEt_3_) which should increase electron density on the aniline N‐atom. This interaction, would lower the aniline oxidation potential and also increase the rate of quenching of the *[Ir] photocatalyst. At this stage, we propose this effect might be perceived as an attenuated version of what usually observed in proton‐coupled electron transfers (PCETs) between amides and phosphate bases.^[11]^ We were therefore intrigued by the possibility that this pre‐association upon facilitating SET oxidation could also be used to synergistically aid a following deprotonation that, circumventing BET, would provide access to the α‐anilinoalkyl radical **D**.

**Scheme 2:**
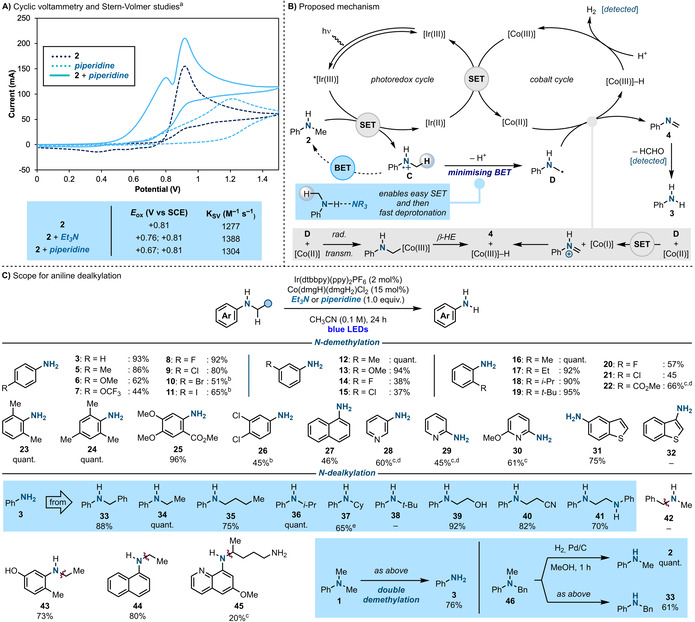
A) Cyclic voltammetry and Stern–Volmer studies. B) Proposed mechanism for aniline demethylation. C) Scope of the process. [a] The cyclic voltammetry data for Et_3_N is in the Supporting Information. [b] The reaction time was 40 h. [c] The reaction time was 72 h. [d] 4CzIPN was used as the photocatalyst. [e]The reaction time was 48 h. β‐HE=β‐hydride elimination‐type process.

In approaching this blueprint for α‐anilinoalkyl radical generation, we envisaged its utilization to target the still elusive demethylation of N‐methyl anilines (Scheme 2 B). Specifically, we proposed the development of a dual photoredox‐cobalt^[12]^ catalytic system where a visible light‐excited [Ir^III^] photocatalyst would, helped by the amine, generate **D** by oxidation and deprotonation of **2** while minimizing BET.^[13]^ As **D** is a nucleophilic radical (local electrophilicity index,^[14]^ ω^+^_rc_=0.36), we speculated it could react with a [Co^II^] co‐catalyst and undergo dehydrogenation to give a [Co^III^]‐H species and imine **4**. A following hydrolysis would provide the desired demethylated product **3**. The pathway for dehydrogenation can be rationalised considering the following two mechanistic options. (1) Radical capture of **D** by [Co^II^] could generate an alkyl‐[Co^III^] species followed by β‐hydride‐type elimination^[15]^ across the N−H bond. (2) Since **D** is also an electron rich radical (IP=6.1 V), an alternative process would involve direct SET between **D** and [Co^II^] to give, via a [Co^I^],^[16]^ **4** and [Co^III^]‐H. While it is difficult at this stage to distinguish between the two mechanistic options, both of them ultimately result in the formation of the [Co^III^]‐H. This species would then evolve H_2_ by reaction with a protic source,^[17]^ with the resulting electron poor [Co^III^] closing both photoredox and cobalt cycles by SET with [Ir^II^].^[18]^

To put this proposal into practice, we first evaluated the demethylation of **2** using both Et_3_N and piperidine (Scheme 2 C). While in their absence no reactivity could be observed,^[10]^ a survey of reaction conditions revealed an optimal process that involved the use of the Ir(dtbbpy)(ppy)_2_PF_6_ ^[19]^ photocatalyst and the cobaloxime co‐catalyst Co(dmgH)(dmgH_2_)Cl_2_ in CH_3_CN solvent under blue light irradiation.^[20]^ Under these mild conditions **3** was formed in high yields. Crude reaction analysis enabled the detection of formaldehyde, indicative of the generation of **4**, as well as H_2_, which supports the interplay of a [Co^II/III^] cycle.^[10]^

The scope of the demethylation process was initially evaluated by using mono‐substituted N‐methyl anilines. *para*‐Substituted aryl groups displaying electron‐rich (**5**, **6**), electron‐deficient (**7**, **8**) and synthetically useful functionalities (**9**–**11**) provided the desired products in good to high yields. The ability to engage substrates containing electron poor arenes is remarkable since BET from their corresponding aminium radicals is accelerated.[^8^, ^21^] *meta*‐Substitution was evaluated next and electronically different functionalities were tolerated albeit in lower yield in the case of electron poor systems (**12**–**15**). We then trialled *ortho*‐substituted aromatics and also in this case very high yields were obtained across a broad range of derivatives (**16**–**22**). In this case, the steric hinderance provided by the substituent decreases the stabilisation of the aminium radical and therefore enhances the rate of deprotonation which cooperates to minimise BET.^[8]^ This effect is also operating in the demethylations leading to **23**–**25** which were quantitative. The chemistry was amenable to a less activated dichloro aniline (**26**) and found compatible to other aromatics like *N*‐methylnapthalen‐1‐amine (**27**), several aminopyridines (**28**–**30**) and 5‐amino‐benzothiophene (**31**). In terms of limitations, we did not succeed in engaging heteroaromatic anilines located on electron rich 5‐membered ring systems (e.g. **32**).

Having developed the first catalytic approach for the demethylation of secondary anilines, we were keen to understand if this dual photoredox‐cobalt approach could be used in a general sense as a N‐dealkylation platform. In this case, to evaluate the impact of the alkyl chain we initially looked at a series of N‐Ph derivatives. Pleasingly, the process displayed broad scope enabling the removal of a benzyl group (**33**) as well as challenging primary (**34** and **35**) and secondary (**36** and **37**) alkyl chains. As an α‐anilinoalkyl radical cannot be formed in the case of a *t*‐Bu substituent, **38** resulted in complete starting material recovery which agrees with our mechanistic analysis. The chemistry was also expanded to the removal of functionalised alkyl chains containing inductively electron withdrawing substituents (**39** and **40**) and could also be used to fully dealkylate an ethane‐1,2‐diamine derivative (**41**). In line with our mechanistic analysis, dealkylation of *N*‐Me‐*N*‐Bn‐amine **42** was not possible and this substrate was recovered at the end of the reaction. In this case, Stern–Volmer and cyclic voltammetry analysis revealed no interaction with Et_3_N or piperidine, which does not enable to overcome BET. The approach was also demonstrated on a series of more complex derivatives (**43**–**45**) which included the blockbuster drug primaquine. We then benchmarked the strategy on the demethylation of **1**. While all previous approaches stop after the removal of the first methyl group,[^3^, ^4^] ours enables, for the first time, complete dealkylation to **3** in good yield by simply extending the reaction time. Finally, we were able to demonstrate the orthogonality that this strategy might offer with respect to standard hydrogenation protocols. Taking *N*‐Me‐*N*‐Bn‐aniline **46**, all reported deprotections enable debenzylation (**46** → **2**) while this approach provides a switch in selectivity and targets the removal of the Me‐group (**46** → **33**).^[22]^

The chemistry described here demonstrates that α‐anilinoalkyl radicals can be conveniently accessed under mild conditions from the corresponding secondary anilines. Since previous exploitation of these open shell intermediates in synthetic chemistry required the preparation and use of N‐aryl glycines or α‐silyl derivatives (see also Scheme 1 B),^[23]^ this approach has the potential to facilitate divergent sp^3^ C−H functionalization.

A relevant application would involve application in radical‐polar crossover^[24]^ reactions involving acrylates for the construction of poly‐substituted pyrrolidones (Scheme 3 A).^[25]^ This would exploit the intrinsic vicinal radical‐ionic di‐nucleophilic nature of **D** and constitute a novel [3+2]‐like retrosynthetic disconnection alternative to more classical approaches based on azomethine ylide chemistry.^[26]^ Pleasingly, the realization of this cascade process was achieved by employing conditions similar to the ones presented above but, in this case, quinuclidine gave slightly increased yields over Et_3_N and piperidine (Scheme 3 B).[^10^, ^27^] Scope investigation started by using **2** as the aniline and a range of different acrylates. This enabled the preparation of several C3‐ (**48** and **52**) and C4‐substituted derivatives (**49** and **50**) as well as products containing a 4‐*gem*‐dimethyl group (**51**). The process tolerated valuable functionalities like free alcohol (**52** and **53**), fluorine (**54**) and acetamide (**55**). We also demonstrated reactivity with tri‐substituted acrylates that led to challenging 3,4‐disubstituted pyrrolidones (**56**–**59**) in good yields.

**Scheme 3:**
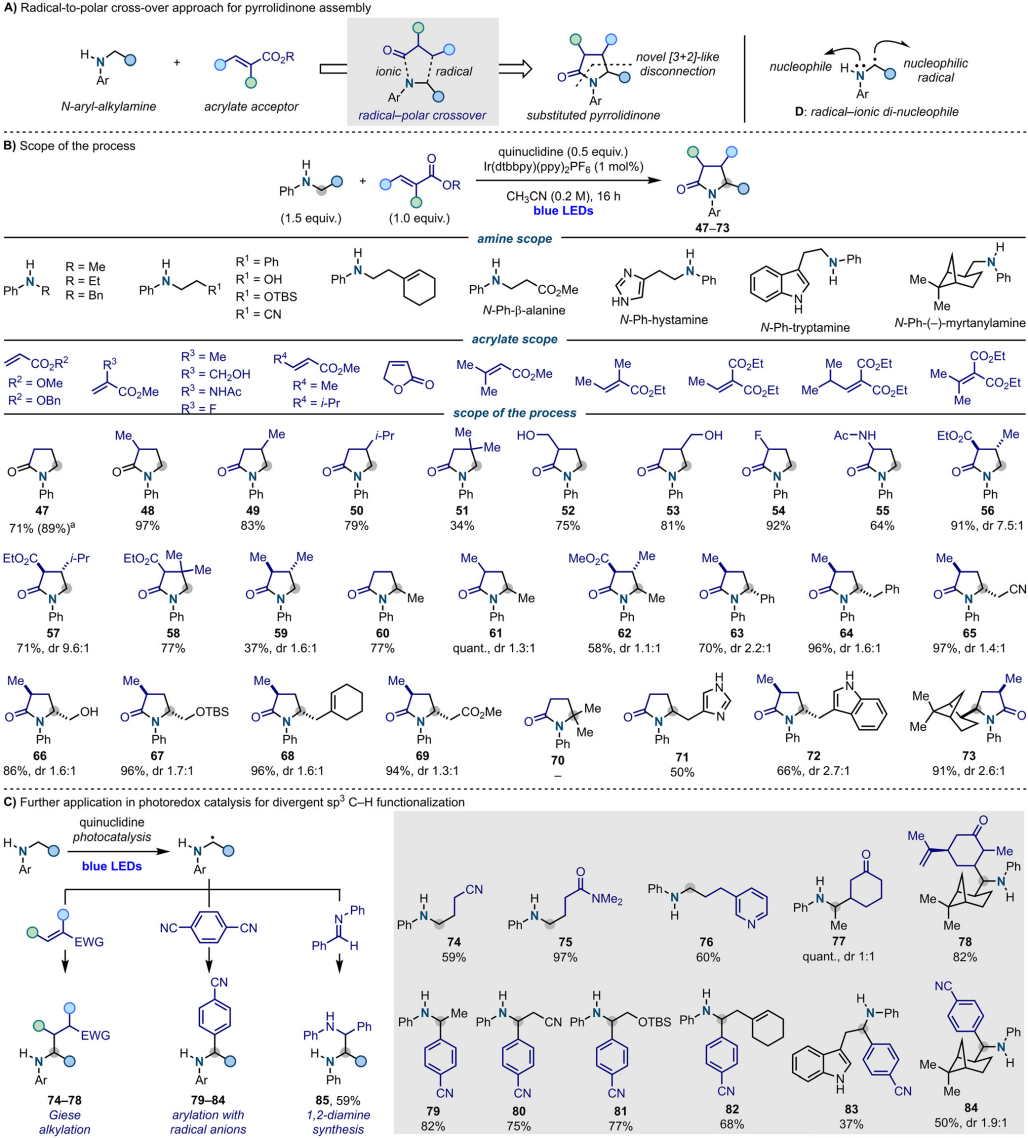
A) Utilization of α‐anilinoalkyl radicals in radical‐polar cross‐over reactions for pyrrolidone assembly. B) Scope of the process. C) Extension of α‐anilinoalkyl radical reactivity in other photoredox mediated transformations and current limitations. [a] Yield using benzyl acrylate. EWG=electron‐withdrawing group.

We then evaluated the range of anilines compatible as modification of the N‐substitution would enable introduction of alkyl/aryl substituents at the 5‐position – a key feature of many bioactive pyrrolidone‐ and pyrrolidine‐based natural products. Pleasingly, the use of N‐Et‐ and N‐Bn‐anilines resulted in the high‐yielding formation of 5‐Me and ‐Ph derivatives (**60**–**63**).

Other N‐alkylanilines containing several functionalities were tolerated and these include nitrile (**65**), free and TBS‐protected hydroxyl (**66** and **67**), alkene (**68**) and, by using a protected β‐alanine, also ester (**69**). At the moment, this reactivity is only feasible on primary N‐alkyl chains as efforts towards the inclusion of *N*‐*i*Pr‐aniline (**70**) proved unsuccessful.

The scope of anilines amenable to this cascade reactivity was further extended by N‐arylating the neurotransmitters histamine and tryptamine and the alkaloid (−)‐*cis*‐myrtanylamine that provided **71**–**73** in good yields. These examples demonstrate that this strategy can be used to install a pyrrolidone system across the linear structure of bio‐active molecules. This can be used to prepare structurally rigid analogues with potential application in bio‐organic and medicinal chemistry.

As a finale effort, we question if this approach for α‐anilinoalkyl radical generation could be used in a general sense within the remit of photoredox catalysis. Scheme 3 C shows successful application in α‐alkylation of anilines via Giese addition to α,β‐unsaturated nitrile (**74**), amide (**75**), 3‐vinylpyridine (**76**) and ketones (**77** and **78**). α‐Aryl amines are valuable synthetic intermediates but standard procedures for α‐N sp^3^ C−H arylation generally require the use of carbamates/amides or tertiary amines and H‐atom abstraction as radical‐generating step.^[2k]^ In this case, treatment of several N‐alkyl anilines with 1,4‐di‐cyanobenzene resulted in the formation of **79**–**84** in good yield. This demonstrate that these conditions are compatible for radical‐radical couplings with persistent aromatic radical anions.^[28]^ Finally, we also succeed in performing a reaction with the N‐Ph‐imine of benzaldehyde, which furnished 1,2‐diamine **85** where both amines can be further functionalised.

The results reported here demonstrate that secondary N‐alkyl anilines can be converted into the corresponding α‐anilinoalkyl radicals using photoredox catalysis. This reactivity is possible through the minimization of back‐electron transfer and by‐passes the current requirement for α‐N pre‐functionalized aniline substrates. The ability to engage the α‐anilinoalkyl radicals in a broad array of C−C bond‐forming processes has enabled divergent sp^3^ C−H functionalization while, dual catalysis with cobalt catalysis has provided the first catalytic approach for secondary aniline dealkylation.

## Conflict of interest

The authors declare no conflict of interest.

## Supporting information

As a service to our authors and readers, this journal provides supporting information supplied by the authors. Such materials are peer reviewed and may be re‐organized for online delivery, but are not copy‐edited or typeset. Technical support issues arising from supporting information (other than missing files) should be addressed to the authors.

Supplementary
