# Supplementary material for: Minimization of Back‐Electron Transfer Enables the Elusive sp3 C−H Functionalization of Secondary Anilines
Source: Angew Chem Int Ed Engl. 2021 Mar 3;60(14):7669–74. doi: 10.1002/anie.202100051 (PMC8048505; doi:10.1002/anie.202100051)
Supplement: Supplementary file 1 — Supplementary [file ANIE-60-7669-s001.pdf]

Angewandte Chemie
Eine Zeitschrift der Gesellschaft Deutscher Chemiker

Supporting Information

Minimization of Back-Electron Transfer Enables the Elusive sp^3 C–H Functionalization of Secondary Anilines

*Huaibo Zhao and Daniele Leonori**

anie_202100051_sm_miscellaneous_information.pdf

Table of Contents

1 General Experimental Details....2

2 Starting Material Synthesis....3

3 Reaction Optimizations....6

3.1 Aniline *N*-Demethylation....6

3.2 [3+2]-Type Cycloadditions....10

3.3 α-*N* sp^3 C–H Arylation....12

4 Picture of Reaction Set-Up....13

5 Substrate Scope....14

5.1 Aniline *N*-Dealkylation....14

5.2 [3+2]-Type Cycloadditions....27

5.3 Giese Reactions....40

5.4 α-*N* sp^3 C–H Arylation....43

5.5 Addition to Imines....47

6 Mechanistic Considerations....48

6.1 Cyclic Voltammetry and Emission Quenching Studies....48

6.2 Detection of H_2....59

6.3 Detection of Formaldehyde....60

6.4 Quantum Yields (Φ) Determination....61

7 NMR Spectra....62

8 References....165

1 General Experimental Details

All required fine chemicals were used directly without purification unless stated otherwise. All air and moisture sensitive reactions were carried out under nitrogen atmosphere using standard Schlenk manifold technique. All solvents were bought from Acros as 99.8% purity. ^{1}H and ^{13}C Nuclear Magnetic Resonance (NMR) spectra were acquired at various field strengths as indicated and were referenced to $CHCl_3$ (7.27 and 77.0 ppm for ^{1}H and ^{13}C respectively). ^{1}H NMR coupling constants are reported in Hertz and refer to apparent multiplicities and not true coupling constants. Data are reported as follows: chemical shift, integration, multiplicity (s = singlet, br s = broad singlet, d = doublet, t = triplet, q = quartet, qi = quintet, sx = sextet, sp = septet, m = multiplet, dd = doublet of doublets, etc.), proton assignment (determined by 2D NMR experiments: NOESY, HSQC and HMBC) where possible. High-resolution mass spectra were obtained using a JEOL JMS-700 spectrometer or a Fissions VG Trio 2000 quadrupole mass spectrometer. Spectra were obtained using electron impact ionization (EI) and chemical ionization (CI) techniques, or positive electrospray (ES). Analytical TLC: aluminum backed plates pre-coated (0.25 mm) with Merck Silica Gel 60 F254. Compounds were visualized by exposure to UV-light or by dipping the plates in permanganate (KMnO4) stain followed by heating. Flash column chromatography was performed using Merck Silica Gel 60 (40–63 μm). All mixed solvent eluents are reported as v/v solutions. Absorption and emission spectra were obtained using an Horiba Duetta spectrometer and 1 mm High Precision Cell made of quartz from Hellma Analytics. The LEDs used are Kessil PR 160 440 nm. All the reactions were conducted in CEM 10 mL glass microwave tubes.

2 Starting Material Synthesis

GP1 – General Procedure for Synthesis of *N*-alkylanilines

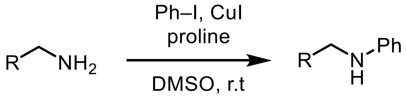

An oven-dried flask was charged with CuI (0.20 equiv.) and L-proline (0.40 equiv.). The flask was fitted with a rubber septum, then evacuated and backfilled with N_2 (x 3). The DMSO (0.5 M) and PhI (1.0 equiv.) were sequentially added. The resulting blue solution was stirred for 5 min before adding the amine (2.0 equiv.). The resulting mixture was stirred under argon at room temperature until completion of the reaction indicated by TLC analysis. Once the reaction was complete, the mixture was diluted with H_2O (20 mL) and EtOAc (20 mL). The layers were separated, and the aqueous layer was extracted with EtOAc (2 × 20 mL). The combined organic layers were washed with brine (20 mL), dried (Na_2SO_4), filtered and evaporated. The crude was purified by column chromatography on silica gel eluting with petrol–EtOAc.

N-Phenethylaniline (S1)

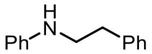

Following **GP1**, iodobenzene (0.56 mL, 5.0 mmol) and phenethylamine (1.26 mL, 10 mmol) gave **S1** (0.88 g, 89%) as an oil. ^{1}H NMR (500 MHz, $CDCl_3$) δ 7.53–7.44 (2H, m), 7.44–7.30 (5H, m), 6.89 (1H, td, *J* = 7.4, 2.6 Hz), 6.80–6.72 (2H, m), 3.71 (1H, brs), 3.53 (2H, t, *J* = 7.1 Hz), 3.05 (2H, t, *J* = 7.1 Hz); ^{13}C NMR (126 MHz, $CDCl_3$) δ 148.0, 139.4, 129.3, 128.8, 128.6, 126.4, 117.4, 113.0, 45.0, 35.5. Data in accordance with the literature.[1]

N-(2-((*tert*-Butyldimethylsilyl)oxy)ethyl)aniline (S2)

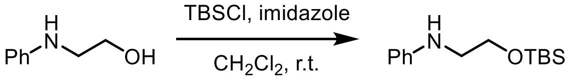

TBSCl (1.28 g, 8.02 mmol, 1.1 equiv.) and imidazole (1.45 g, 21.86 mmol, 3.0 equiv.) were added to a solution of *N*-(2-hydroxyethyl)aniline (1.00 g, 7.29 mmol, 1.0 equiv.) in CH_2Cl_2 (20 mL, 0.36 M) at room temperature. The reaction was stirred at room temperature until judged complete by TLC analysis. The mixture was diluted with sat. NH_4Cl (20 mL) and CH_2Cl_2 (20 mL). The layers were separated, and the aqueous layer was extracted with CH_2Cl_2 (2 × 20 mL). The combined organic layers were washed with brine (20 mL), dried (Na_2SO_4), filtered and evaporated. The crude was purified by column chromatography on silica gel eluting with petrol–EtOAc (15:1) to give **S2** (1.74 g, 95%) as an oil. ^{1}H NMR (400

MHz, $CDCl_3$) δ 7.25–7.15 (2H, m), 6.73 (1H, tt, *J* = 7.3, 1.1 Hz), 6.69–6.62 (2H, m), 4.09 (1H, brs), 3.84 (2H, t, *J* = 5.4 Hz), 3.24 (2H, t, *J* = 5.4 Hz), 0.93 (9H, s), 0.09 (6H, s); ^{13}C NMR (101 MHz, $CDCl_3$) δ 148.5, 129.3, 117.7, 113.4, 61.7, 46.1, 26.0, 18.5, −5.2. Data in accordance with the literature.[2]

_N_-(2-(Cyclohex-1-en-1-yl)ethyl)aniline (S3)

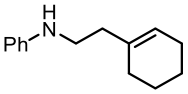

Following **GP1**, iodobenzene (1.11 mL, 10 mmol) and 2-(1-cyclohexenyl)ethylamine (2.78 mL, 20 mmol) gave **S3** (1.97 g, 98%) as yellow oil. ^{1}H NMR (500 MHz, $CDCl_3$) δ 7.24 (2H, t, *J* = 7.7 Hz), 6.76 (1H, t, *J* = 7.3 Hz), 6.67 (2H, d, *J* = 8.0 Hz), 5.61–5.58 (1H, m), 3.61 (1H, brs), 3.22 (2H, t, *J* = 6.8 Hz), 2.32 (2H, t, *J* = 6.9 Hz), 2.12–2.07 (2H, m), 2.05–1.98 (2H, m), 1.74–1.67 (2H, m), 1.67–1.60 (2H, m); ^{13}C NMR (126 MHz, $CDCl_3$) δ 148.5, 135.0, 129.3, 123.7, 117.3, 112.9, 41.5, 37.8, 28.0, 25.4, 23.0, 22.5. Data in accordance with the literature.[3]

_N_-(2-(1_H_-Imidazol-4-yl)ethyl)aniline (S4)

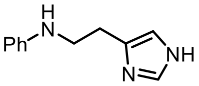

Following **GP1**, iodobenzene (0.56 mL, 5.0 mmol) and histamine (1.11 g, 10 mmol) gave **S4** (0.10 g, 11%) as an oil. ^{1}H NMR (500 MHz, $CDCl_3$) δ 7.57 (1H, s), 7.20–7.12 (2H, m), 6.84 (1H, s), 6.70 (1H, t, *J* = 7.3 Hz), 6.63–6.58 (2H, m), 6.48 (1H, brs), 3.40 (2H, t, *J* = 6.6 Hz), 2.92 (2H, t, *J* = 6.6 Hz); ^{13}C NMR (126 MHz, $CDCl_3$) δ 148.2, 135.7, 134.9, 129.4, 117.7, 117.0, 113.2, 43.8, 26.9; HRMS (ASAP) Found M−H^{+} 186.1035, $C_{11}H_{12}N_3$ requires 186.1037.

_N_-(2-(1_H_-Indol-3-yl)ethyl)aniline (S5)

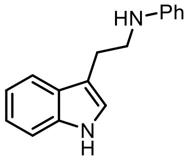

Following **GP1**, iodobenzene (0.56 mL, 5.0 mmol) and tryptamine (1.60 g, 10 mmol) gave **S5** (1.10 g, 93%) as a solid. ^{1}H NMR (500 MHz, $CDCl_3$) δ 7.84 (1H, s), 7.58 (1H, d, *J* = 7.8 Hz), 7.27 (1H, d, *J* = 8.1 Hz), 7.21–7.13 (3H, m), 7.10 (1H, t, *J* = 7.4 Hz), 6.90 (1H, s), 6.69 (1H, t, *J* = 7.4 Hz), 6.58 (2H, d, *J* = 7.9 Hz), 3.69 (1H, brs), 3.41 (2H, t, *J* = 6.8 Hz), 3.03 (2H,

t, J = 6.8 Hz); ^{13}C NMR (126 MHz, $CDCl_3$) δ 148.3, 136.4, 129.4, 127.5, 122.2 (2 x C), 119.5, 118.9, 117.5, 113.3, 113.1, 111.3, 44.0, 25.1. Data in accordance with the literature.[4]

N-(((1S,2S,5S)-6,6-Dimethylbicyclo[3.1.1]heptan-2-yl)methyl)aniline (S6)

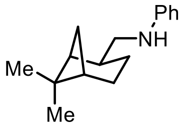

Following **GP1**, iodobenzene (0.56 mL, 5.0 mmol) and (−)-*cis*-myrtanylamine (1.20 mL, 7.5 mmol) gave **S6** (0.81 g, 71%) as yellow oil. ^{1}H NMR (500 MHz, $CDCl_3$) δ 7.25–7.17 (2H, m), 6.72 (1H, t, J = 7.3 Hz), 6.63 (2H, d, J = 8.1 Hz), 3.70 (1H, brs), 3.15 (2H, qd, J = 12.0, 7.5 Hz), 2.47–2.40 (1H, m), 2.35 (1H, ddt, J = 12.9, 7.8, 3.9 Hz), 2.10–1.89 (5H, m), 1.64–1.53 (1H, m), 1.26 (3H, s), 1.09 (3H, s), 0.97 (1H, d, J = 9.6 Hz); ^{13}C NMR (126 MHz, $CDCl_3$) δ 148.6, 129.3, 117.0, 112.7, 50.0, 44.2, 41.6, 41.2, 38.8, 33.5, 28.2, 26.3, 23.5, 20.5. Data in accordance with the literature.[5]

3 Reaction Optimizations

3.1 Aniline *N*-Demethylation

General Procedure for the Reaction Optimization – GP2

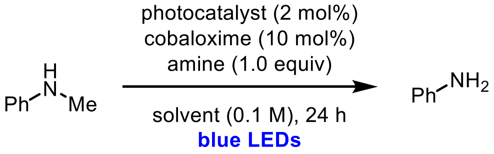

A dry tube equipped with a stirring bar was charged with the photocatalyst (2 µmol, 2 mol%), the cobaloxime (4 µmol, 4 mol%), and the amine (if solid) (0.10 mmol, 1.0 equiv.). The tube was capped with a Supelco aluminium crimp seal with septum (PTFE/butyl), then evacuated under high vacuum and backfilled with N_2 (x 3). Degassed solvent (0.1 M) and **2** (10.8 µL, 0.10 mmol, 1.0 equiv.) and the base (if liquid) (0.10 mmol, 1.0 equiv.) were sequentially added. The reaction mixture was cooled to –78 °C and degassed by three cycles of freeze, pump (5 min), backfill (with N_2), and thaw. After the reaction mixture was thoroughly degassed, the vial was sealed with parafilm and placed approximately 4 cm from blue LEDs. The blue LEDs were switched on and the mixture was stirred under irradiation without fan for 24 hours. The tube was opened, and the mixture was diluted with H_2O (0.5 mL) and EtOAc (2 mL). 1,3,5-Trimethoxybenzene (17 mg, 0.10 mmol, 1.0 equiv.) was added and the layers were separated. The aqueous layer was extracted with EtOAc (2 mL x 3) and the combined organic layers were washed with brine, dried (Na_2SO_4), filtered, and evaporated under reduced pressure (25 ºC water bath). The crude was solubilised in $CDCl_3$ (0.6 mL) and analysed by ^{1}H NMR spectroscopy to determine the ^{1}H NMR yield.

Table S1

Entry	Photocatalyst	Cobaloxime	Amine	Solvent	Yield (%)
**1**	Ir(dtbbpy)(ppy)$_2$]PF$_6$	Co(dmgH)(dmgH$_2$)Cl$_2$	Et$_3$N	CH$_3$CN	86
**2[a]**	Ir(dtbbpy)(ppy)$_2$]PF$_6$	Co(dmgH)(dmgH$_2$)Cl$_2$	Et$_3$N	CH$_3$CN	93
**3**	Ir(dtbbpy)(ppy)$_2$]PF$_6$	Co(dmgH)(dmgH$_2$)Cl$_2$	piperidine	CH$_3$CN	83
**4**	Ir(dtbbpy)(ppy)$_2$]PF$_6$	Co(dmgH)(dmgH$_2$)Cl$_2$	(*i*-Pr)$_2$NEt	CH$_3$CN	26
**5**	Ir(dtbbpy)(ppy)$_2$]PF$_6$	Co(dmgH)(dmgH$_2$)Cl$_2$	*n*-Bu$_3$N	CH$_3$CN	44
**6**	Ir(dtbbpy)(ppy)$_2$]PF$_6$	Co(dmgH)(dmgH$_2$)Cl$_2$	*i*-Bu$_3$N	CH$_3$CN	27
**7**	Ir(dtbbpy)(ppy)$_2$]PF$_6$	Co(dmgH)(dmgH$_2$)Cl$_2$	Ph$_3$N	CH$_3$CN	–
**8**	Ir(dtbbpy)(ppy)$_2$]PF$_6$	Co(dmgH)(dmgH$_2$)Cl$_2$	quinuclidine	CH$_3$CN	68
**9**	Ir(dtbbpy)(ppy)$_2$]PF$_6$	Co(dmgH)(dmgH$_2$)Cl$_2$	Cs$_2$CO$_3$	CH$_3$CN	15
**10**	Ir(dtbbpy)(ppy)$_2$]PF$_6$	Co(dmgH)(dmgH$_2$)Cl$_2$	NaHCO$_3$	CH$_3$CN	31
**11**	Ir(dtbbpy)(ppy)$_2$]PF$_6$	Co(dmgH)(dmgH$_2$)Cl$_2$	K$_3$PO$_4$	CH$_3$CN	32
**12**	Ir(dtbbpy)(ppy)$_2$]PF$_6$	Co(dmgH)(dmgH$_2$)Cl$_2$	K$_2$HPO$_4$	CH$_3$CN	27
**13**	Ir(dtbbpy)(ppy)$_2$]PF$_6$	Co(dmgH)(dmgH$_2$)Cl$_2$	NaOAc	CH$_3$CN	32
**14**	Ir(ppy)$_3$	Co(dmgH)(dmgH$_2$)Cl$_2$	Et$_3$N	CH$_3$CN	10
**15**	Ru(bpy)$_3$Cl$_2$	Co(dmgH)(dmgH$_2$)Cl$_2$	Et$_3$N	CH$_3$CN	12
**16**	4CzIPN	Co(dmgH)(dmgH$_2$)Cl$_2$	Et$_3$N	CH$_3$CN	25
**17**	(Ir[dF(CF$_3$)ppy]$_2$(dtbpy))PF$_6$	Co(dmgH)(dmgH$_2$)Cl$_2$	Et$_3$N	CH$_3$CN	57
**18**	Ir(Fppy)$_3$	Co(dmgH)(dmgH$_2$)Cl$_2$	Et$_3$N	CH$_3$CN	8
**19**	Mes-Acr•ClO$_4$	Co(dmgH)(dmgH$_2$)Cl$_2$	Et$_3$N	CH$_3$CN	trace

Entry	Photocatalyst	Cobaloxime	Amine	Solvent	Yield (%)
**20**	Ir(dtbbpy)(ppy)$_2$]PF$_6$	Co(dmgH)(dmgH$_2$)Cl$_2$	Et$_3$N	DMSO	37
**21**	Ir(dtbbpy)(ppy)$_2$]PF$_6$	Co(dmgH)(dmgH$_2$)Cl$_2$	Et$_3$N	1,4-dioxane	60
**22**	Ir(dtbbpy)(ppy)$_2$]PF$_6$	Co(dmgH)$_2$(DMAP)Cl	Et$_3$N	CH$_3$CN	56
**23**	Ir(dtbbpy)(ppy)$_2$]PF$_6$	Co(dmgH)$_2$pyCl	Et$_3$N	CH$_3$CN	46
**24**	Ir(dtbbpy)(ppy)$_2$]PF$_6$	Co(dmgH)$_2$(4-MeO-py)Cl	Et$_3$N	CH$_3$CN	55
**25**	Ir(dtbbpy)(ppy)$_2$]PF$_6$	Co(dmgH)$_2$(4-*t*Bu-py)Cl	Et$_3$N	CH$_3$CN	55
**26**	Ir(dtbbpy)(ppy)$_2$]PF$_6$	Co(dmgH)$_2$(4-CN-py)Cl	Et$_3$N	CH$_3$CN	60
**27**	Ir(dtbbpy)(ppy)$_2$]PF$_6$	Co(dppe)Br$_2$	Et$_3$N	CH$_3$CN	27
Control Experiments					
**28**	–	Co(dmgH)(dmgH$_2$)Cl$_2$	Et$_3$N	CH$_3$CN	–
**29**	Ir(dtbbpy)(ppy)$_2$]PF$_6$	–	Et$_3$N	CH$_3$CN	–
**30**	Ir(dtbbpy)(ppy)$_2$]PF$_6$	Co(dmgH)(dmgH$_2$)Cl$_2$	–	CH$_3$CN	traces
**31**[b]	Ir(dtbbpy)(ppy)$_2$]PF$_6$	Co(dmgH)(dmgH$_2$)Cl$_2$	Et$_3$N	CH$_3$CN	55
**32**[c]	Ir(dtbbpy)(ppy)$_2$]PF$_6$	Co(dmgH)(dmgH$_2$)Cl$_2$	Et$_3$N	CH$_3$CN	–
(a) reaction run with 15 mol% of Co(dmgH)(dmgH$_2$)Cl$_2$; (b) reaction run under air; (c) reaction run in the dark					

Entry	Photocatalyst	Cobaloxime	Amine	Solvent	Yield (%)
	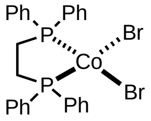<br>Co(dppe)Br_2	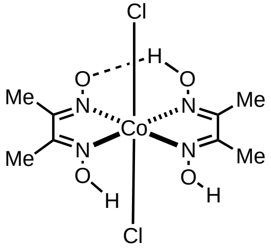<br>Co(dmgH)($dmgH_2$)Cl_2<br>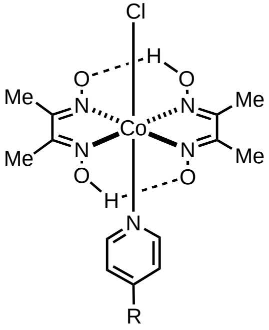<br>R = H Co$(dmgH)_2$pyCl<br>R = NMe_2 Co$(dmgH)_2$DMAPCl<br>R = OMe Co$(dmgH)_2$(4-MeO-py)Cl<br>R = *t*-Bu Co$(dmgH)_2$(4-*t*Bu-py)Cl<br>R = CN Co$(dmgH)_2$(4-CN-py)Cl			

3.2 [3+2]-Type Cycloadditions

General Procedure for the Reaction Optimization – GP3

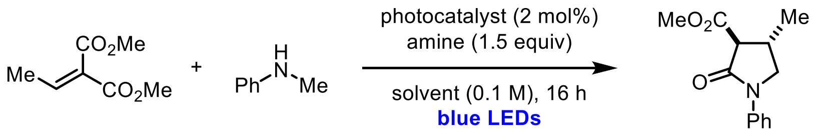

A dry tube equipped with a stirring bar was charged with the photocatalyst (2 µmol, 2 mol%), and the amine (if solid) (0.15 mmol, 1.5 equiv.). The tube was capped with a Supelco aluminium crimp seal with septum (PTFE/butyl), then evacuated under high vacuum and backfilled with N_2 (x 3). Degassed solvent (0.1 M), dimethyl 2-ethylidenemalonate (14 µL, 0.1 mmol, 1.0 equiv.), *N*-methylaniline (16 µL, 0.15 mmol, 1.5 equiv.), and the amine (if liquid) (0.15 mmol, 1.5 equiv.) were sequentially added. The vial was purged with a stream of N_2 and the lid sealed with parafilm and placed approximately 4 cm from blue LEDs. The blue LEDs were switched on and the mixture was stirred under irradiation without fan for 16 hours. The tube was opened, 1,3,5-trimethoxybenzene (17 mg, 0.1 mmol, 1.0 equiv.) was added and the solvent was evaporated under reduced pressure. The crude was solubilised in $CDCl_3$ (0.6 mL) and analysed by ^1H NMR spectroscopy to determine the ^1H NMR yield.

Table S2.

Entry	Photocatalyst	Amine	Solvent	Yield (%)
**1**	$Ir(dtbbpy)(ppy)_2]PF_6$	piperidine	CH_3CN	67
**2**	$Ir(dtbbpy)(ppy)_2]PF_6$	DABCO	CH_3CN	70
**3**	$Ir(dtbbpy)(ppy)_2]PF_6$	DABCO	NMP	30
**4**	$Ir(dtbbpy)(ppy)_2]PF_6$	DABCO	DMSO	40
**5**	$Ru(bpy)_3Cl_2$	DABCO	CH_3CN	7
**6**	$Ir(dtbbpy)(ppy)_2]PF_6$	quinuclidine	CH_3CN	80
**7**	$Ir(dtbbpy)(ppy)_2]PF_6$	3-quinuclidinol	CH_3CN	57
**8**	$Ir(dtbbpy)(ppy)_2]PF_6$	NaOAc	CH_3CN	9
**9**	$Ir(dtbbpy)(ppy)_2]PF_6$	K_2HPO_4	CH_3CN	14

In order to further optimise the reaction, we then used the same procedure but $Ir(dtbbpy)(ppy)_2]PF_6$ (1 µmol, 1 mol%) evaluated the equivalents of quinuclidine that proved optimum (Table S3, entry 3).

Table S3.

Entry	Quinuclidine equiv.	Yield (%)
**1**	1.5	77
**2**	1.0	81
**3**	0.5	95
**4**	0.2	62
Control Experiments		
**6**[a]	0.5	–
**7**	–	–
**8**[b]	0.5	74
**9**[c]	0.5	–

(a) the reaction was run without Ir(dtbbpy)$(ppy)_2$]PF_6; (b) the reaction was run under air; (c) the reaction was run in the dark.

3.3 α-*N* sp^3 C–H Arylation

General Procedure for the Reaction Optimization – GP4

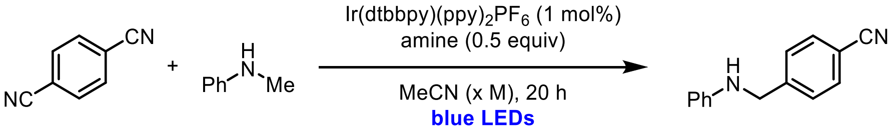

A dry tube equipped with a stirring bar was charged with Ir(dtbbpy)(ppy)$_2$]PF_6 (2 mg, 2 µmol, 1 mol%), 1,4-dicyanobenzene (26 mg, 0.2 mmol, 1.0 equiv.) and the amine (if solid) (0.1 mmol, 0.5 equiv.). The tube was capped with a Supelco aluminium crimp seal with septum (PTFE/butyl), then evacuated under high vacuum and backfilled with N_2 (x 3). Degassed CH_3CN, *N*-methylaniline (32.5 µL, 0.3 mmol, 1.5 equiv.), and the amine (if liquid) (0.1 mmol, 0.5 equiv.) were sequentially added. The vial was purged with a stream of N_2 and the lid sealed with parafilm and placed approximately 4 cm from blue LEDs. The blue LEDs were switched on and the mixture was stirred under irradiation without fan for 20 hours. The tube was opened, 1,3,5-trimethoxybenzene (17 mg, 0.1 mmol, 1.0 equiv.) was added and the solvent was evaporated under reduced pressure. The crude was solubilised in $CDCl_3$ (0.6 mL) and analysed by ^{1}H NMR spectroscopy to determine the ^{1}H NMR yield.

Table S4.

Entry	Amine	[Solvent] (M)	Yield (%)
**1**	quinuclidine	0.2	27
**2**	quinuclidine	0.1	49
**3**	Et_3N	0.1	18
**4**	3-quinuclidinol	0.1	20
**5**	quinuclidine	0.06	50
**6**	quinuclidine	0.05	68

4 Picture of Reaction Set-Up

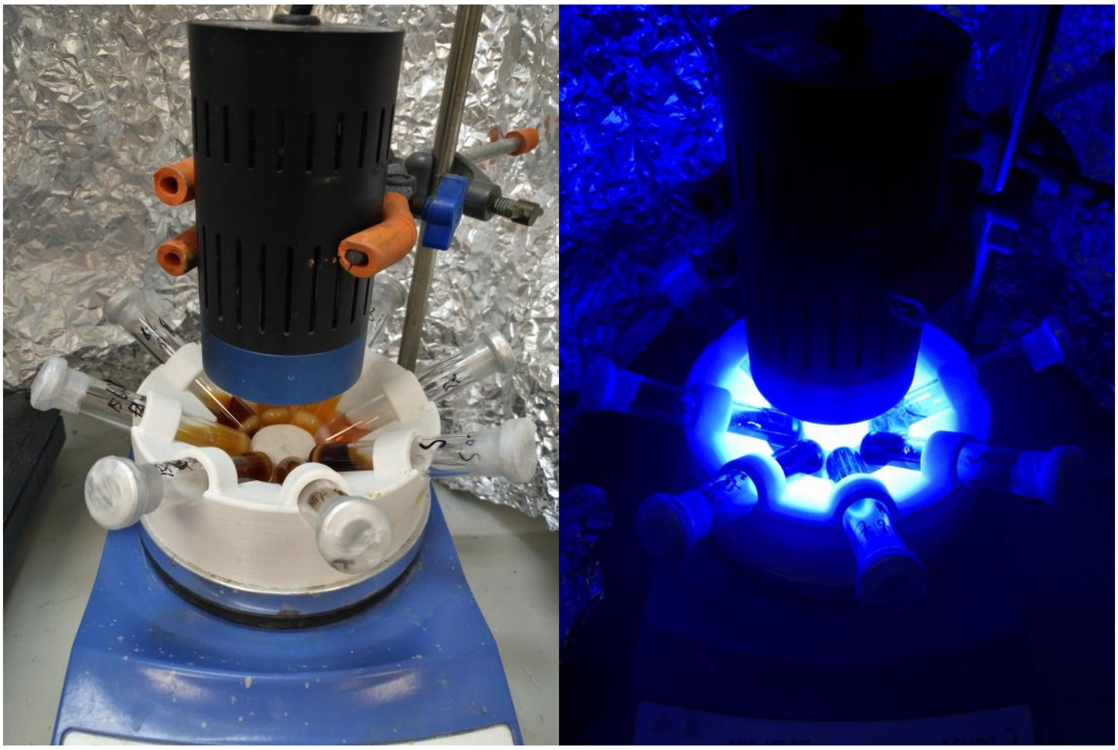

Figure S1

Once the LEDs were turned on the entire reaction set up was wrapped in aluminium foil. In this way the reactions temperature remained uniform at around 40-45 ºC.

5 Substrate Scope

5.1 Aniline *N*-Dealkylation

General Procedure for the Aniline *N*-Dealkylation Reaction– GP5

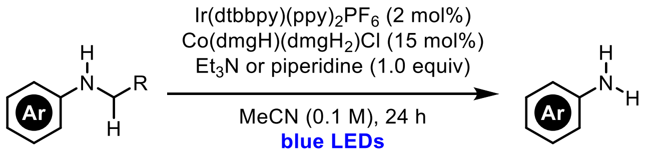

A dry tube equipped with a stirring bar was charged with Ir(dtbbpy)(ppy)$_2$PF$_6$ (4 mg, 4 µmol, 2 mol%), Co(dmgH)(dmgH$_2$)Cl$_2$ (11 mg, 0.03 mmol, 15 mol%), and the *N*-alkyl aniline (if solid) (0.20 mmol, 1.0 equiv.). The tube was capped with a Supelco aluminium crimp seal with septum (PTFE/butyl), then evacuated under high vacuum and backfilled with N_2 (x 3). Degassed CH_3CN (2.0 mL, 0.1 M), the *N*-alkyl aniline (if liquid) (0.20 mmol, 1.0 equiv.) and Et_3N (28 µL, 0.20 mmol, 1.0 equiv.) or piperidine (18 µL, 0.20 mmol, 1.0 equiv.) were sequentially added. The reaction mixture was cooled to –78 °C and degassed by three cycles of freeze, pump (5 min), backfill (with N_2), and thaw. After the reaction mixture was thoroughly degassed, the vial was sealed with parafilm and placed approximately 4 cm from blue LEDs. The blue LEDs were switched on and the mixture was stirred under irradiation without fan for 24 hours. The tube was opened and the mixture was diluted with H_2O (10 mL) and EtOAc (10 mL). The layers were separated and the aqueous layer was extracted with EtOAc (2 × 10 mL). The combined organic layers were washed with brine (10 mL), dried (Na_2SO_4), filtered and evaporated. The crude was purified by flash column chromatography on silica gel with hexane–EtOAc.

Aniline (3)

Ph–NH$_2$

• Following **GP5**, Et_3N and *N*-methylaniline **2** (22 µL, 0.20 mmol) gave **3** (93% NMR yield). ^{1}H NMR (500 MHz, $CDCl_3$) δ 7.22–7.13 (2H, m), 6.82–6.75 (1H, m), 6.74–6.67 (2H, m), 3.65 (2H, brs); ^{13}C NMR (126 MHz, $CDCl_3$) δ 146.5, 129.4, 118.6, 115.2. Data in accordance with the literature.[6]

• Following **GP5**, piperidine and *N*-methylaniline **2** (22 µL, 0.20 mmol) gave **3** (91% NMR yield).

• Following **GP5**, Et_3N and *N*-benzylaniline **33** (37 mg, 0.20 mmol) gave **3** (43% NMR yield).

• Following **GP5**, piperidine (2.0 equiv) and *N*-benzylaniline **33** (37 mg, 0.20 mmol) gave **3** (88% NMR yield).

• Following **GP5**, Et_3N and *N*-ethylaniline **34** (25 μL, 0.20 mmol) gave **3** (quant. NMR yield).
• Following **GP5**, piperidine and *N*-ethylaniline **34** (25 μL, 0.20 mmol) gave **3** (95% NMR yield).

• Following **GP5**, Et_3N and *N*-butylaniline **35** (32 μL, 0.20 mmol) gave **3** (75% NMR yield).
• Following **GP5**, piperidine and *N*-butylaniline **35** (32 μL, 0.20 mmol) gave **3** (quant. NMR yield).

• Following **GP5**, Et_3N and *N*-isopropylaniline **36** (29 μL, 0.20 mmol) gave **3** (24% NMR yield).
• Following **GP5**, piperidine and *N*-isopropylaniline **36** (29 μL, 0.20 mmol) gave **3** (quant. NMR yield).

• Following **GP5** but stirring the reaction for 48 h, Et_3N and *N*-cyclohexylaniline **37** (35 μL, 0.20 mmol) gave **3** (11% NMR yield).
• Following **GP5** but stirring the reaction for 48 h, piperidine and *N*-cyclohexylaniline **37** (35 μL, 0.20 mmol) gave **3** (65% NMR yield).

• Following **GP5** but stirring the reaction for 48 h, Et_3N and *N*-(2-hydroxyethyl)aniline **39** (25 μL, 0.20 mmol) gave **3** (63% NMR yield).
• Following **GP5**, piperidine and *N*-(2-hydroxyethyl)aniline **39** (25 μL, 0.20 mmol) gave **3** (92% NMR yield).

• Following **GP5**, Et_3N and 3-anilinopropionitrile **40** (29 mg, 0.20 mmol) gave **3** (82% NMR yield).

• Following **GP5**, Et_3N and *N,N'*-diphenylethylenediamine **41** (21 mg, 0.10 mmol) gave **3** (70% NMR yield).
• Following **GP5**, piperidine and *N,N'*-diphenylethylenediamine **41** (21 mg, 0.10 mmol) gave **3** (80% NMR yield).

• Following **GP5** but stirring the reaction for 48 h, Et_3N and *N*,*N*-dimethylaniline **1** (26 μL, 0.10 mmol) gave **3** (76% NMR yield).

• Following **GP5** but stirring the reaction for 48 h, piperidine and *N*,*N*-dimethylaniline **1** (26 µL, 0.10 mmol) gave **3** (58% NMR yield).

***p*-Toluidine (5)**

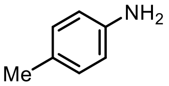

• Following **GP5**, Et_3N and *N*-methyl-*p*-toluidine (25 µL, 0.20 mmol) gave **5** (18.5 mg, 86%) as a solid. ^{1}H NMR (400 MHz, $CDCl_3$) δ 7.02–6.90 (2H, m), 6.67–6.57 (2H, m), 3.53 (2H, brs), 2.24 (3H, s); ^{13}C NMR (101 MHz, $CDCl_3$) δ 143.9, 129.9, 127.9, 115.3, 20.6. Data in accordance with the literature.[6]

• Following **GP5**, piperidine and *N*-methyl-*p*-toluidine (25 µL, 0.20 mmol) gave **5** (18 mg, 85%) as a solid.

***p*-Anisidine (6)**

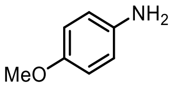

• Following **GP5**, Et_3N and 4-methoxy-*N*-methylaniline (27.5 mg, 0.20 mmol) gave **6** (15.5 mg, 62%) as a solid. ^{1}H NMR (400 MHz, $CDCl_3$) δ 6.84–6.70 (2H, m), 6.70–6.60 (2H, m), 3.75 (3H, s), 3.43 (2H, brs); ^{13}C NMR (101 MHz, $CDCl_3$) δ 152.8, 140.1, 116.5, 114.9, 55.8. Data in accordance with the literature.[7]

• Following **GP5**, piperidine and 4-methoxy-*N*-methylaniline (27.5 mg, 0.20 mmol) gave **6** (15.5 mg, 62%) as a solid.

4-(Trifluoromethoxy)aniline (7)

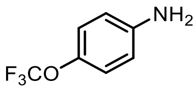

• Following **GP5**, Et_3N and *N*-methyl-4-(trifluoromethoxy)aniline (30 µL, 0.20 mmol) gave **7** (15.5 mg, 44%) as an oil. ^{1}H NMR (400 MHz, $CDCl_3$) δ 7.04–6.97 (2H, m), 6.67–6.59 (2H, m), 3.69 (2H, brs); ^{13}C NMR (126 MHz, $CDCl_3$) δ 145.4, 141.4 (q, J = 2.2 Hz), 122.5, 120.8 (q, J = 255.5 Hz), 115.6; ^{19}F NMR (376 MHz, $CDCl_3$) δ −58.5. Data in accordance with the literature.[8]

• Following **GP5**, piperidine and *N*-methyl-4-(trifluoromethoxy)aniline (30 µL, 0.20 mmol) gave **7** (26 mg, 73%) as an oil.

4-Fluoroaniline (8)

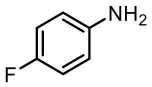

• Following **GP5**, Et_3N and 4-fluoro-*N*-methylaniline (24 µL, 0.20 mmol) gave **8** (20.5 mg, 92%) as an oil. ^{1}H NMR (400 MHz, $CDCl_3$) δ 6.92–6.72 (2H, m), 6.69–6.52 (2H, m), 3.54 (2H, brs); ^{13}C NMR (126 MHz, $CDCl_3$) δ 156.5 (d, *J* = 235.7 Hz), 142.5 (d, *J* = 2.0 Hz), 116.2 (d, *J* = 7.5 Hz), 115.8 (d, *J* = 22.5 Hz); ^{19}F NMR (376 MHz, $CDCl_3$) δ −126.84. Data in accordance with the literature.[7]

• Following **GP5**, piperidine and 4-fluoro-*N*-methylaniline (24 µL, 0.20 mmol) gave **8** (20 mg, 88%) as an oil.

4-Chloroaniline (9)

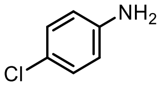

• Following **GP5**, Et_3N and 4-chloro-*N*-methylaniline (24 µL, 0.20 mmol) gave **9** (20.5 mg, 80%) as a solid. ^{1}H NMR (500 MHz, $CDCl_3$) δ 7.17–7.07 (2H, m), 6.69–6.57 (2H, m), 3.67 (2H, brs); ^{13}C NMR (101 MHz, $CDCl_3$) δ 145.1, 129.2, 123.3, 116.3. Data in accordance with the literature.[7]

• Following **GP5** but stirring the reaction for 40 h, piperidine and 4-chloro-*N*-methylaniline (24 µL, 0.20 mmol) gave **9** (18.5 mg, 72%) as a solid.

4-Bromoaniline (10)

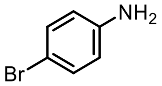

Following **GP5** but stirring the reaction for 40 h, Et_3N and 4-bromo-*N*-methylaniline (25 µL, 0.20 mmol) gave **10** (13 mg, 37%) as a solid. ^{1}H NMR (500 MHz, $CDCl_3$) δ 7.25–7.20 (2H, m), 6.59–6.52 (2H, m), 3.66 (2H, brs); ^{13}C NMR (126 MHz, $CDCl_3$) δ 145.5, 132.1, 116.8, 110.3. Data in accordance with the literature.[6]

• Following **GP5** but stirring the reaction for 40 h, piperidine and 4-bromo-*N*-methylaniline (25 µL, 0.20 mmol) gave **10** (17.5 mg, 51%) as a solid.

4-Iodoaniline (11)

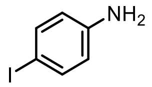

• Following **GP5** but stirring the reaction for 40 h, Et_3N and 4-iodo-*N*-methylaniline (46.5 mg, 0.20 mmol) gave **11** (10 mg, 22%) as a solid. ^1H NMR (500 MHz, $CDCl_3$) δ 7.44–7.37 (2H, m), 6.52–6.43 (2H, m), 3.65 (2H, brs); ^{13}C NMR (126 MHz, $CDCl_3$) δ 146.2, 138.0, 117.4, 79.5. Data in accordance with the literature.[8]

• Following **GP5** but stirring the reaction for 40 h, piperidine and 4-iodo-*N*-methylaniline (46.5 mg, 0.20 mmol) gave **11** (28.5 mg, 65%) as a solid.

***m*-Toluidine (12)**

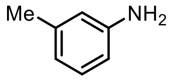

• Following **GP5**, Et_3N (1.0 equiv) and 3,*N*-dimethylaniline (25 µL, 0.20 mmol) gave **12** (21.5 mg, quant.) as an oil. ^1H NMR (500 MHz, $CDCl_3$) δ 7.07 (1H, t, J = 7.6 Hz), 6.61 (1H, d, J = 7.1 Hz), 6.56–6.49 (2H, m), 3.60 (2H, brs), 2.29 (3H, s); ^{13}C NMR (126 MHz, $CDCl_3$) δ 146.4, 139.2, 129.3, 119.5, 116.0, 112.3, 21.5. Data in accordance with the literature.[6]

• Following **GP5**, piperidine and 3,*N*-dimethylaniline (25 µL, 0.20 mmol) gave **12** (21 mg, 97%) as an oil.

***m*-Anisidine (13)**

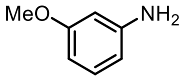

• Following **GP5**, Et_3N and 3-methoxy-*N*-methylaniline (26 µL, 0.20 mmol) gave **13** (23 mg, 94%) as an oil. ^1H NMR (500 MHz, $CDCl_3$) δ 7.08 (1H, t, J = 8.1 Hz), 6.34 (1H, ddd, J = 8.1, 2.4, 0.8 Hz), 6.30 (1H, ddd, J = 7.9, 2.2, 0.8 Hz), 6.25 (1H, t, J = 2.3 Hz), 3.77 (3H, s), 3.68 (2H, brs); ^{13}C NMR (126 MHz, $CDCl_3$) δ 160.8, 147.9, 130.2, 108.0, 104.0, 101.1, 55.1. Data in accordance with the literature.[9]

• Following **GP5**, piperidine and 3-methoxy-*N*-methylaniline (26 µL, 0.20 mmol) gave **13** (20 mg, 81%) as an oil.

3-Fluoroaniline (14)

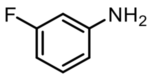

• Following **GP5**, Et_3N and 3-fluoro-*N*-methylaniline (22.5 µL, 0.20 mmol) gave **14** (7 mg, 30%) as an oil. ^{1}H NMR (500 MHz, $CDCl_3$) δ 7.08 (1H, td, *J* = 8.1, 6.6 Hz), 6.48–6.41 (2H, m), 6.38 (1H, dt, *J* = 10.9, 2.3 Hz), 3.75 (2H, brs); ^{13}C NMR (126 MHz, $CDCl_3$) δ 164.0 (d, *J* = 243.2 Hz), 148.3 (d, *J* = 10.8 Hz), 130.6 (d, *J* = 9.9 Hz), 110.8 (d, *J* = 2.4 Hz), 105.2 (d, *J* = 21.3 Hz), 102.1 (d, *J* = 24.7 Hz); ^{19}F NMR (376 MHz, $CDCl_3$) δ −113.2. Data in accordance with the literature.[10]

• Following **GP5**, piperidine and 3-fluoro-*N*-methylaniline (22.5 µL, 0.20 mmol) gave **14** (8.5 mg, 38%) as an oil.

3-Chloroaniline (15)

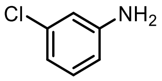

• Following **GP5**, Et_3N and 3-chloro-*N*-methylaniline (24.5 µL, 0.20 mmol) gave **15** (8 mg, 31%) as an oil. ^{1}H NMR (400 MHz, $CDCl_3$) δ 7.07 (1H, t, *J* = 8.0 Hz), 6.73 (1H, ddd, *J* = 7.9, 2.1, 0.9 Hz), 6.67 (1H, t, *J* = 2.1 Hz), 6.54 (1H, ddd, *J* = 8.0, 2.3, 0.9 Hz), 3.72 (2H, brs); ^{13}C NMR (101 MHz, $CDCl_3$) δ 147.7, 134.9, 130.4, 118.5, 115.0, 113.3. Data in accordance with the literature.[7]

• Following **GP5**, piperidine and 3-chloro-*N*-methylaniline (24.5 µL, 0.20 mmol) gave **15** (9.5 mg, 37%) as an oil.

***o*-Toluidine (16)**

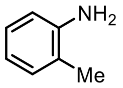

• Following **GP5**, Et_3N and *N*-methyl-*o*-toluidine (25 µL, 0.20 mmol) gave **16** (21.5 mg, quant.) as an oil. ^{1}H NMR (400 MHz, $CDCl_3$) δ 7.17–7.08 (2H, m), 6.79 (1H, td, *J* = 7.4, 1.2 Hz), 6.73 (1H, d, *J* = 7.8 Hz), 3.63 (2H, brs), 2.23 (3H, s); ^{13}C NMR (101 MHz, $CDCl_3$) δ 144.6, 130.5, 127.0, 122.3, 118.6, 114.9, 17.4. Data in accordance with the literature.[6]

• Following **GP5**, piperidine and *N*-methyl-*o*-toluidine (25 µL, 0.20 mmol) gave **16** (21 mg, 98%) as an oil.

2-Ethylaniline (17)

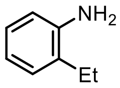

• Following **GP5**, Et_3N and 2-ethyl-*N*-methylaniline (27 mg, 0.20 mmol) gave **17** (24 mg, quant.) as an oil. ^{1}H NMR (400 MHz, $CDCl_3$) δ 7.17–7.05 (2H, m), 6.81 (1H, td, *J* = 7.5, 1.3 Hz), 6.72 (1H, dd, *J* = 7.8, 1.3 Hz), 3.63 (2H, brs), 2.57 (2H, q, *J* = 7.5 Hz), 1.31 (3H, t, *J* = 7.5 Hz); ^{13}C NMR (101 MHz, $CDCl_3$) δ 144.0, 128.4, 128.1, 126.9, 118.9, 115.4, 24.1, 13.1. Data in accordance with the literature.[11]

• Following **GP5**, piperidine and 2-ethyl-*N*-methylaniline (27 mg, 0.20 mmol) gave **17** (22 mg, 92%) as an oil.

2-Isopropylaniline (18)

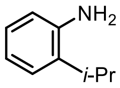

• Following **GP5**, Et_3N and 2-isopropyl-*N*-methylaniline (30 mg, 0.20 mmol) gave **18** (27 mg, quant.) as an oil. ^{1}H NMR (400 MHz, $CDCl_3$) δ 7.17 (1H, dd, *J* = 7.7, 1.5 Hz), 7.04 (1H, td, *J* = 7.6, 1.5 Hz), 6.81 (1H, td, *J* = 7.5, 1.3 Hz), 6.70 (1H, dd, *J* = 7.9, 1.3 Hz), 3.69 (2H, brs), 2.93 (1H, hept, *J* = 6.8 Hz), 1.29 (6H, d, *J* = 6.8 Hz); ^{13}C NMR (101 MHz, $CDCl_3$) δ 143.3, 132.8, 126.6, 125.5, 119.2, 116.0, 27.8, 22.4. Data in accordance with the literature.[9]

• Following **GP5**, piperidine and 2-isopropyl-*N*-methylaniline (30 mg, 0.20 mmol) gave **18** (24 mg, 90%) as an oil.

2-(*tert*-Butyl)aniline (19)

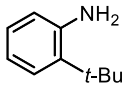

• Following **GP5**, Et_3N and 2-(*tert*-butyl)-*N*-methylaniline (33 mg, 0.20 mmol) gave **19** (30 mg, quant.) as an oil. ^{1}H NMR (400 MHz, $CDCl_3$) δ 7.27 (1H, dd, *J* = 7.8, 1.5 Hz), 7.07 (1H, ddd, *J* = 7.8, 7.2, 1.5 Hz), 6.78 (1H, ddd, *J* = 7.8, 7.2, 1.4 Hz), 6.68 (1H, dd, *J* = 7.8, 1.4 Hz), 3.83 (2H, brs), 1.46 (9H, s); ^{13}C NMR (101 MHz, $CDCl_3$) δ 144.7, 133.9, 127.1, 126.7, 118.8, 117.9, 34.4, 29.7; HRMS (ASAP) Found MH^+ 150.1275, $C_{10}H_{16}N$ requires 150.1277.

• Following **GP5**, piperidine and 2-(*tert*-butyl)-*N*-methylaniline (33 mg, 0.20 mmol) gave **19** (28.5 mg, 95%) as an oil.

2-Fluoroaniline (20)

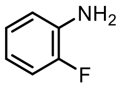

• Following **GP5**, Et_3N and 2-fluoro-*N*-methylaniline (23 µL, 0.20 mmol) gave **20** (13 mg, 57%) as an oil. ^{1}H NMR (500 MHz, $CDCl_3$) δ 6.98 (1H, ddd, *J* = 11.3, 8.1, 1.4 Hz), 6.93 (1H, td, *J* = 7.7, 1.3 Hz), 6.78 (1H, ddd, *J* = 9.3, 7.9, 1.7 Hz), 6.73–6.66 (1H, m), 3.71 (2H, brs); ^{13}C NMR (126 MHz, $CDCl_3$) δ 151.9 (d, *J* = 238.6 Hz), 134.6 (d, *J* = 12.7 Hz), 124.6 (d, *J* = 3.5 Hz), 118.8 (d, *J* = 6.8 Hz), 117.1 (d, *J* = 3.5 Hz), 115.4 (d, *J* = 18.3 Hz); ^{19}F NMR (376 MHz, $CDCl_3$) δ −135.4. Data in accordance with the literature.[9]

• Following **GP5**, piperidine and 2-fluoro-*N*-methylaniline (23 µL, 0.20 mmol) gave **20** (12 mg, 55%) as an oil.

2-Chloroaniline (21)

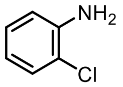

• Following **GP5**, Et_3N and 2-chloro-*N*-methylaniline (24.5 µL, 0.20 mmol) gave **21** (11.5 mg, 45%) as an oil. ^{1}H NMR (400 MHz, $CDCl_3$) δ 7.26 (1H, dd, *J* = 8.0, 1.5 Hz), 7.08 (1H, td, *J* = 7.7, 1.5 Hz), 6.77 (1H, dd, *J* = 8.0, 1.5 Hz), 6.70 (1H, td, *J* = 7.7, 1.5 Hz), 4.04 (2H, brs); ^{13}C NMR (101 MHz, $CDCl_3$) δ 143.0, 129.5, 127.7, 119.4, 119.1, 116.0. Data in accordance with the literature.[10]

• Following **GP5**, piperidine and 2-chloro-*N*-methylaniline (24.5 µL, 0.20 mmol) gave **21** (11.5 mg, 45%) as an oil.

Methyl 2-aminobenzoate (22)

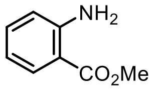

• Following **GP5** but using 4CzIPN as the photocatalyst and stirring the reaction for 72 h, Et_3N and methyl *N*-methylanthranilate (29 µL, 0.20 mmol) gave **22** (12.5 mg, 41%) as an oil. ^{1}H NMR (500 MHz, $CDCl_3$) δ 7.85 (1H, dd, *J* = 8.0, 1.6 Hz), 7.30–7.22 (1H, m), 6.69–6.60 (2H, m), 5.71 (2H, brs), 3.86 (3H, s); ^{13}C NMR (126 MHz, $CDCl_3$) δ 168.7, 150.5, 134.2, 131.3, 116.8, 116.4, 110.9, 51.7. Data in accordance with the literature.[12]

• Following **GP5** but using 4CzIPN as the photocatalyst and stirring the reaction for 72 h, piperidine and methyl *N*-methylanthranilate (29.3 µL, 0.20 mmol) gave **22** (20 mg, 66%) as an oil.

2,6-Dimethylaniline (23)

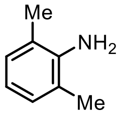

• Following **GP5**, Et_3N and *N*,2,6-trimethylaniline (27 mg, 0.20 mmol) gave **23** (24 mg, quant.) as an oil. ^{1}H NMR (400 MHz, $CDCl_3$) δ 7.05 (2H, d, *J* = 7.4 Hz), 6.75 (1H, t, *J* = 7.4 Hz), 3.63 (2H, brs), 2.27 (6H, s); ^{13}C NMR (101 MHz, $CDCl_3$) δ 142.8, 128.3, 121.7, 118.0, 17.7. Data in accordance with the literature.[8]

• Following **GP5**, piperidine and *N*,2,6-trimethylaniline (27 mg, 0.20 mmol) gave **23** (23 mg, 96%) as an oil.

2,4,6-Trimethylaniline (24)

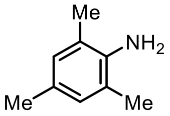

• Following **GP5**, Et_3N and 2,4,6-trimethyl-*N*-methylaniline (32 µL, 0.20 mmol) gave **24** (27 mg, quant.) as an oil. ^{1}H NMR (400 MHz, $CDCl_3$) δ 6.81 (2H, s), 3.48 (2H, brs), 2.25 (3H, s), 2.20 (6H, s); ^{13}C NMR (101 MHz, $CDCl_3$) δ 140.2, 128.9, 127.2, 121.9, 20.5, 17.7. Data in accordance with the literature.[13]

• Following **GP5**, piperidine and 2,4,6-trimethyl-*N*-methylaniline (32 µL, 0.20 mmol) gave **24** (27 mg, quant.) as an oil.

Methyl 2-amino-4,5-dimethoxybenzoate (25)

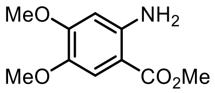

• Following **GP5** but stirring the reaction for 72 h, Et_3N and methyl 4,5-dimethoxy-2-(methylamino)benzoate (45 mg, 0.20 mmol) gave **25** (29 mg, 69%) as a solid. ^{1}H NMR (500 MHz, $CDCl_3$) δ 7.27 (1H, s), 6.13 (1H, s), 5.54 (2H, brs), 3.84 (3H, s), 3.83 (3H, s), 3.81 (3H, s); ^{13}C NMR (126 MHz, $CDCl_3$) δ 168.2, 154.8, 147.1, 140.7, 112.5, 102.1, 99.4, 56.4, 55.8, 51.4. Data in accordance with the literature.[14]

• Following **GP5** but stirring the reaction for 72 h, piperidine and methyl 4,5-dimethoxy-2-(methylamino)benzoate (45 mg, 0.20 mmol) gave **25** (40.5 mg, 96%) as a solid.

3,4-Dichloroaniline (26)

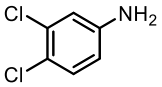

Following **GP5** but stirring the reaction for 40 h, piperidine and 3,4-dichloro-*N*-methylaniline (25.5 μL, 0.20 mmol) gave **26** (14.5 mg, 45%) as a solid. ^{1}H NMR (500 MHz, $CDCl_3$) δ 7.17 (1H, d, *J* = 8.6 Hz), 6.76 (1H, d, *J* = 2.5 Hz), 6.50 (1H, dd, *J* = 8.6, 2.5 Hz), 3.72 (2H, brs); ^{13}C NMR (126 MHz, $CDCl_3$) δ 146.1, 132.8, 130.8, 121.2, 116.5, 114.7. Data in accordance with the literature.[15]

Naphthalen-1-amine (27)

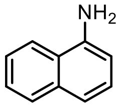

• Following **GP5**, Et_3N and *N*-methyl-*N*-1-naphthylamine (31.5 mg, 0.20 mmol) gave **27** (14 mg, 48%) as a solid. ^{1}H NMR (400 MHz, $CDCl_3$) δ 7.87–7.76 (2H, m), 7.50–7.41 (2H, m), 7.35–7.27 (2H, m), 6.79 (1H, dd, *J* = 6.8, 1.6 Hz), 4.15 (2H, brs); ^{13}C NMR (101 MHz, $CDCl_3$) δ 142.2, 134.4, 128.6, 126.4, 125.9, 124.9, 123.7, 120.9, 119.0, 109.7. Data in accordance with the literature.[9]

• Following **GP5**, piperidine and *N*-methyl-*N*-1-naphthylamine (31.5 mg, 0.20 mmol) gave **27** (24 mg, 84%) as a solid.

• Following **GP5**, Et_3N and *N*-ethyl-1-naphthylamine **44** (32 μL, 0.20 mmol) gave **27** (23 mg, 80%) as a solid.

• Following **GP5**, piperidine and *N*-ethyl-1-naphthylamine **44** (32 μL, 0.20 mmol) gave **27** (27 mg, 94%) as a solid.

Pyridin-3-amine (28)

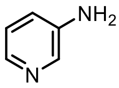

• Following **GP5** but using 4CzIPN as the photocatalyst and stirring the reaction for 72 h, Et_3N and *N*-methylpyridin-3-amine (21.5 mg, 0.20 mmol) gave **28** (7.5 mg, 40%) as a solid. ^{1}H NMR (500 MHz, $CDCl_3$) δ 8.05 (1H, d, *J* = 2.8 Hz), 7.97 (1H, d, *J* = 4.6 Hz), 7.03 (1H, dd, *J* = 8.2, 4.6 Hz), 6.97–6.88 (1H, m), 3.74 (2H, brs); ^{13}C NMR (126 MHz, $CDCl_3$) δ 142.7, 139.9, 137.5, 123.8, 121.5. Data in accordance with the literature.[11]

• Following **GP5** but using 4CzIPN as the photocatalyst and stirring the reaction for 72 h, 4CzIPN (2 mol%), piperidine and *N*-methylpyridin-3-amine (21.5 mg, 0.20 mmol) gave **28** (11 mg, 60%) as a solid.

Pyridin-2-amine (29)

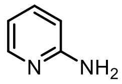

• Following **GP5** but using 4CzIPN as the photocatalyst and stirring the reaction for 72 h, Et_3N and 2-(methylamino)pyridine (21.5 mg, 0.20 mmol) gave **29** (5 mg, 25%) as a solid. ^{1}H NMR (500 MHz, $CDCl_3$) δ 8.07–8.02 (1H, m), 7.43–7.34 (1H, m), 6.64–6.56 (1H, m), 6.50–6.44 (1H, m), 4.52 (2H, brs); ^{13}C NMR (126 MHz, $CDCl_3$) δ 158.6, 148.2, 137.8, 114.0, 108.7. Data in accordance with the literature.[16]

• Following **GP5** but using 4CzIPN as the photocatalyst and stirring the reaction for 72 h, piperidine and 2-(methylamino)pyridine (21.5 mg, 0.20 mmol) gave **29** (8.5 mg, 45%) as a solid.

6-Methoxypyridin-2-amine (30)

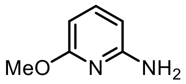

• Following **GP5** but stirring the reaction for 72 h, Et_3N and 6-methoxy-*N*-methylpyridin-2-amine (25.5 µL, 0.20 mmol) gave **30** (14 mg, 57%) as an oil. ^{1}H NMR (500 MHz, $CDCl_3$) δ 7.34 (1H, t, *J* = 7.8 Hz), 6.09 (1H, dd, *J* = 7.9, 0.6 Hz), 6.06 (1H, dd, *J* = 7.7, 0.6 Hz), 4.29 (2H, brs), 3.84 (3H, s); ^{13}C NMR (126 MHz, $CDCl_3$) δ 163.9, 157.3, 140.4, 99.8, 98.7, 53.4. Data in accordance with the literature.[17]

• Following **GP5** but stirring the reaction for 72 h, piperidine and 6-methoxy-*N*-methylpyridin-2-amine (25.5 µL, 0.20 mmol) gave **30** (15 mg, 61%) as an oil.

5-Aminobenzo[*b*]thiophene (31)

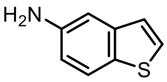

• Following **GP5**, Et_3N and *N*-methylbenzo[*b*]thiophen-5-amine (32.5 mg, 0.20 mmol) gave **31** (19.5 mg, 65%) as a solid. ^{1}H NMR (500 MHz, $CDCl_3$) δ 7.64 (1H, d, *J* = 8.5 Hz), 7.38 (1H, d, *J* = 5.4 Hz), 7.15 (1H, d, *J* = 5.4 Hz), 7.11 (1H, d, *J* = 2.2 Hz), 6.79 (1H, dd, *J* = 8.5, 2.2 Hz), 3.63 (2H, brs); ^{13}C NMR (126 MHz, $CDCl_3$) δ 143.6, 141.0, 130.5, 127.2, 123.2, 123.1, 115.0, 108.4. Data in accordance with the literature.[17]

• Following **GP5**, piperidine and *N*-methylbenzo[*b*]thiophen-5-amine (32.5 mg, 0.20 mmol) gave **31** (22.5 mg, 75%) as a solid.

3-Amino-4-methylphenol (S7)

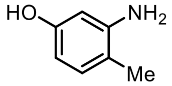

• Following **GP5** but using 2.0 equiv. of the amine, Et_3N and 3-ethylamino-4-methylphenol **43** (30 mg, 0.20 mmol) gave **S7** (12 mg, 48%) as a solid. ^{1}H NMR (400 MHz, MeOH-d_4) δ 6.77 (1H, d, *J* = 8.1 Hz), 6.22 (1H, d, *J* = 2.4 Hz), 6.10 (1H, dd, *J* = 8.1, 2.4 Hz), 4.85 (3H, s), 2.05 (3H, s); ^{13}C NMR (101 MHz, MeOH-d_4) δ 157.2, 147.2, 131.7, 115.4, 106.5, 103.6, 16.6. Data in accordance with the literature.[18]

• Following **GP5** but using 2.0 equiv. of the amine, piperidine and 3-ethylamino-4-methylphenol (30 mg, 0.20 mmol) **43** gave **S7** (18 mg, 73%) as a solid.

6-Methoxyquinolin-8-amine (S8)

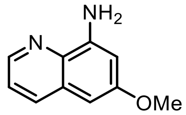

• Following **GP5** but stirring the reaction for 72 h, piperidine and primaquine **45** (52 mg, 0.20 mmol) gave **S8** (7 mg, 20%) as an oil. ^{1}H NMR (500 MHz, $CDCl_3$) δ 8.60 (1H, d, *J* = 3.8 Hz), 7.95 (1H, d, *J* = 8.2 Hz), 7.32 (1H, dd, *J* = 8.3, 4.2 Hz), 6.58 (1H, d, *J* = 2.5 Hz), 6.48 (1H, d, *J* = 2.5 Hz), 4.99 (2H, brs), 3.88 (3H, s); ^{13}C NMR (126 MHz, $CDCl_3$) δ 159.0, 145.1, 135.5, 135.0, 134.9, 130.0, 122.0, 101.8, 94.7, 55.4. Data in accordance with the literature.[19]

***N*-Benzylaniline (33)**

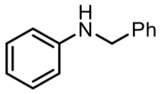

• Following **GP5**, Et_3N and *N*-benzyl-*N*-methylaniline **46** (39.5 mg, 0.20 mmol) gave **33** (22.5 mg, 61%) as a solid. ^{1}H NMR (500 MHz, $CDCl_3$) δ 7.43–7.33 (4H, m), 7.33–7.27 (1H, m), 7.24–7.17 (2H, m), 6.78–6.71 (1H, m), 6.70–6.64 (2H, m), 4.35 (2H, s), 4.14 (1H, brs); ^{13}C NMR (126 MHz, $CDCl_3$) δ 148.2, 139.5, 129.4, 128.8, 127.7, 127.4, 117.7, 113.0, 48.5. Data in accordance with the literature.[20]

• Following **GP5**, piperidine and *N*-benzyl-*N*-methylaniline **46** (39.5 mg, 0.20 mmol) gave **33** (15.5 mg, 42%) as a solid.

***N*-Methylaniline (2)**

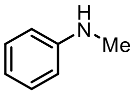

An oven-dried flask was charged with *N*-benzyl-*N*-methylaniline **46** (39.5 mg, 0.20 mmol) and 10% Pd/C (11 mg, 5 mol%). The flask was fitted with a rubber septum, then evacuated under high vacuum and backfilled with N_2 (x 3). Then MeOH (0.1 M) was added. The resulting mixture was stirred under hydrogen at room temperature for 1.5 h, then the solvent was evaporated under reduced pressure. The crude was purified by column chromatography on silica gel eluting with petrol–EtOAc (15:1) to give **2** as an oil (21.5 mg, quant.). ^{1}H NMR (500 MHz, $CDCl_3$) δ 7.25–7.17 (2H, m), 6.73 (1H, t, J = 7.2 Hz), 6.67–6.61 (2H, m), 3.72 (1H, brs), 2.85 (3H, s); ^{13}C NMR (126 MHz, $CDCl_3$) δ 149.4, 129.3, 117.4, 112.6, 30.9. Data in accordance with the literature.[20]

5.2 [3+2]-Type Cycloadditions

General Procedure for the [3+2]-Type Cycloadditions – GP6

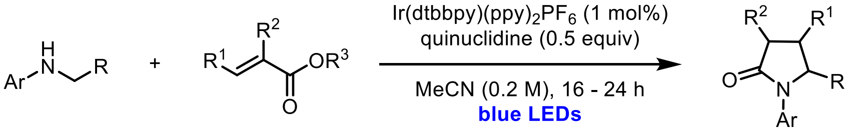

A dry tube equipped with a stirring bar was charged with the Ir(dtbbpy)(ppy)$_2$PF$_6$ (2 mg, 2 µmol, 1 mol%), quinuclidine (11 mg, 0.10 mmol, 0.5 equiv.), the acrylate (if solid) (0.20 mmol, 1.0 equiv.) and the *N*-alkyl aniline (if solid) (0.30 mmol, 1.5 equiv.). The tube was capped with a Supelco aluminium crimp seal with septum (PTFE/butyl), then evacuated under high vacuum and backfilled with N_2 (x 3). Degassed CH_3CN (0.2 M), the acrylate (if liquid) (0.20 mmol, 1.0 equiv.) and the *N*-alkyl aniline (if liquid) (0.30 mmol, 1.5 equiv.) were sequentially added. The vial was purged with a stream of N_2 and the lid sealed with parafilm and placed approximately 4 cm from blue LEDs. The blue LEDs were switched on and the mixture was stirred under irradiation without fan for 16-24 hours. The tube was opened, and the solvent was evaporated under reduced. The crude was purified by flash column chromatography on silica gel with Hexane–EtOAc.

1-Phenylpyrrolidin-2-one (47)

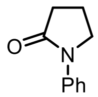

• Following **GP6**, *N*-methylaniline (32.5 µL, 0.3 mmol) and methyl acrylate (18 µL, 0.2 mmol) gave **47** (23 mg, 71%) as a solid. ^{1}H NMR (500 MHz, $CDCl_3$) δ 7.64–7.57 (2H, m), 7.39–7.34 (2H, m), 7.14 (1H, t, *J* = 7.4 Hz), 3.86 (2H, t, *J* = 7.0 Hz), 2.61 (2H, t, *J* = 8.1 Hz), 2.16 (2H, p, *J* = 7.6 Hz); ^{13}C NMR (126 MHz, $CDCl_3$) δ 174.3, 139.5, 128.9, 124.6, 120.1, 48.9, 32.9, 18.2. Data in accordance with the literature.[21]

• Following **GP6**, *N*-methylaniline (32.5 µL, 0.3 mmol) and benzyl acrylate (31 µL, 0.2 mmol) gave **47** (28.5 mg, 89%) as a solid.

3-Methyl-1-phenylpyrrolidin-2-one (48)

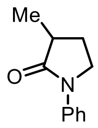

Following **GP6**, *N*-methylaniline (32.5 µL, 0.3 mmol) and methyl methacrylate (21 µL, 0.2 mmol) gave **48** (34 mg, 97%) as a solid. ^{1}H NMR (500 MHz, $CDCl_3$) δ 7.67–7.60 (2H, m),

7.39–7.32 (2H, m), 7.13 (1H, tt, J = 7.4, 1.2 Hz), 3.82–3.71 (2H, m), 2.72–2.60 (1H, m), 2.43–2.29 (1H, m), 1.83–1.70 (1H, m), 1.30 (3H, d, J = 7.1 Hz); ^{13}C NMR (126 MHz, $CDCl_3$) δ 176.7, 139.8, 128.9, 124.4, 119.7, 46.6, 38.4, 27.1, 16.3. Data in accordance with the literature.[22]

4-Methyl-1-phenylpyrrolidin-2-one (49)

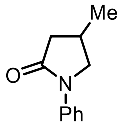

Following **GP6**, *N*-methylaniline (32.5 µL, 0.3 mmol) and methyl crotonate (21 µL, 0.2 mmol) gave **49** (29 mg, 83%) as an oil. ^{1}H NMR (500 MHz, $CDCl_3$) δ 7.56–7.49 (2H, m), 7.32–7.25 (2H, m), 7.09–7.03 (1H, m), 3.86 (1H, dd, J = 9.4, 7.5 Hz), 3.37 (1H, dd, J = 9.4, 6.4 Hz), 2.67 (1H, dd, J = 16.8, 8.4 Hz), 2.54–2.40 (1H, m), 2.18 (1H, dd, J = 16.8, 7.4 Hz), 1.13 (3H, d, J = 6.8 Hz); ^{13}C NMR (126 MHz, $CDCl_3$) δ 173.9, 139.5, 128.9, 124.5, 120.0, 56.0, 41.1, 26.4, 19.6. Data in accordance with the literature.[23]

4-Isopropyl-1-phenylpyrrolidin-2-one (50)

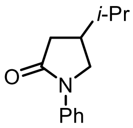

Following **GP6**, *N*-methylaniline (32.5 µL, 0.3 mmol) and methyl 4-methyl-2-pentenoate (28 µL, 0.2 mmol) gave **50** (32 mg, 79%) as an oil. ^{1}H NMR (400 MHz, $CDCl_3$) δ 7.64–7.56 (2H, m), 7.41–7.31 (2H, m), 7.18–7.09 (1H, m), 3.87 (1H, dd, J = 9.5, 8.0 Hz), 3.56 (1H, dd, J = 9.5, 8.2 Hz), 2.68 (1H, dd, J = 16.9, 8.6 Hz), 2.35 (1H, dd, J = 16.9, 9.7 Hz), 2.23–2.10 (1H, m), 1.75–1.59 (1H, m), 0.98 (6H, d, J = 6.7 Hz); ^{13}C NMR (126 MHz, $CDCl_3$) δ 174.0, 139.5, 128.9, 124.6, 120.1, 53.2, 38.9, 37.9, 32.6, 20.6, 20.2; HRMS (ESI) Found MNa^+ 226.1199, $C_{13}H_{17}NONa$ requires 226.1202.

4,4-Dimethyl-1-phenylpyrrolidin-2-one (51)

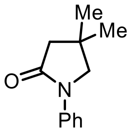

Following **GP6**, *N*-methylaniline (32.5 µL, 0.3 mmol) and methyl 3-methylbut-2-enoate (24.5 µL, 0.2 mmol) gave **51** (13 mg, 34%) as an oil. ^{1}H NMR (400 MHz, $CDCl_3$) δ 7.64–

7.54 (2H, m), 7.40–7.32 (2H, m), 7.19–7.10 (1H, m), 3.57 (2H, s), 2.44 (2H, s), 1.25 (6H, s); ^{13}C NMR (126 MHz, $CDCl_3$) δ 173.7, 139.7, 129.0, 124.6, 120.1, 61.8, 47.8, 32.6, 27.7; HRMS (ASAP) Found MH^+ 190.1229, $C_{12}H_{16}NO$ requires 190.1226.

3-(Hydroxymethyl)-1-phenylpyrrolidin-2-one (52)

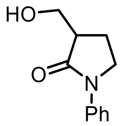

Following **GP6**, *N*-methylaniline (32.5 µL, 0.3 mmol) and methyl 2-(hydroxymethyl)acrylate (20.5 µL, 0.2 mmol) gave **52** (29 mg, 75%) as an oil. ^{1}H NMR (500 MHz, $CDCl_3$) δ 7.65–7.56 (2H, m), 7.42–7.31 (2H, m), 7.19–7.12 (1H, m), 3.96 (1H, dd, *J* = 11.0, 4.6 Hz), 3.89–3.79 (3H, m), 3.17 (1H, brs), 2.92–2.83 (1H, m), 2.31–2.22 (1H, m), 2.04–1.93 (1H, m); ^{13}C NMR (126 MHz, $CDCl_3$) δ 175.7, 139.1, 129.0, 125.0, 120.1, 63.2, 47.4, 45.0, 21.2; HRMS (ESI) Found MNa^+ 214.0836, $C_{11}H_{13}NO_2Na$ requires 214.0838.

4-(Hydroxymethyl)-1-phenylpyrrolidin-2-one (53)

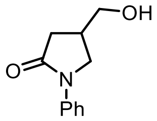

Following **GP6**, *N*-methylaniline (32.5 µL, 0.3 mmol) and 2(5*H*)-furanone (14 µL, 0.2 mmol) gave **53** (31 mg, 81%) as an oil. ^{1}H NMR (500 MHz, $CDCl_3$) δ 7.23–7.16 (2H, m), 6.76 (1H, tt, *J* = 7.3, 1.1 Hz), 6.65–6.59 (2H, m), 4.44 (1H, dd, *J* = 9.3, 7.2 Hz), 4.17 (1H, dd, *J* = 9.3, 5.2 Hz), 3.81 (1H, brs), 3.24 (2H, d, *J* = 7.3 Hz), 2.95–2.83 (1H, m), 2.69 (1H, dd, *J* = 17.7, 8.6 Hz), 2.38 (1H, dd, *J* = 17.7, 5.9 Hz); ^{13}C NMR (126 MHz, $CDCl_3$) δ 176.7, 147.5, 129.6, 118.4, 113.0, 71.4, 46.4, 35.4, 32.5; HRMS (ESI) Found MNa^+ 214.0837, $C_{11}H_{13}NO_2Na$ requires 214.0838.

3-Fluoro-1-phenylpyrrolidin-2-one (54)

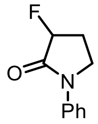

Following **GP6**, *N*-methylaniline (32.5 µL, 0.3 mmol) and methyl 2-acetamidoacrylate (19 µL, 0.2 mmol) gave **54** (33 mg, 92%) as a solid. ^{1}H NMR (500 MHz, $CDCl_3$) δ 7.65 (2H, d, *J* = 8.0 Hz), 7.40 (2H, t, *J* = 7.8 Hz), 7.21 (1H, t, *J* = 7.4 Hz), 5.21 (1H, dt, *J* = 52.6, 7.3 Hz), 3.93 (1H, td, *J* = 9.3, 3.5 Hz), 3.85–3.76 (1H, m), 2.70–2.55 (1H, m), 2.44–2.28 (1H, m); ^{13}C

NMR (126 MHz, $CDCl_3$) δ 168.5 (d, J = 20.6 Hz), 138.6, 129.2, 125.6, 120.0, 89.4 (d, J = 184.8 Hz), 44.6 (d, J = 4.0 Hz), 26.2 (d, J = 20.4 Hz); ^{19}F NMR (376 MHz, $CDCl_3$) δ −187.40; HRMS (ESI) Found MNa^+ 202.0635, $C_{10}H_{10}FNONa$ requires 202.0639.

_N_-(2-Oxo-1-Phenylpyrrolidin-3-yl)acetamide (55)

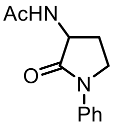

Following **GP6** but stirring the reaction for 48 h, *N*-methylaniline (32.5 µL, 0.3 mmol) and methyl 2-acetamidoacrylate (28.5 mg, 0.2 mmol) gave **55** (28 mg, 64%) as an oil. ^{1}H NMR (400 MHz, $CDCl_3$) δ 7.62–7.54 (2H, m), 7.43–7.32 (2H, m), 7.21–7.14 (1H, m), 6.55 (1H, s), 4.57 (1H, ddd, J = 11.1, 8.2, 5.5 Hz), 3.85–3.72 (2H, m), 2.84 (1H, dddd, J = 12.5, 7.9, 5.9, 1.5 Hz), 2.06 (3H, s), 1.97–1.90 (1H, m); ^{13}C NMR (101 MHz, $CDCl_3$) δ 171.8, 171.0, 139.0, 129.1, 125.3, 119.9, 53.0, 45.6, 27.8, 23.2; HRMS (ESI) Found MNa^+ 241.0941, $C_{12}H_{14}N_2O_2Na$ requires 241.0947.

Methyl 4-Methyl-2-oxo-1-phenylpyrrolidine-3-carboxylate (S9)

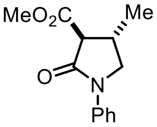

Following **GP6**, *N*-methylaniline (32.5 µL, 0.3 mmol) and dimethyl 2-ethylidenemalonate (28.5 µL, 0.2 mmol) gave **S9** (43.5 mg, 93%) as an inseparable mixture of *trans* and *cis* isomers as a solid. *trans*:*cis* 5.9:1.

Data for the *trans* isomer: ^{1}H NMR (400 MHz, $CDCl_3$) δ 7.59 (2H, d, J = 7.8 Hz), 7.36 (2H, t, J = 7.9 Hz), 7.16 (1H, t, J = 7.4 Hz), 4.01 (1H, dd, J = 9.4, 7.8 Hz), 3.82 (3H, s), 3.45 (1H, dd, J = 9.4, 7.3 Hz), 3.29 (1H, d, J = 8.5 Hz), 2.97–2.78 (1H, m), 1.26 (3H, d, J = 6.8 Hz); ^{13}C NMR (101 MHz, $CDCl_3$) δ 169.9, 168.6, 139.0, 129.0, 125.1, 120.1, 57.8, 54.2, 52.8, 31.2, 18.3.

Data for the *cis* isomer: ^{1}H NMR (400 MHz, $CDCl_3$) δ 7.59 (2H, d, J = 7.8 Hz), 7.36 (2H, t, J = 7.9 Hz), 7.16 (1H, t, J = 7.4 Hz), 3.87 (1H, dd, J = 9.2, 7.8 Hz), 3.75 (3H, s), 3.74–3.69 (1H, m), 3.61 (1H, d, J = 9.1 Hz), 2.89–2.78 (1H, m), 1.17 (3H, d, J = 6.9 Hz); ^{13}C NMR (101 MHz, $CDCl_3$) δ 169.5, 169.2, 139.0, 129.0, 125.1, 120.4, 55.3, 54.8, 52.3, 30.3, 14.2. HRMS (ESI) Found MNa^+ 256.0937, $C_{13}H_{15}NO_3Na$ requires 256.0944.

Ethyl 4-Methyl-2-oxo-1-phenylpyrrolidine-3-carboxylate (56)

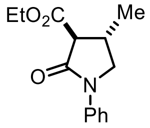

Following **GP6**, *N*-methylaniline (32.5 µL, 0.3 mmol) and diethyl 2-ethylidenemalonate (36.5 µL, 0.2 mmol) gave **56** (45 mg, 91%) as an inseparable mixture of *trans* and *cis* isomers as a solid. *trans*:*cis* 7.5:1.

Data for the *trans* isomer: ^{1}H NMR (400 MHz, $CDCl_3$) δ 7.64–7.49 (2H, m), 7.42–7.28 (2H, m), 7.20–7.06 (1H, m), 4.26 (2H, qd, *J* = 7.1, 2.6 Hz), 3.99 (1H, dd, *J* = 9.4, 7.7 Hz), 3.42 (1H, dd, *J* = 9.4, 7.4 Hz), 3.25 (1H, d, *J* = 8.5 Hz), 2.88 (1H, dq, *J* = 14.4, 7.2 Hz), 1.31 (3H, t, *J* = 7.2 Hz), 1.24 (3H, d, *J* = 6.8 Hz); ^{13}C NMR (101 MHz, $CDCl_3$) δ 169.4, 168.7, 139.0, 128.9, 124.9, 120.0, 61.7, 57.9, 54.1, 31.1, 18.2, 14.3.

Data for the *cis* isomer: ^{1}H NMR (400 MHz, $CDCl_3$) 7.64–7.49 (2H, m), 7.42–7.28 (2H, m), 7.20–7.06 (1H, m), 4.30–4.15 (2H, m), 3.85 (1H, dd, *J* = 9.2, 7.7 Hz), 3.70 (1H, t, *J* = 9.2 Hz), 3.56 (1H, d, *J* = 8.9 Hz), 2.97–2.75 (1H, m), 1.28–1.27 (3H, m), 1.17 (3H, d, *J* = 6.9 Hz); ^{13}C NMR (101 MHz, $CDCl_3$) δ 169.6, 168.7, 139.1, 128.9, 125.0, 120.4, 61.3, 55.3, 54.8, 30.3, 17.4, 14.0. HRMS (ESI) Found MH^+ 248.1275, $C_{14}H_{18}NO_3$ requires 248.1281. Data in accordance with the literature.[24]

Ethyl 4-Isopropyl-2-oxo-1-phenylpyrrolidine-3-carboxylate (57)

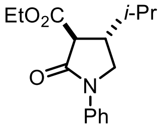

Following **GP6**, *N*-methylaniline (32.5 µL, 0.3 mmol) and diethyl isobutylidenemalonate (43 mg, 0.2 mmol) gave **57** (39 mg, 71%) as an inseparable mixture of *trans* and *cis* isomers as a solid. *trans*:*cis* 9.6:1.

Data for the *trans* isomer: ^{1}H NMR (500 MHz, $CDCl_3$) δ 7.63–7.56 (2H, m), 7.40–7.31 (2H, m), 7.20–7.11 (1H, m), 4.28 (2H, q, *J* = 7.1 Hz), 3.93 (1H, dd, *J* = 9.5, 8.4 Hz), 3.53 (1H, dd, *J* = 9.5, 8.3 Hz), 3.40 (1H, d, *J* = 9.4 Hz), 2.67 (1H, p, *J* = 8.5 Hz), 1.83–1.68 (1H, m), 1.32 (3H, t, *J* = 7.1 Hz), 0.99 (3H, d, *J* = 6.7 Hz), 0.96 (3H, d, *J* = 6.7 Hz); ^{13}C NMR (126 MHz, $CDCl_3$) δ 170.4, 169.1, 138.9, 128.9, 125.0, 120.2, 61.8, 55.2, 51.4, 42.6, 32.2, 20.3, 20.0, 14.3; HRMS (ESI) Found MNa^+ 298.1407, $C_{16}H_{21}NO_3Na$ requires 298.1414.

Ethyl 4,4-Dimethyl-2-oxo-1-phenylpyrrolidine-3-carboxylate (58)

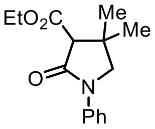

Following **GP6**, *N*-methylaniline (32.5 µL, 0.3 mmol) and diethyl 2-(1-methylethylidene)malonate (39 µL, 0.2 mmol) gave **58** (40 mg, 77%) as an oil. ^{1}H NMR (400 MHz, $CDCl_3$) δ 7.64–7.53 (2H, m), 7.42–7.32 (2H, m), 7.20–7.12 (1H, m), 4.22 (2H, q, *J* = 7.2 Hz), 3.82 (1H, d, *J* = 9.2 Hz), 3.49 (1H, d, *J* = 9.2 Hz), 3.25 (1H, s), 1.33 (3H, s), 1.29 (3H, t, *J* = 7.2 Hz), 1.21 (3H, s); ^{13}C NMR (101 MHz, $CDCl_3$) δ 169.4, 168.7, 139.2, 129.0, 125.1, 120.5, 61.9, 61.4, 60.7, 36.4, 28.8, 22.9, 14.4. Data in accordance with the literature.[25]

3,4-Dimethyl-1-phenylpyrrolidin-2-one (59)

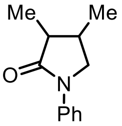

Following **GP6**, *N*-methylaniline (32.5 µL, 0.3 mmol) and ethyl tiglate (28 µL, 0.2 mmol) gave **59** (14 mg, 37%) as an inseparable mixture of *trans* and *cis* isomers as a solid. *trans*:*cis* = 1.6:1. ^{1}H NMR (400 MHz, $CDCl_3$, diastereomers) δ 7.63 (2H, d, *J* = 8.0 Hz), 7.36 (2H, t, *J* = 8.1 Hz), 7.12 (1H, t, *J* = 7.4 Hz), 3.90 (0.7H, dd, *J* = 9.5, 6.7 Hz), 3.83 (0.4H, dd, *J* = 9.4, 7.6 Hz), 3.46–3.31 (1H, m), 2.73 (0.6H, p, *J* = 7.5 Hz), 2.59 (0.7H, hd, *J* = 7.0, 2.4 Hz), 2.21 (0.4H, dq, *J* = 10.1, 6.9 Hz), 2.15–2.00 (0.4H, m), 1.28 (1.3H, d, *J* = 6.9 Hz), 1.22 (1.3H, d, *J* = 6.5 Hz), 1.18 (2.1H, d, *J* = 7.3 Hz), 1.07 (2.1H, d, *J* = 7.0 Hz); ^{13}C NMR (101 MHz, $CDCl_3$, diastereomers) δ 176.6, 176.4, 139.9, 139.8, 128.9, 124.3, 119.7 (2 x C), 54.2, 54.0, 46.1, 42.4, 35.6, 30.4, 17.3, 14.4, 14.3, 10.7; HRMS (ESI) Found MNa^+ 212.1042, $C_{12}H_{15}NONa$ requires 212.1046.

5-Methyl-1-phenylpyrrolidin-2-one (60)

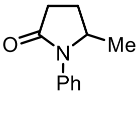

Following **GP6**, *N*-ethylaniline (38 µL, 0.3 mmol) and methyl acrylate (18 µL, 0.2 mmol) gave **60** (27 mg, 77%) as a solid. ^{1}H NMR (400 MHz, $CDCl_3$) δ 7.42–7.32 (4H, m), 7.24–7.16 (1H, m), 4.30 (1H, dp, *J* = 7.4, 6.3 Hz), 2.64 (1H, ddd, *J* = 17.0, 9.5, 6.1 Hz), 2.54 (1H, ddd, *J* = 17.0, 9.5, 7.3 Hz), 2.37 (1H, dddd, *J* = 12.7, 9.5, 7.4, 6.1 Hz), 1.80–1.70 (1H, m),

1.20 (3H, d, J = 6.3 Hz); ^{13}C NMR (101 MHz, $CDCl_3$) δ 174.3, 137.7, 129.1, 125.9, 124.2, 55.7, 31.5, 26.9, 20.3. Data in accordance with the literature.[26]

3,5-Dimethyl-1-phenylpyrrolidin-2-one (61)

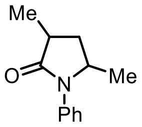

Following **GP6**, *N*-ethylaniline (38 µL, 0.3 mmol) and methyl methacrylate (21 µL, 0.2 mmol) gave **61** (38 mg, quant.) as a separable mixture of *trans* and *cis* isomers. *trans*:*cis* = 1.3:1. The relative configuration was assigned by NOESY experiment.

Data for the *trans* isomer: ^{1}H NMR (500 MHz, $CDCl_3$) δ 7.51–7.44 (2H, m), 7.40–7.33 (2H, m), 7.20–7.13 (1H, m), 4.37–4.21 (1H, m), 2.77 (1H, tq, J = 8.7, 7.2 Hz), 2.09–1.89 (2H, m), 1.27 (3H, d, J = 7.2 Hz), 1.22 (3H, d, J = 6.4 Hz); ^{13}C NMR (126 MHz, $CDCl_3$) δ 176.6, 138.1, 129.1, 125.3, 123.0, 53.5, 36.5, 35.2, 19.6, 16.3; HRMS (ASAP) Found MH^+ 190.1226, $C_{12}H_{16}NO$ requires 190.1226.

Data for the *cis* isomer: ^{1}H NMR (500 MHz, $CDCl_3$) δ 7.42–7.34 (2H, m), 7.32–7.27 (2H, m), 7.24–7.17 (1H, m), 4.16 (1H, dp, J = 8.5, 6.1 Hz), 2.68–2.49 (2H, m), 1.40–1.33 (1H, m), 1.32 (3H, d, J = 6.7 Hz), 1.18 (3H, d, J = 6.1 Hz); ^{13}C NMR (126 MHz, $CDCl_3$) δ 176.8, 137.7, 128.9, 125.9, 124.7, 53.2, 37.4, 36.8, 20.9, 16.7; HRMS (ASAP) Found MH^+ 190.1226, $C_{12}H_{16}NO$ requires 190.1226.

Methyl 4,5-Dimethyl-2-oxo-1-phenylpyrrolidine-3-carboxylate (62)

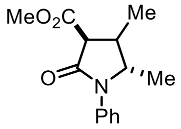

Following **GP6**, *N*-ethylaniline (38 µL, 0.3 mmol) and dimethyl 2-ethylidenemalonate (28.5 µL, 0.2 mmol) gave **62** (28.5 mg, 58%) as a separable mixture of *trans*-*trans* and *trans*-*cis* isomers. *trans*-*trans*:*trans*-*cis* = 1.1:1. The relative configuration was assigned by NOESY experiment.

Data for the *trans*-*trans* isomer: ^{1}H NMR (500 MHz, $CDCl_3$) δ 7.51–7.42 (2H, m), 7.41–7.33 (2H, m), 7.22–7.16 (1H, m), 4.35 (1H, p, J = 6.8 Hz), 3.81 (3H, s), 3.36 (1H, d, J = 10.6 Hz), 3.04 (1H, dp, J = 10.7, 7.0 Hz), 1.15 (3H, d, J = 7.0 Hz), 1.12 (3H, d, J = 6.6 Hz); ^{13}C NMR (126 MHz, $CDCl_3$) δ 170.0, 168.5, 137.7, 129.2, 125.9, 123.2, 57.8, 55.7, 52.8, 35.3, 14.1, 14.0; HRMS (ASAP) Found MH^+ 248.1277, $C_{14}H_{18}NO_3$ requires 248.1281.

Data for the *trans-cis* isomer: ^{1}H NMR (500 MHz, $CDCl_3$) δ 7.41–7.36 (2H, m), 7.30–7.26 (2H, m), 7.25–7.20 (1H, m), 3.83 (3H, s), 3.79–3.74 (1H, m), 3.26 (1H, d, *J* = 10.0 Hz), 2.51–2.40 (1H, m), 1.26 (3H, d, *J* = 6.7 Hz), 1.23 (3H, d, *J* = 6.1 Hz); ^{13}C NMR (126 MHz, $CDCl_3$) δ 170.4, 168.6, 137.0, 129.1, 126.5, 124.8, 60.7, 56.7, 52.8, 39.8, 18.6, 17.1; HRMS (ASAP) Found MH^+ 248.1277, $C_{14}H_{18}NO_3$ requires 248.1281.

3-Methyl-1,5-diphenylpyrrolidin-2-one (63)

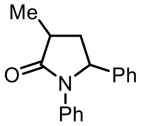

Following **GP6**, *N*-benzylaniline (37 mg, 0.20 mmol) and methyl methacrylate (21 µL, 0.2 mmol) gave **63** (35 mg, 70%) as separable mixture of *trans* and *cis* isomers. *trans*:*cis* = 2.2:1.
Data for the *trans* isomer: ^{1}H NMR (500 MHz, $CDCl_3$) δ 7.55 (2H, d, *J* = 8.2 Hz), 7.36–7.30 (2H, m), 7.29–7.20 (5H, m), 7.10–7.03 (1H, m), 5.21 (1H, dd, *J* = 7.5, 3.0 Hz), 2.90–2.78 (1H, m), 2.33–2.20 (2H, m), 1.31 (3H, d, *J* = 7.0 Hz); ^{13}C NMR (126 MHz, $CDCl_3$) δ 177.4, 141.3, 138.9, 129.1, 128.8, 127.7, 125.8, 124.6, 121.2, 61.6, 38.0, 35.9, 16.0. Data in accordance with the literature.[27]
Data for the *cis* isomer: ^{1}H NMR (500 MHz, $CDCl_3$) δ 7.34–7.30 (2H, m), 7.27–7.15 (7H, m), 7.06–7.00 (1H, m), 5.16 (1H, dd, *J* = 8.8, 6.7 Hz), 2.86–2.78 (1H, m), 2.77–2.67 (1H, m), 1.65 (1H, ddd, *J* = 12.0, 9.9, 8.6 Hz), 1.37 (3H, d, *J* = 6.8 Hz); ^{13}C NMR (101 MHz, $CDCl_3$) δ 177.4, 141.4, 138.1, 128.9, 128.6, 127.8, 126.6, 125.0, 123.1, 62.0, 39.1, 37.8, 16.7. Data in accordance with the literature.[27]

5-Benzyl-3-methyl-1-phenylpyrrolidin-2-one (64)

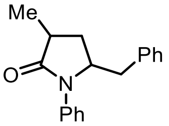

Following **GP6**, *N*-phenethylaniline **S1** (59 mg, 0.3 mmol) and methyl methacrylate (21 µL, 0.2 mmol) gave **64** (51 mg, 96%) as separable mixture of *trans* and *cis* isomers. *trans*:*cis* = 1.6:1. The relative configuration was assigned by NOESY experiment.
Data for the *trans* isomer: ^{1}H NMR (400 MHz, $CDCl_3$) δ 7.62–7.54 (2H, m), 7.45–7.35 (2H, m), 7.29–7.23 (2H, m), 7.22–7.15 (2H, m), 7.13–7.06 (2H, m), 4.42 (1H, tdd, *J* = 8.6, 3.5, 2.2 Hz), 2.92 (1H, dd, *J* = 13.6, 3.5 Hz), 2.64 (1H, dd, *J* = 13.6, 8.7 Hz), 2.39 (1H, ddq, *J* = 9.9, 8.5, 7.1 Hz), 2.15 (1H, ddd, *J* = 12.9, 8.6, 2.2 Hz), 1.74 (1H, ddd, *J* = 12.9, 9.9, 8.3 Hz), 1.14

(3H, d, *J* = 7.1 Hz); ^{13}C NMR (101 MHz, $CDCl_3$) δ 176.7, 138.0, 137.2, 129.4, 129.2, 128.7, 126.9, 125.4, 122.8, 58.5, 38.5, 36.2, 31.7, 16.3; HRMS (ASAP) Found MH$^+$ 266.1545, $C_{18}H_{20}NO$ requires 266.1539.

Data for the *cis* isomer: ^{1}H NMR (500 MHz, $CDCl_3$) δ 7.47–7.38 (4H, m), 7.31–7.21 (4H, m), 7.13–7.06 (2H, m), 4.33 (1H, tdd, *J* = 9.6, 7.0, 3.7 Hz), 3.14 (1H, dd, *J* = 13.4, 3.7 Hz), 2.55 (1H, ddt, *J* = 16.7, 9.6, 7.1 Hz), 2.44 (1H, dd, *J* = 13.4, 9.6 Hz), 2.32 (1H, ddd, *J* = 12.8, 9.0, 6.7 Hz), 1.46 (1H, ddd, *J* = 12.8, 10.2, 8.3 Hz), 1.24 (3H, d, *J* = 7.0 Hz); ^{13}C NMR (126 MHz, $CDCl_3$) δ 177.0, 137.7, 137.0, 129.3, 129.2, 128.7, 126.8, 126.1, 124.7, 58.6, 40.6, 37.1, 33.7, 16.8; HRMS (ASAP) Found MH$^+$ 266.1546, $C_{18}H_{20}NO$ requires 266.1539.

2-(4-Methyl-5-oxo-1-phenylpyrrolidin-2-yl)acetonitrile (65)

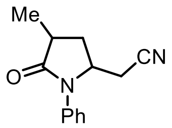

Following **GP6**, 3-anilinopropionitrile (44 mg, 0.3 mmol) and methyl methacrylate (21 µL, 0.2 mmol) gave **65** (42 mg, 97%) as separable mixture of *trans* and *cis* isomers. *trans*:*cis* = 1.4:1. The relative configuration was assigned by NOESY experiment.

Data for the *trans* isomer: ^{1}H NMR (500 MHz, $CDCl_3$) δ 7.45–7.35 (4H, m), 7.27–7.22 (1H, m), 4.43 (1H, tt, *J* = 6.9, 3.1 Hz), 2.92 (1H, br h, *J* = 7.2 Hz), 2.60 (1H, dd, *J* = 16.9, 3.5 Hz), 2.52 (1H, dd, *J* = 16.9, 7.0 Hz), 2.36 (1H, ddd, *J* = 13.2, 8.9, 2.8 Hz), 2.14 (1H, dt, *J* = 13.2, 8.7 Hz), 1.30 (3H, d, *J* = 7.2 Hz); ^{13}C NMR (126 MHz, $CDCl_3$) δ 176.2, 136.5, 129.6, 126.7, 124.0, 116.7, 54.2, 35.9, 32.3, 22.2, 16.4; HRMS (ASAP) Found MH$^+$ 215.1178, $C_{13}H_{15}N_2O$ requires 215.1179.

Data for the *cis* isomer: ^{1}H NMR (500 MHz, $CDCl_3$) δ 7.44–7.35 (2H, m), 7.29–7.22 (3H, m), 4.31 (1H, br h, *J* = 7.3 Hz), 2.75–2.66 (2H, m), 2.63 (1H, dd, *J* = 17.0, 3.1 Hz), 2.50 (1H, dd, *J* = 17.0, 6.6 Hz), 1.73–1.60 (1H, m), 1.36 (3H, d, *J* = 6.5 Hz); ^{13}C NMR (126 MHz, $CDCl_3$) δ 176.4, 136.2, 129.6, 127.2, 125.1, 116.0, 53.5, 36.7, 33.2, 23.3, 16.6; HRMS (ASAP) Found MH$^+$ 215.1178, $C_{13}H_{15}N_2O$ requires 215.1179.

5-(Hydroxymethyl)-3-methyl-1-phenylpyrrolidin-2-one (66)

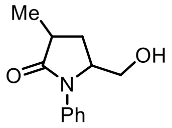

Following **GP6**, *N*-(2-hydroxyethyl)aniline (37.5 µL, 0.3 mmol) and methyl methacrylate (21 µL, 0.2 mmol) gave **66** (35 mg, 86%) as an inseparable mixture of *trans* and *cis* isomers an oil. *trans*:*cis* = 1.6:1. ^{1}H NMR (500 MHz, $CDCl_3$, diastereomers) δ 7.47–7.41 (2H, m), 7.39–7.32 (3.1H, m), 7.31–7.28 (1H, m), 7.21 (0.6H, t, *J* = 7.3 Hz), 7.17 (1H, t, *J* = 7.3 Hz), 4.23–4.13 (1.5H, m), 3.66–3.48 (3.2H, m), 2.82 (1H, ddt, *J* = 16.5, 9.4, 7.2 Hz), 2.63 (0.6H, ddt, *J* = 16.7, 9.7, 7.1 Hz), 2.50 (1H, s), 2.43 (0.6H, ddd, *J* = 12.8, 9.3, 7.4 Hz), 2.37 (1H, ddd, *J* = 13.0, 8.9, 2.3 Hz), 1.94 (0.5H, s), 1.88 (1H, dt, *J* = 12.9, 9.1 Hz), 1.75 (0.6H, ddd, *J* = 12.9, 9.6, 8.0 Hz), 1.31 (1.8H, d, *J* = 7.1 Hz), 1.23 (3H, d, *J* = 7.2 Hz); ^{13}C NMR (126 MHz, $CDCl_3$, diastereomers) δ 177.8, 177.7, 137.8, 137.3, 129.2 (2 x C), 126.4, 125.9, 124.7, 123.6, 62.4, 61.9, 59.3, 58.8, 36.9 (2 x C), 30.5, 29.6, 16.8 (2 x C). Data in accordance with the literature.[28]

5-(((*tert*-Butyldimethylsilyl)oxy)methyl)-3-methyl-1-phenylpyrrolidin-2-one (67)

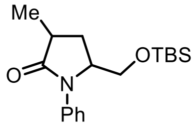

Following **GP6**, *N*-(2-((*tert*-butyldimethylsilyl)oxy)ethyl)aniline **S2** (75.5 mg, 0.3 mmol) and methyl methacrylate (21 µL, 0.2 mmol) gave **67** (61 mg, 96%) as separable mixture of *trans* and *cis* isomers. *trans*:*cis* = 1.7:1. The relative configuration was assigned by NOESY experiment.

Data for the *trans* isomer: ^{1}H NMR (500 MHz, $CDCl_3$) δ 7.46 (2H, d, *J* = 8.1 Hz), 7.36 (2H, t, *J* = 7.7 Hz), 7.17 (1H, t, *J* = 7.4 Hz), 4.20 (1H, ddt, *J* = 8.8, 4.2, 2.1 Hz), 3.62 (1H, dd, *J* = 10.5, 4.2 Hz), 3.57 (1H, dd, *J* = 10.5, 2.7 Hz), 2.83 (1H, br h, *J* = 7.2 Hz), 2.34 (1H, ddd, *J* = 12.6, 8.9, 1.8 Hz), 1.89 (1H, dt, *J* = 12.6, 9.2 Hz), 1.26 (3H, d, *J* = 7.2 Hz), 0.84 (9H, s), −0.07 (6H, d, *J* = 16.4 Hz); ^{13}C NMR (126 MHz, $CDCl_3$) δ 177.4, 138.1, 129.0, 125.5, 123.5, 62.9, 59.2, 37.0, 30.9, 25.9, 18.2, 17.0, −5.5; HRMS (ASAP) Found MH^+ 320.2033, $C_{18}H_{30}NO_2Si$ requires 320.2040.

Data for the *cis* isomer: ^{1}H NMR (500 MHz, $CDCl_3$) δ 7.36 (2H, t, *J* = 7.7 Hz), 7.31 (2H, dt, *J* = 8.5, 1.3 Hz), 7.20 (1H, td, *J* = 7.3, 1.3 Hz), 4.18 (1H, tt, *J* = 7.2, 3.3 Hz), 3.60 (1H, dd, *J* = 10.6, 4.1 Hz), 3.52 (1H, dd, *J* = 10.6, 2.3 Hz), 2.64 (1H, br h, *J* = 7.2 Hz), 2.42 (1H, ddd, *J* =

12.6, 8.9, 7.4 Hz), 1.81–1.73 (1H, m), 1.33 (3H, d, *J* = 7.2 Hz), 0.82 (9H, s), −0.11 (3H, s), −0.18 (3H, s); ^{13}C NMR (126 MHz, $CDCl_3$) δ 177.3, 137.6, 128.9, 126.0, 124.9, 62.1, 58.9, 36.9, 29.6, 25.9, 18.3, 17.2, −5.6, −5.7; HRMS (ASAP) Found MH^+ 320.2034, $C_{18}H_{30}NO_2Si$ requires 320.2040.

5-(Cyclohex-1-en-1-ylmethyl)-3-methyl-1-phenylpyrrolidin-2-one (68)

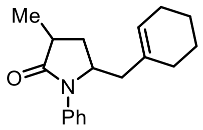

Following **GP6**, *N*-(2-(cyclohex-1-en-1-yl)ethyl)aniline **S3** (60.5 mg, 0.3 mmol) and methyl methacrylate (21 µL, 0.2 mmol) gave **68** (52 mg, 96%) as separable mixture of *trans* and *cis* isomers. *trans*:*cis* = 1.6:1. The relative configuration was assigned by NOESY experiment.

Data for the *trans* isomer: ^{1}H NMR (500 MHz, $CDCl_3$) δ 7.50 (2H, d, *J* = 8.1 Hz), 7.40–7.32 (2H, m), 7.15 (1H, t, *J* = 7.4 Hz), 5.44 (1H, t, *J* = 3.6 Hz), 4.29 (1H, dddd, *J* = 10.0, 8.1, 3.9, 2.1 Hz), 2.80–2.68 (1H, m), 2.31 (1H, dd, *J* = 13.1, 3.5 Hz), 2.18 (1H, ddd, *J* = 12.6, 8.5, 2.1 Hz), 2.01–1.92 (3H, m), 1.92–1.78 (3H, m), 1.64–1.45 (4H, m), 1.25 (3H, d, *J* = 7.0 Hz); ^{13}C NMR (126 MHz, $CDCl_3$) δ 176.6, 138.2, 133.7, 129.0, 125.2, 124.9, 122.9, 56.1, 41.4, 36.3, 32.3, 28.8, 25.4, 22.9, 22.3, 16.3; HRMS (ASAP) Found MH^+ 270.1850, $C_{18}H_{24}NO$ requires 270.1852.

Data for the *cis* isomer: ^{1}H NMR (500 MHz, $CDCl_3$) δ 7.41–7.34 (2H, m), 7.33–7.27 (2H, m), 7.23–7.16 (1H, m), 5.44–5.38 (1H, m), 4.20 (1H, dtd, *J* = 10.5, 7.4, 3.7 Hz), 2.59 (1H, br h, *J* = 7.0 Hz), 2.53–2.42 (2H, m), 2.00–1.93 (2H, m), 1.93–1.82 (2H, m), 1.75 (1H, dd, *J* = 13.6, 10.0 Hz), 1.63–1.55 (2H, m), 1.55–1.48 (2H, m), 1.40 (1H, ddd, *J* = 12.8, 9.9, 8.1 Hz), 1.32 (3H, d, *J* = 7.0 Hz); ^{13}C NMR (126 MHz, $CDCl_3$) δ 177.0, 137.8, 133.1, 128.9, 125.9, 124.7 (2 x C), 56.1, 43.5, 37.2, 34.1, 28.8, 25.4, 22.9, 22.4, 17.0; HRMS (ASAP) Found MH^+ 270.1842, $C_{18}H_{24}NO$ requires 270.1852.

Methyl 2-(4-Methyl-5-oxo-1-phenylpyrrolidin-2-yl)acetate (69)

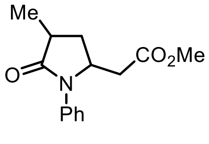

Following **GP6**, methyl 3-anilinopropanoate (54 mg, 0.3 mmol) and methyl methacrylate (21 µL, 0.2 mmol) gave **69** (46 mg, 94%) as separable mixture of *trans* and *cis* isomers. *trans*:*cis* = 1.3:1. The relative configuration was assigned by NOESY experiment.

Data for the *trans* isomer: ^{1}H NMR (500 MHz, $CDCl_3$) δ 7.49–7.43 (2H, m), 7.41–7.34 (2H, m), 7.21–7.16 (1H, m), 4.62–4.55 (1H, m), 3.62 (3H, s), 2.80–2.71 (1H, m), 2.68 (1H, dd, *J* = 15.6, 3.8 Hz), 2.41 (1H, dd, *J* = 15.6, 9.6 Hz), 2.18 (1H, ddd, *J* = 13.0, 8.5, 2.4 Hz), 2.05 (1H, dt, *J* = 13.0, 8.9 Hz), 1.27 (3H, d, *J* = 7.1 Hz); ^{13}C NMR (126 MHz, $CDCl_3$) δ 176.4, 171.2, 137.4, 129.3, 125.9, 123.3, 54.6, 52.0, 37.7, 36.1, 33.1, 16.2; HRMS (ASAP) Found MH^+ 248.1276, $C_{14}H_{18}NO_3$ requires 248.1281.

Data for the *cis* isomer: ^{1}H NMR (500 MHz, $CDCl_3$) δ 7.36–7.29 (2H, m), 7.24–7.20 (2H, m), 7.18–7.14 (1H, m), 4.48–4.38 (1H, m), 3.54 (3H, s), 2.72 (1H, dd, *J* = 15.8, 4.2 Hz), 2.66 (1H, ddd, *J* = 12.2, 9.0, 6.6 Hz), 2.63–2.52 (1H, m), 2.18 (1H, dd, *J* = 15.8, 9.2 Hz), 1.40 (1H, dt, *J* = 12.2, 9.1 Hz), 1.26 (3H, d, *J* = 6.9 Hz); ^{13}C NMR (126 MHz, $CDCl_3$) δ 176.7, 171.1, 137.1, 129.2, 126.5, 124.9, 54.3, 51.9, 39.7, 37.0, 34.5, 16.9; HRMS (ASAP) Found MH^+ 248.1281, $C_{14}H_{18}NO_3$ requires 248.1281.

5-((1*H*-Imidazol-4-yl)methyl)-1-phenylpyrrolidin-2-one (71)

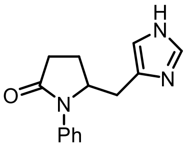

Following **GP6**, *N*-(2-(1*H*-imidazol-4-yl)ethyl)aniline **S4** (56 mg, 0.3 mmol) and methyl acrylate (18 µL, 0.2 mmol) gave **71** (24 mg, 50%) as an oil. ^{1}H NMR (400 MHz, $CDCl_3$) δ 7.54 (1H, s), 7.47 (2H, d, *J* = 7.9 Hz), 7.39 (2H, t, *J* = 7.7 Hz), 7.20 (1H, t, *J* = 7.4 Hz), 6.68 (1H, s), 6.39 (1H, brs), 4.57 (1H, tt, *J* = 7.9, 3.7 Hz), 2.94 (1H, dd, *J* = 14.6, 3.7 Hz), 2.70 (1H, dd, *J* = 14.6, 8.1 Hz), 2.48–2.34 (2H, m), 2.30–2.18 (1H, m), 2.08–1.97 (1H, m); ^{13}C NMR (101 MHz, $CDCl_3$) δ 174.9, 137.6, 134.8, 134.1, 129.3, 126.1, 124.0, 116.1, 59.6, 31.3, 31.1, 23.4; HRMS (ASAP) Found MH^+ 242.1285, $C_{14}H_{16}N_3O$ requires 242.1288.

5-((1*H*-Indol-3-yl)methyl)-3-methyl-1-phenylpyrrolidin-2-one (72)

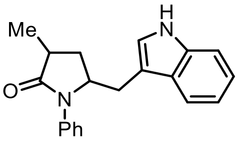

Following **GP6** but stirring the reaction for 48 h, *N*-(2-(1*H*-indol-3-yl)ethyl)aniline **S5** (71 mg, 0.3 mmol) and methyl methacrylate (21 µL, 0.2 mmol) gave **72** (40 mg, 66%) as an inseparable mixture of *trans* and *cis* isomers an oil. *trans*:*cis* = 2.7:1. ^{1}H NMR (500 MHz, $CDCl_3$, diastereomers) δ 8.37–8.22 (1.3H, m), 7.67 (2H, d, *J* = 8.0 Hz), 7.52 (1.4H, d, *J* = 7.9 Hz), 7.49–7.44 (3H, m), 7.35 (1.4H, d, *J* = 8.0 Hz), 7.26 (1H, s), 7.24 (0.5H, s), 7.21 (1.5H, t,

J = 7.8 Hz), 7.14 (1.5H, t, *J* = 7.5 Hz), 6.93 (1H, d, *J* = 2.3 Hz), 6.89 (0.4H, d, *J* = 2.4 Hz), 4.56 (1H, tt, *J* = 8.6, 2.8 Hz), 4.47 (0.3H, tdd, *J* = 9.7, 7.3, 3.4 Hz), 3.28 (0.4H, dd, *J* = 14.4, 3.5 Hz), 3.14 (1H, dd, *J* = 14.6, 3.2 Hz), 2.84 (1H, dd, *J* = 14.6, 8.9 Hz), 2.65 (0.4H, dd, *J* = 14.4, 9.4 Hz), 2.60–2.48 (1.4H, m), 2.38 (0.4H, ddd, *J* = 12.8, 9.0, 6.8 Hz), 2.24 (1H, ddd, *J* = 12.8, 8.6, 2.3 Hz), 1.81 (1H, dt, *J* = 12.8, 8.9 Hz), 1.51 (0.4H, ddd, *J* = 12.8, 10.1, 8.2 Hz), 1.25 (0.9H, d, *J* = 7.1 Hz), 1.19 (3H, d, *J* = 7.1 Hz); ^{13}C NMR (126 MHz, $CDCl_3$, diastereomers) δ 177.3, 177.1, 138.2, 137.8, 136.2 (2 x C), 129.3, 129.2, 127.7 (2 x C), 126.1, 125.5, 124.7, 123.1, 122.8, 122.6, 122.2 (2 x C), 119.6, 119.5, 118.6, 118.4, 111.5, 111.4, 111.1, 110.7, 58.1, 57.8, 37.2, 36.4, 34.0, 32.4, 30.1, 28.2, 16.9, 16.5; HRMS (ASAP) Found MH$^+$ 305.1641, $C_{20}H_{21}N_2O$ requires 305.1648.

5-((1*S*,2*S*,5*S*)-6,6-Dimethylbicyclo[3.1.1]heptan-2-yl)-3-methyl-1-phenylpyrrolidin-2-one (73)

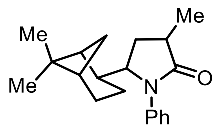

Following **GP6**, *N*-(((1*S*,2*S*,5*S*)-6,6-dimethylbicyclo[3.1.1]heptan-2-yl)methyl)aniline **S6** (69 mg, 0.3 mmol) and methyl methacrylate (21 µL, 0.2 mmol) gave **73** (54 mg, 91%) as separable mixture of *trans* and *cis* isomers. *cis*:*trans* = 2.6:1). The relative configuration was assigned by NOESY experiment.

Data for the *cis* isomer: ^{1}H NMR (400 MHz, $CDCl_3$) δ 7.40–7.30 (4H, m), 7.21–7.10 (1H, m), 4.24 (1H, dd, *J* = 10.9, 7.9 Hz), 2.77 (1H, dp, *J* = 11.7, 7.0 Hz), 2.31–2.08 (3H, m), 1.99–1.79 (4H, m), 1.75 (1H, tt, *J* = 5.8, 2.9 Hz), 1.70 (1H, ddd, *J* = 6.9, 4.8, 2.0 Hz), 1.54–1.39 (1H, m), 1.22 (3H, d, *J* = 7.0 Hz), 0.90 (3H, s), 0.71 (1H, d, *J* = 9.7 Hz), 0.62 (3H, s); ^{13}C NMR (101 MHz, $CDCl_3$) δ 176.8, 140.0, 128.7, 125.5, 124.8, 62.7, 46.0, 42.6, 40.8, 38.1, 35.2, 33.4, 33.2, 27.0, 26.3, 23.0, 20.2, 15.9; HRMS (ASAP) Found MH$^+$ 298.2160, $C_{20}H_{28}NO$ requires 298.2165.

Data for the *trans* isomer: ^{1}H NMR (400 MHz, $CDCl_3$) δ 7.40–7.34 (2H, m), 7.26–7.19 (3H, m), 4.24 (1H, ddd, *J* = 10.1, 7.3, 5.7 Hz), 2.63–2.45 (2H, m), 2.25–2.13 (1H, m), 2.13–2.05 (1H, m), 1.96–1.79 (3H, m), 1.79–1.68 (2H, m), 1.53–1.46 (2H, m), 1.34 (3H, d, *J* = 6.9 Hz), 0.98 (3H, s), 0.75 (3H, s), 0.67 (1H, d, *J* = 9.7 Hz); ^{13}C NMR (101 MHz, $CDCl_3$) δ 177.8, 140.0, 128.8, 126.2, 126.0, 62.8, 49.3, 42.9, 40.8, 37.9, 36.7, 33.6, 32.2, 27.3, 26.2, 23.3, 18.7, 18.0; HRMS (ASAP) Found MH$^+$ 298.2160, $C_{20}H_{28}NO$ requires 298.2165.

5.3 Giese Reactions

4-(Phenylamino)butanenitrile (74)

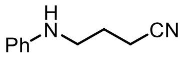

Following **GP6**, *N*-methylaniline (32.5 µL, 0.3 mmol) and acrylonitrile (13 µL, 0.2 mmol) gave **74** (19 mg, 59%) as an oil. ^{1}H NMR (400 MHz, $CDCl_3$) δ 7.24–7.15 (2H, m), 6.74 (1H, tt, *J* = 7.3, 1.1 Hz), 6.67–6.57 (2H, m), 3.70 (1H, brs), 3.32 (2H, t, *J* = 6.6 Hz), 2.48 (2H, t, *J* = 7.1 Hz), 1.97 (2H, p, *J* = 6.8 Hz); ^{13}C NMR (101 MHz, $CDCl_3$) δ 147.6, 129.5, 119.5, 118.1, 113.0, 42.4, 25.4, 14.9. Data in accordance with the literature.[29]

***N,N*-Dimethyl-4-(phenylamino)butanamide (75)**

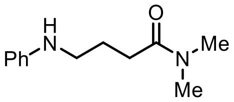

Following **GP6**, *N*-methylaniline (32.5 µL, 0.3 mmol) and *N,N*-dimethylacrylamide (20.5 µL, 0.2 mmol) gave **75** (40 mg, 97%) as a solid. ^{1}H NMR (500 MHz, $CDCl_3$) δ 7.20–7.10 (2H, m), 6.67 (1H, tt, *J* = 7.3, 1.1 Hz), 6.63–6.57 (2H, m), 3.92 (1H, brs), 3.18 (2H, t, *J* = 6.6 Hz), 2.98 (3H, s), 2.95 (3H, s), 2.44 (2H, t, *J* = 7.0 Hz), 1.98 (2H, p, *J* = 6.8 Hz); ^{13}C NMR (126 MHz, $CDCl_3$) δ 172.7, 148.5, 129.3, 117.2, 112.7, 43.8, 37.3, 35.6, 31.1, 24.6. Data in accordance with the literature.[29]

***N*-(3-(Pyridin-3-yl)propyl)aniline (76)**

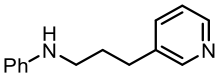

Following **GP6**, *N*-methylaniline (32.5 µL, 0.3 mmol) and 3-vinylpyridine (21.5 µL, 0.2 mmol) gave **76** (25.5 mg, 60%) as an oil. ^{1}H NMR (500 MHz, $CDCl_3$) δ 8.48 (1H, d, *J* = 2.3 Hz), 8.46 (1H, dd, *J* = 4.8, 1.6 Hz), 7.51 (1H, dt, *J* = 7.8, 2.0 Hz), 7.22 (1H, dd, *J* = 7.8, 4.8 Hz), 7.20–7.14 (2H, m), 6.71 (1H, td, *J* = 7.3, 1.1 Hz), 6.64–6.54 (2H, m), 3.41 (1H, brs), 3.17 (2H, t, *J* = 7.0 Hz), 2.74 (2H, t, *J* = 7.6 Hz), 2.01–1.89 (2H, m); ^{13}C NMR (126 MHz, $CDCl_3$) δ 150.0, 148.3, 147.7, 137.0, 135.9, 129.4, 123.5, 117.5, 112.8, 43.3, 30.9, 30.6; HRMS (ASAP) Found MH^+ 213.1388, $C_{14}H_{17}N_2$ requires 213.1386.

3-(1-(Phenylamino)ethyl)cyclohexan-1-one (77)

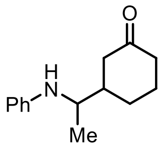

Following **GP6**, *N*-ethylaniline (38 µL, 0.3 mmol) and 2-cyclopentenone (19.5 µL, 0.2 mmol) gave **77** (43.5 mg, quant.) as an inseparable mixture of diastereomers an oil. dr: 1:1. ^{1}H NMR (500 MHz, $CDCl_3$, diastereomers) δ 7.21–7.12 (2H, m), 6.69 (1H, t, *J* = 7.3 Hz), 6.61–6.53 (2H, m), 3.52–3.42 (2H, m), 2.59–2.41 (1H, m), 2.40–2.33 (1H, m), 2.32–2.14 (2H, m), 2.14–2.06 (1H, m), 2.06–1.88 (2H, m), 1.70–1.55 (1H, m), 1.55–1.38 (1H, m), 1.19–1.16 (3H, m); ^{13}C NMR (126 MHz, $CDCl_3$, diastereomers) δ 211.6, 147.5, 147.4, 129.4, 117.2 (2 x C), 113.1 (2 x C), 52.3, 52.1, 44.6, 44.4, 44.2, 44.1, 41.4 (2 x C), 27.4, 27.2, 25.3, 25.2, 17.7 (2 x C). Data in accordance with the literature.[29]

(5*R*)-3-(((1*S*,2*S*,5*S*)-6,6-Dimethylbicyclo[3.1.1]heptan-2-yl)(phenylamino)methyl)-2-methyl-5-(prop-1-en-2-yl)cyclohexan-1-one (78)

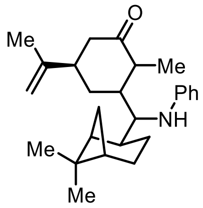

Following **GP6**, *N*-(((1*S*,2*S*,5*S*)-6,6-dimethylbicyclo[3.1.1]heptan-2-yl)methyl)aniline **S6** (69 mg, 0.3 mmol) and (*R*)-(−)-carvone (31 µL, 0.2 mmol) gave **78** (62 mg, 82%) as an oil. dr: 1.3:0.4:0.1:1.2:0.6:0.2:0.4:1. ^{1}H NMR (400 MHz, $CDCl_3$, diastereoisomers) δ 7.19–7.06 (2H, m), 6.73–6.44 (3H, m), 4.94–4.56 (2H, m), 3.93 (0.1H, d, *J* = 10.4 Hz), 3.74–3.42 (0.9H, m), 3.32 (1H, brs), 3.20–3.07 (0.4H, m), 2.87–2.81 (0.2H, m), 2.81–2.64 (0.5H, m), 2.63–1.45 (18H, m), 1.30–1.17 (2.6H, m), 1.17–1.13 (1H, m), 1.13–1.08 (1.4H, m), 1.08–0.99 (2.4H, m), 0.99–0.92 (2.4H, m), 0.92–0.74 (1.7H, m); ^{13}C NMR (101 MHz, $CDCl_3$, diastereoisomers) δ 214.7, 214.5, 214.1, 213.4, 213.0 (2 x C), 212.8, 211.9, 149.6, 149.5, 148.8, 148.6, 148.3, 148.2, 147.9, 147.8, 147.6 (2 x C), 147.5, 147.4, 147.0, 146.8, 146.6, 144.7, 129.6 (2 x C), 129.5 (3 x C), 129.4 (2 x C), 129.3, 117.2, 117.0 (2 x C), 116.8 (2 x C), 116.5, 115.2, 113.2, 112.9 (2 x C), 112.8, 112.7, 112.5, 112.3, 112.2, 111.6, 111.3, 110.6, 110.5, 110.2, 110.1 (2 x C), 110.0, 109.7, 61.6, 61.3, 61.1, 60.4, 58.4, 57.7, 57.6, 57.2, 49.3, 48.6, 47.6, 47.1 (2 x C), 47.0, 46.9 (3 x C), 46.7, 46.5, 46.4, 46.2 (2 x C), 46.1, 46.0 (3 x C), 45.9, 45.8, 45.2, 44.9, 44.8, 44.7, 44.6, 44.5, 44.4, 44.2, 44.1, 44.0, 43.7, 43.3 (2 x C), 43.1, 42.8 (2 x C), 42.6, 42.4

(3 x C), 42.3, 42.2, 41.9, 41.7, 41.5, 41.4 (2 x C), 41.3 (3 x C), 40.5, 40.4 (2 x C), 40.2, 40.0, 38.7, 38.6 (2 x C), 38.5, 38.4 (2 x C), 35.9, 35.1, 35.0, 34.9, 34.7, 34.2 (2 x C), 33.6, 33.0, 32.4, 31.3, 30.9, 30.7, 30.1, 29.8, 29.6 (2 x C), 28.3 (2 x C), 28.1 (2 x C), 27.9, 27.8, 26.8 (2 x C), 26.7 (2 x C), 26.6, 26.5, 26.1 (2 x C), 25.4, 23.7, 23.6 (2 x C), 23.4 (2 x C), 23.3, 23.2, 22.4, 21.7, 21.6, 21.3, 21.0, 20.9, 20.7 (2 x C), 20.6 (2 x C), 20.5 (2 x C), 20.3, 19.6, 19.2, 15.8, 15.7, 14.5, 14.2, 14.1, 13.2, 12.7, 12.4; HRMS (ESI) Found MH^+ 380.2942, $C_{26}H_{38}NO$ requires 380.2948.

5.4 α-*N* sp[3] C–H Arylation

General Procedure for the α-*N* sp[3] C–H Arylation Reaction – GP7

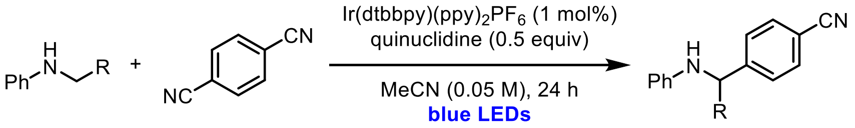

A dry tube equipped with a stirring bar was charged with the Ir(dtbbpy)(ppy)$_2$PF$_6$ (2 mg, 2 µmol, 1 mol%), 1,4-dicyanobenzene (25.5 mg, 0.2 mmol, 1.0 equiv.), quinuclidine (11 mg, 0.10 mmol, 0.5 equiv) and the *N*-alkyl aniline (if solid) (0.30 mmol, 1.5 equiv.). The tube was capped with a Supelco aluminium crimp seal with septum (PTFE/butyl), then evacuated under high vacuum and backfilled with N_2 (x 3). Degassed CH_3CN (0.05 M) and *N*-alkyl aniline (if liquid) (0.30 mmol, 1.5 equiv.) were sequentially added. The vial was purged with a stream of N_2 and the lid sealed with parafilm and placed approximately 4 cm from blue LEDs. The blue LEDs were switched on and the mixture was stirred under irradiation without fan for 24 hours. The tube was opened, and the solvent was evaporated under reduced. The crude was purified by flash column chromatography on silica gel with Hexane–EtOAc.

4-((Phenylamino)methyl)benzonitrile (S10)

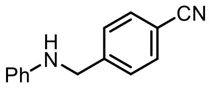

Following **GP7**, *N*-methylaniline (32.5 µL, 0.3 mmol) and 1,4-dicyanobenzene (25.5 mg, 0.2 mmol) gave **S10** (29 mg, 69%) as a solid. ^{1}H NMR (500 MHz, $CDCl_3$) δ 7.62 (2H, d, *J* = 8.0 Hz), 7.48 (2H, d, *J* = 8.0 Hz), 7.17 (2H, t, *J* = 7.7 Hz), 6.74 (1H, t, *J* = 7.3 Hz), 6.58 (2H, d, *J* = 8.0 Hz), 4.43 (2H, s), 4.20 (1H, brs); ^{13}C NMR (126 MHz, $CDCl_3$) δ 147.5, 145.5, 132.6, 129.5, 127.8, 119.0, 118.3, 113.0, 111.1, 47.9. Data in accordance with the literature.[30]

4-(1-(Phenylamino)ethyl)benzonitrile (79)

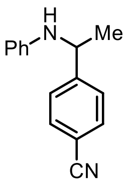

Following **GP7**, *N*-ethylaniline (38 µL, 0.3 mmol) and 1,4-dicyanobenzene (25.5 mg, 0.2 mmol) gave **79** (36.5 mg, 82%) as a solid. ^{1}H NMR (400 MHz, $CDCl_3$) δ 7.65–7.57 (2H, m), 7.53–7.46 (2H, m), 7.14–7.05 (2H, m), 6.69 (1H, tt, *J* = 7.3, 1.1 Hz), 6.50–6.41 (2H, m), 4.52 (1H, q, *J* = 6.8 Hz), 4.20 (1H, brs), 1.53 (3H, d, *J* = 6.8 Hz); ^{13}C NMR (101 MHz, $CDCl_3$) δ

151.0, 146.6, 132.7, 129.3, 126.8, 119.1, 118.1, 113.5, 110.9, 53.7, 25.0. Data in accordance with the literature.[30]

4-(2-Cyano-1-(phenylamino)ethyl)benzonitrile (80)

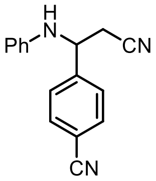

Following **GP7**, 3-anilinopropionitrile (44 mg, 0.3 mmol) and 1,4-dicyanobenzene (25.5 mg, 0.2 mmol) gave **80** (37 mg, 75%) as a solid. ^{1}H NMR (500 MHz, $CDCl_3$) δ 7.72–7.66 (2H, m), 7.59–7.54 (2H, m), 7.19–7.12 (2H, m), 6.79 (1H, t, *J* = 7.4 Hz), 6.58–6.52 (2H, m), 4.81 (1H, t, *J* = 6.0 Hz), 4.32 (1H, brs), 2.96 (1H, dd, *J* = 16.8, 6.0 Hz), 2.89 (1H, dd, *J* = 16.8, 6.0 Hz); ^{13}C NMR (101 MHz, $CDCl_3$) δ 145.3, 145.1, 133.2, 129.6, 127.3, 119.6, 118.4, 116.4, 114.1, 112.7, 54.2, 26.4. Data in accordance with the literature.[31]

4-(2-((*tert*-Butyldimethylsilyl)oxy)-1-(phenylamino)ethyl)benzonitrile (81)

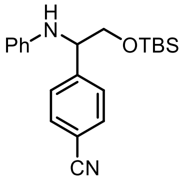

Following **GP7**, *N*-(2-((*tert*-butyldimethylsilyl)oxy)ethyl)aniline **S2** (75.5 mg, 0.3 mmol) and 1,4-dicyanobenzene (25.5 mg, 0.2 mmol) gave **81** (54 mg, 77%) as an oil. ^{1}H NMR (400 MHz, $CDCl_3$) δ 7.62 (2H, d, *J* = 8.3 Hz), 7.51 (2H, d, *J* = 8.3 Hz), 7.15–7.04 (2H, m), 6.71 (1H, t, *J* = 7.3 Hz), 6.48 (2H, d, *J* = 7.9 Hz), 4.84 (1H, brs), 4.42 (1H, dd, *J* = 6.8, 4.3 Hz), 3.92 (1H, dd, *J* = 10.1, 4.3 Hz), 3.68 (1H, dd, *J* = 10.1, 6.8 Hz), 0.87 (9H, s), 0.00 (3H, s), −0.02 (3H, s); ^{13}C NMR (101 MHz, $CDCl_3$) δ 146.8, 132.5, 129.3, 128.0, 119.0, 118.5, 114.2, 111.4, 67.2, 60.2, 25.9, 18.4, −5.3, −5.4; HRMS (ASAP) Found MH^+ 353.2042, $C_{21}H_{29}N_2OSi$ requires 353.2044.

4-(2-(Cyclohex-1-en-1-yl)-1-(phenylamino)ethyl)benzonitrile (82)

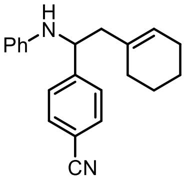

Following **GP7**, *N*-(2-(cyclohex-1-en-1-yl)ethyl)aniline **S3** (60.5 mg, 0.3 mmol) and 1,4-dicyanobenzene (25.5 mg, 0.2 mmol) gave **82** (41 mg, 68%) as an oil. ^{1}H NMR (500 MHz, $CDCl_3$) δ 7.61 (2H, d, *J* = 8.2 Hz), 7.51 (2H, d, *J* = 8.1 Hz), 7.12–7.05 (2H, m), 6.69 (1H, t, *J* = 7.3 Hz), 6.42 (2H, d, *J* = 8.0 Hz), 5.58 (1H, dt, *J* = 3.8, 1.9 Hz), 4.37 (1H, dd, *J* = 10.2, 4.7 Hz), 4.21 (1H, brs), 2.45–2.39 (1H, m), 2.26 (1H, dd, *J* = 14.0, 10.2 Hz), 2.12–1.99 (2H, m), 1.99–1.85 (2H, m), 1.64–1.51 (4H, m); ^{13}C NMR (126 MHz, $CDCl_3$) δ 150.7, 147.2, 133.8, 132.7, 129.3, 127.1, 126.5, 119.1, 118.1, 113.7, 110.8, 55.8, 48.2, 27.7, 25.5, 22.8, 22.4; HRMS (ASAP) Found MH$^+$ 331.2164, $C_{23}H_{27}N_2$ requires 331.2169.

4-(2-(1*H*-Indol-3-yl)-1-(phenylamino)ethyl)benzonitrile (83)

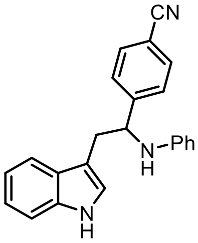

Following **GP7**, *N*-(2-(1*H*-indol-3-yl)ethyl)aniline **S5** (71 mg, 0.3 mmol) and 1,4-dicyanobenzene (25.5 mg, 0.2 mmol) gave **83** (25 mg, 37%) as a solid. ^{1}H NMR (400 MHz, $CDCl_3$) δ 8.05 (1H, s), 7.62–7.56 (2H, m), 7.50 (1H, d, *J* = 7.9 Hz), 7.48–7.44 (2H, m), 7.40–7.36 (1H, m), 7.25–7.21 (1H, m), 7.16–7.11 (1H, m), 7.08–7.02 (2H, m), 6.87 (1H, d, *J* = 2.4 Hz), 6.66 (1H, tt, *J* = 7.3, 1.2 Hz), 6.43–6.36 (2H, m), 4.74 (1H, dd, *J* = 7.8, 5.6 Hz), 4.38 (1H, brs), 3.28 (1H, dd, *J* = 14.7, 5.6 Hz), 3.19 (1H, dd, *J* = 14.7, 7.8 Hz); ^{13}C NMR (101 MHz, $CDCl_3$) δ 149.9, 146.8, 136.4, 132.6, 129.3, 127.5, 127.4, 122.9, 122.6, 120.0, 119.1, 118.6, 118.2, 113.8, 111.5, 111.1, 110.9, 58.3, 34.8; HRMS (ESI) Found MH$^+$ 338.1638, $C_{23}H_{20}N_3$ requires 338.1652.

4-(((1*S*,2*S*,5*S*)-6,6-Dimethylbicyclo[3.1.1]heptan-2-yl)(phenylamino)methyl)benzonitrile (84)

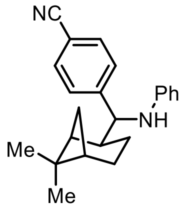

Following **GP7**, *N*-(((1*S*,2*S*,5*S*)-6,6-dimethylbicyclo[3.1.1]heptan-2-yl)methyl)aniline **S6** (69 mg, 0.3 mmol) and 1,4-dicyanobenzene (25.5 mg, 0.2 mmol) gave **84** (33 mg, 50%) as an inseparable mixture of diastereomers an oil. dr = 1.9:1. ^{1}H NMR (400 MHz, $CDCl_3$, diastereomers) δ 7.38 (1H, d, *J* = 3.3 Hz), 7.36 (2H, d, *J* = 3.3 Hz), 7.22 (2H, d, *J* = 7.8 Hz), 7.18 (1H, d, *J* = 7.9 Hz), 6.87–6.80 (3H, m), 6.45–6.38 (1.5H, m), 6.30–6.19 (3H, m), 4.15 (0.5H, d, *J* = 10.1 Hz), 4.10 (1H, d, *J* = 10.4 Hz), 3.90 (1.5H, brs), 2.26–2.20 (1.5H, m), 2.10–1.91 (3H, m), 1.76–1.64 (4.5H, m), 1.61–1.47 (1.5H, m), 1.25–1.13 (1.5H, m), 0.99 (3H, s), 0.99 (1.6H, s), 0.92 (3H, s), 0.91 (1.6H, s), 0.67 (1H, d, *J* = 9.4 Hz), 0.60 (0.6H, d, *J* = 9.9 Hz); ^{13}C NMR (126 MHz, $CDCl_3$, diastereomers) δ 149.9, 149.8, 147.2, 147.0, 132.4 (2 x C), 129.3 (2 x C), 129.2, 128.4, 119.0 (2 x C), 117.8, 117.7, 113.3, 113.2, 110.9 (2 x C), 63.8, 62.0, 48.2, 48.0, 43.0, 42.6, 41.4, 41.1, 38.8, 38.4, 33.9, 33.2, 28.2, 28.0, 26.3, 26.2, 24.3, 23.2, 20.6, 20.0; HRMS (ASAP) Found MH^+ 331.2164, $C_{23}H_{27}N_2$ requires 331.2169.

5.5 Addition to Imines

***N*¹,*N*²,1-Triphenylethane-1,2-diamine (85)**

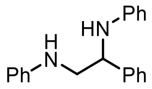

Following **GP6**, *N*-methylaniline (32.5 µL, 0.3 mmol) and (*E*)-*N*,1-diphenylmethanimine (36 mg, 0.2 mmol) gave **85** (34 mg, 59%) as an oil. ^{1}H NMR (400 MHz, $CDCl_3$) δ 7.43–7.33 (4H, m), 7.33–7.26 (1H, m), 7.24–7.17 (2H, m), 7.14–7.07 (2H, m), 6.79 (1H, tt, *J* = 7.3, 1.1 Hz), 6.74–6.65 (3H, m), 6.62–6.54 (2H, m), 4.65 (1H, dd, *J* = 8.1, 4.8 Hz), 4.57 (2H, brs), 3.53 (1H, dd, *J* = 12.6, 4.8 Hz), 3.44 (1H, dd, *J* = 12.6, 8.1 Hz); ^{13}C NMR (101 MHz, $CDCl_3$) δ 147.3, 146.9, 141.2, 129.6, 129.3, 129.0, 127.8, 126.7, 118.9, 118.2, 114.0, 113.9, 57.6, 51.3. Data in accordance with the literature.[32]

6 Mechanistic Considerations

6.1 Cyclic Voltammetry and Emission Quenching Studies

Cyclic Voltammetry General Experimental Details

Cyclic voltammetry was conducted on an EmStat (PalmSens) potentiostat using a 3-electrode cell configuration. A glassy carbon working electrode was employed alongside a platinum wire counter electrode and a Ag/AgCl reference electrode. All the solutions were degassed by bubbling N_2 prior to measurements. 5 mM solutions of the desired compounds were freshly prepared in dry acetonitrile along with 0.1 M of tetrabutylammonium hexafluorophosphate as supporting electrolyte and were examined at a scan rate of 0.1 V s^{-1}. Ferrocene ($E_{1/2}$ = +0.42 V vs SCE)[33] was added at the end of the measurements as an internal standard to determine the precise potential scale. Potential values are given versus the saturated calomel electrode (SCE). Irreversible waves were obtained in all cases; therefore, the potentials were estimated at half the maximum current, as previously described by Nicewicz.[34] Oxidation potentials higher than +1.5 V vs SCE was not determined.

Emission Quenching Studies General Experimental Details

Stern-Volmer experiments were carried out monitoring the emission intensity of argon-degassed solutions of $[Ir(ppy)_2(dtbbpy)]PF_6$ (5 x 10^{-5} M) containing variable amounts of the quencher in dry acetonitrile. The reported excited-state lifetime for $[Ir(ppy)_2(dtbbpy)]PF_6$ in MeCN (0.56 μs) was used for k_q calculations.[35]

Table S5.

Ir(dtbbpy)(ppy)$_2$]PF$_6$ redox properties			
***E_{red} (V vs SCE)**	***E_{ox} (V vs SCE)**	**E_{red} (V vs SCE)**	**E_{ox} (V vs SCE)**
+0.66	–0.96	–1.51	+1.21

Table S6.

	**E_{ox} (V vs SCE)**	**k_{SV} (M^{-1} s^{-1})**	**k_q (M^{-1} s^{-1})**
PhNHMe	+0.81	1277	2.3 10^9
Et_3N	+0.80	83	–
piperidine	+1.09	27	–
quniclidine	+1.05	40	–
PhNHMe + Et_3N	+0.76; +0.81	1388	2.5 10^9
PhNHMe + piperidine	+0.67; +0.81	1304	2.3 10^9
PhNHMe + quinuclidine	+0.65; +0.81	1364	2.4 10^9
$PhNH_2$	+0.98	64	–
N-Me-*N*-Bn-amine	+1.11	21	–

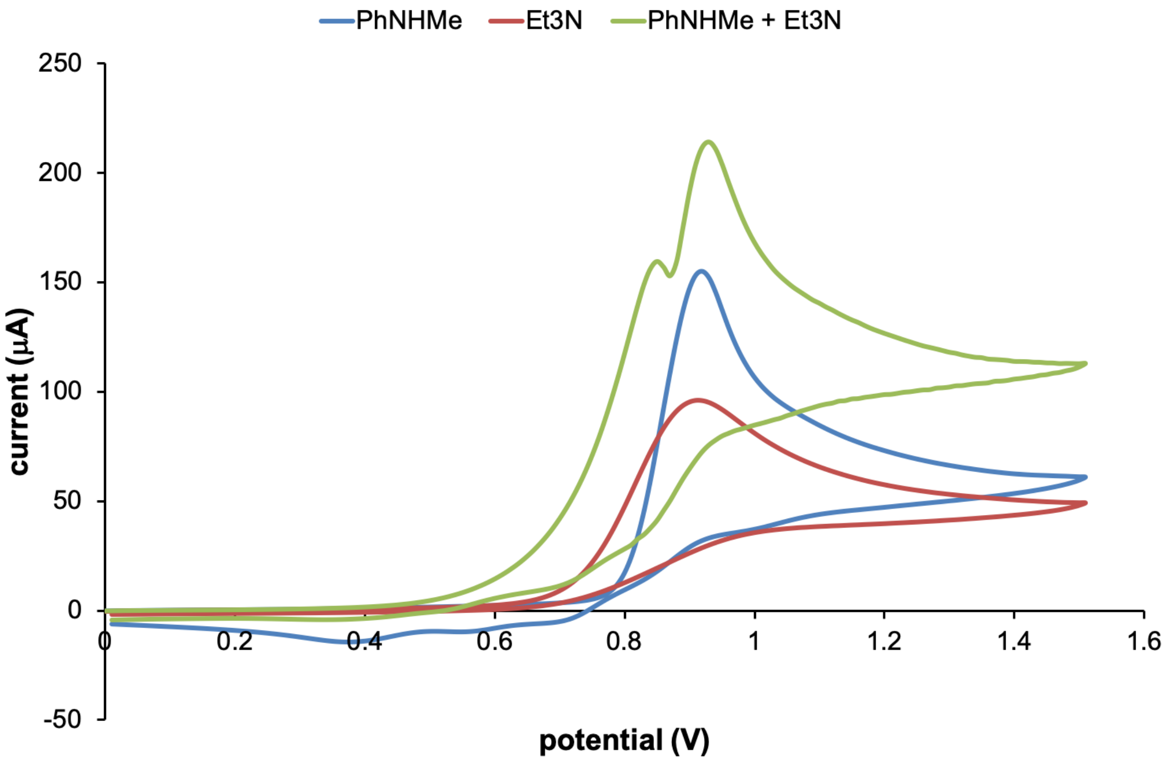

Figure S2.

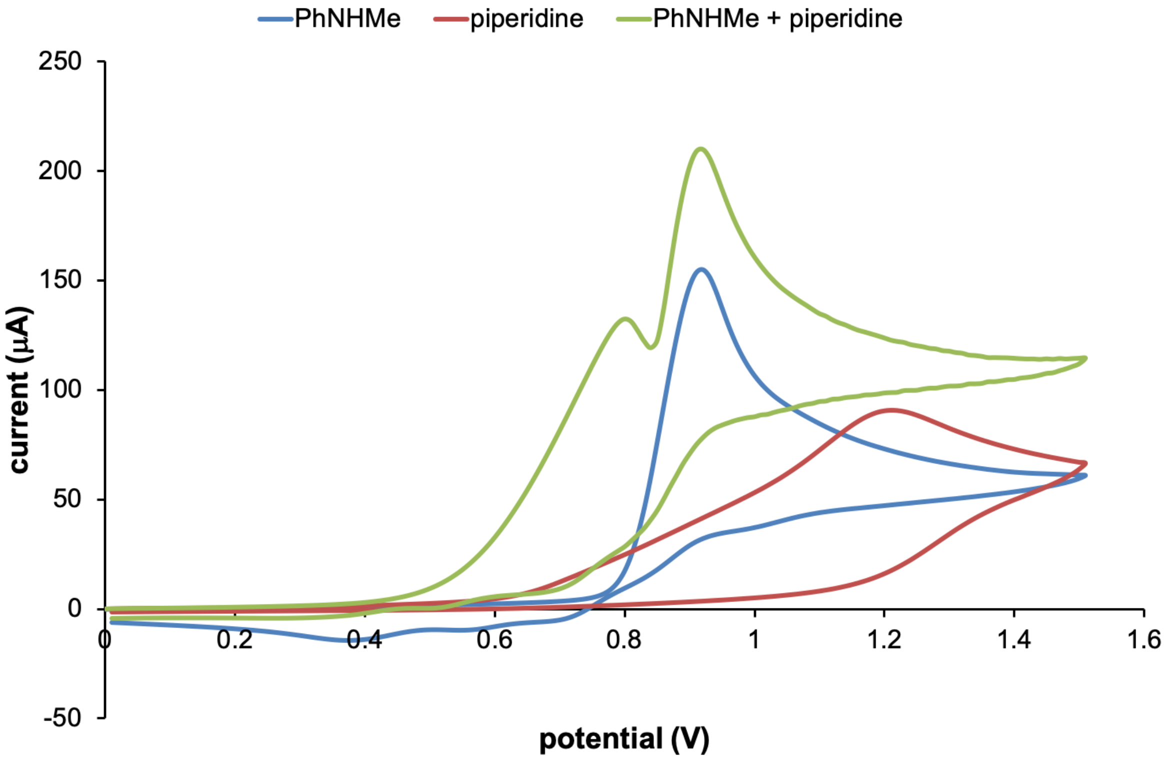

Figure S3.

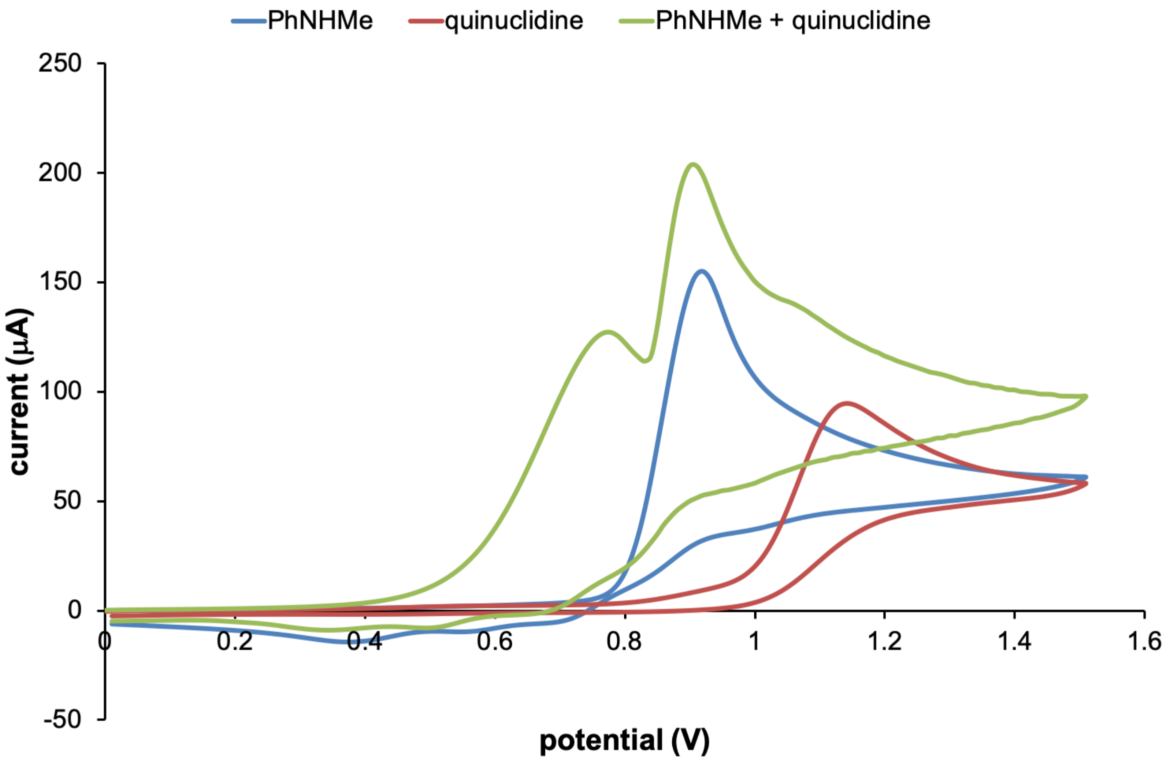

Figure S4.

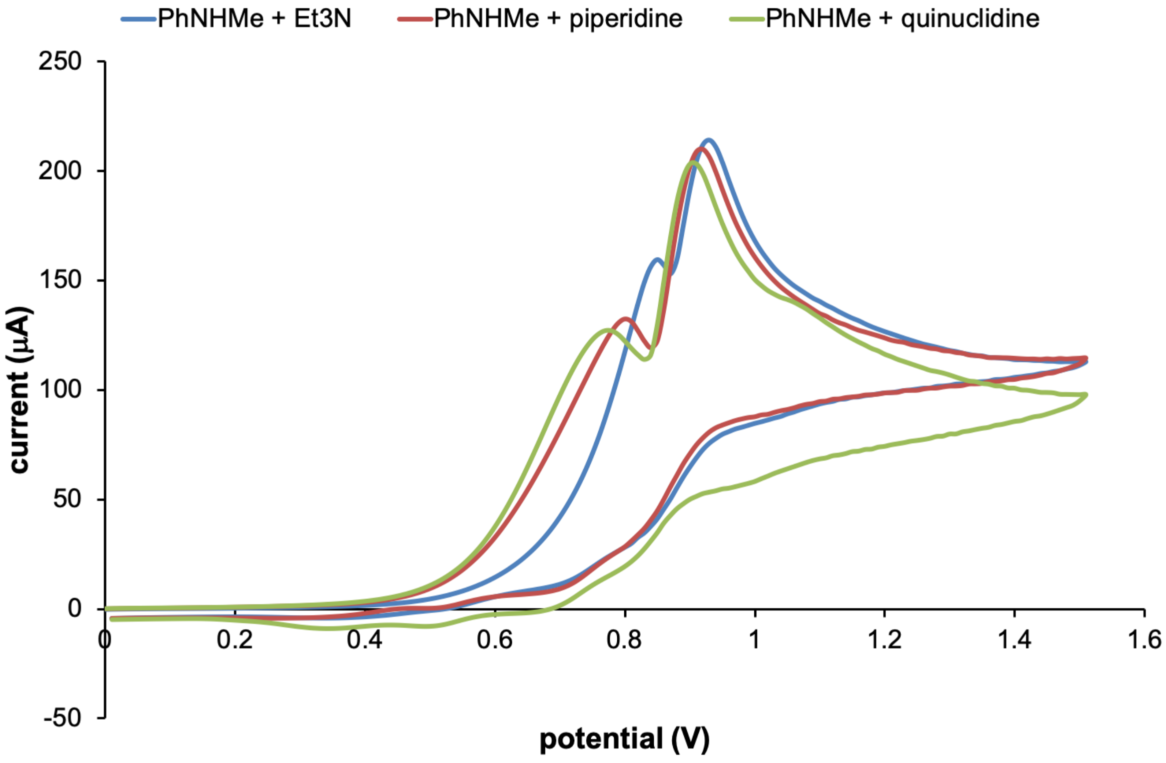

Figure S5.

The peak for the new species observed in the CV upon mixing PhNHMe with Et_3N and piperidine increases its intensity upon increasing the amount of the two amines which is in line with a stronger binding.

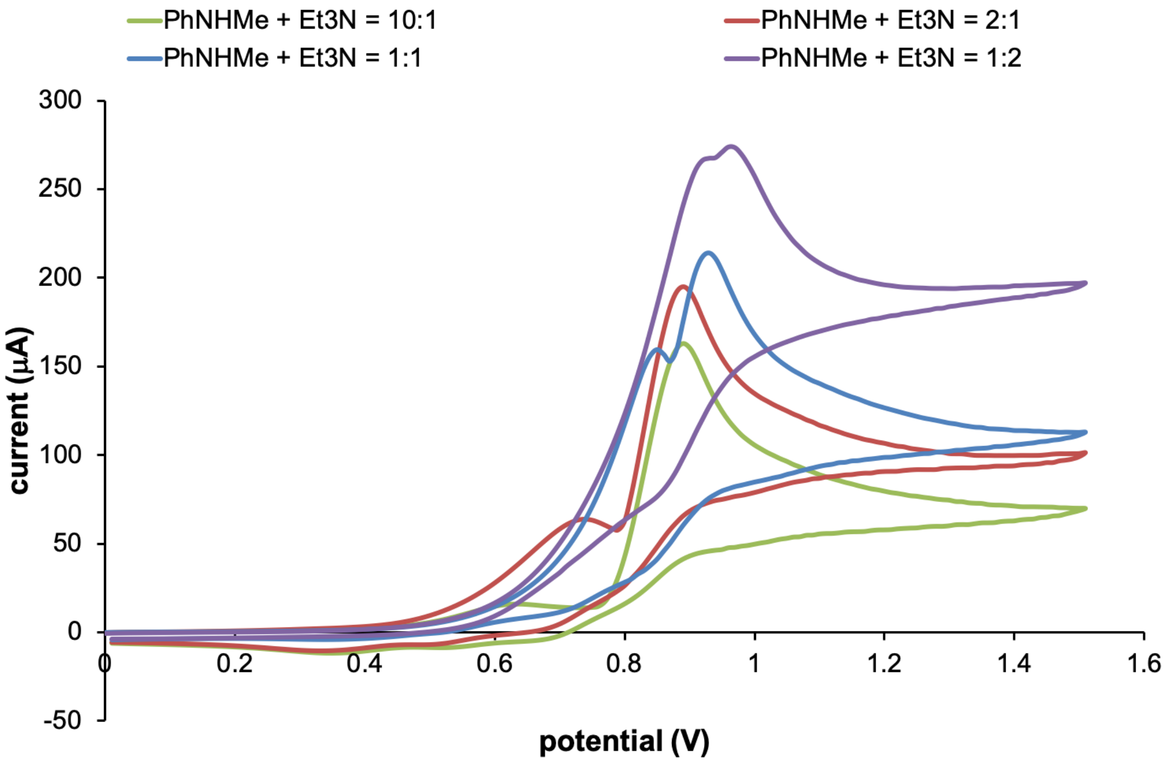

Figure S6.

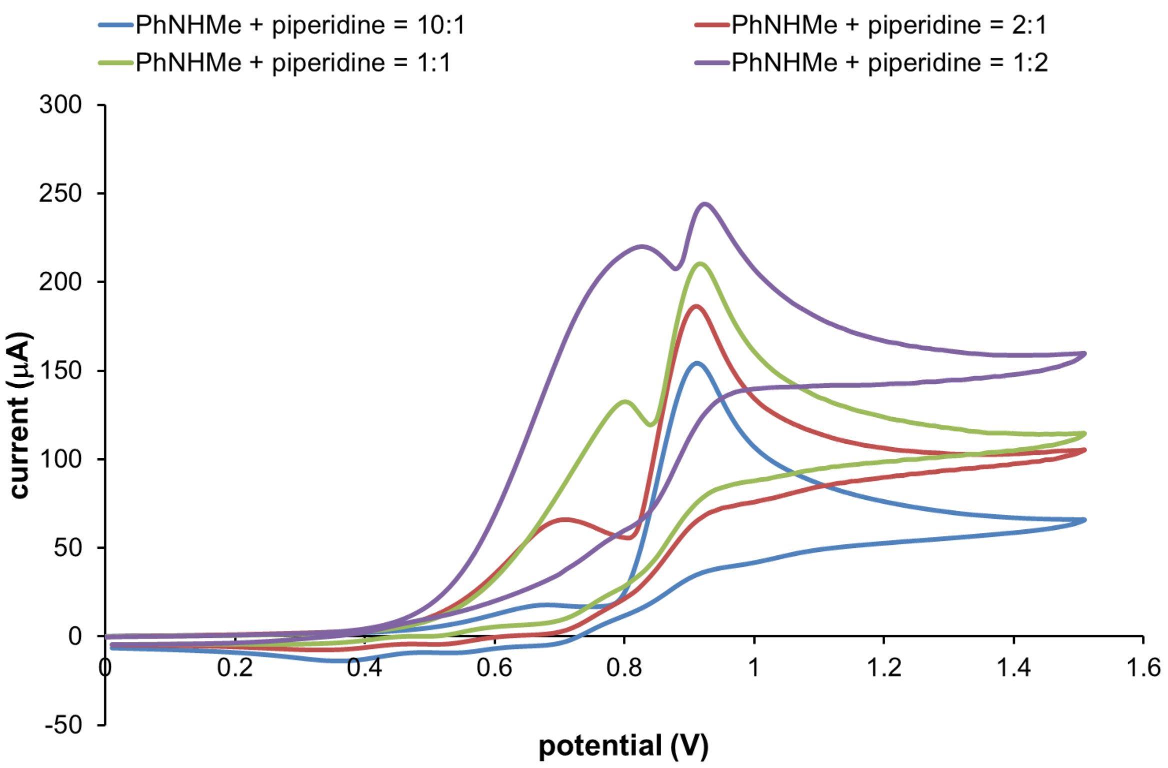

Figure S7.

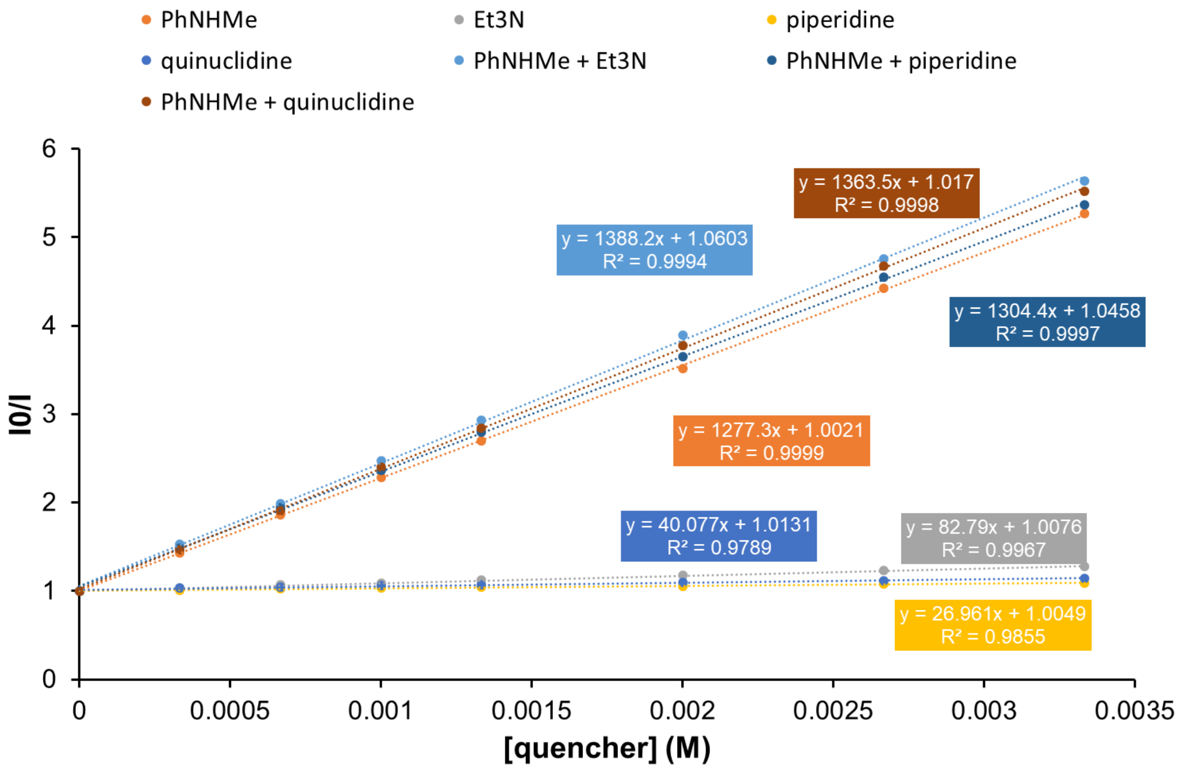

Figure S8.

In the case of BnNHMe no new peak was observed upon addition of Et_3N or piperidine. As a result, this substrate could not be used in the chemistry.

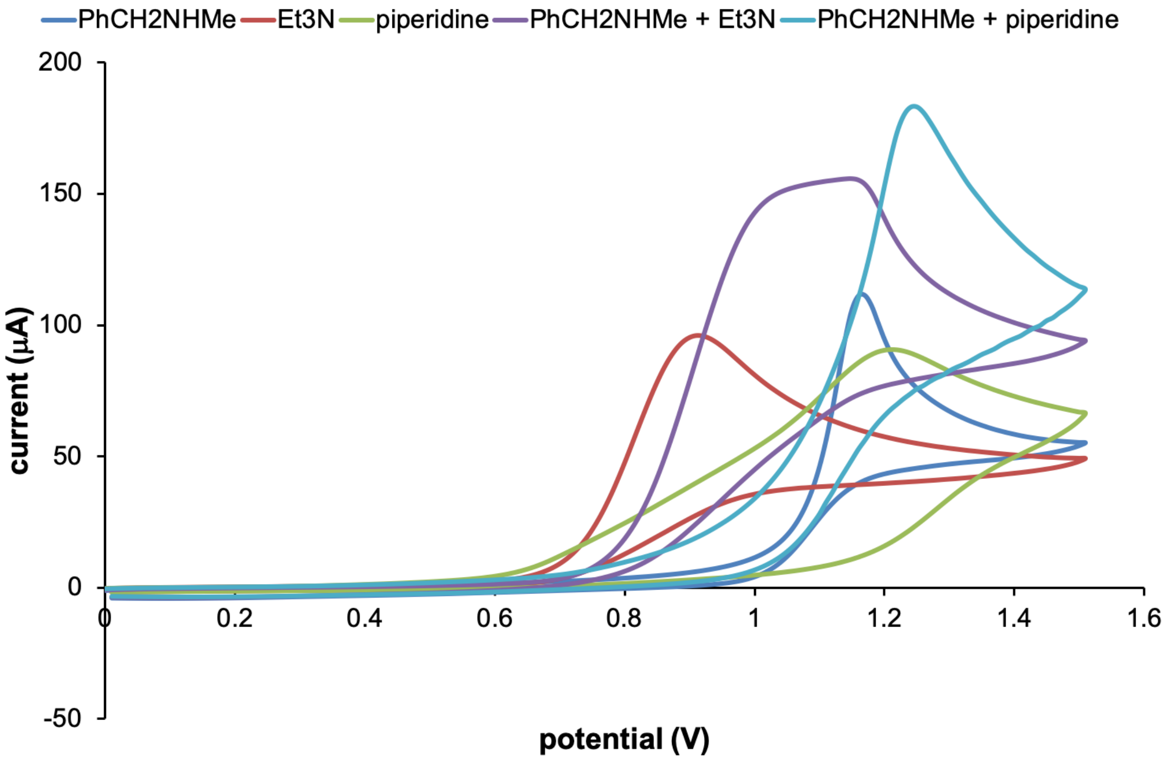

Figure S9.

In the case of $PhNMe_2$ no new peak was observed upon addition of Et_3N or piperidine as this substrate does not contain a N–H for binding. As BET is not a problem, $PhNMe_2$ can however be engaged in redox chemistry as discussed in the manuscript.

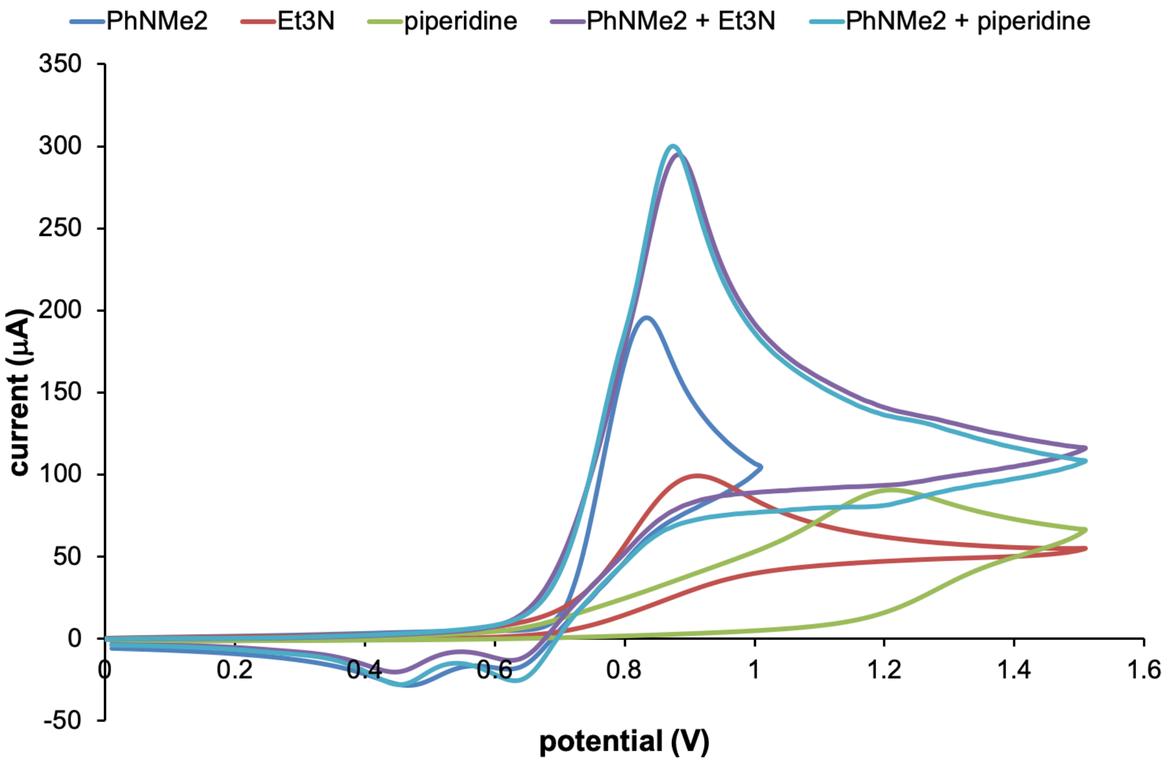

Figure S10.

In the case of *o*-CO_2Me-PhNHMe, 4CzIPN proved to be the optimum photocatalyst. In this case the o-electron withdrawing group renders the initial oxidation more difficult but still leads to the formation of a new species with a lower oxidation potential upon addition of Et_3N and piperidine.

Stern-Volmer experiments were carried out monitoring the emission intensity of argon-degassed solutions of 4CzIPN (5×10^{-5} M) containing variable amounts of the quencher in dry acetonitrile.

Table S7.

	E_{ox} (V vs SCE)	k_{SV} (M^{-1} s^{-1})
o-CO_2Me-PhNHMe	+1.07	329
Et_3N	+0.80	238
piperidine	+1.09	181
o-CO_2Me-PhNHMe + Et_3N	+0.82; +1.07	484
o-CO_2Me-PhNHMe + piperidine	+0.97; +1.07	424

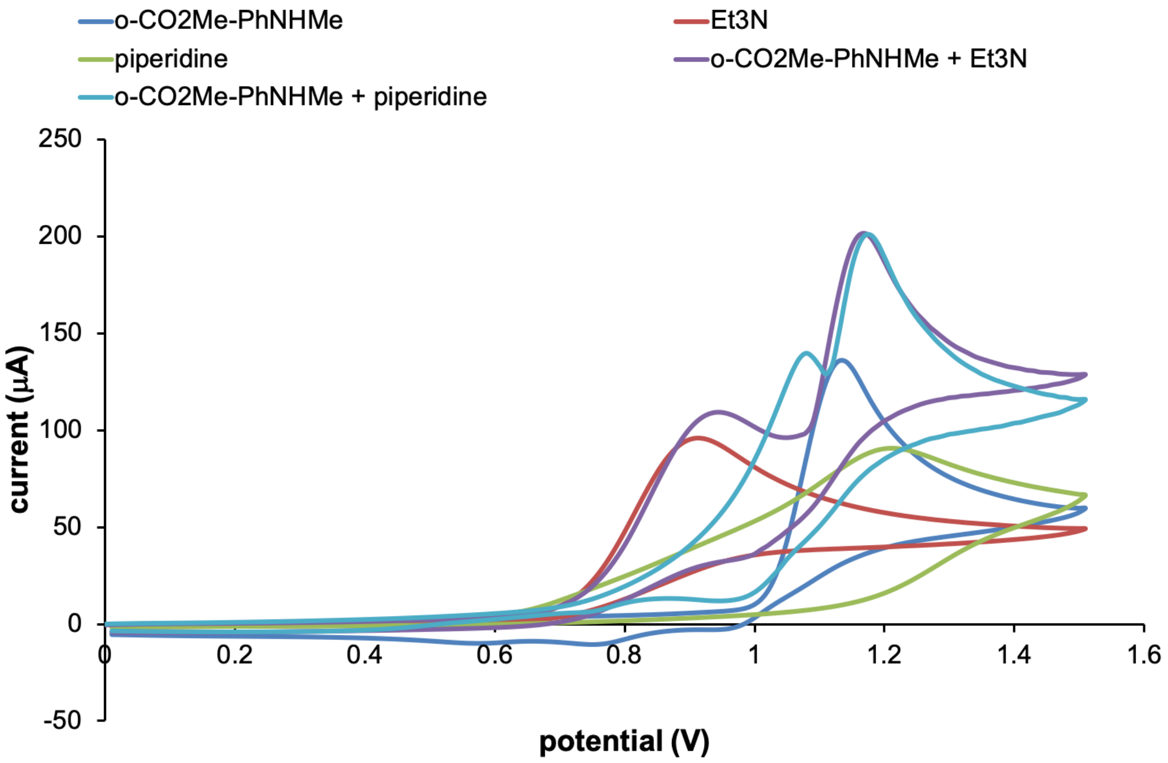

Figure S11.

Figure S12 demonstrates that the intensity of peak resulting from the interaction of Et_3N with the ArNHMe increases in the presence of electron withdrawing groups on the aromatic group as they lead to a more acidic N–H bond.

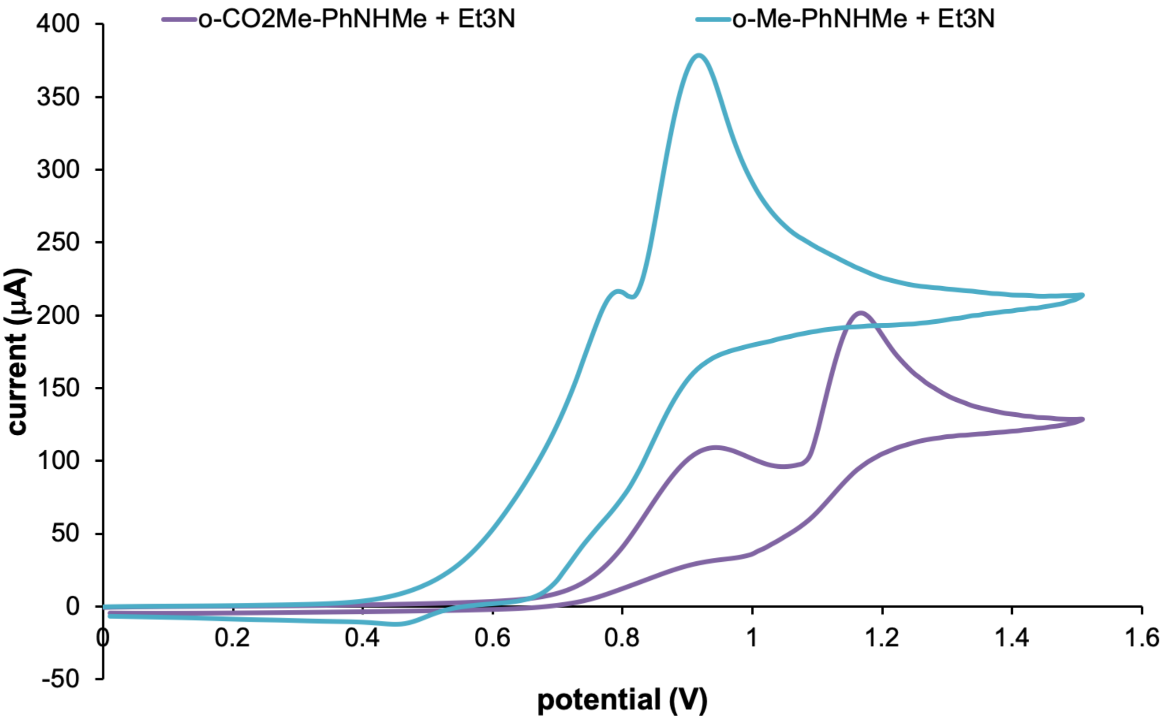
o-CO2Me-PhNHMe + Et3N
o-Me-PhNHMe + Et3N
400
350
300
250
200
150
100
50
0
-50
current (μA)
0
0.2
0.4
0.6
0.8
1
1.2
1.4
1.6
potential (V)

Figure S12.

As the interaction between PhNHMe and the alkyl amines should be weak, the use of a strongly H-bonding accepting solvent should disrupt this interaction.

Indeed, when repeating the cyclic voltammeter studies in DMF and DMSO a noticeable decrease of the second peak intensity was observed (Figure S13).

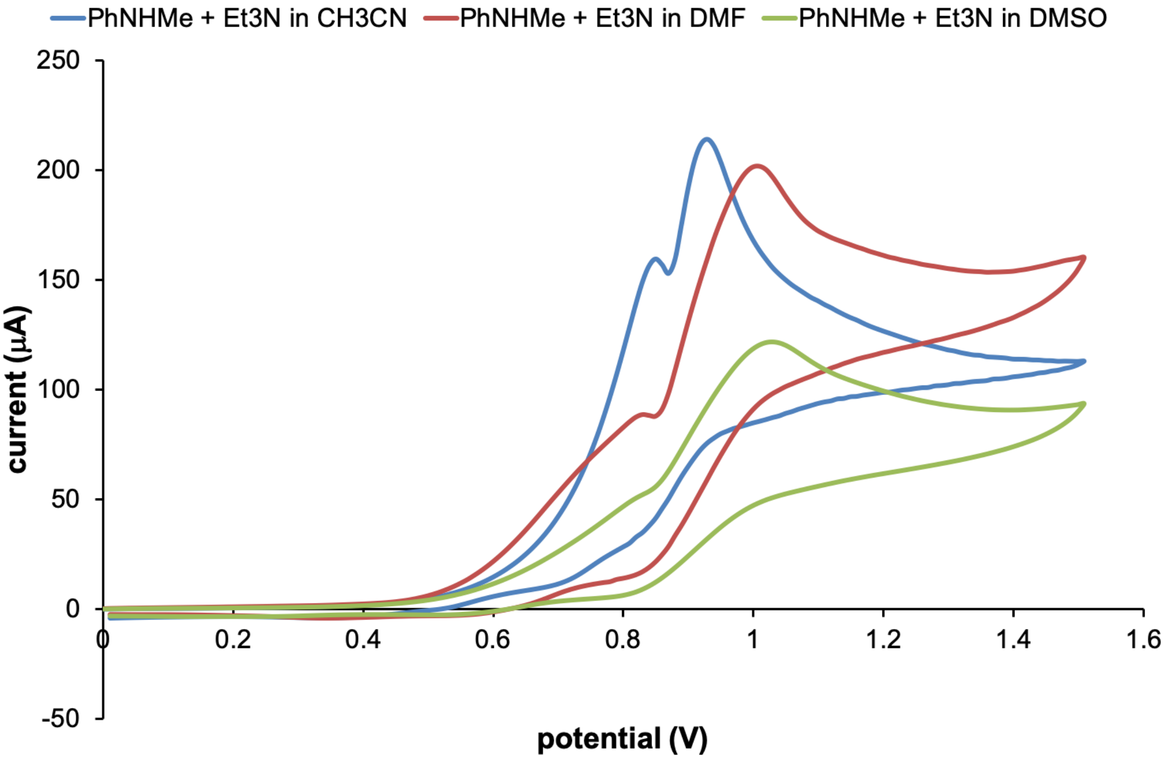

Figure S13.

6.2 Detection of H_2

The reaction depicted in Scheme S1 was run in an J. Young tube NMR and immediately analysed by ^{1}H NMR spectroscopy which revealed the formation of H_2 (Figure S14).

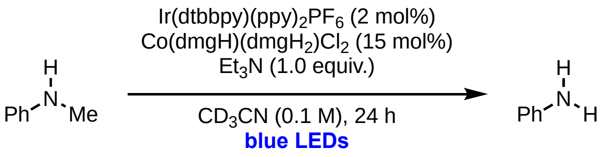

Scheme S1.

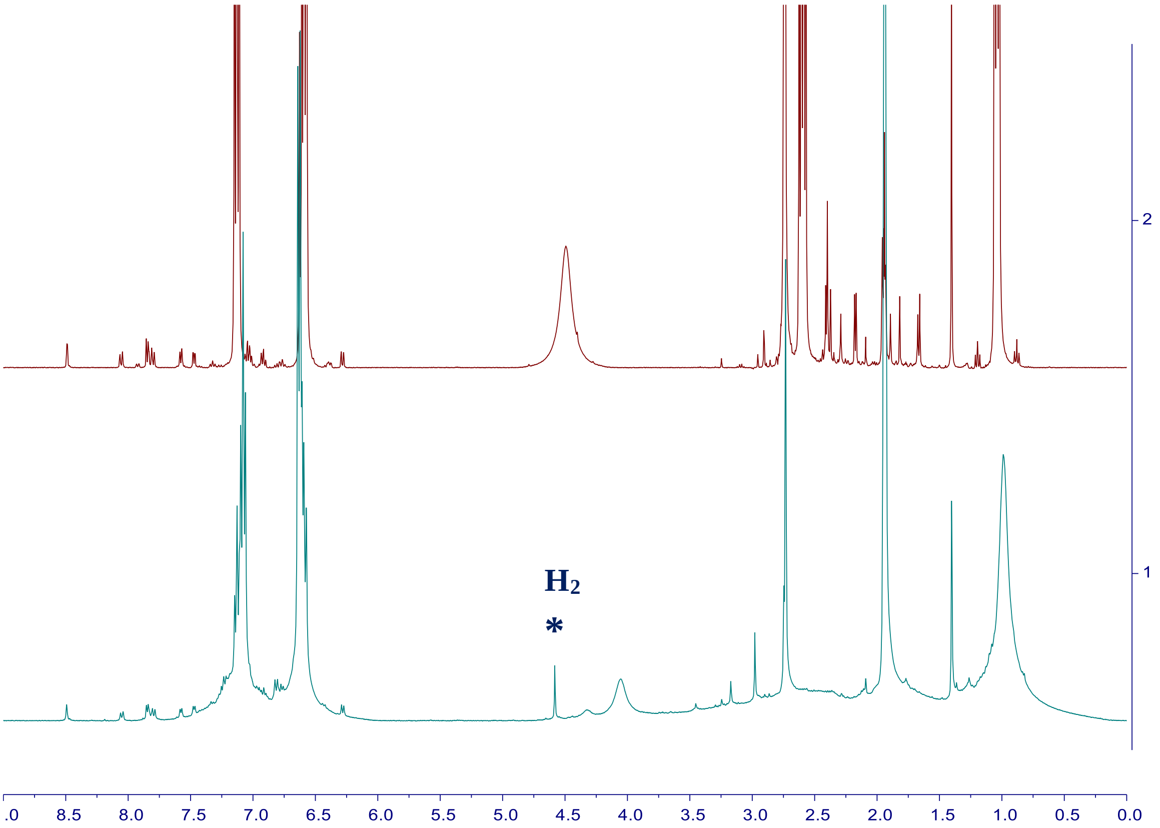

Figure S14.

6.3 Detection of Formaldehyde

The reaction depicted in Scheme S2 was run following the **GP5** and analysed by ^{1}H NMR spectroscopy which revealed the formation of aldehyde (Figure S15).

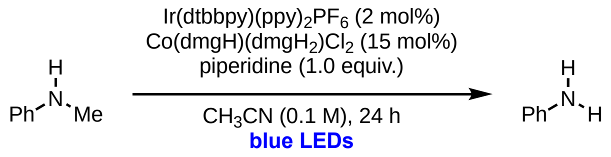

Scheme S2.

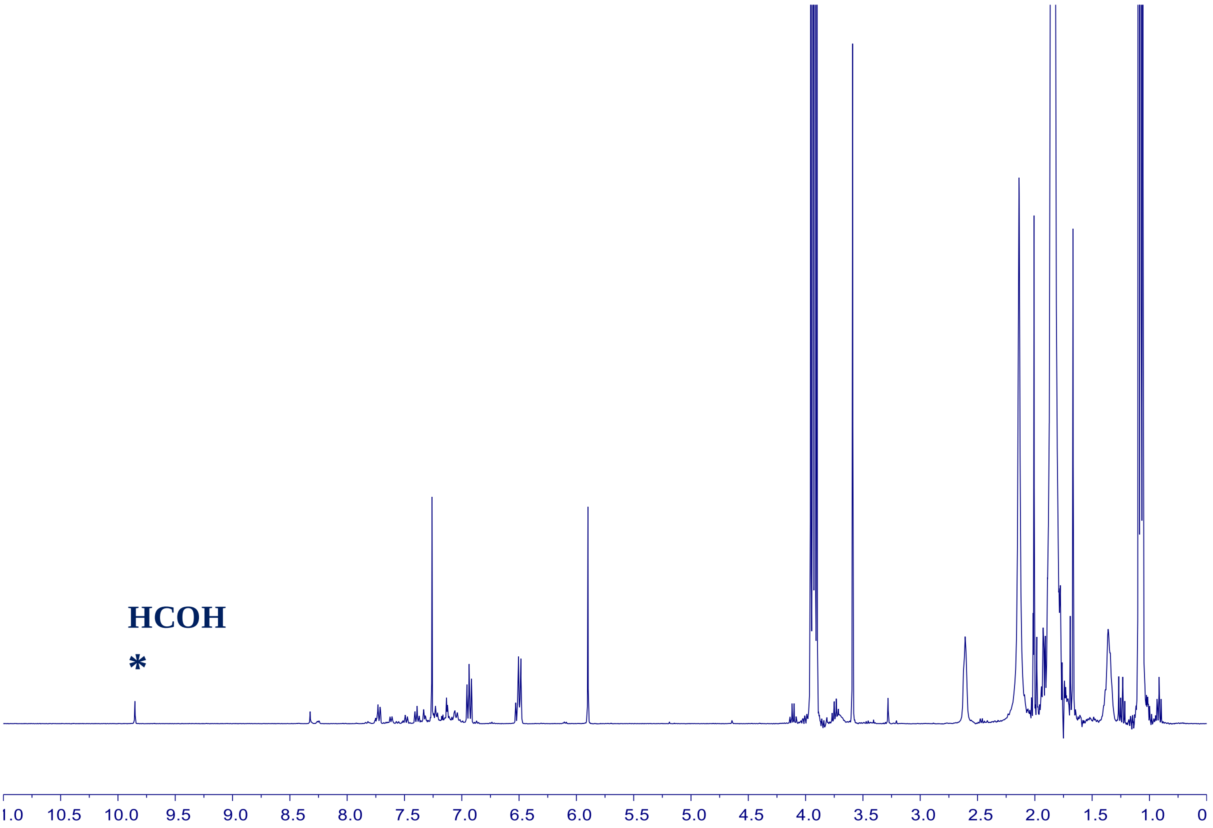

Figure S15.

6.4 Quantum Yields (Φ) Determination

The quantum yield of the photochemical reaction depicted in Scheme S3 was determined at 298 K following procedures described in literature.[36] Degassed reaction tubes were irradiated using as the light source blue LEDs plates (λ_{max} = 444 nm) for 30-60 min. The yield of products was determined by ^{1}H NMR spectroscopy; in all cases the conversion was lower than 20%. The photon flux of the blue LEDs used was determined by standard ferrioxalate actinometry.

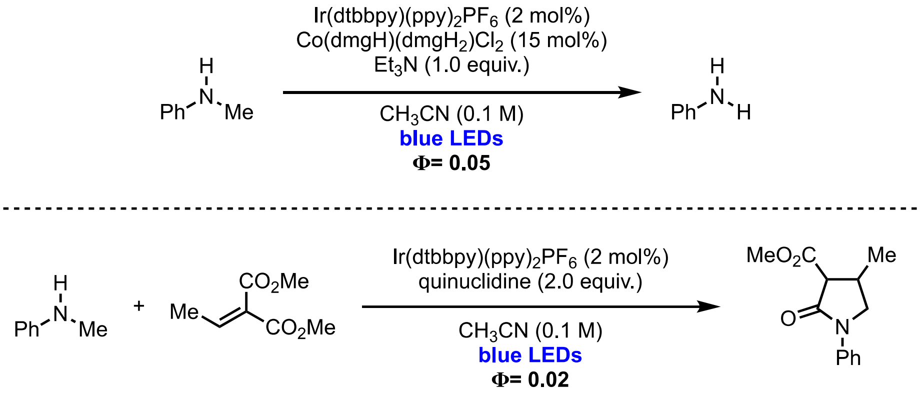

Scheme S3.

These results demonstrate that efficient radical chain propagations are not operating under these reaction conditions.

7 NMR Spectra

S1 – ^{1}H NMR (500 MHz, $CDCl_3$)

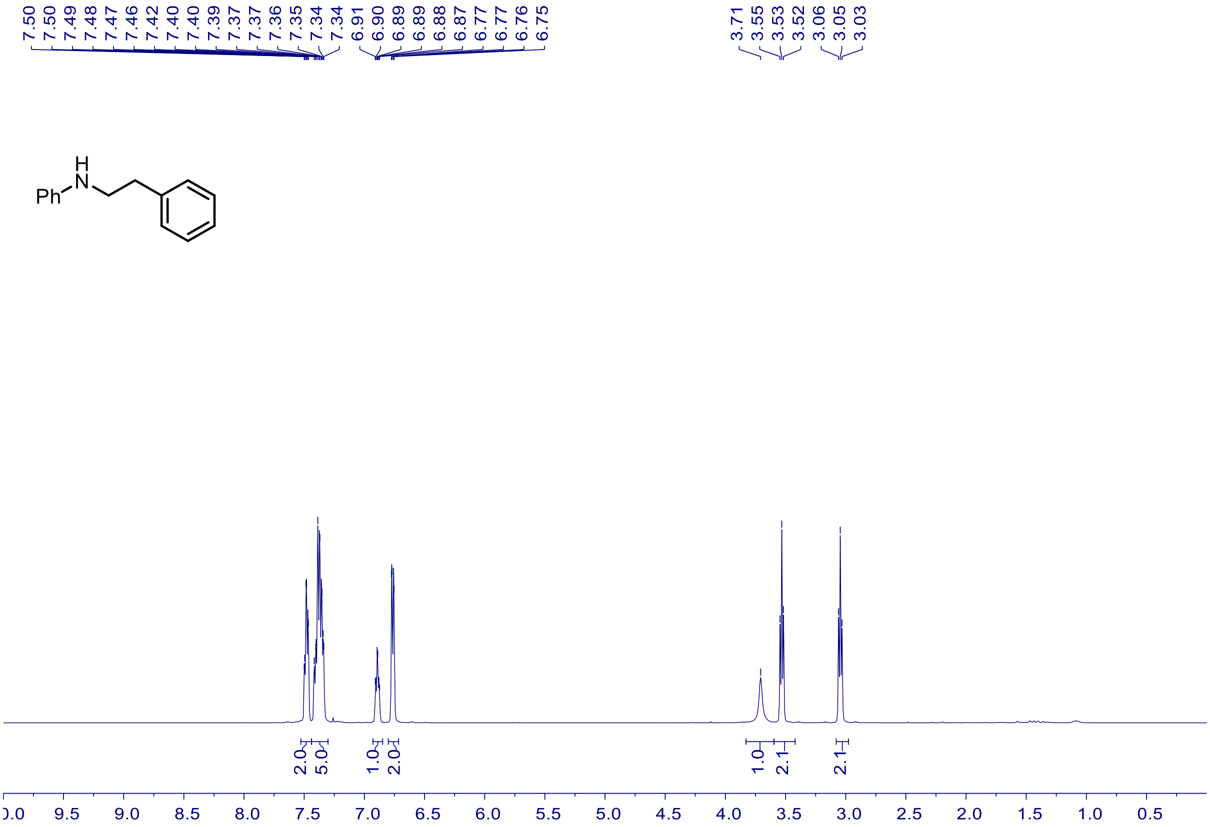

S1 – ^{13}C NMR (126 MHz, $CDCl_3$)

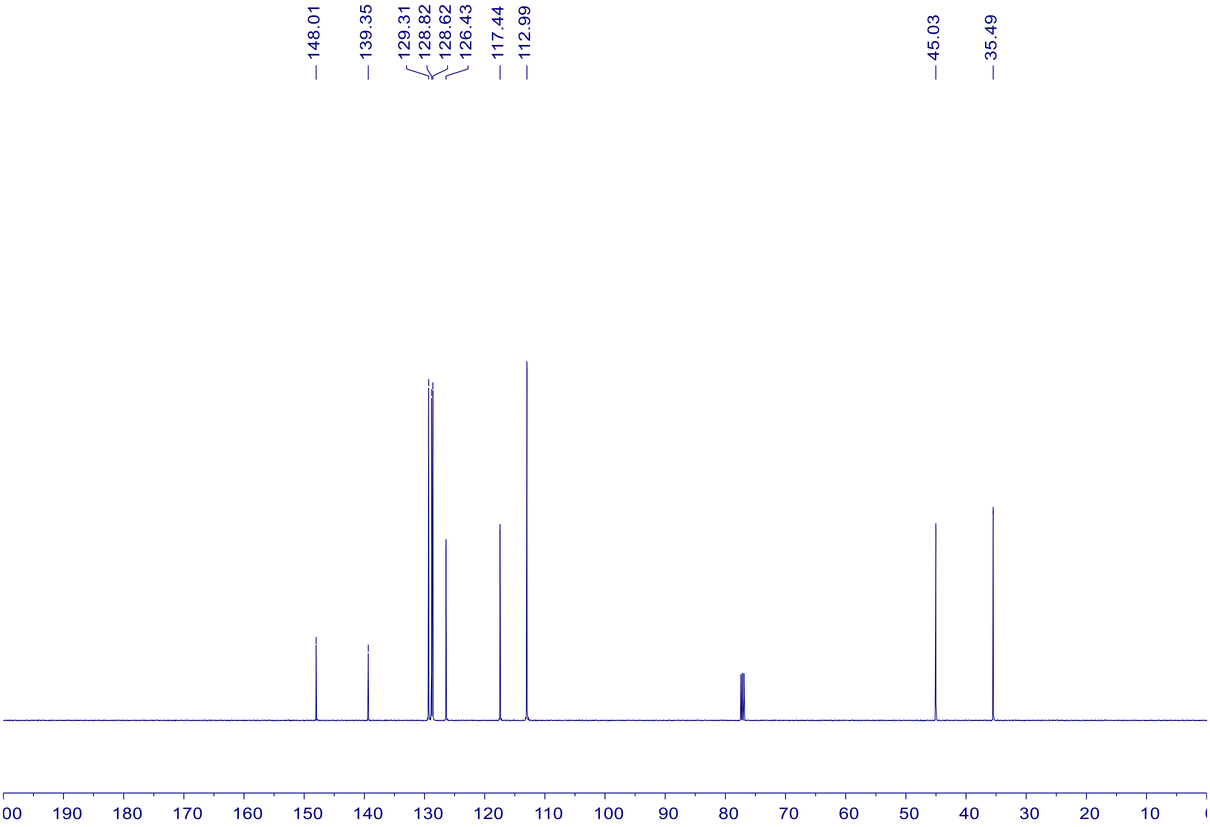

S2 – ^{1}H NMR (400 MHz, $CDCl_3$)

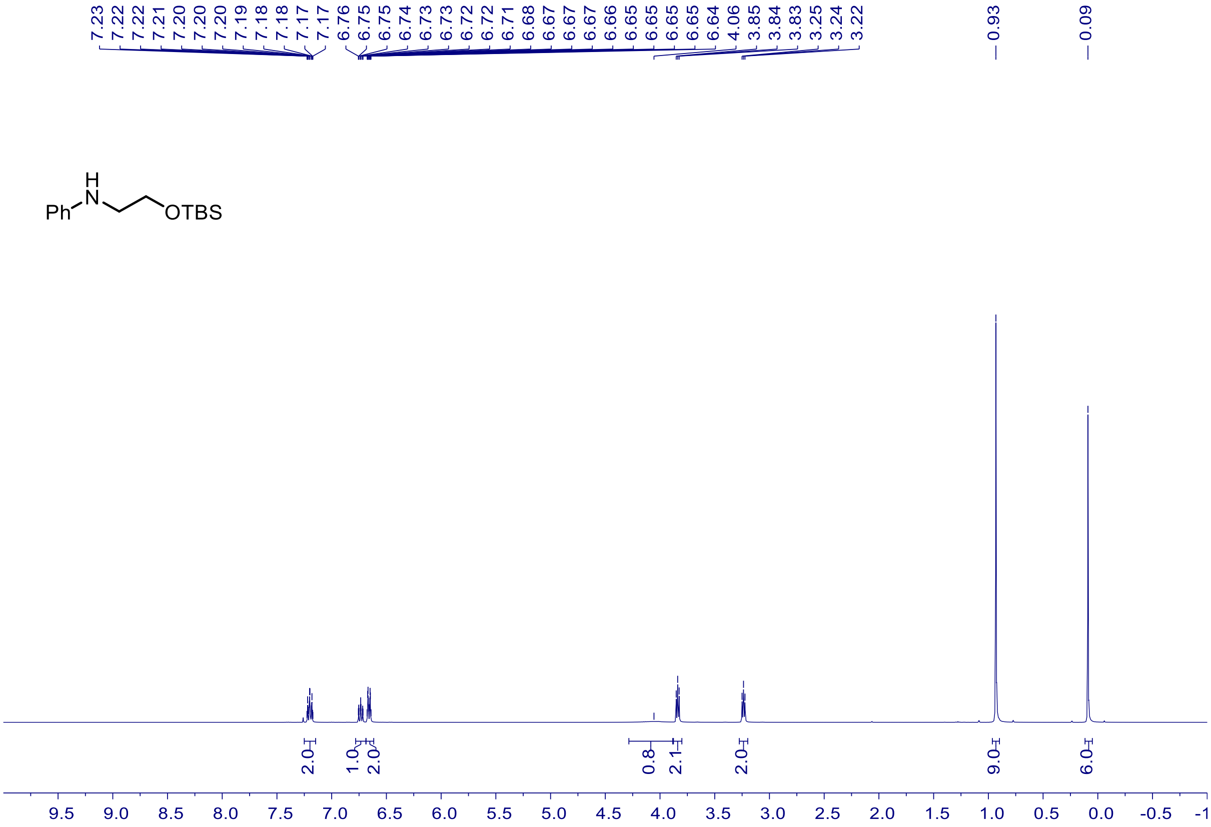

S2 – ^{13}C NMR (101 MHz, $CDCl_3$)

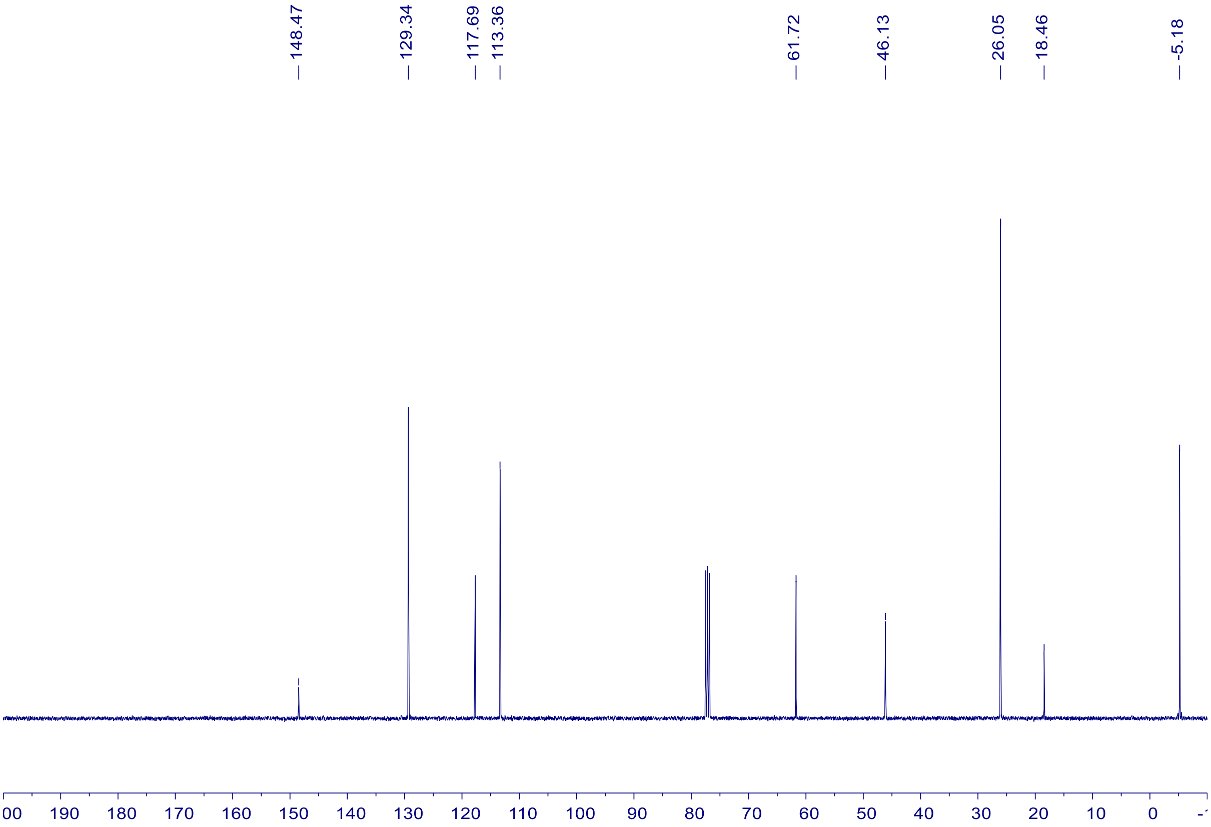

S3 – ^{1}H NMR (500 MHz, $CDCl_3$)

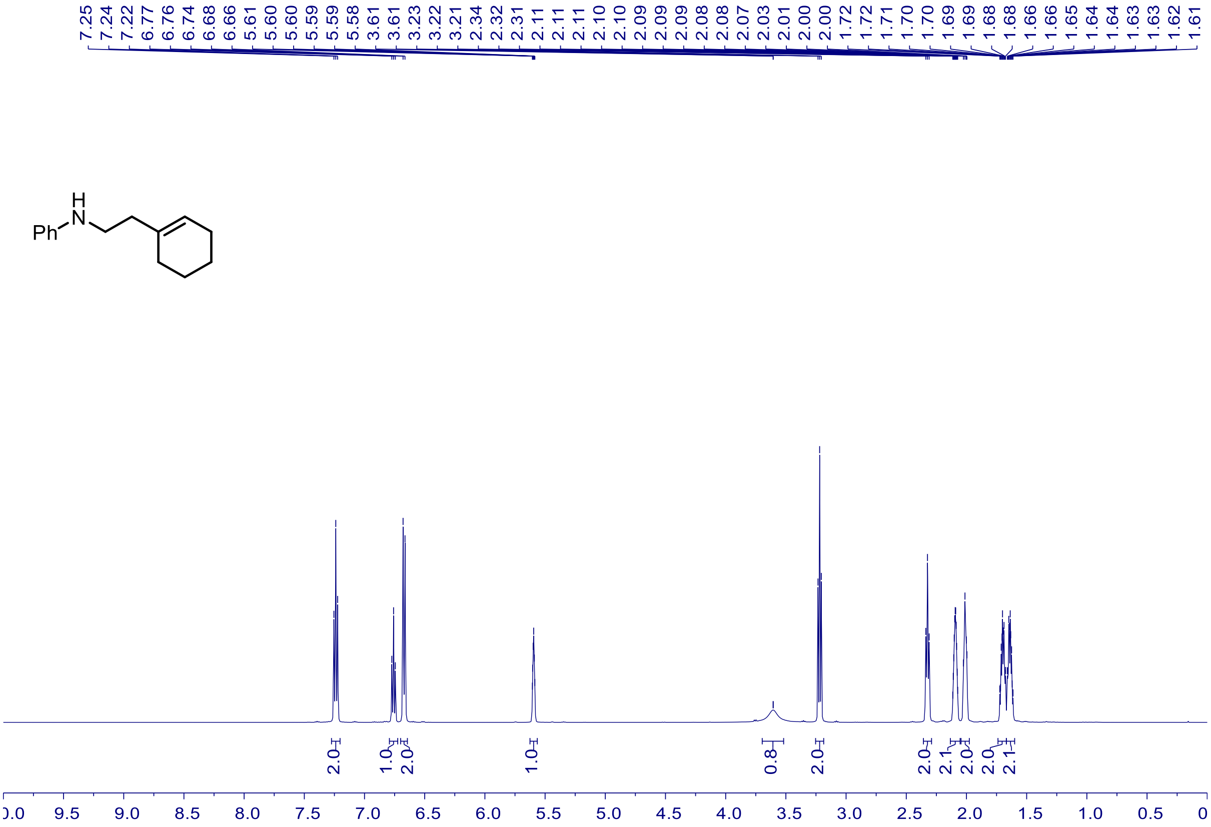

S3 – ^{13}C NMR (126 MHz, $CDCl_3$)

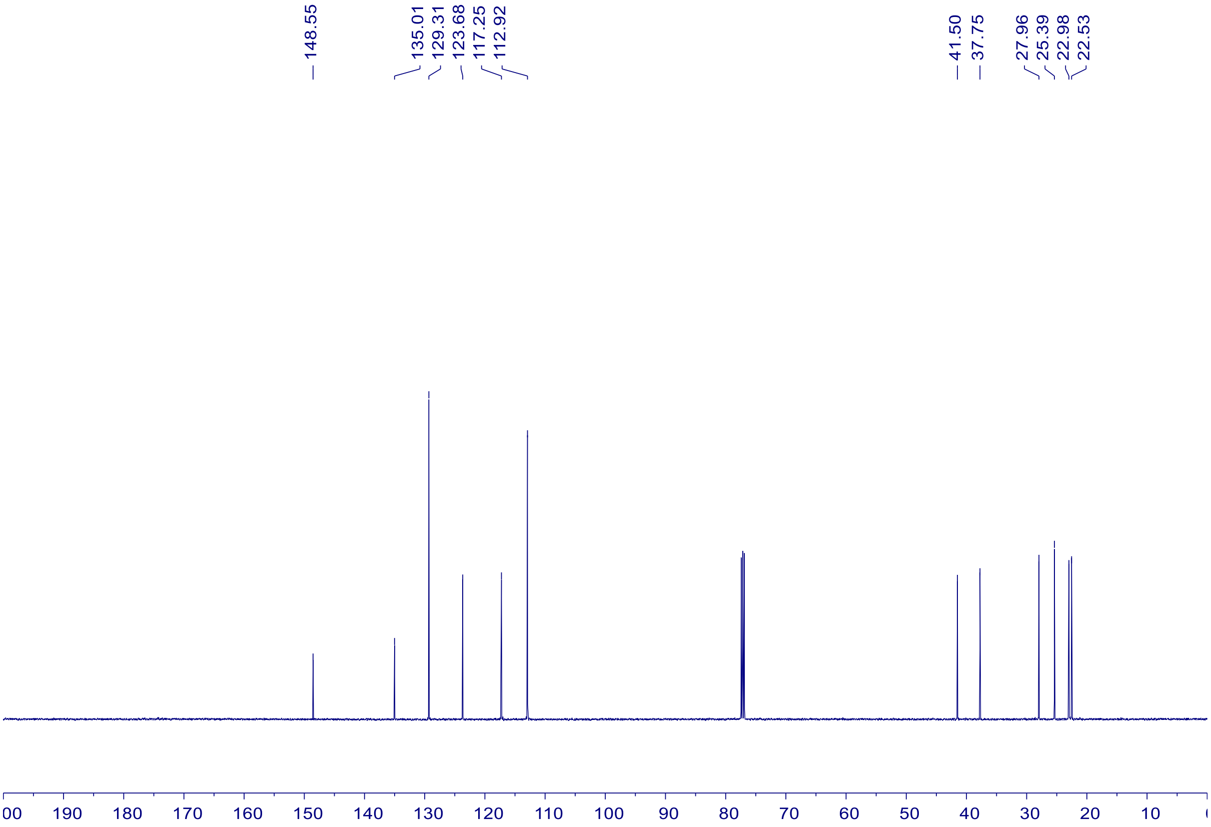

S4 – ^{1}H NMR (500 MHz, $CDCl_3$)

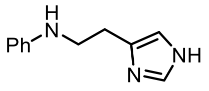

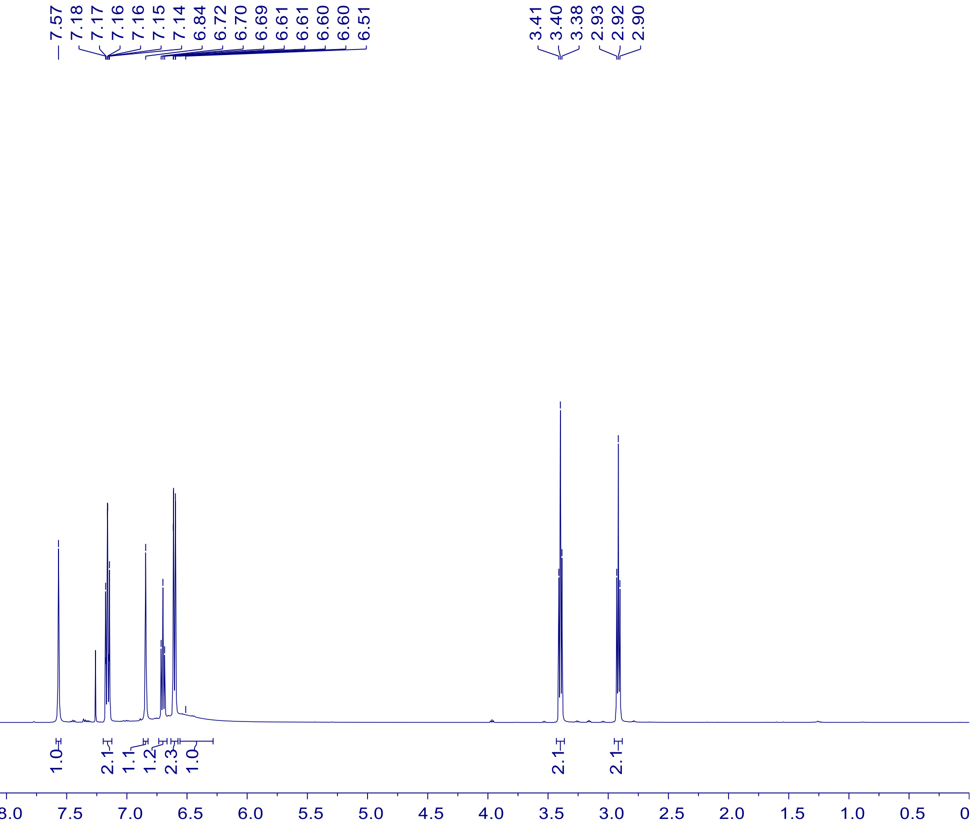

S4 – ^{13}C NMR (126 MHz, $CDCl_3$)

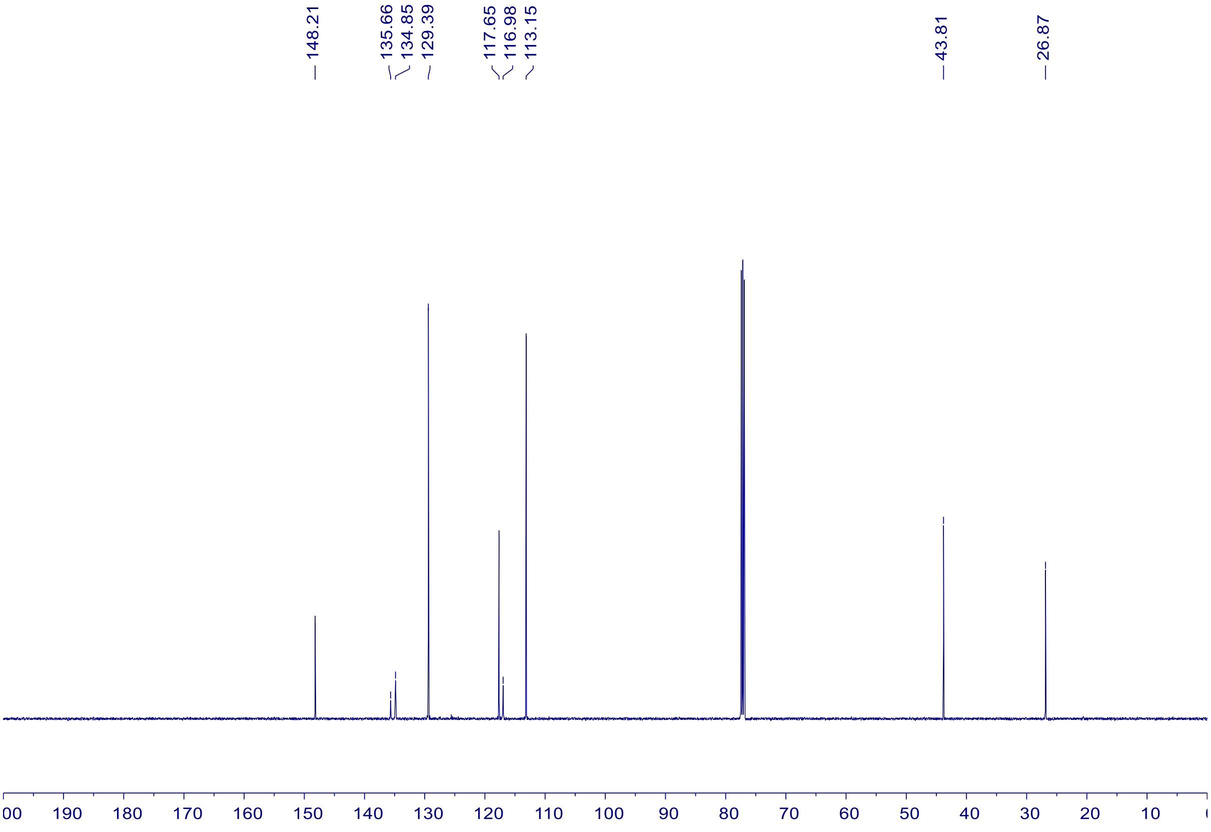

S5 – ^{1}H NMR (500 MHz, $CDCl_3$)

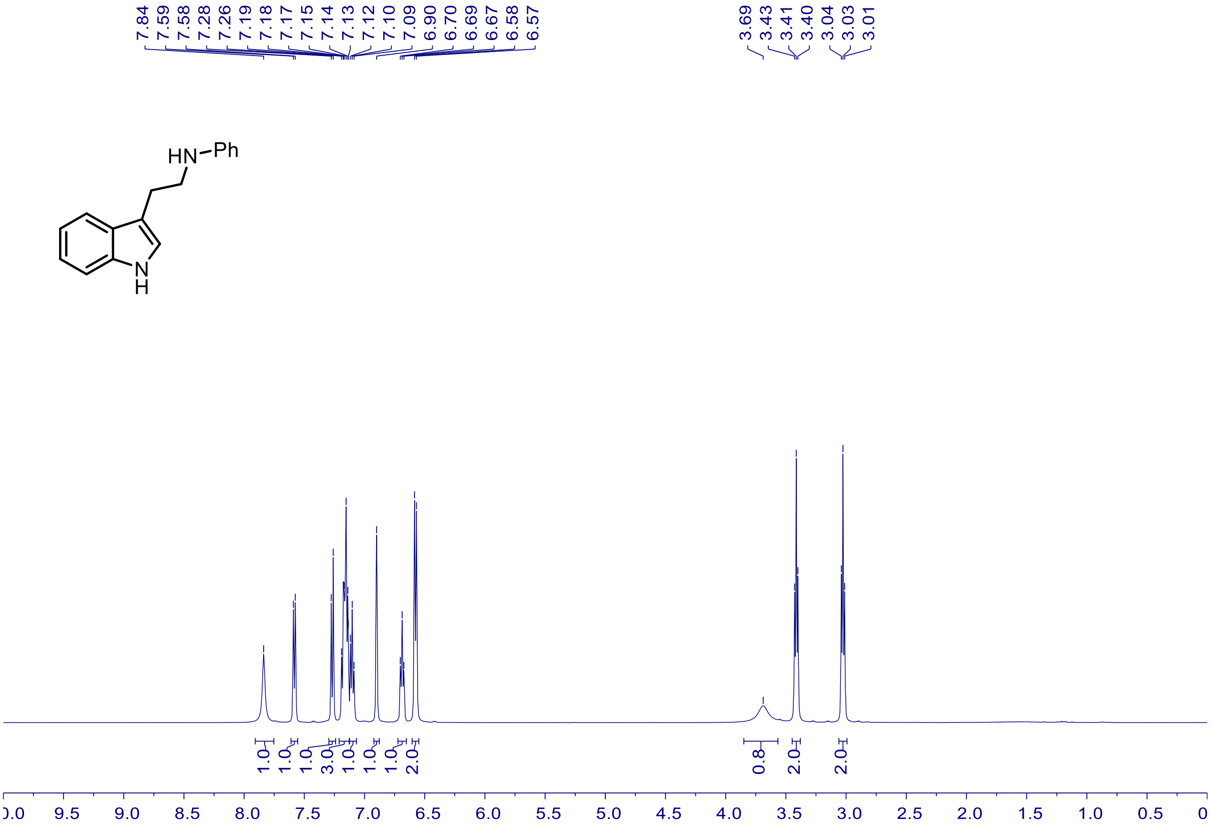

S5 – ^{13}C NMR (126 MHz, $CDCl_3$)

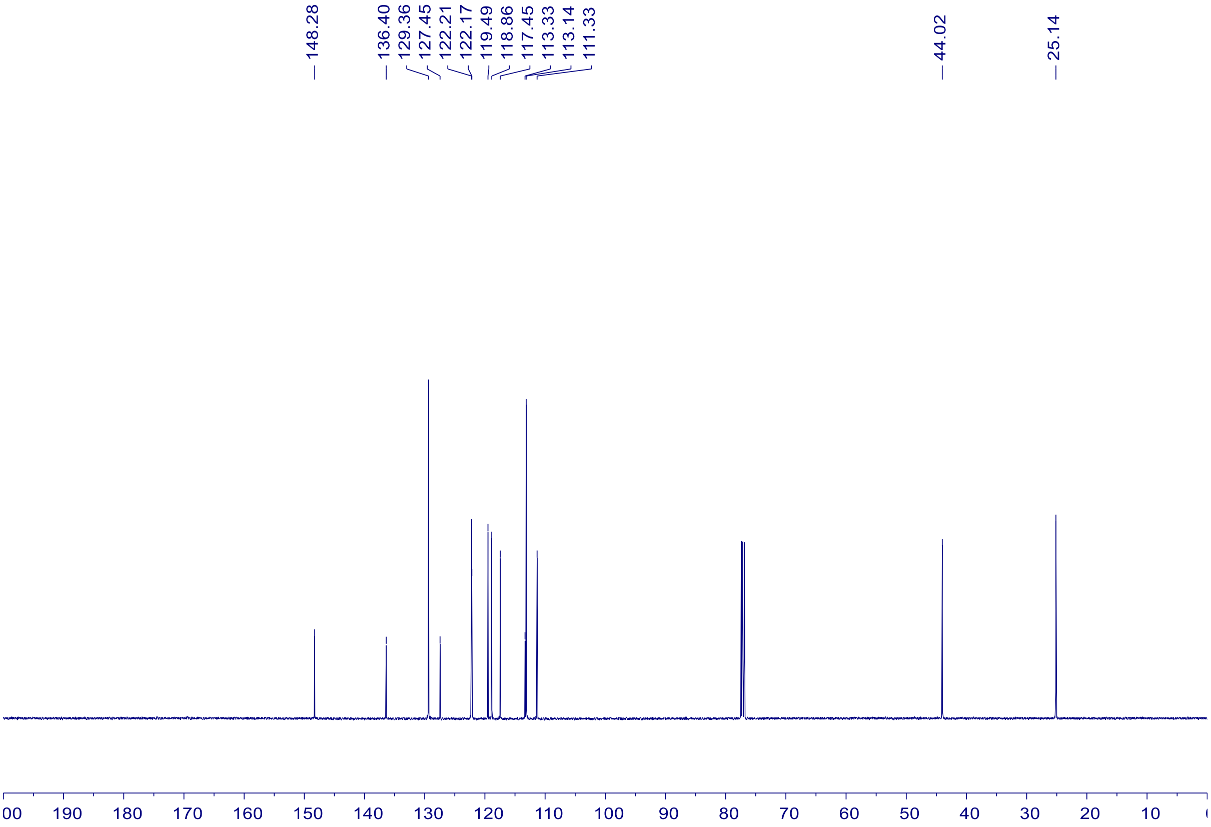

S6 – ^{1}H NMR (500 MHz, $CDCl_3$)

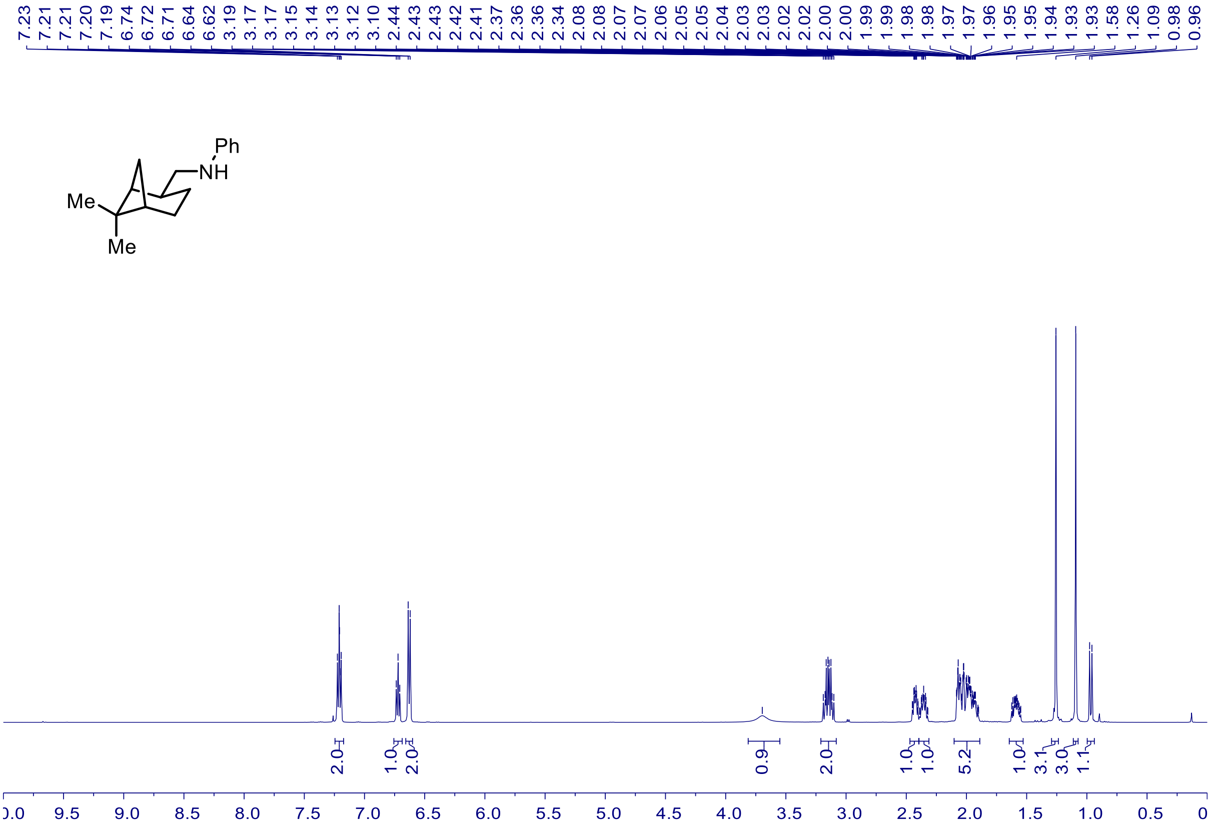

S6 – ^{13}C NMR (126 MHz, $CDCl_3$)

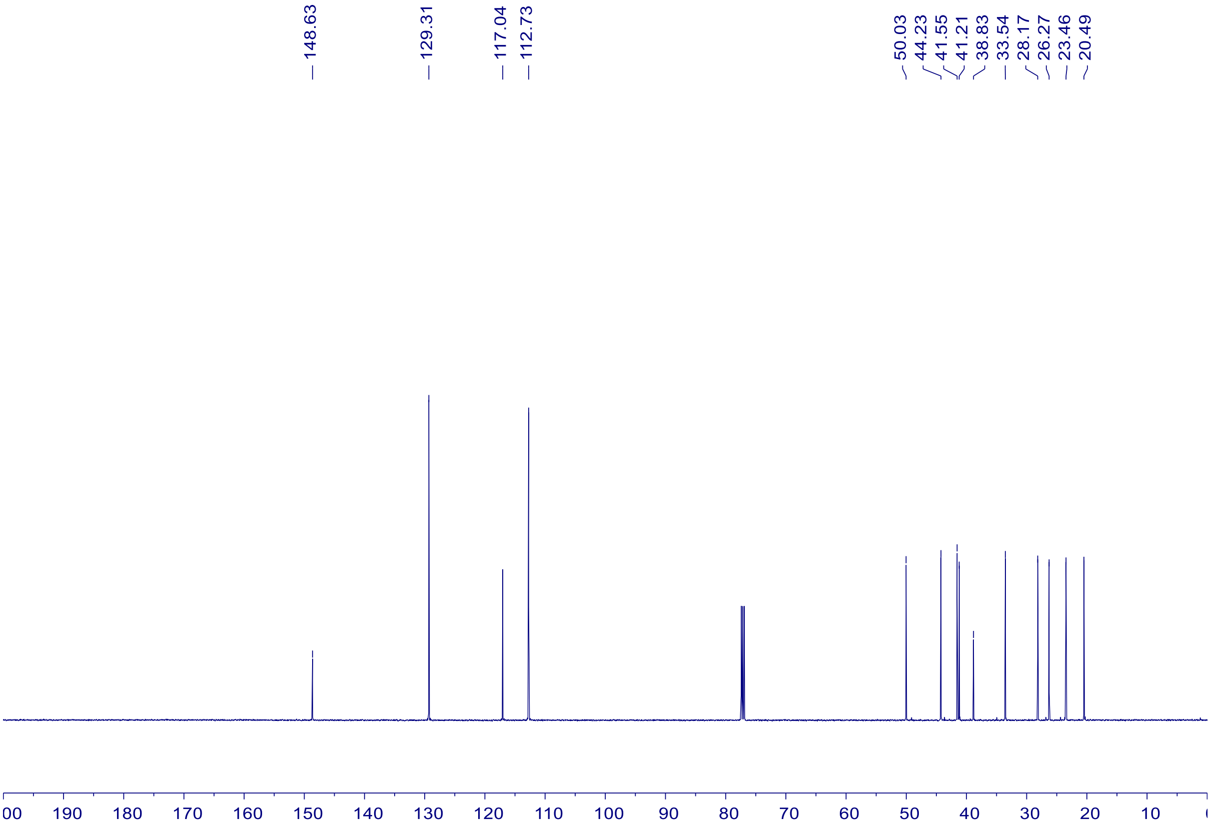

5 – ^{1}H NMR (400 MHz, $CDCl_3$)

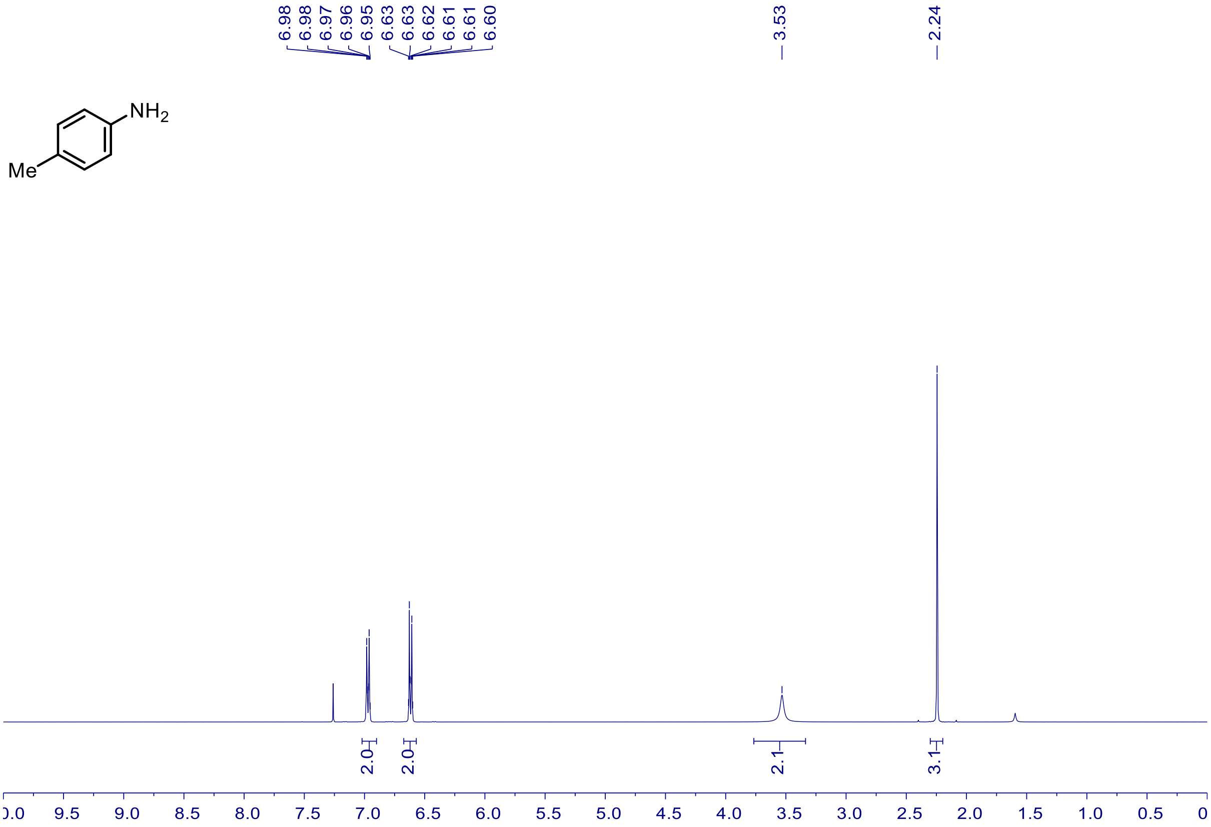

5 – ^{13}C NMR (101 MHz, $CDCl_3$)

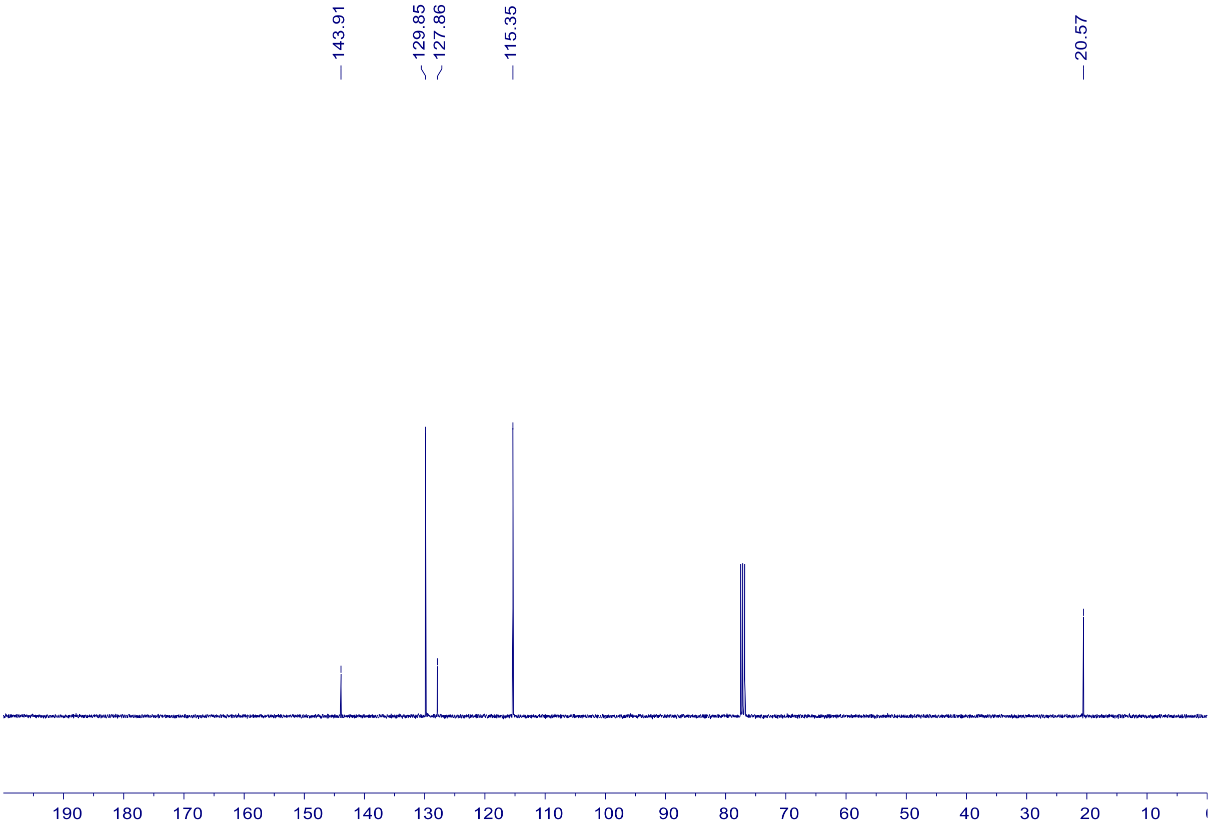

6 – ^{1}H NMR (400 MHz, $CDCl_3$)

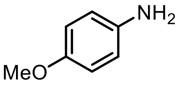

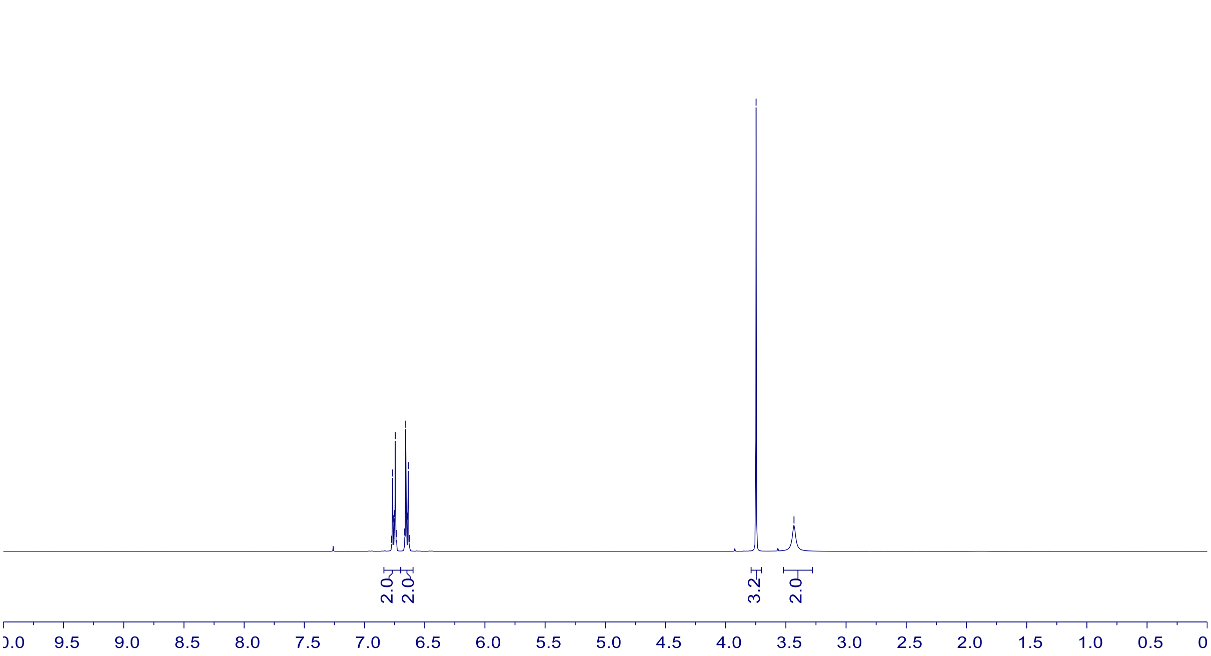

6 – ^{13}C NMR (101 MHz, $CDCl_3$)

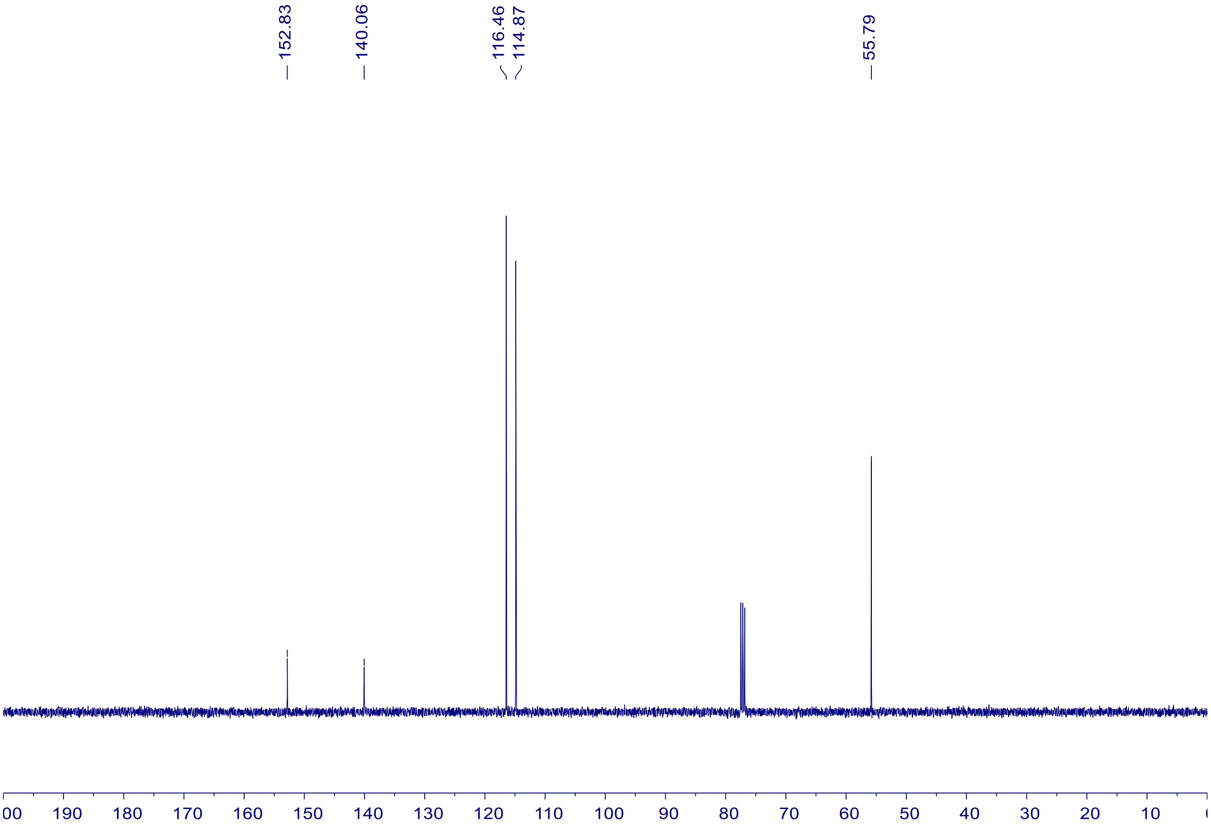

7 – ^{1}H NMR (400 MHz, $CDCl_3$)

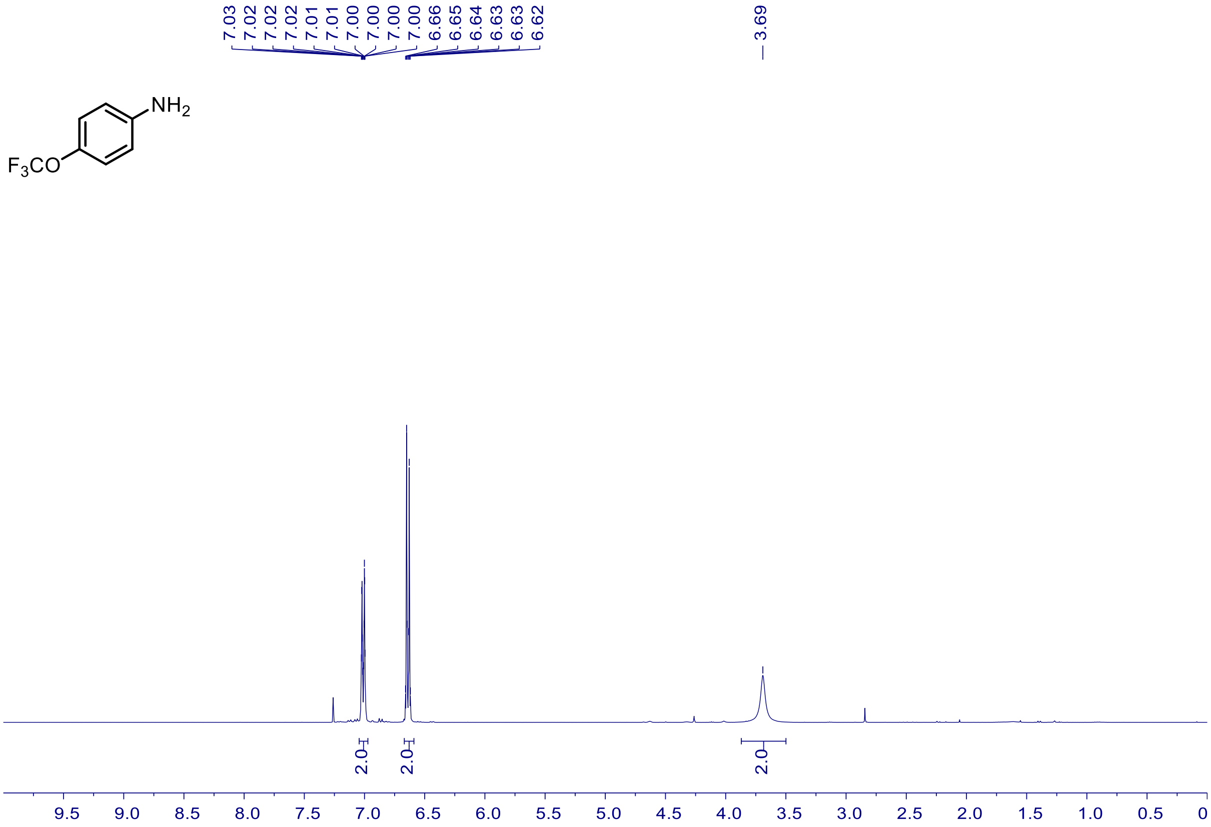

7 – ^{13}C NMR (126 MHz, $CDCl_3$)

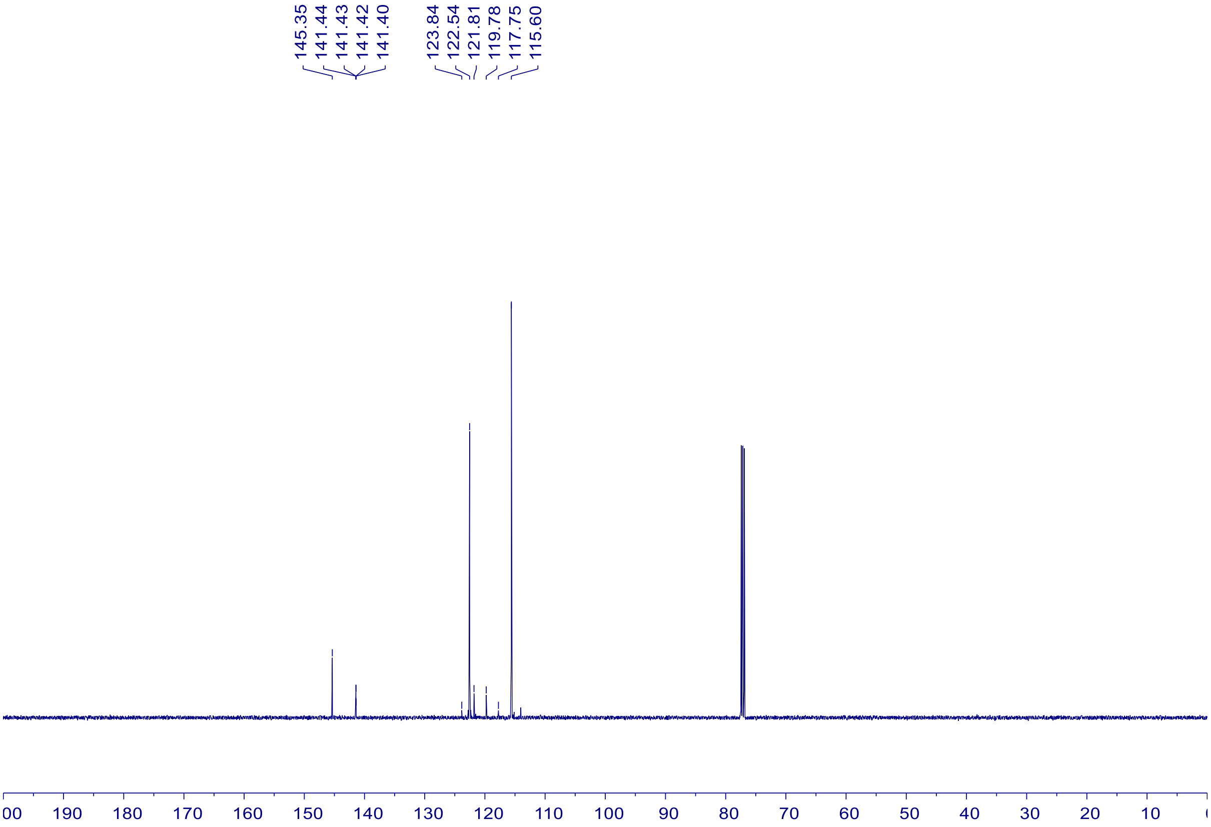

7 – ^{19}F NMR (376 MHz, $CDCl_3$)

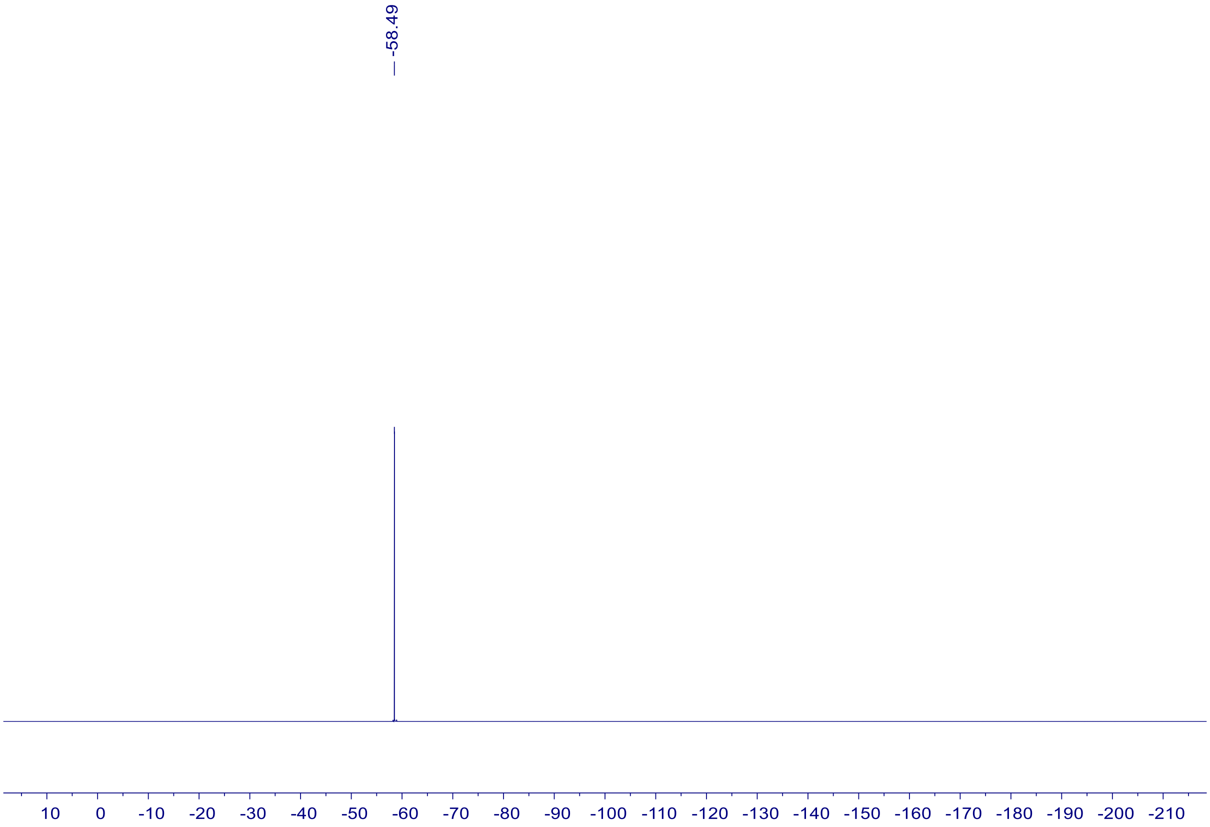

8 – ^{1}H NMR (400 MHz, $CDCl_3$)

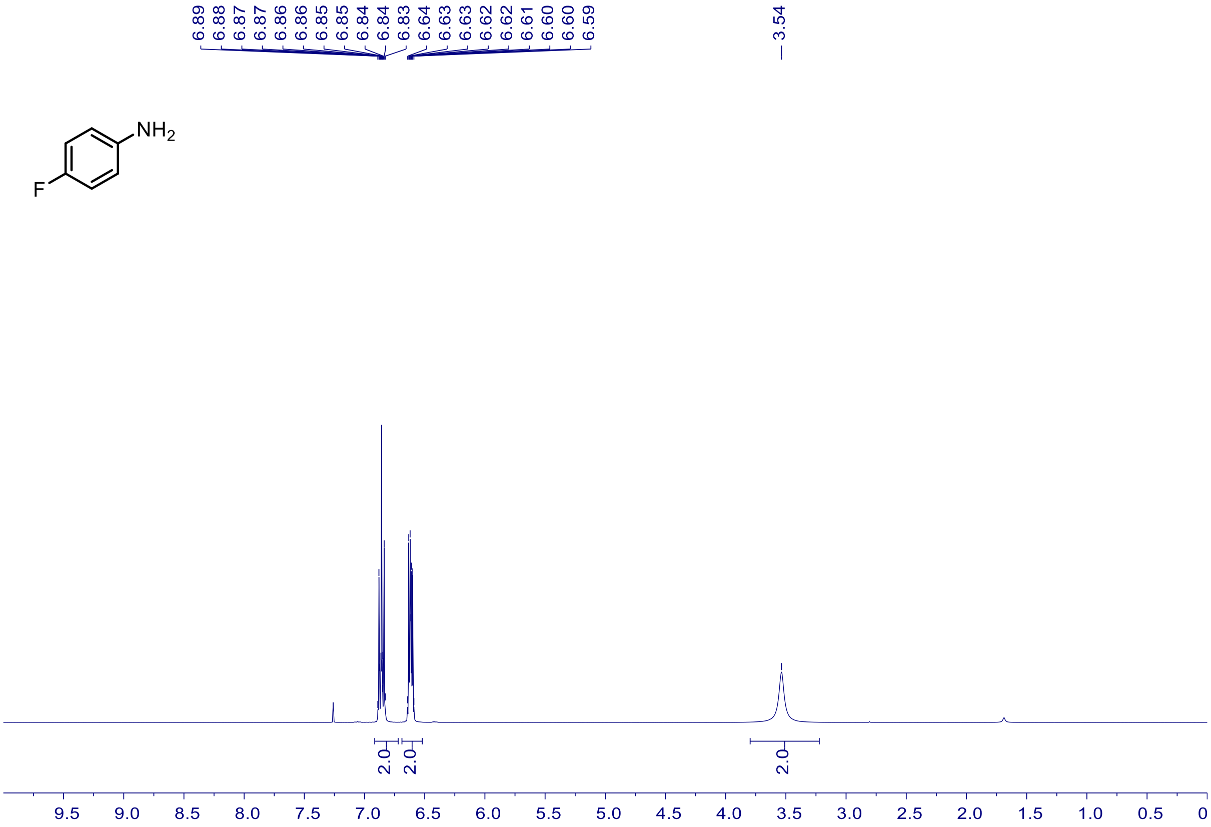

8 – ^{13}C NMR (126 MHz, $CDCl_3$)

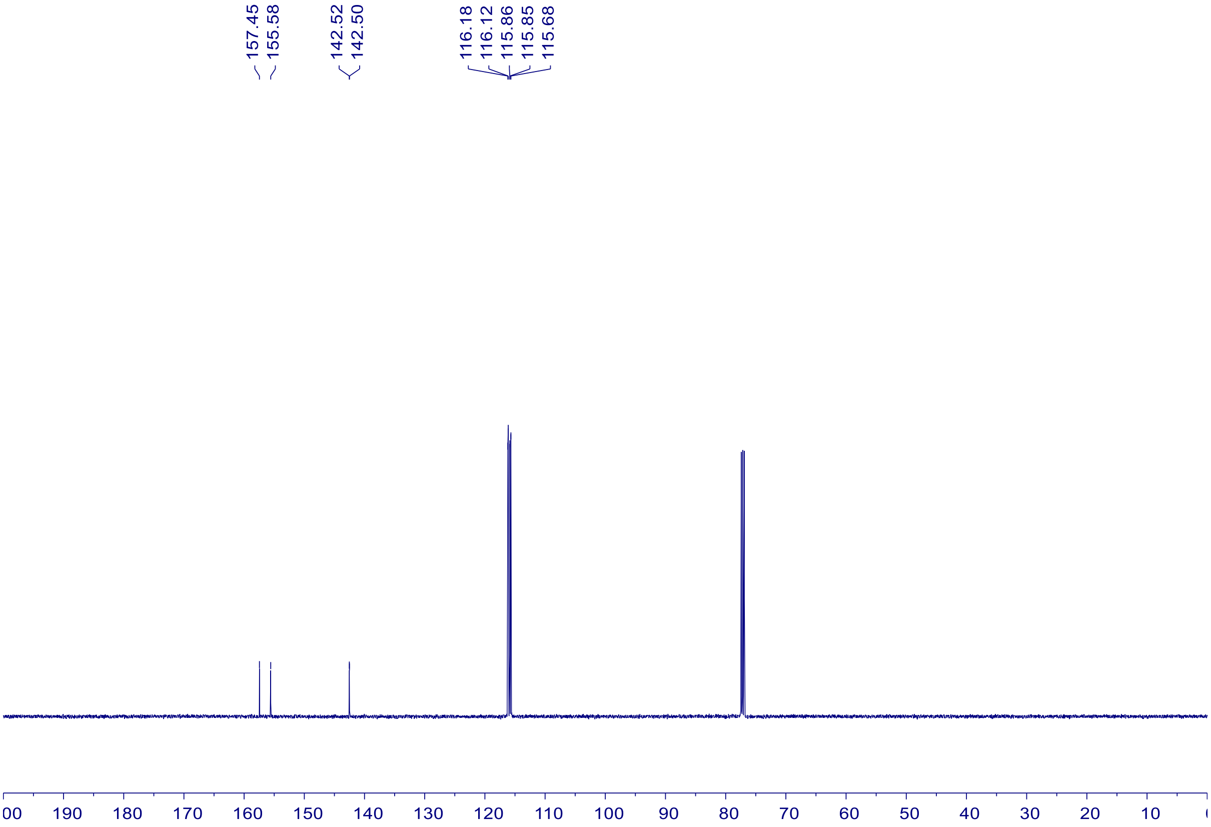

8 – ^{19}F NMR (376 MHz, $CDCl_3$)

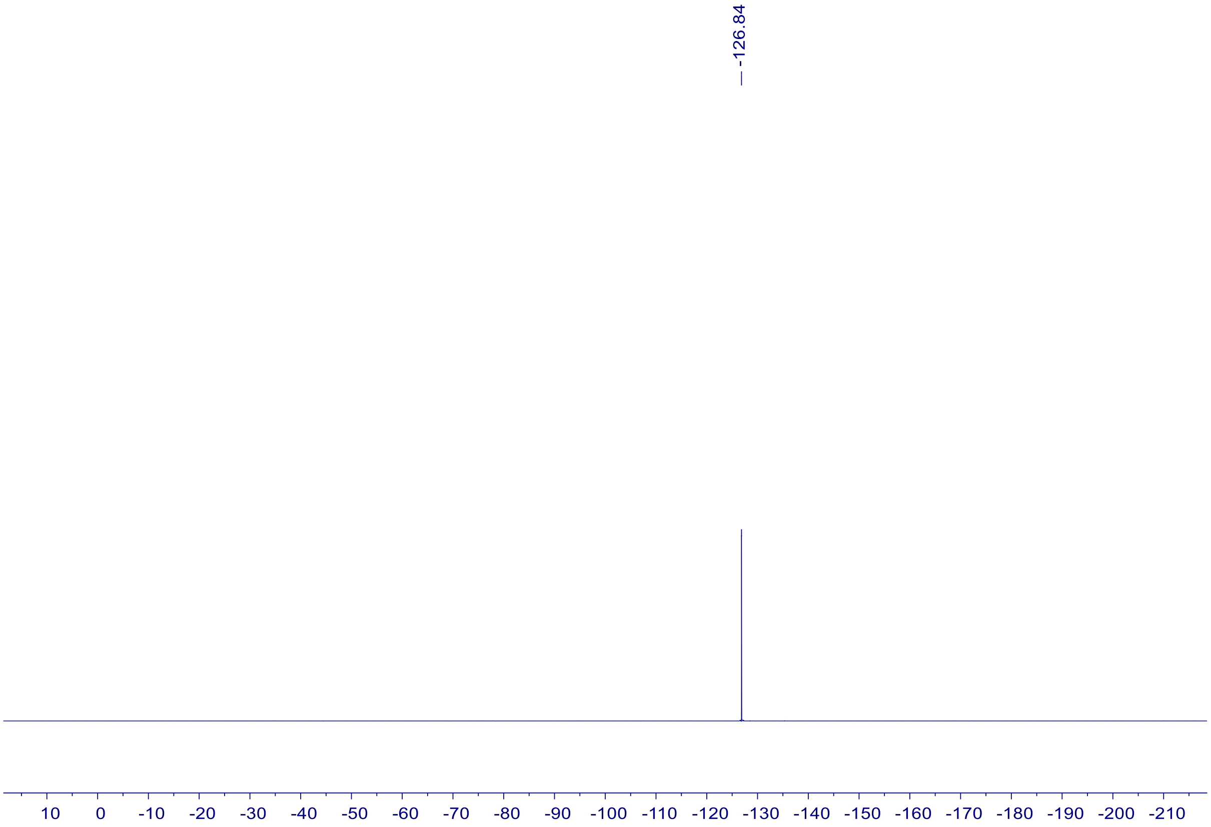

9 – ^{1}H NMR (500 MHz, $CDCl_3$)

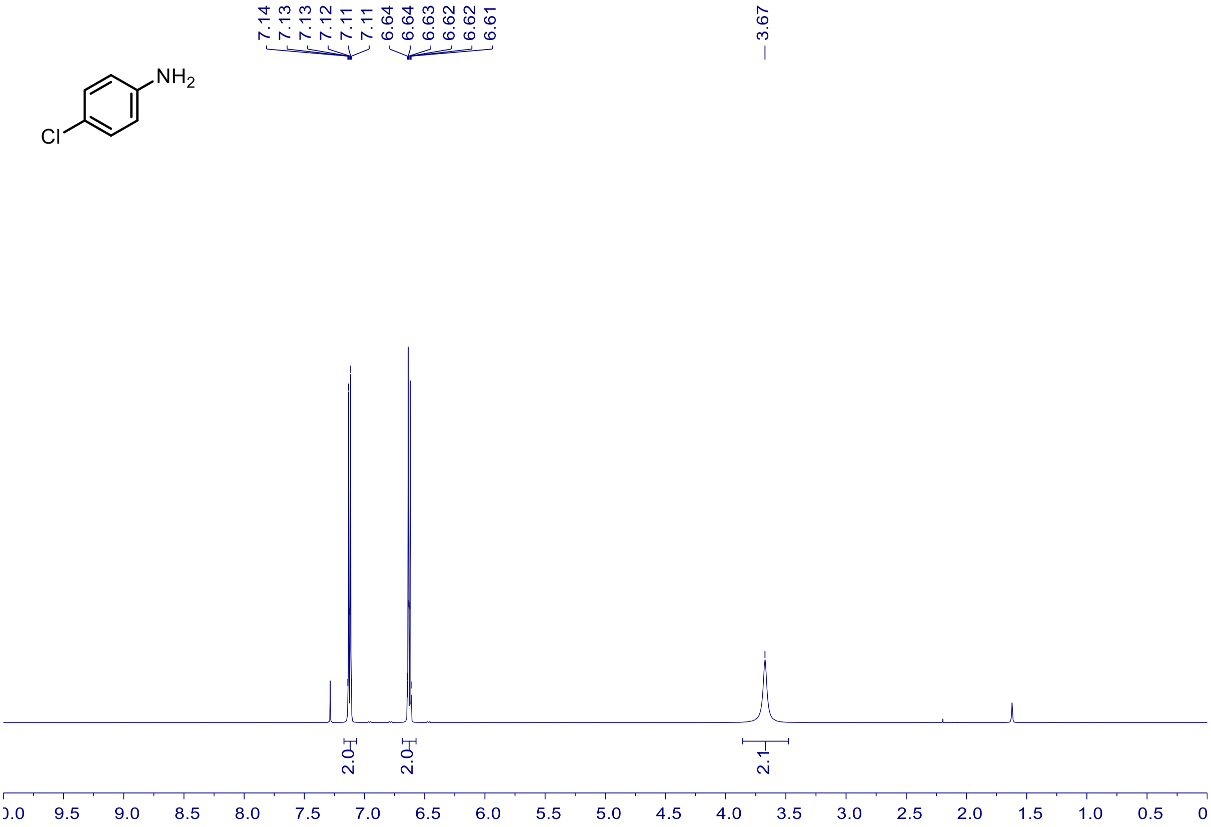

9 – ^{13}C NMR (101 MHz, $CDCl_3$)

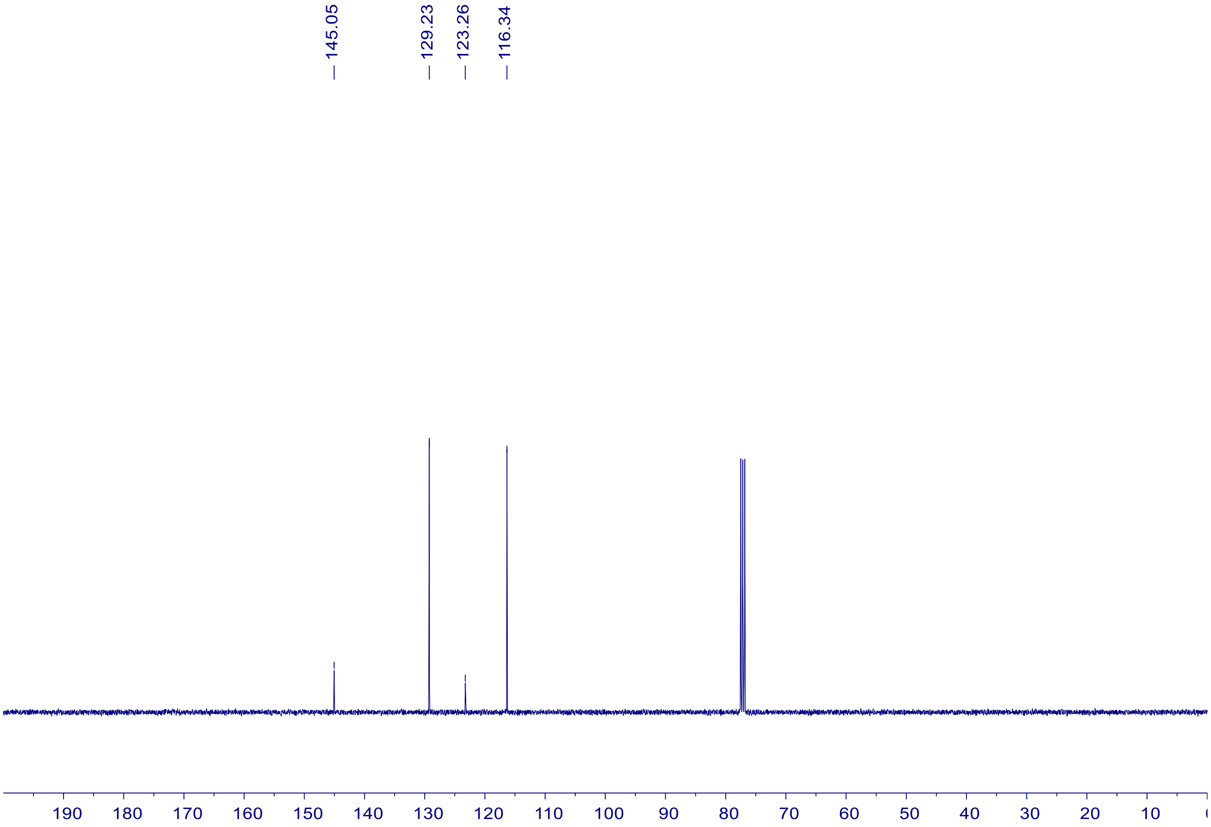

10 – ^{1}H NMR (500 MHz, $CDCl_3$)

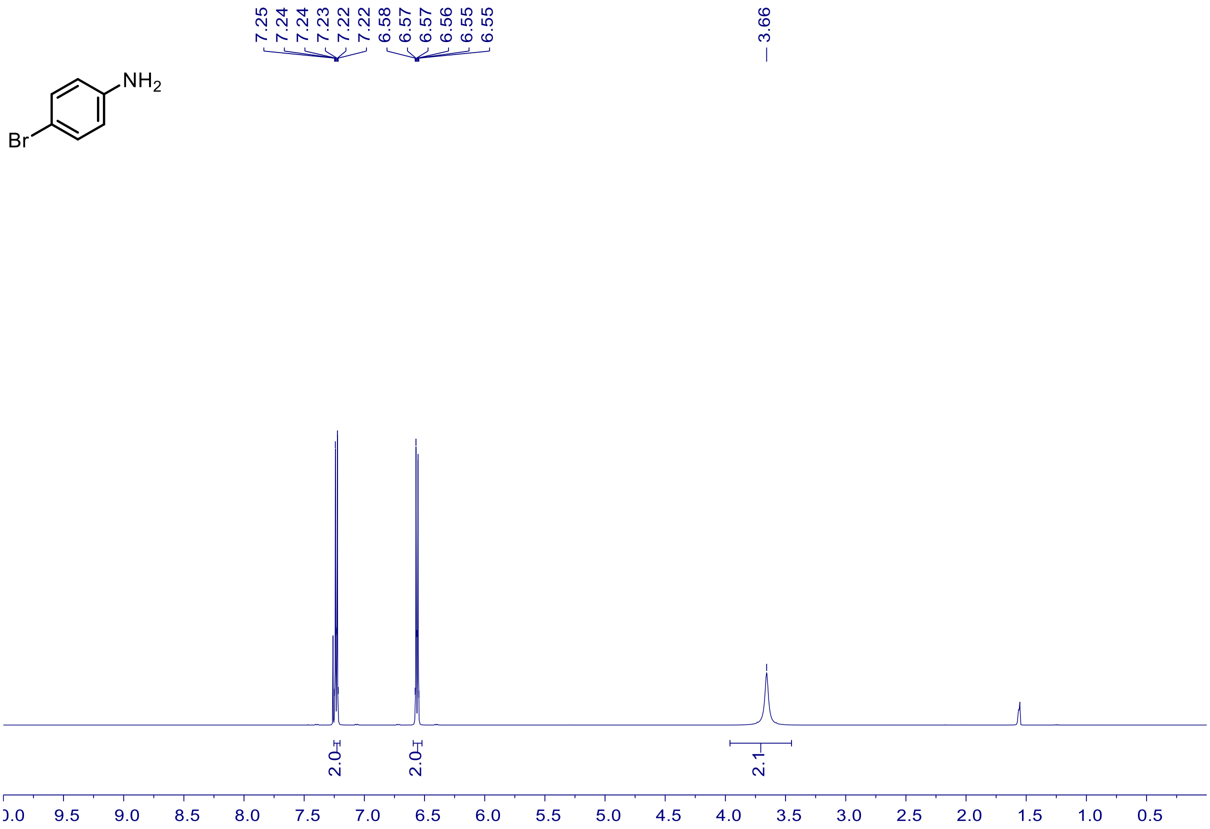

10 – ^{13}C NMR (126 MHz, $CDCl_3$)

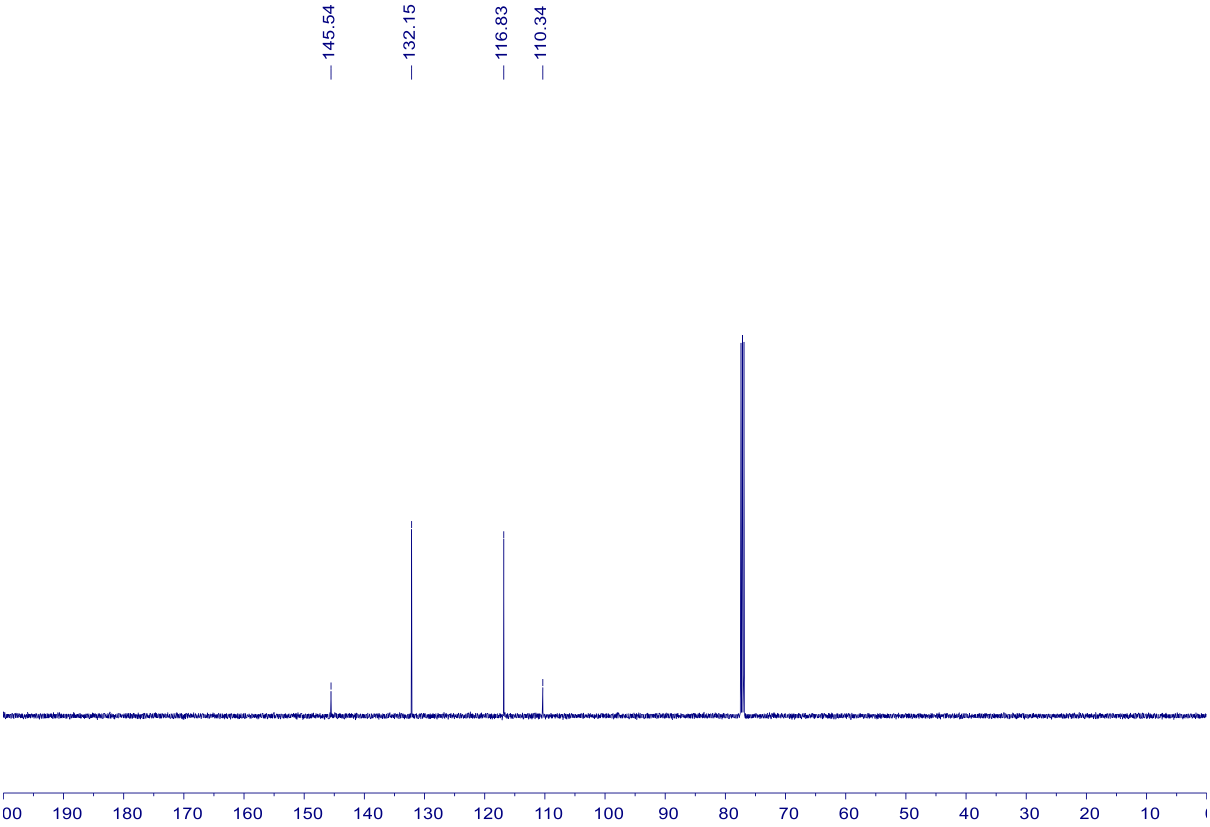

11 – ^{1}H NMR (500 MHz, $CDCl_3$)

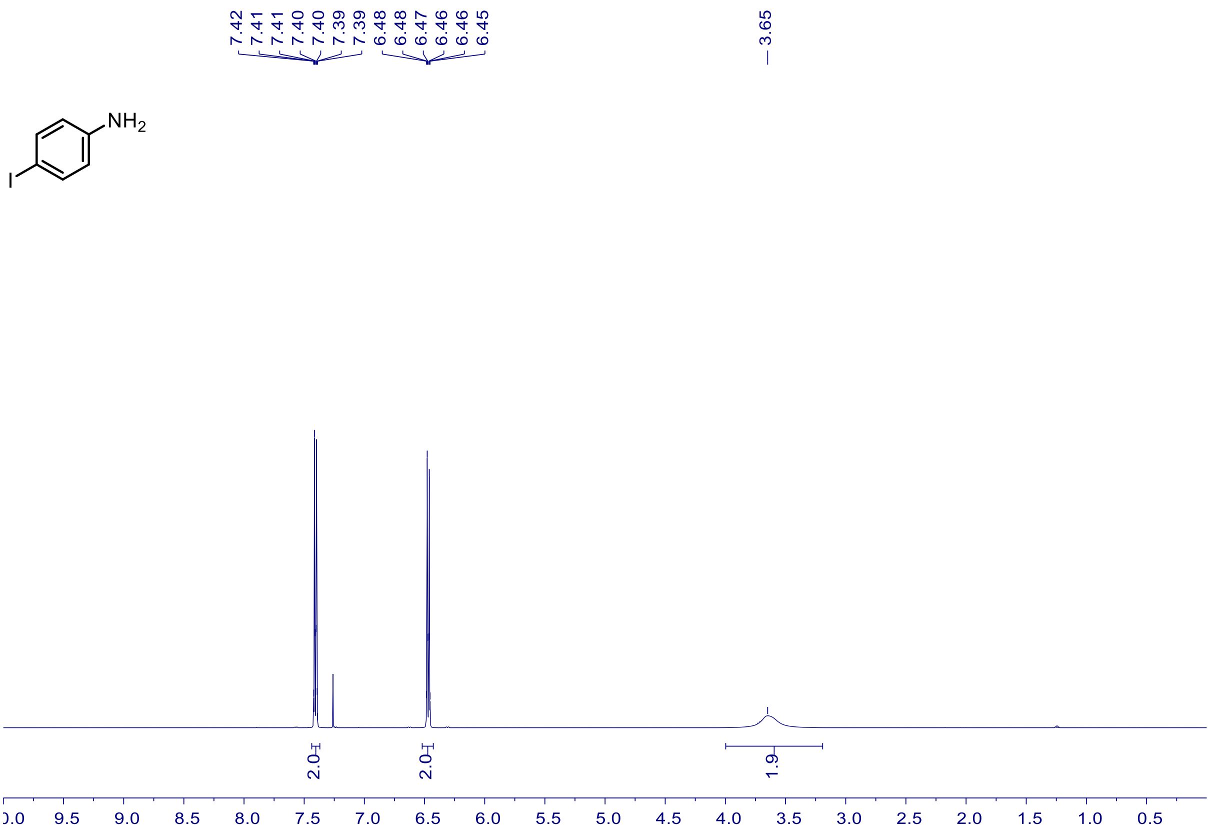

11 – ^{13}C NMR (126 MHz, $CDCl_3$)

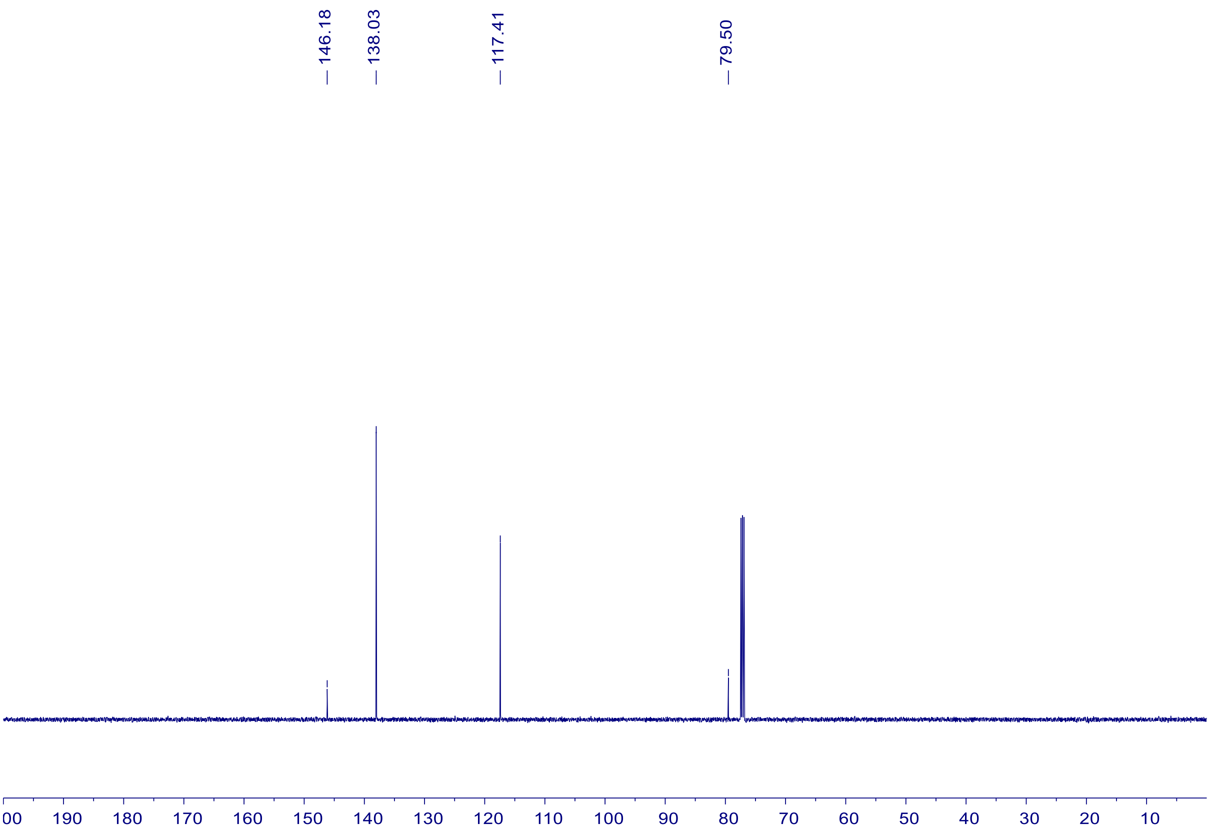

12 – ^{1}H NMR (500 MHz, $CDCl_3$)

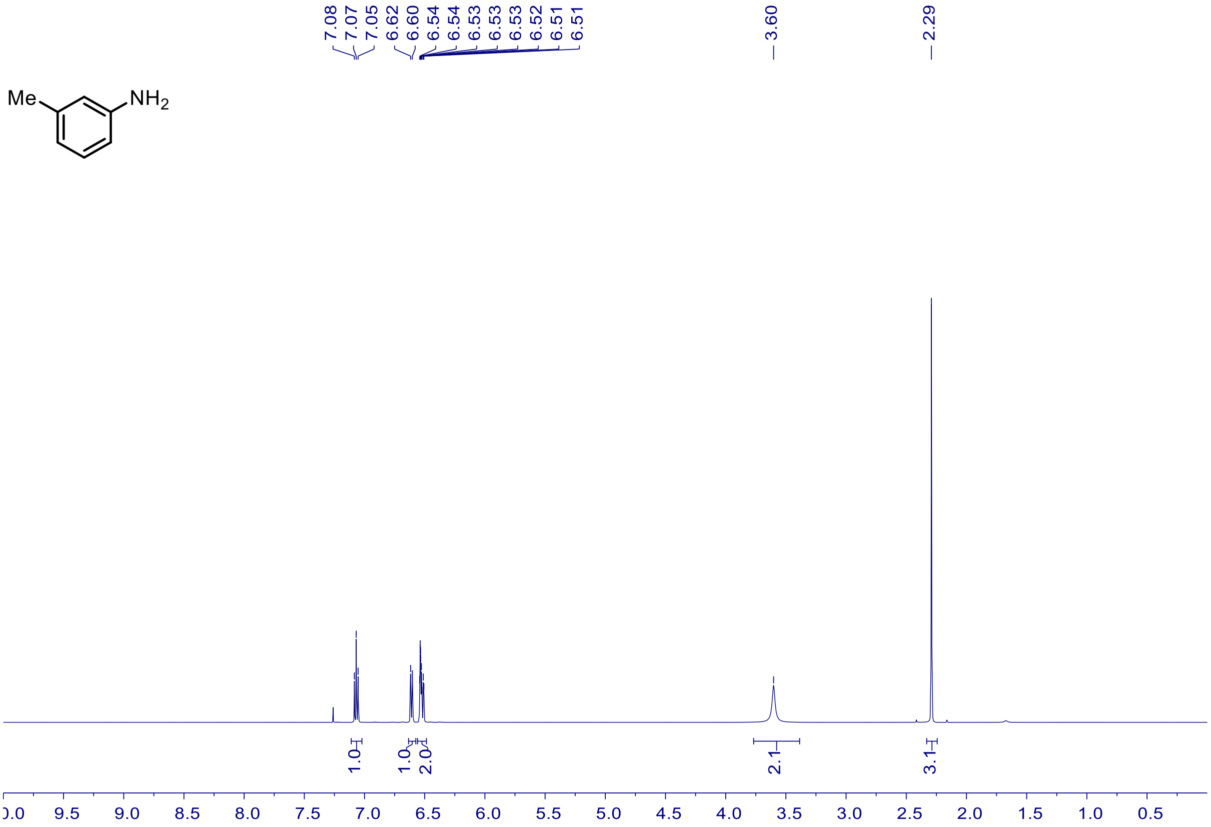

12 – ^{13}C NMR (126 MHz, $CDCl_3$)

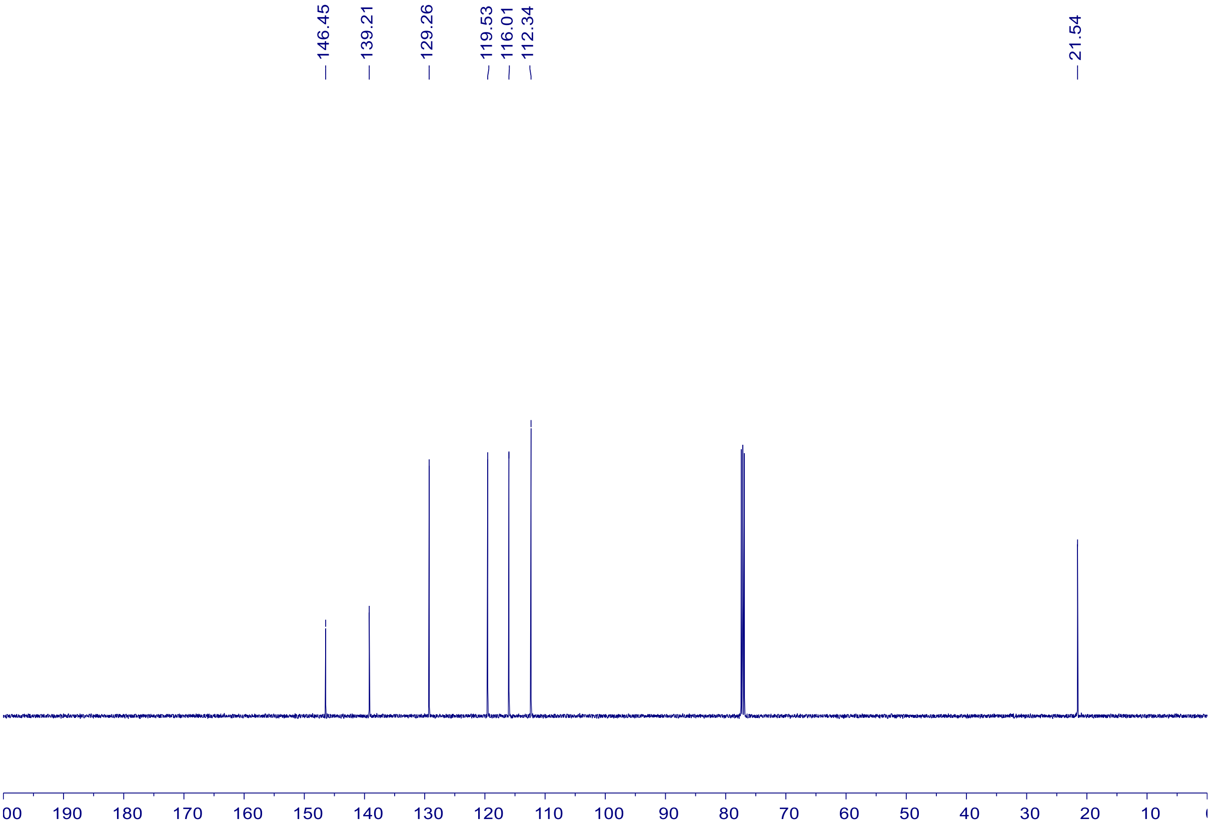

13 – ^{1}H NMR (500 MHz, $CDCl_3$)

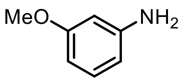

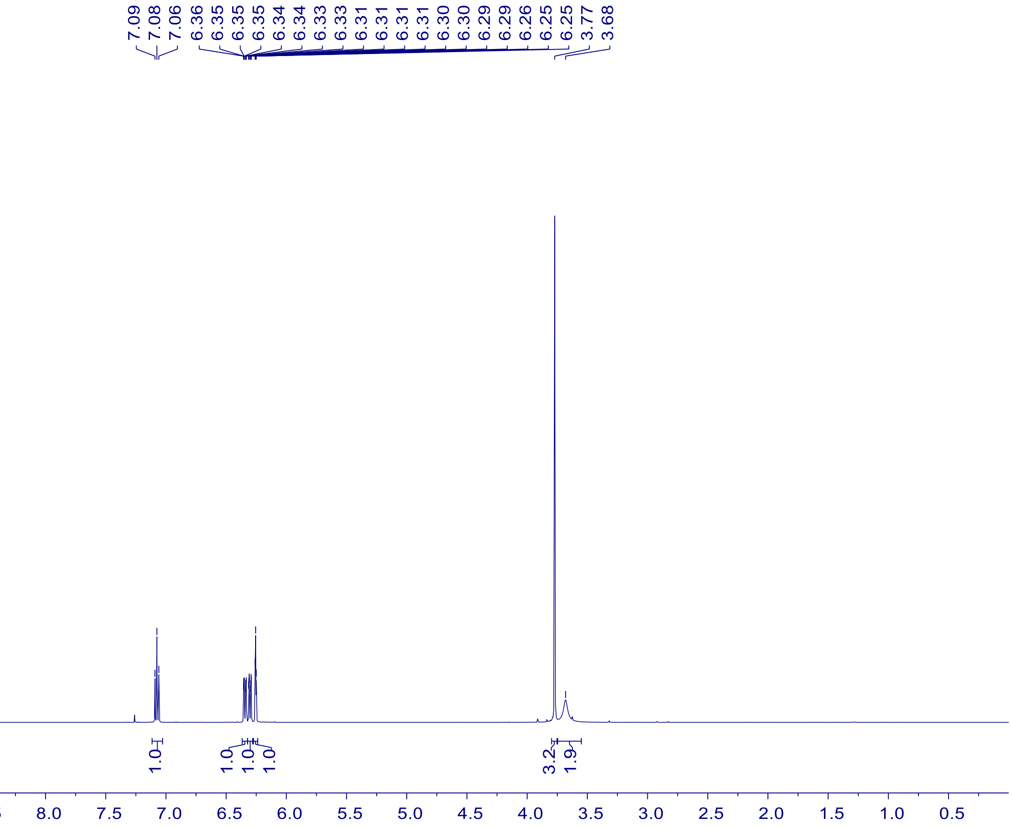

13 – ^{13}C NMR (126 MHz, $CDCl_3$)

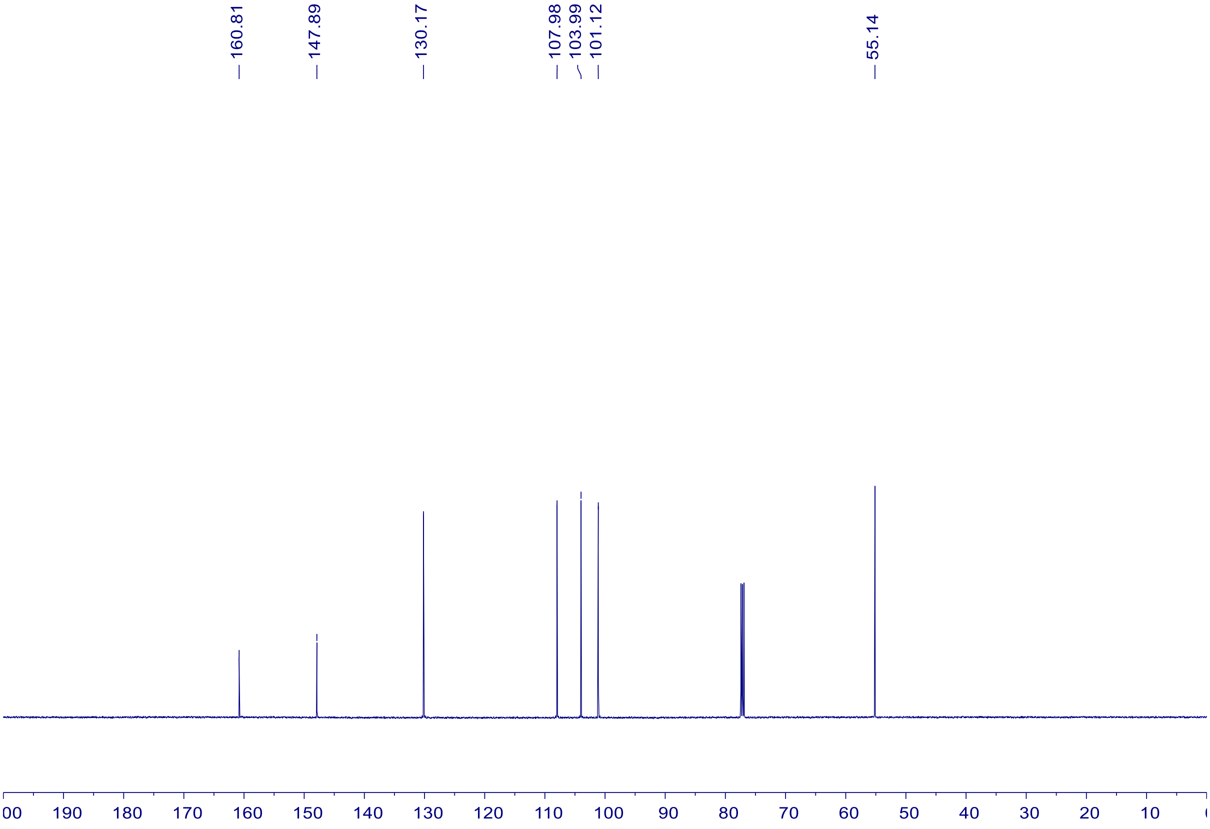

14 – ^{1}H NMR (500 MHz, $CDCl_3$)

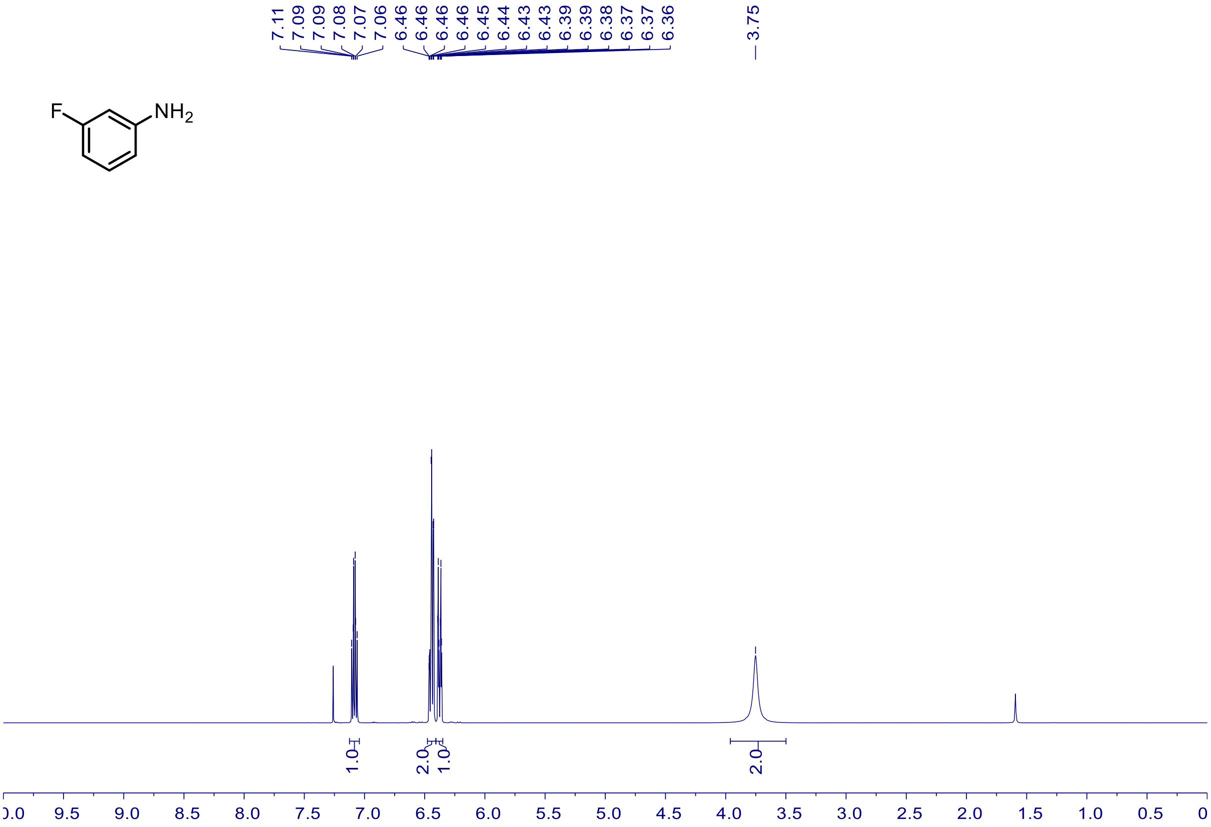

14 – ^{13}C NMR (126 MHz, $CDCl_3$)

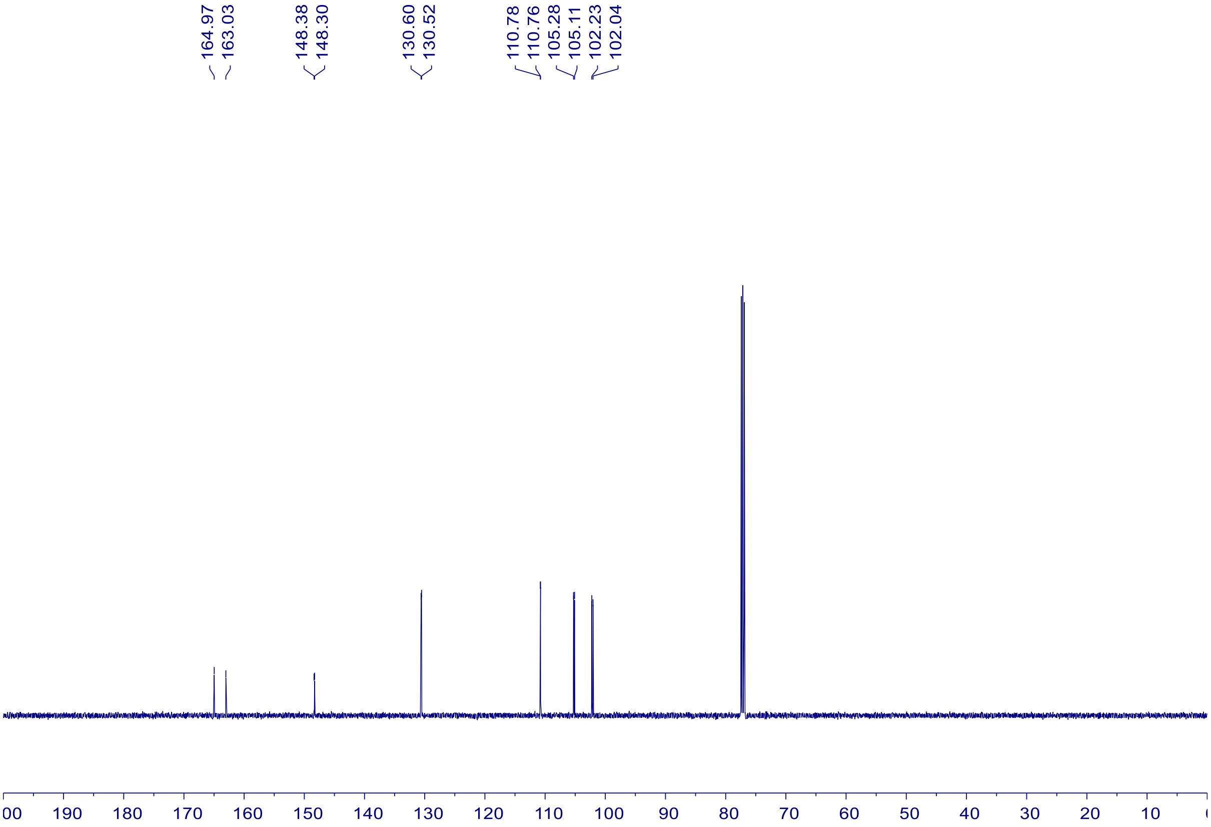

14 – ^{19}F NMR (376 MHz, $CDCl_3$)

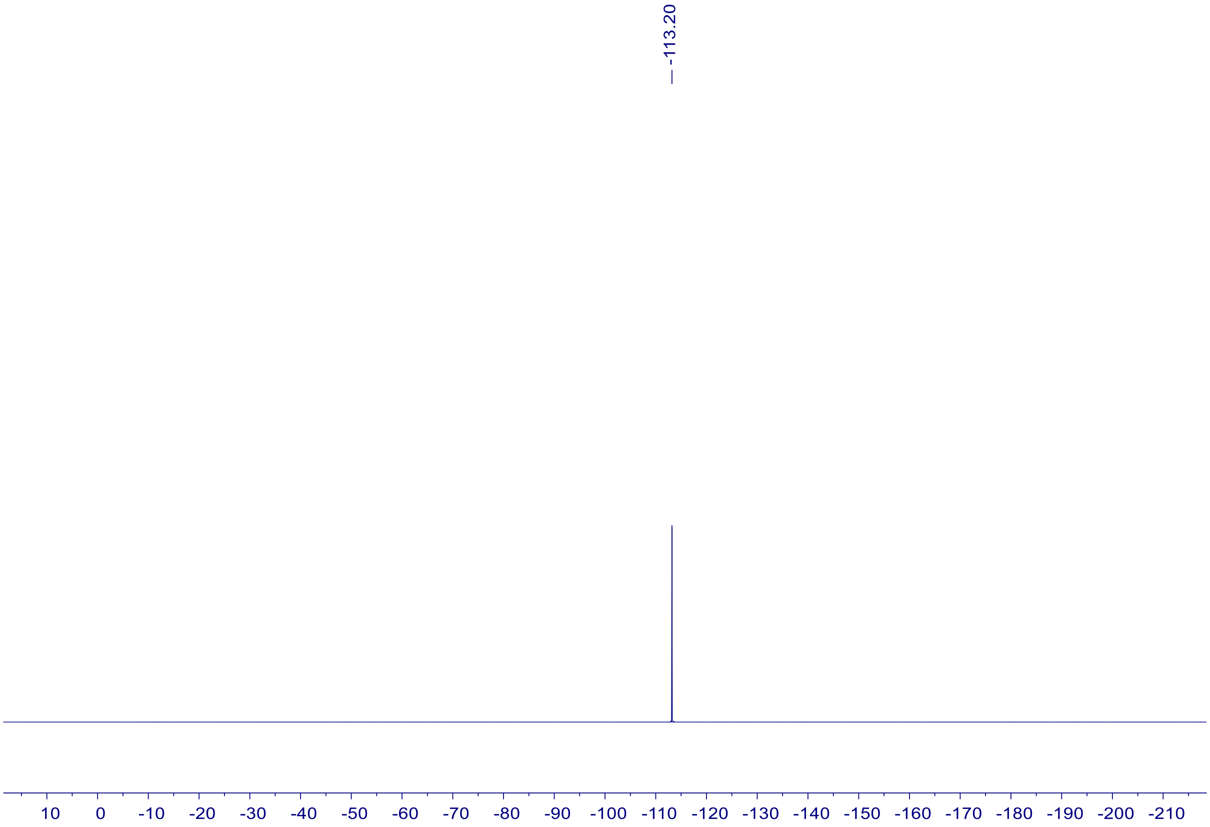

15 – ^{1}H NMR (400 MHz, $CDCl_3$)

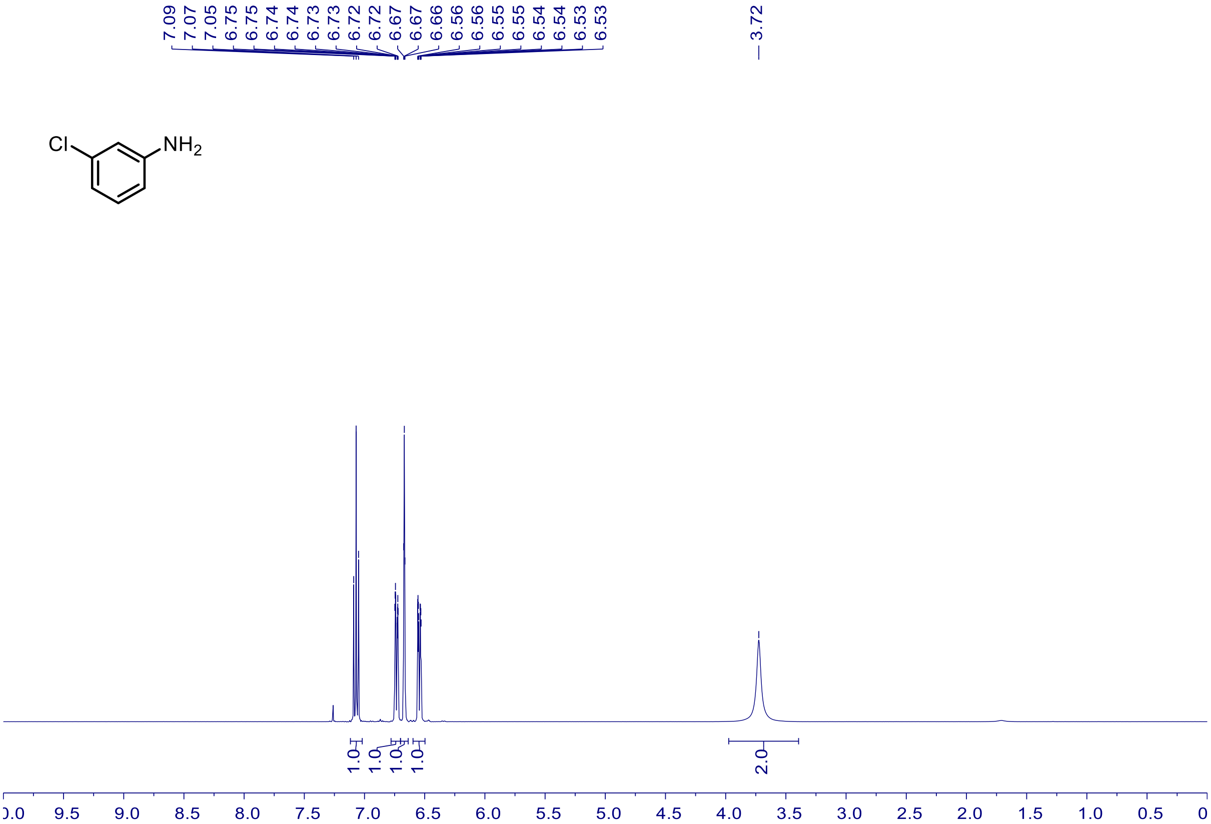

15 – ^{13}C NMR (101 MHz, $CDCl_3$)

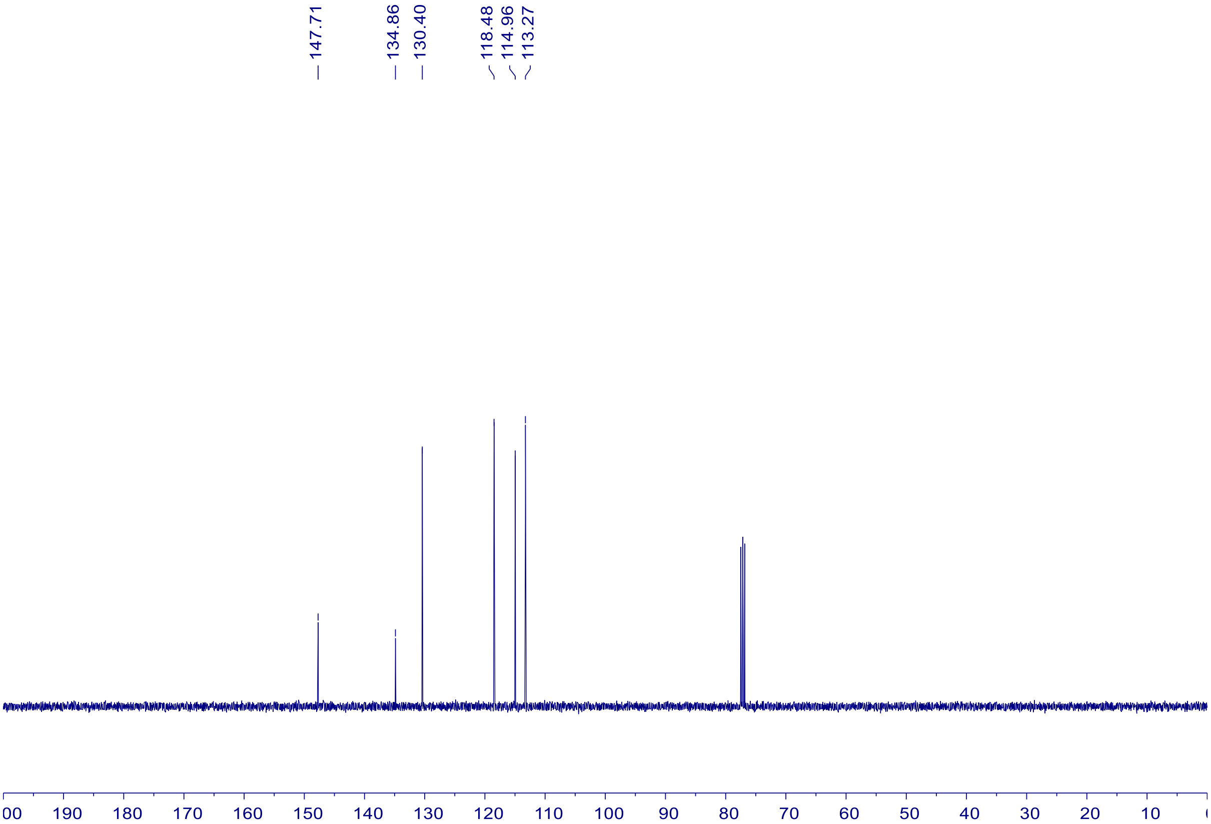

16 – ^{1}H NMR (400 MHz, $CDCl_3$)

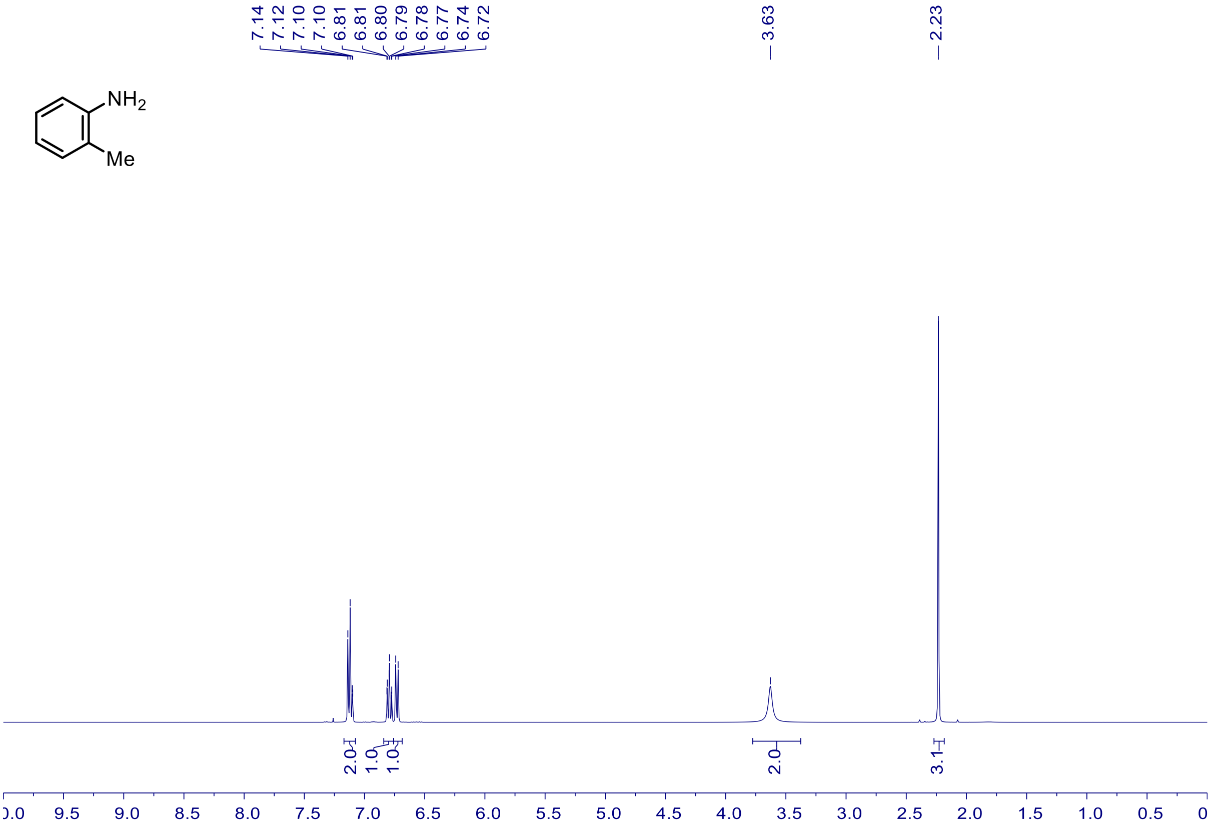

16 – ^{13}C NMR (101 MHz, $CDCl_3$)

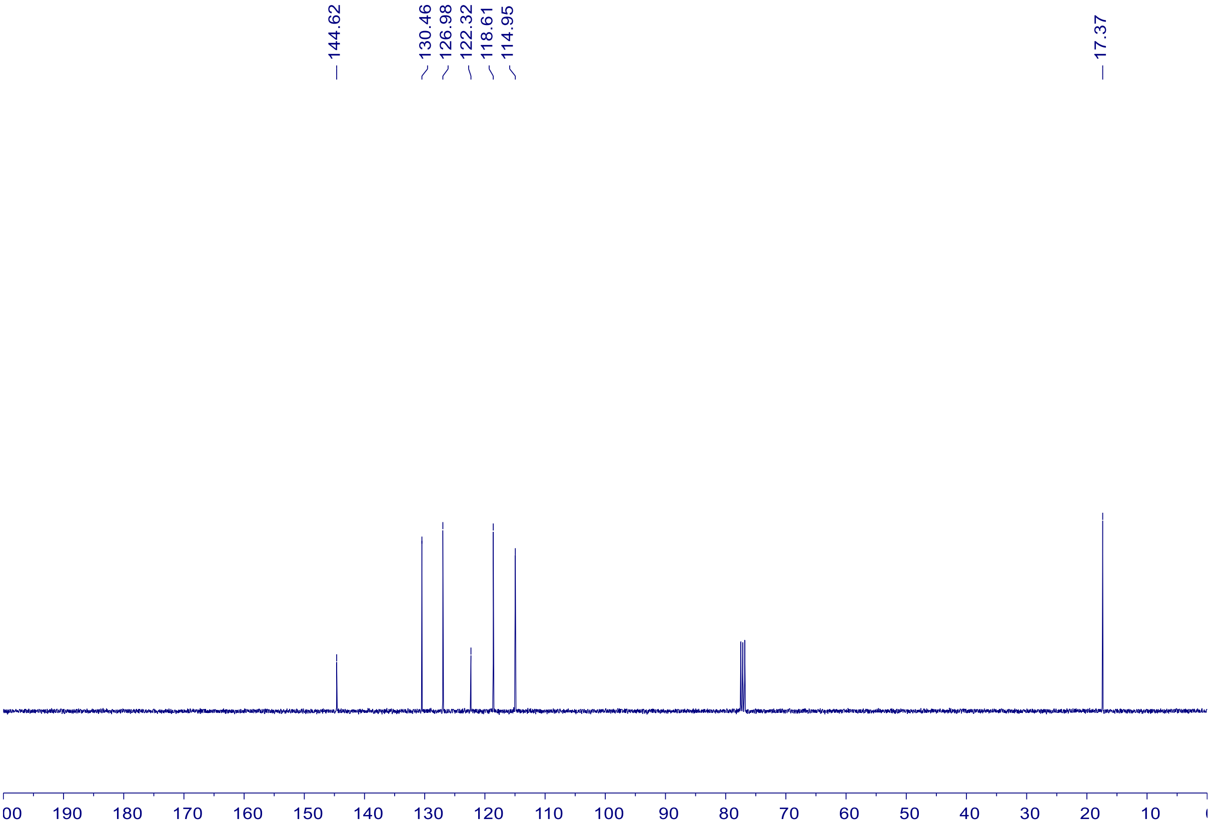

17 – ^{1}H NMR (400 MHz, $CDCl_3$)

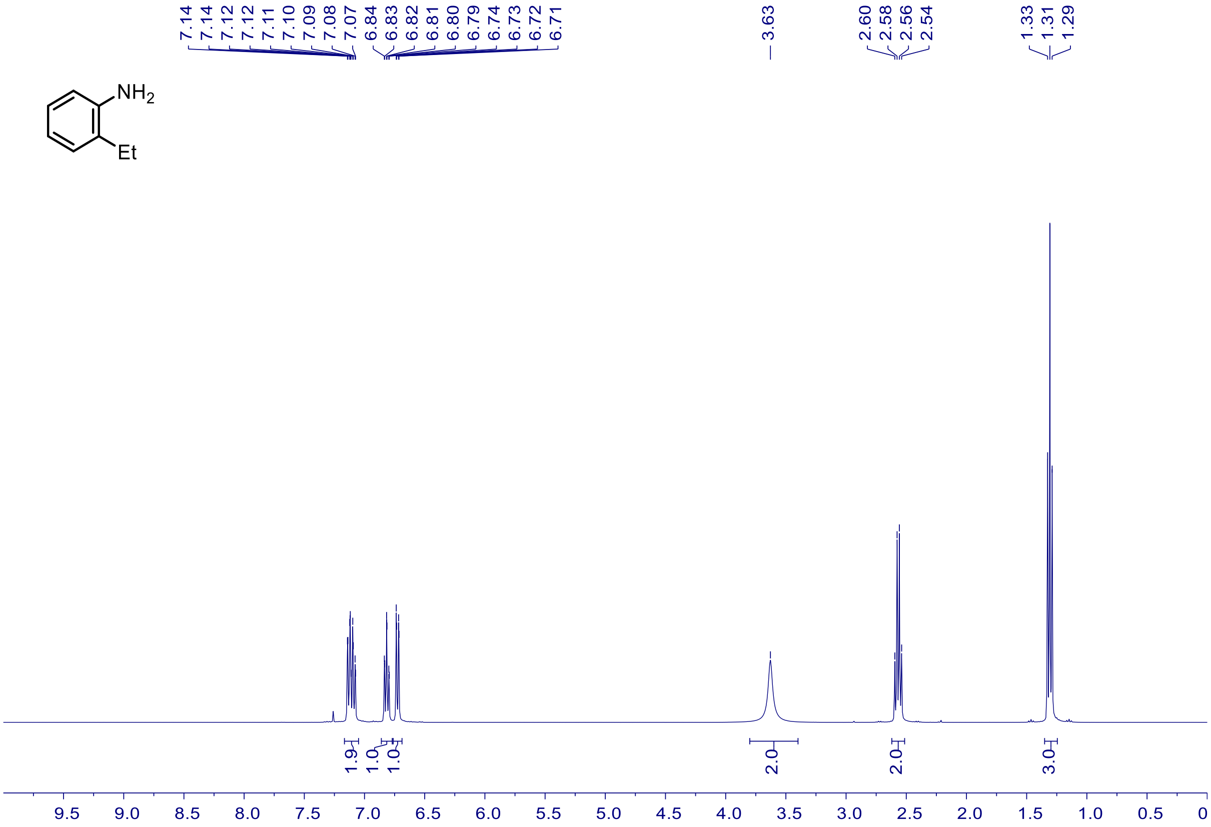

17 – ^{13}C NMR (101 MHz, $CDCl_3$)

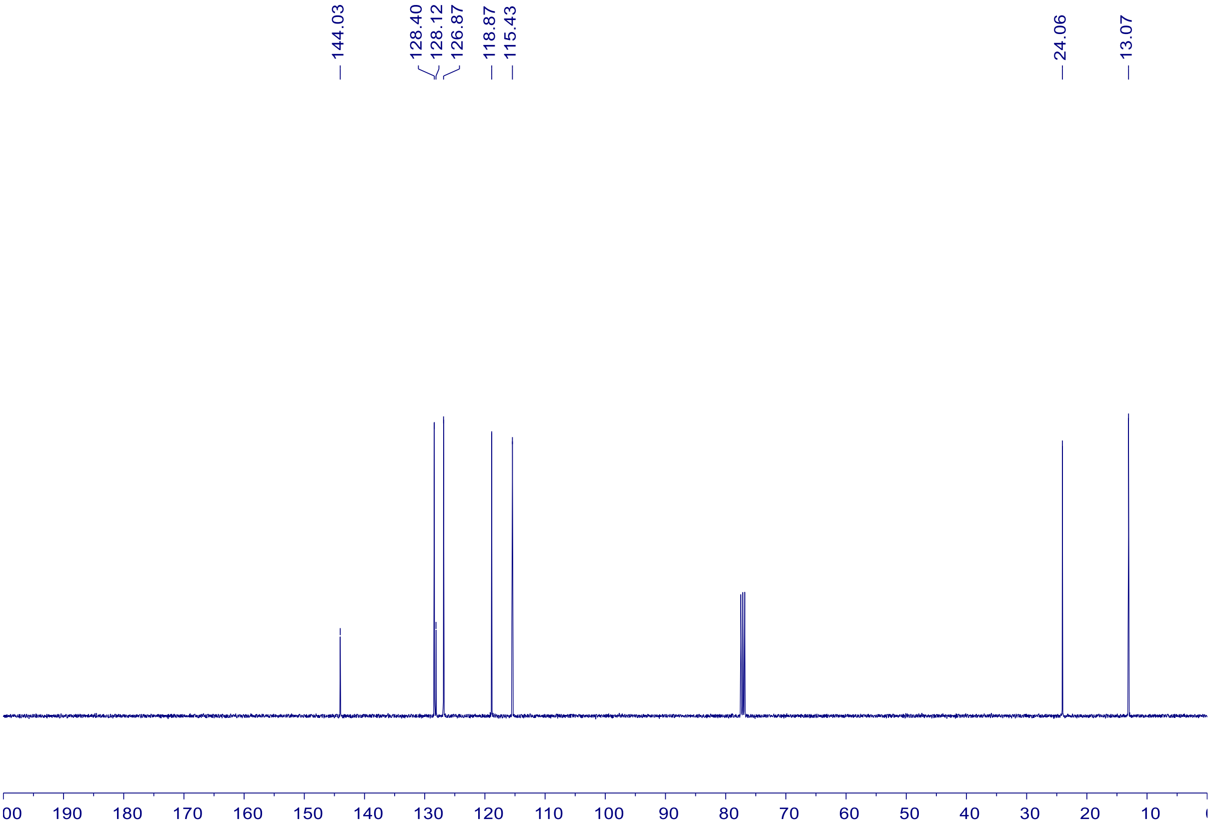

18 – ^{1}H NMR (400 MHz, $CDCl_3$)

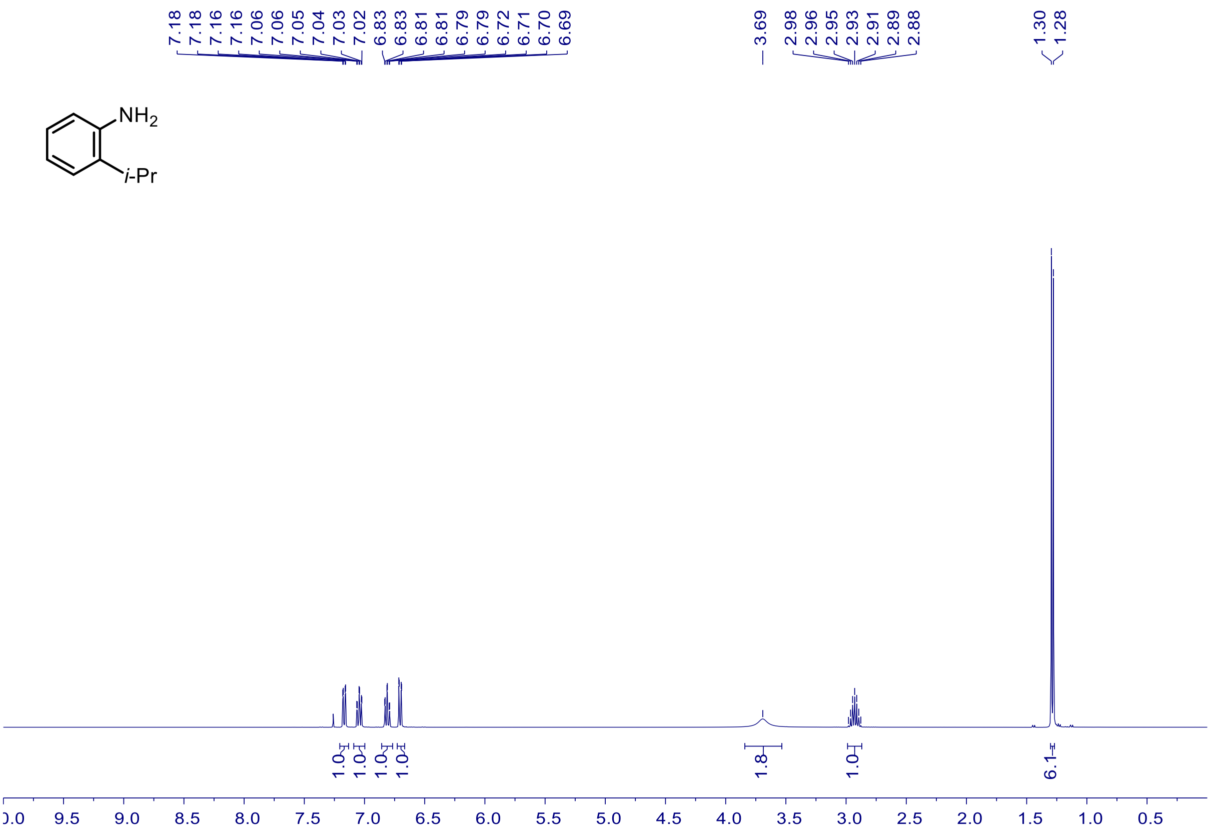

18 – ^{13}C NMR (101 MHz, $CDCl_3$)

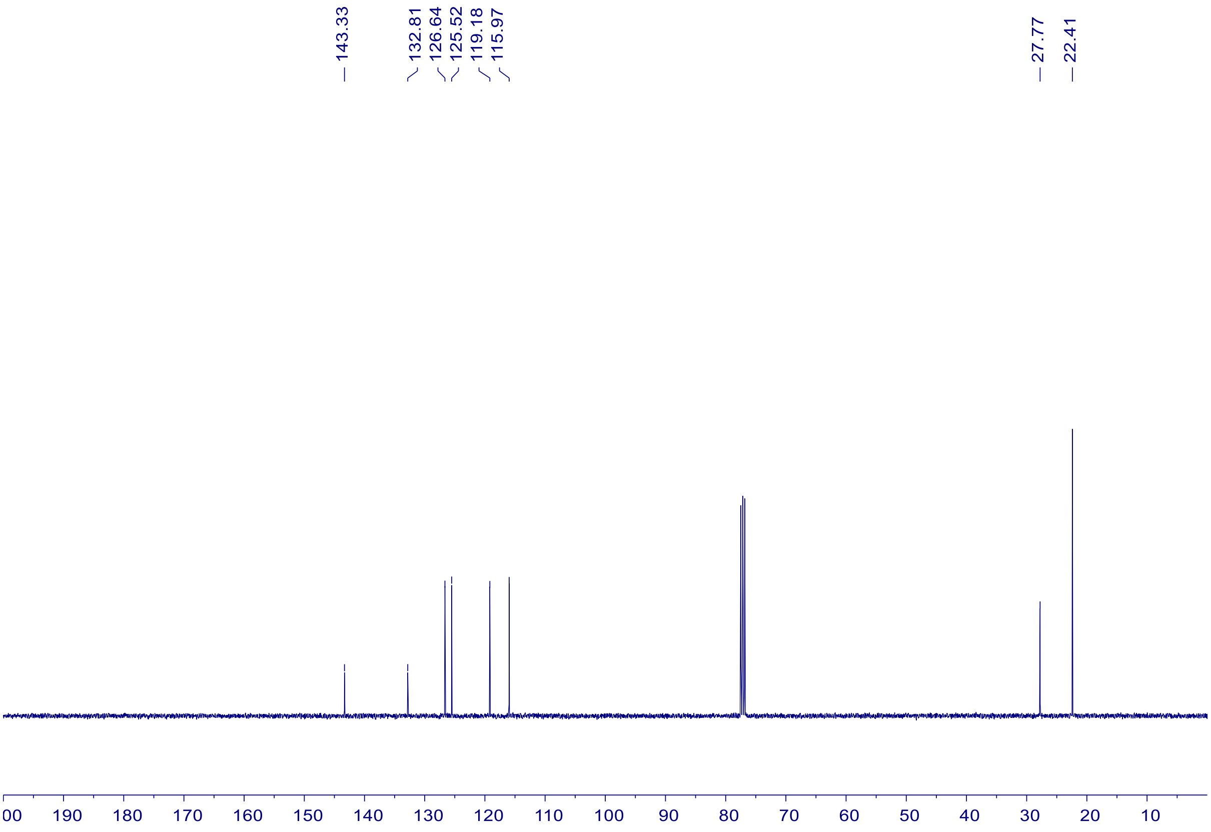

19 – ^{1}H NMR (400 MHz, $CDCl_3$)

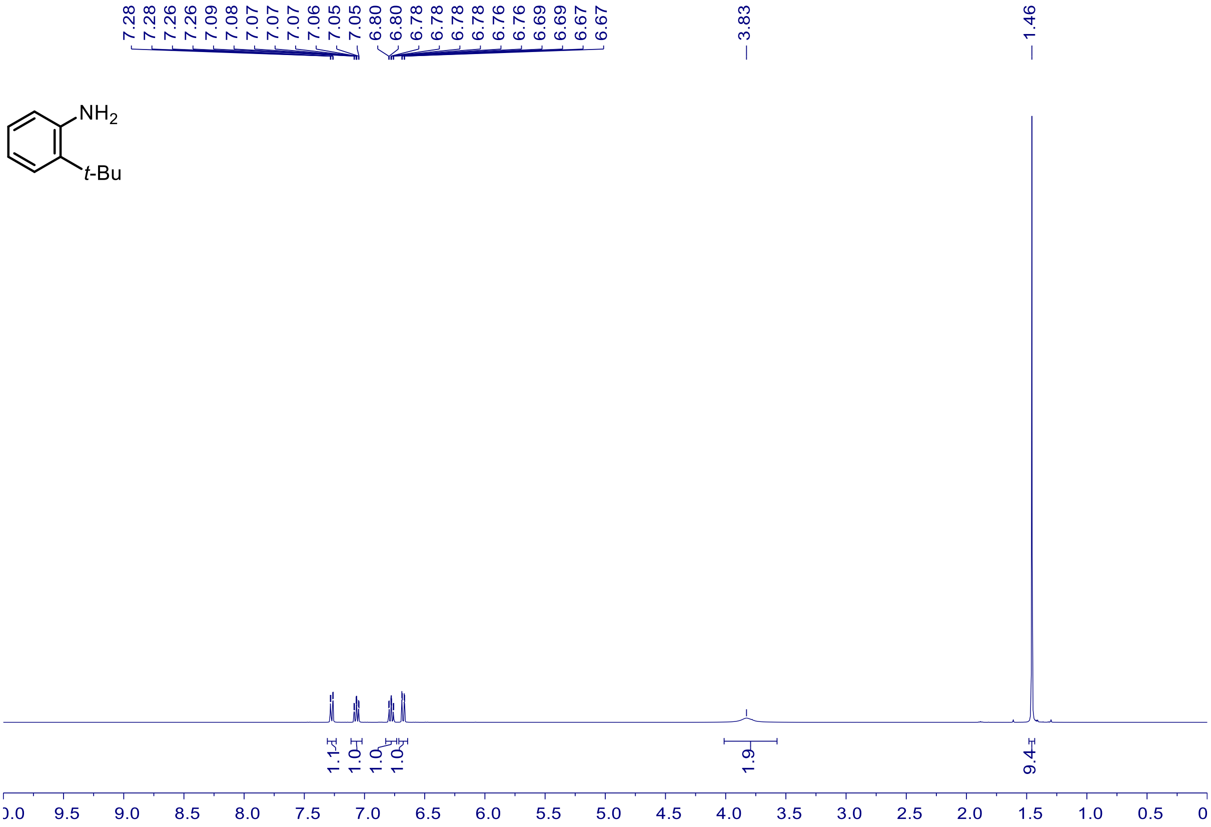

19 – ^{13}C NMR (101 MHz, $CDCl_3$)

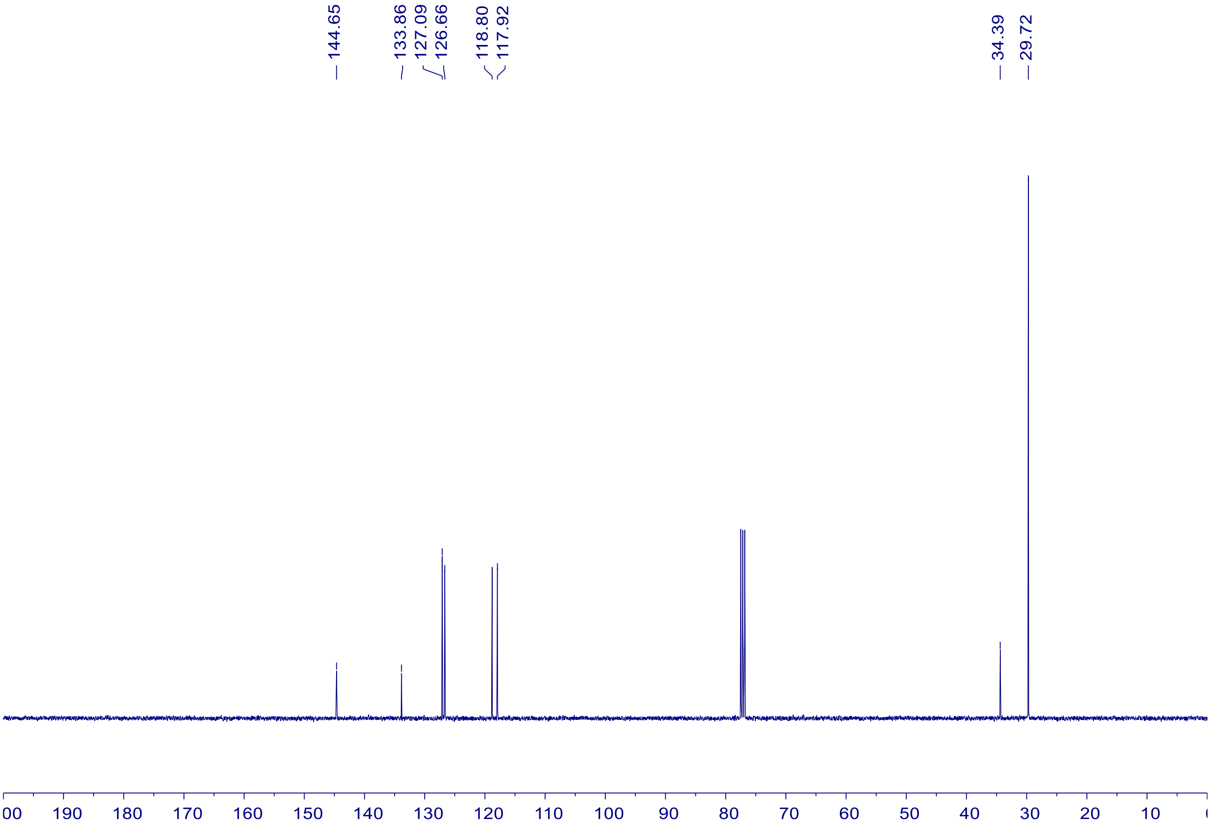

20 – ^{1}H NMR (500 MHz, $CDCl_3$)

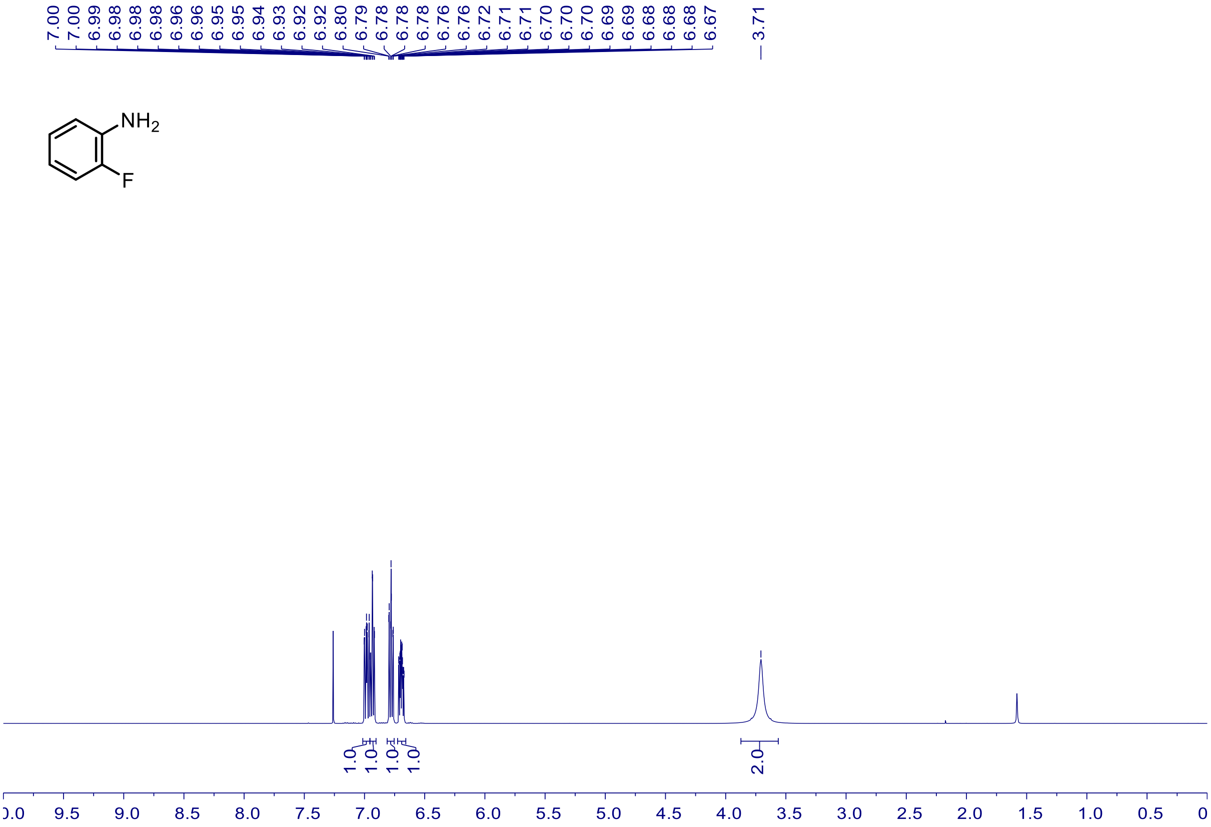

20 – ^{13}C NMR (126 MHz, $CDCl_3$)

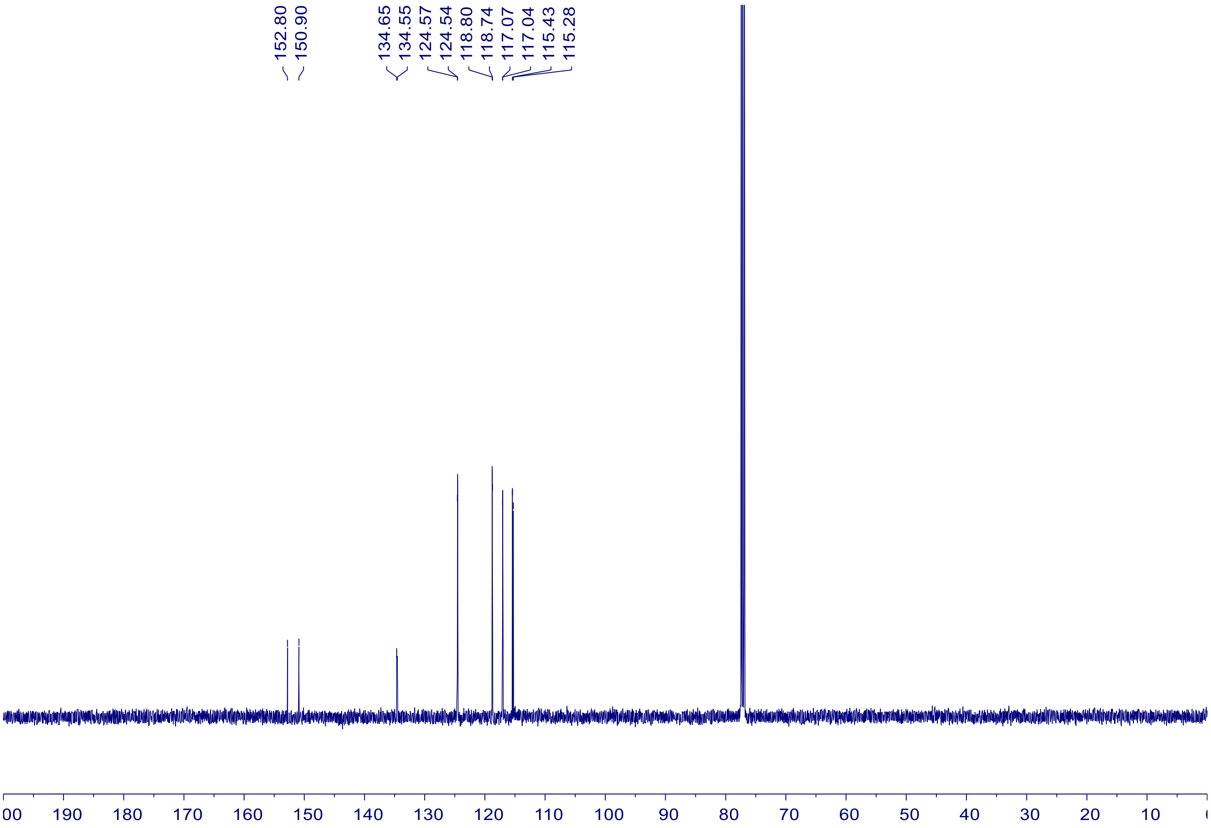

20 – ^{19}F NMR (376 MHz, $CDCl_3$)

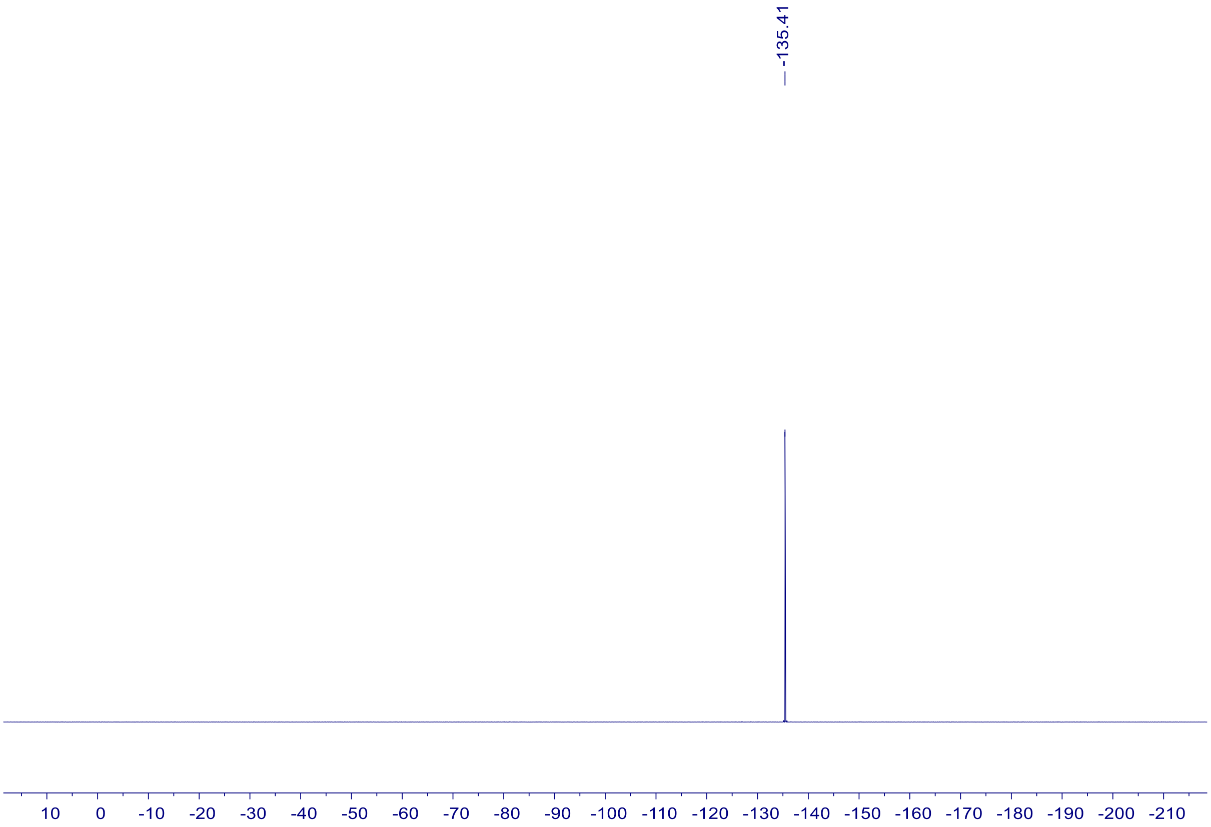

21 – ^{1}H NMR (400 MHz, $CDCl_3$)

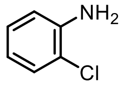

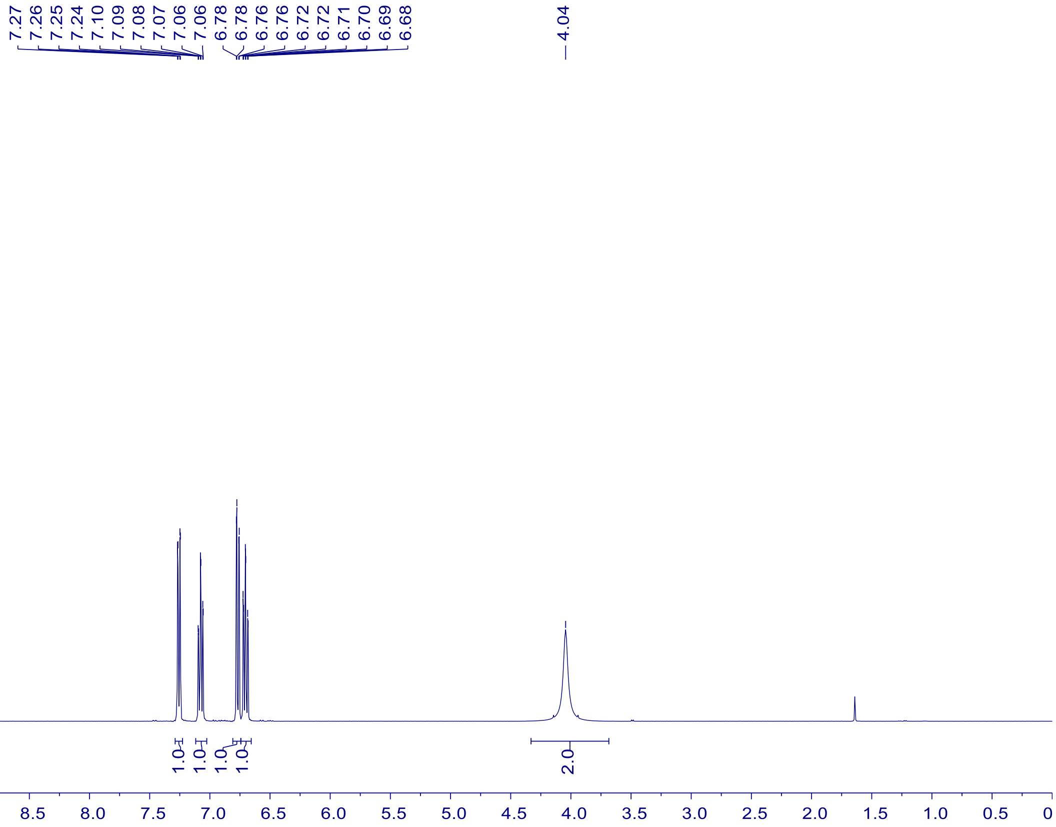

21 – ^{13}C NMR (101 MHz, $CDCl_3$)

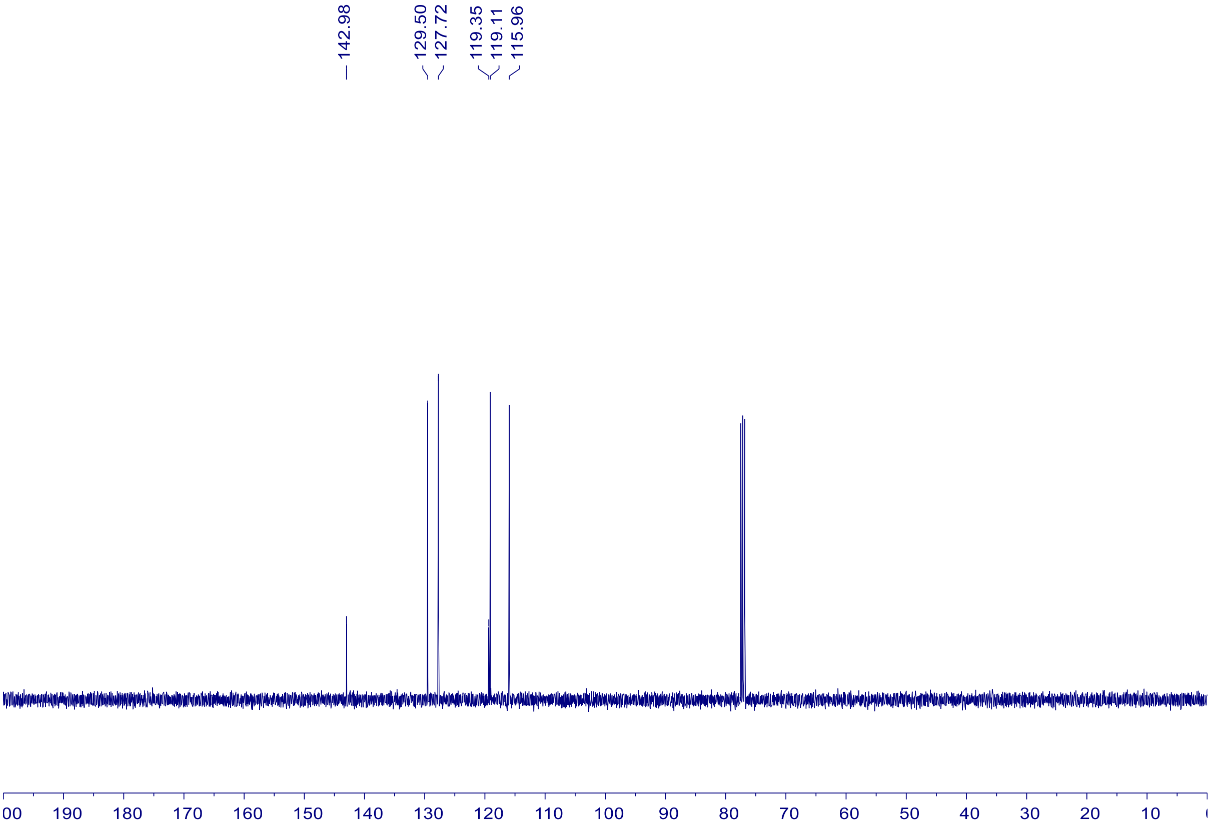

22 – ^{1}H NMR (500 MHz, $CDCl_3$)

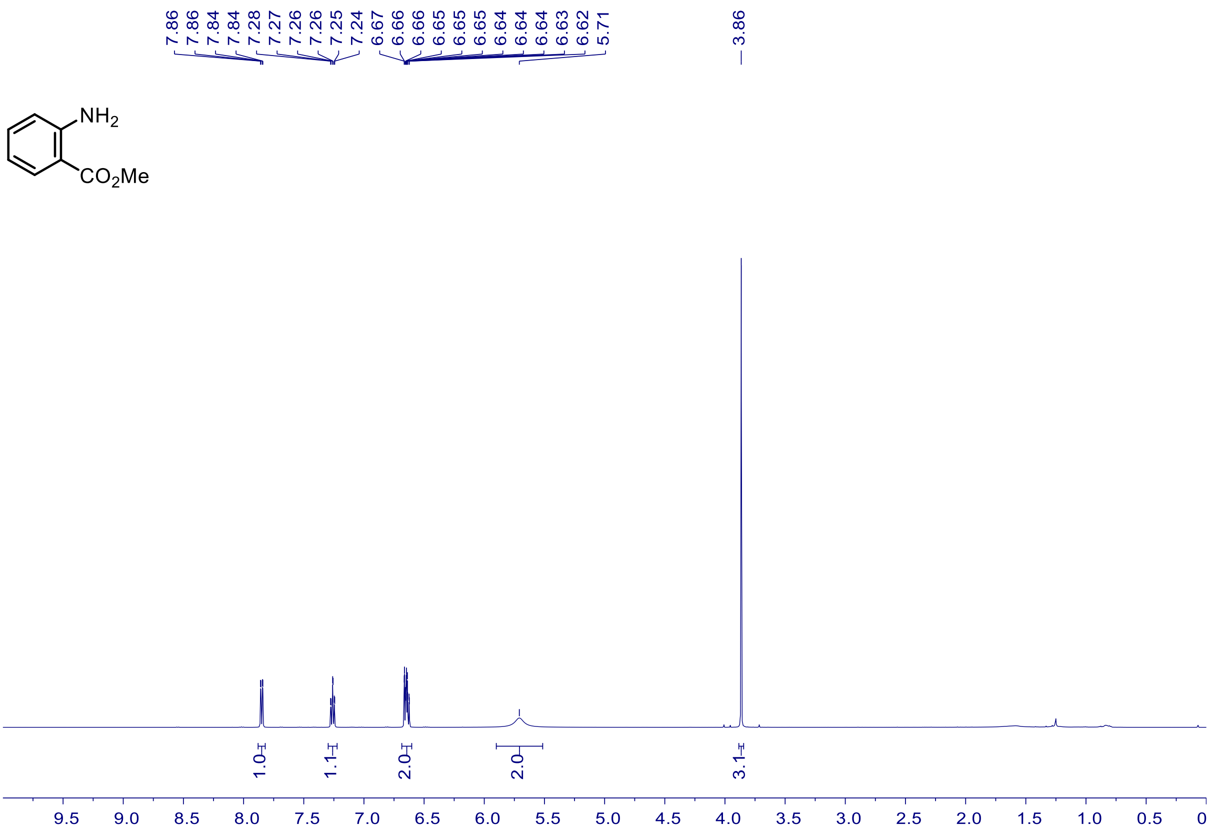

22 – ^{13}C NMR (126 MHz, $CDCl_3$)

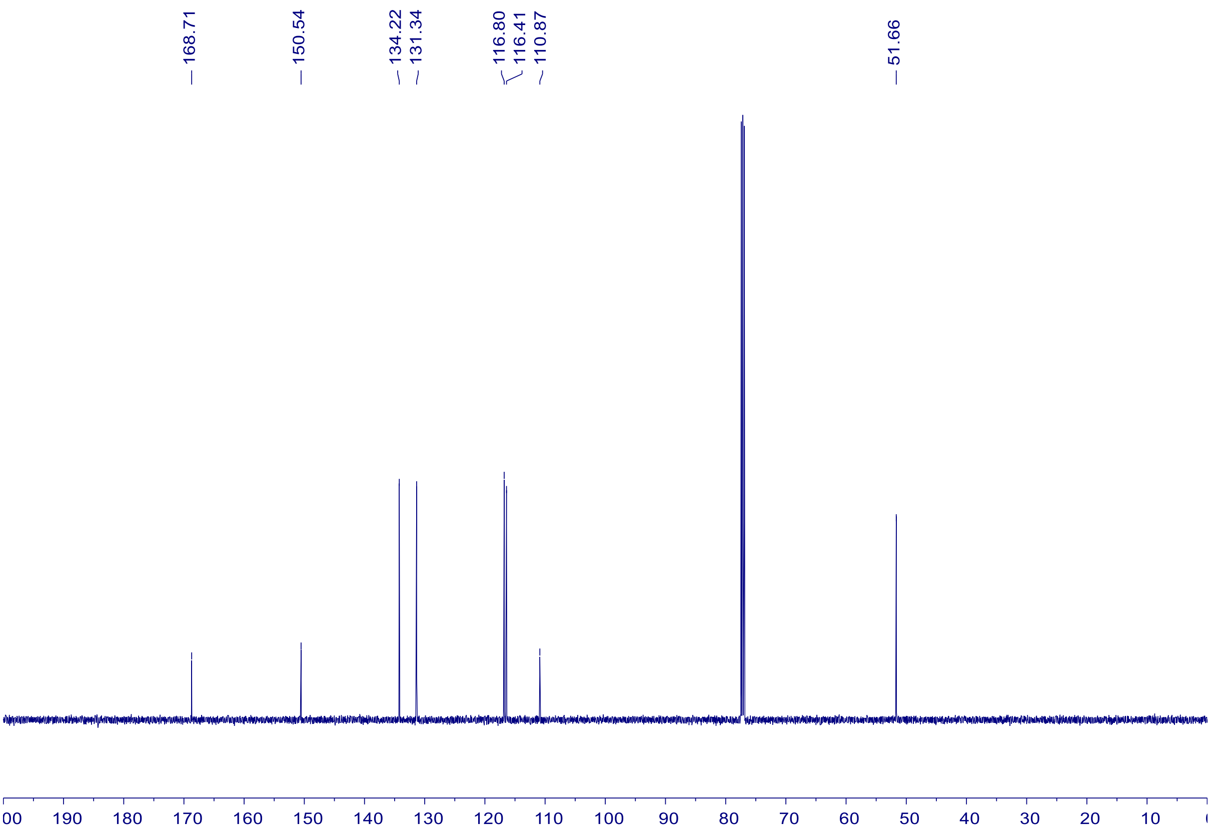

23 – ^{1}H NMR (400 MHz, $CDCl_3$)

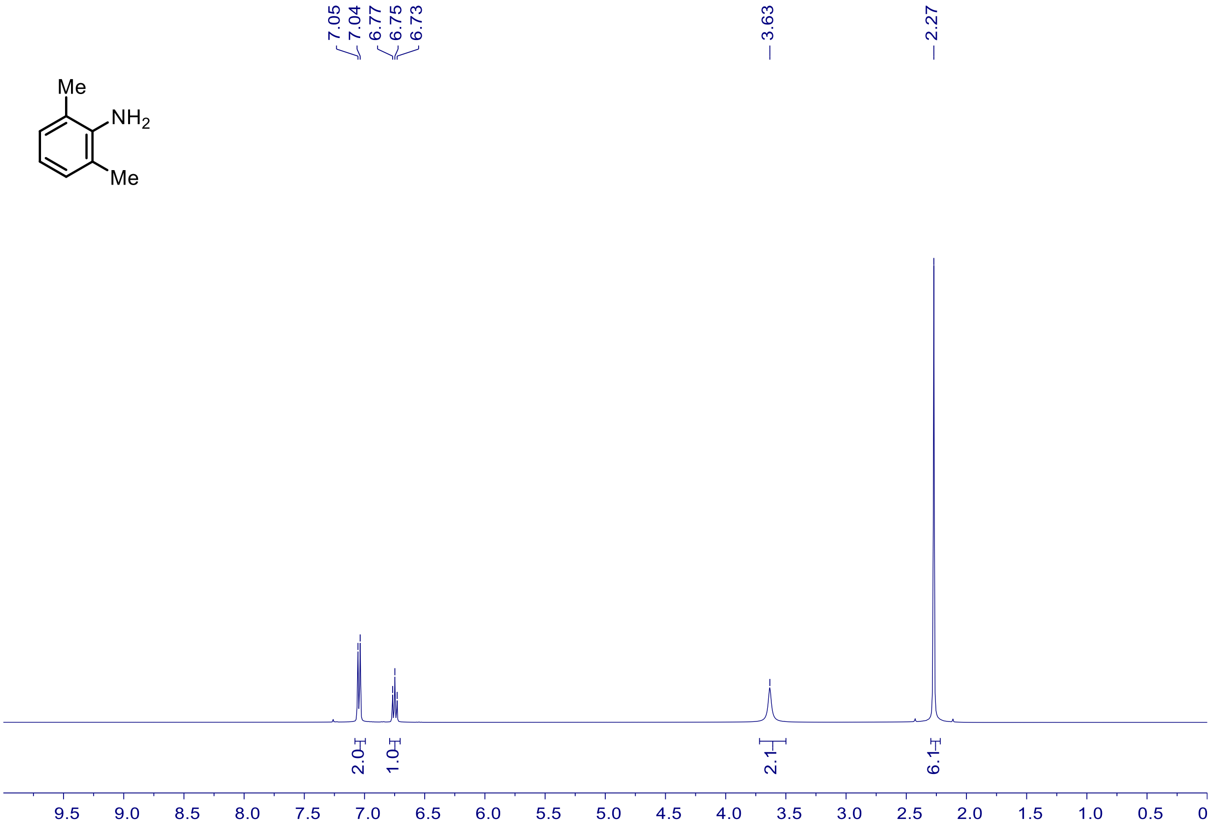

23 – ^{13}C NMR (101 MHz, $CDCl_3$)

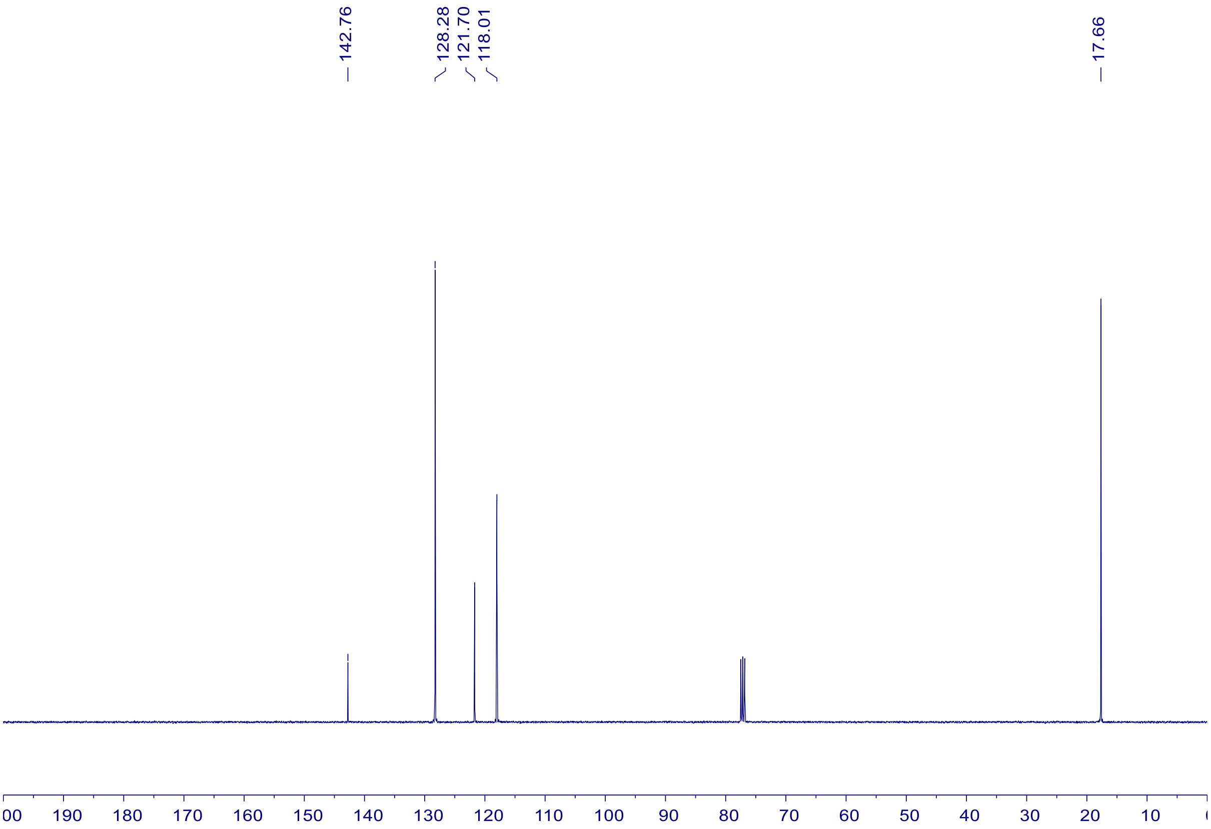

24 – ^{1}H NMR (400 MHz, $CDCl_3$)

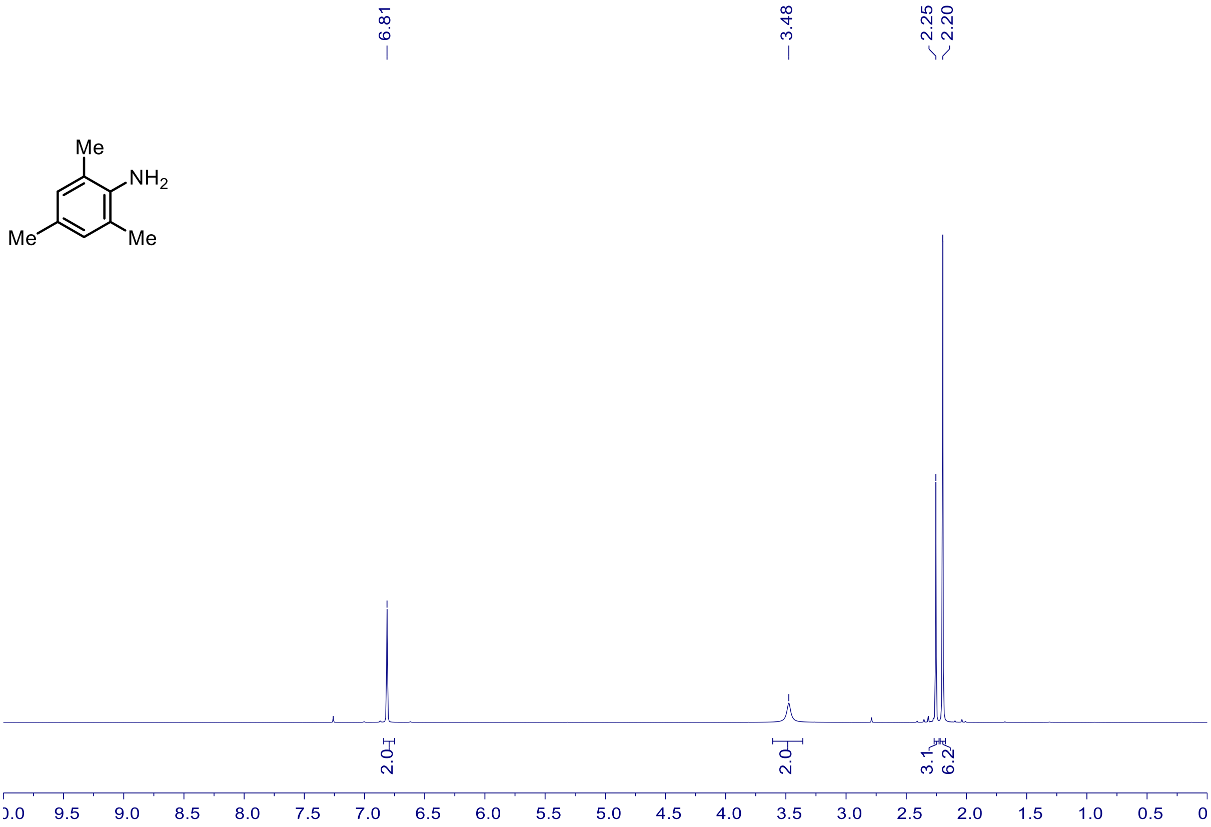

24 – ^{13}C NMR (101 MHz, $CDCl_3$)

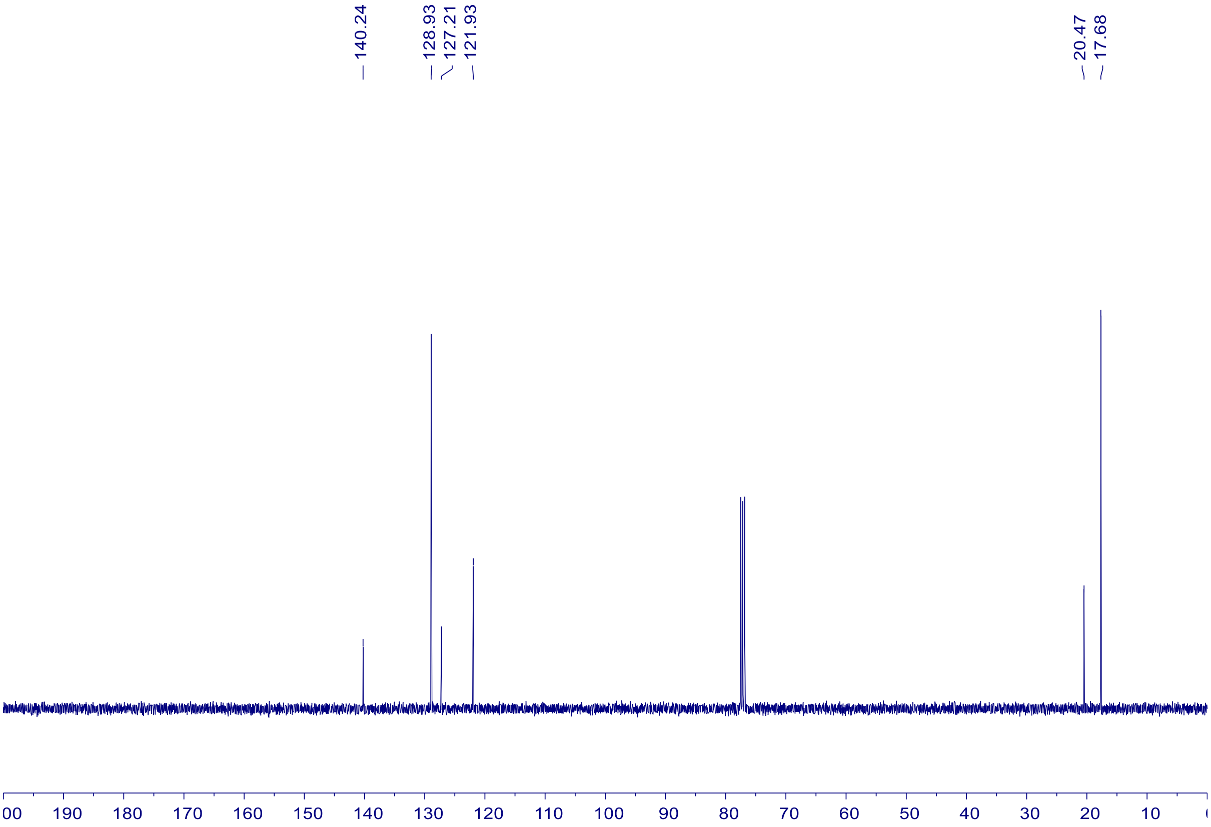

25 – ^{1}H NMR (500 MHz, $CDCl_3$)

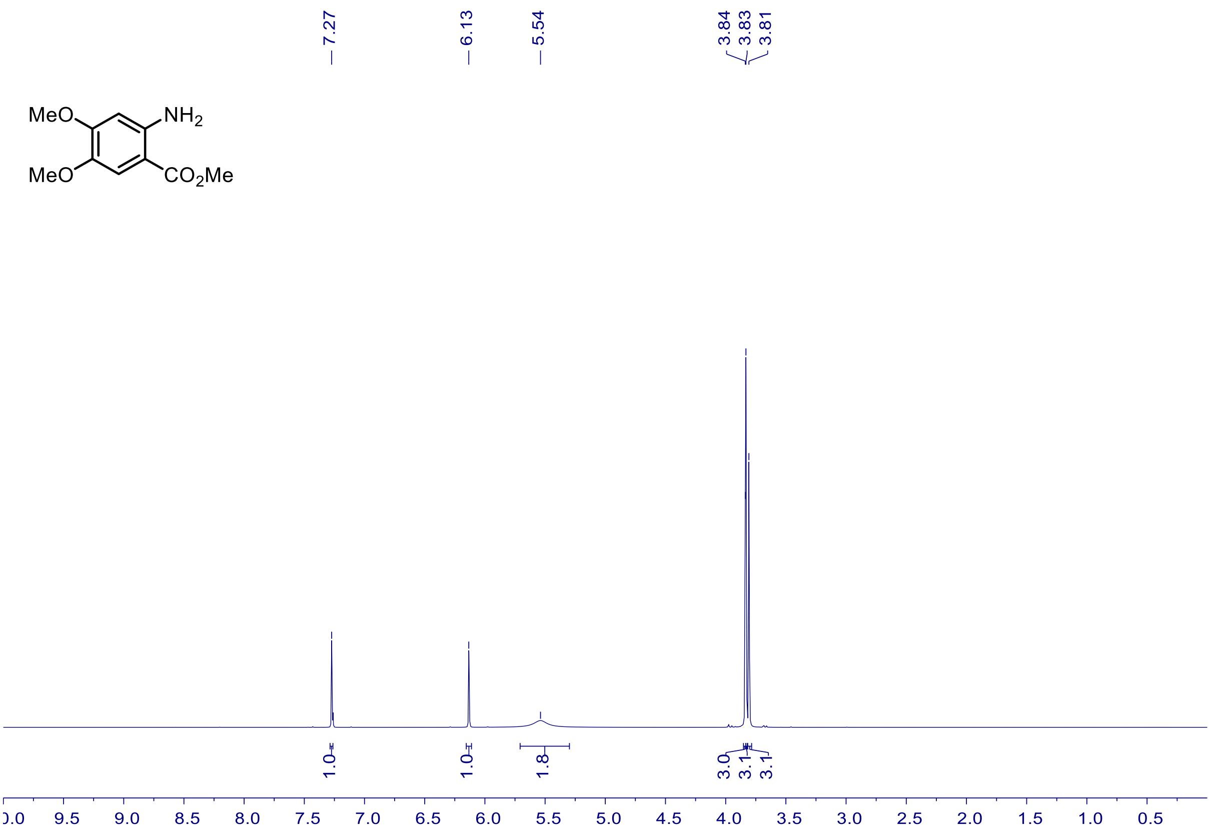

25 – ^{13}C NMR (126 MHz, $CDCl_3$)

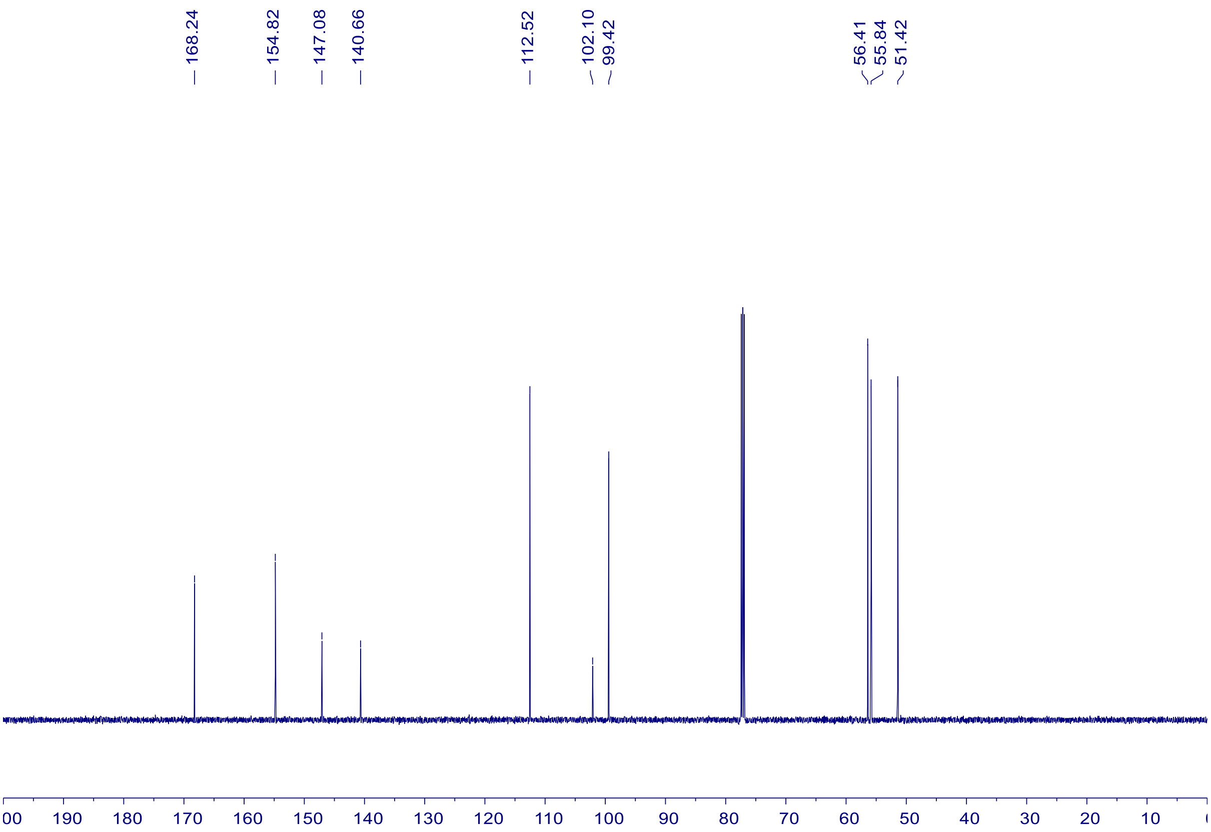

26 – ^{1}H NMR (500 MHz, $CDCl_3$)

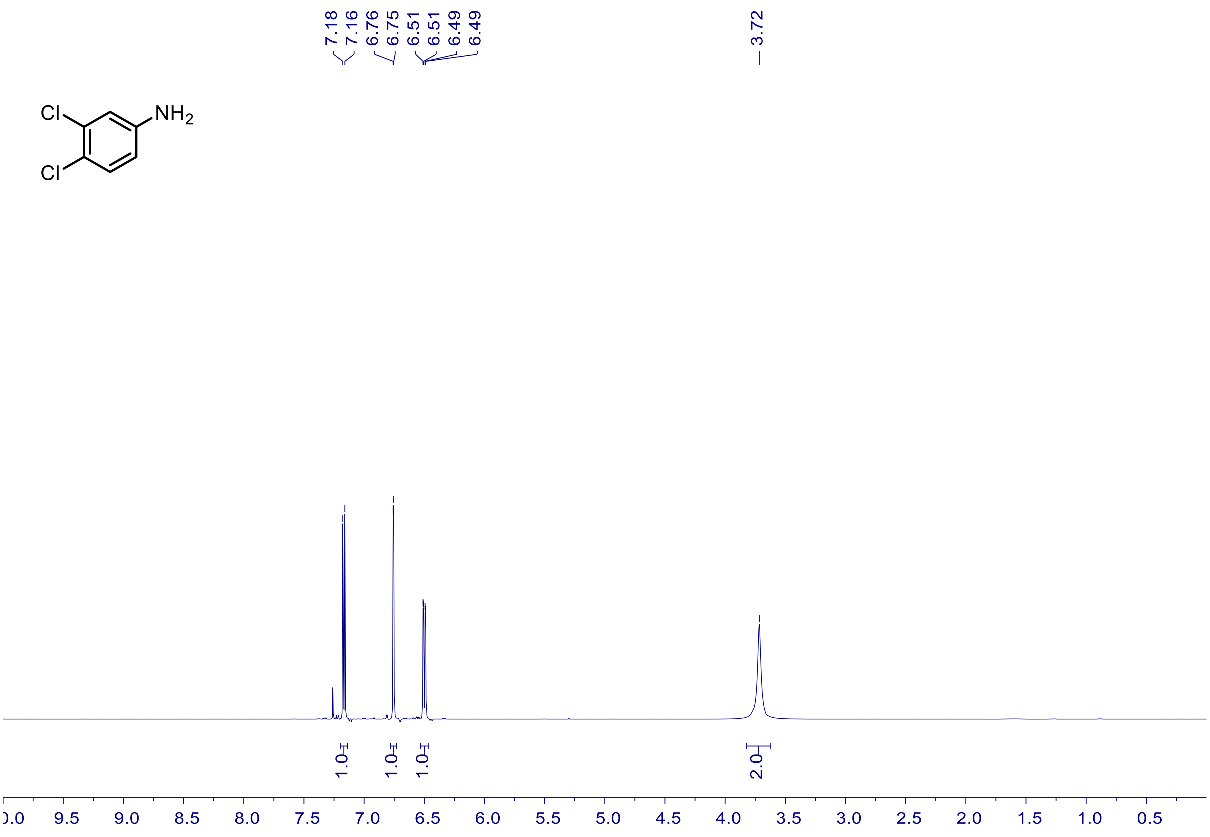

26 – ^{13}C NMR (126 MHz, $CDCl_3$)

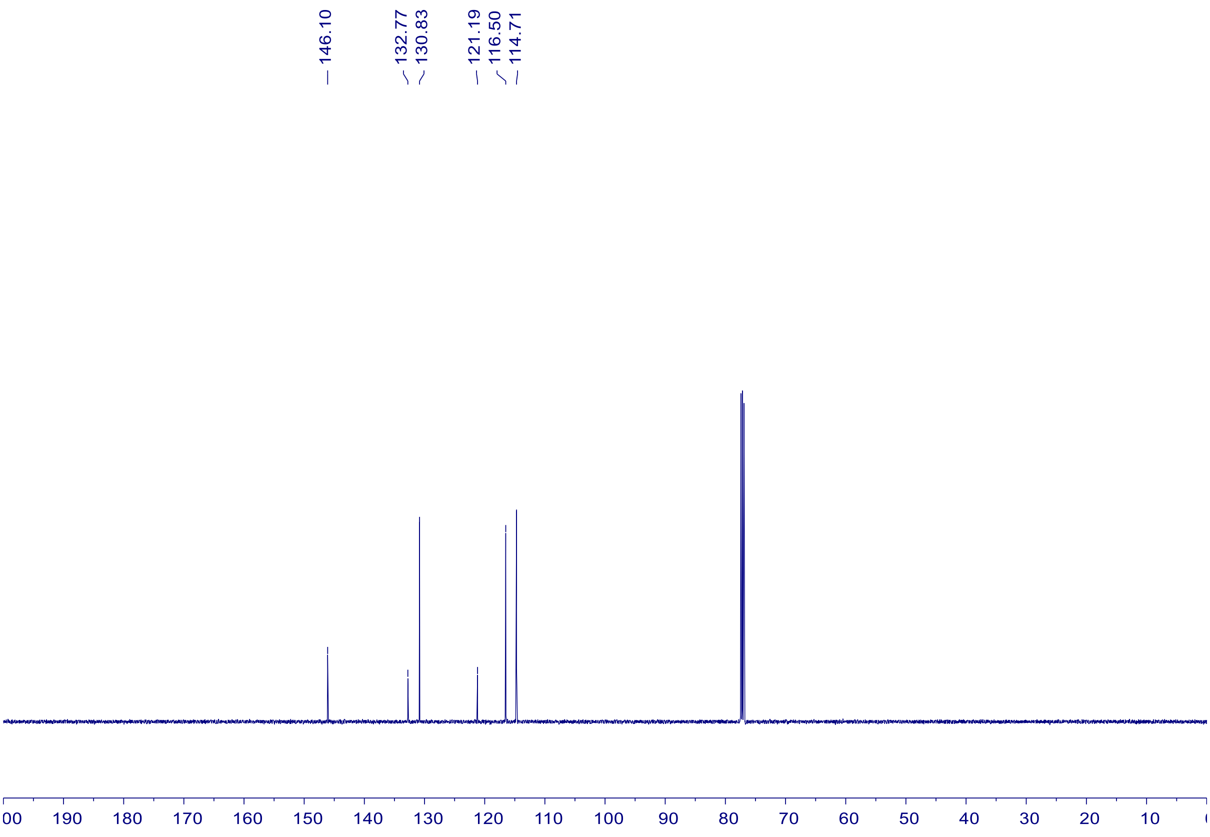

27 – ^{1}H NMR (400 MHz, $CDCl_3$)

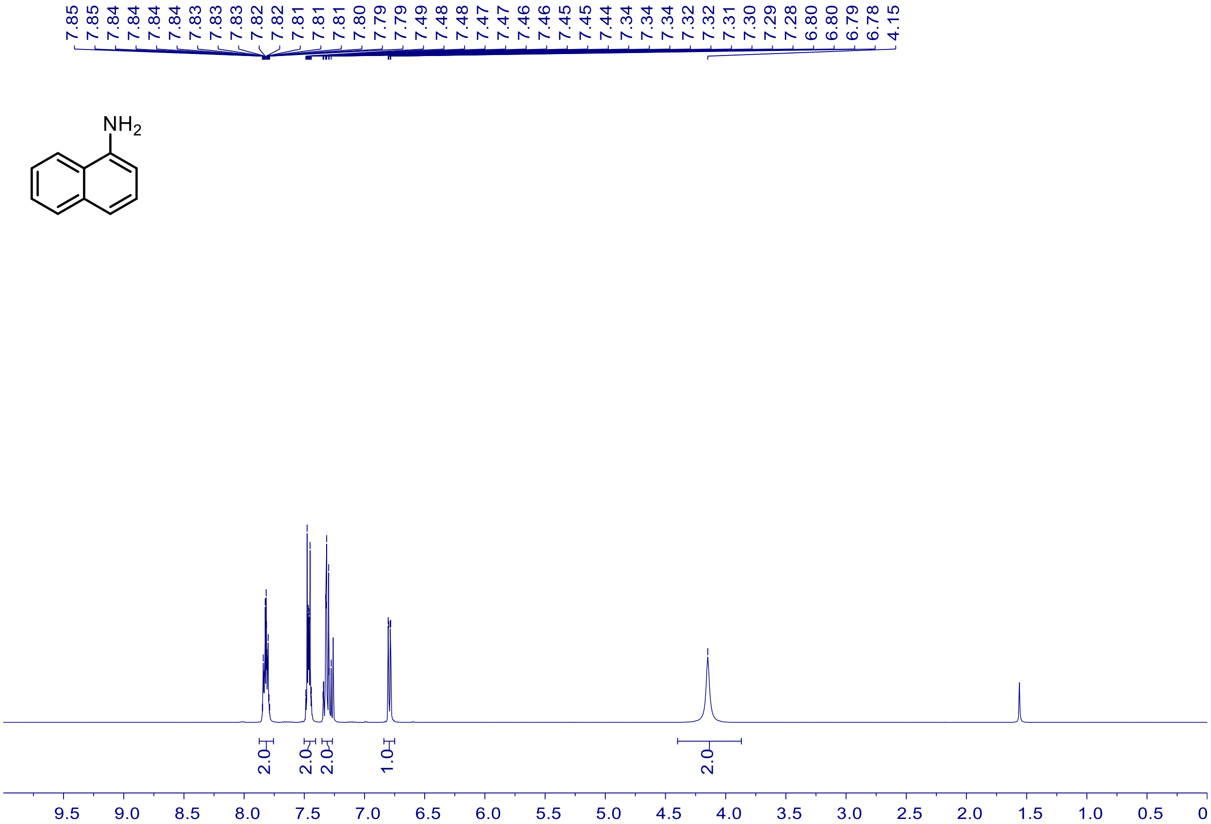

27 – ^{13}C NMR (101 MHz, $CDCl_3$)

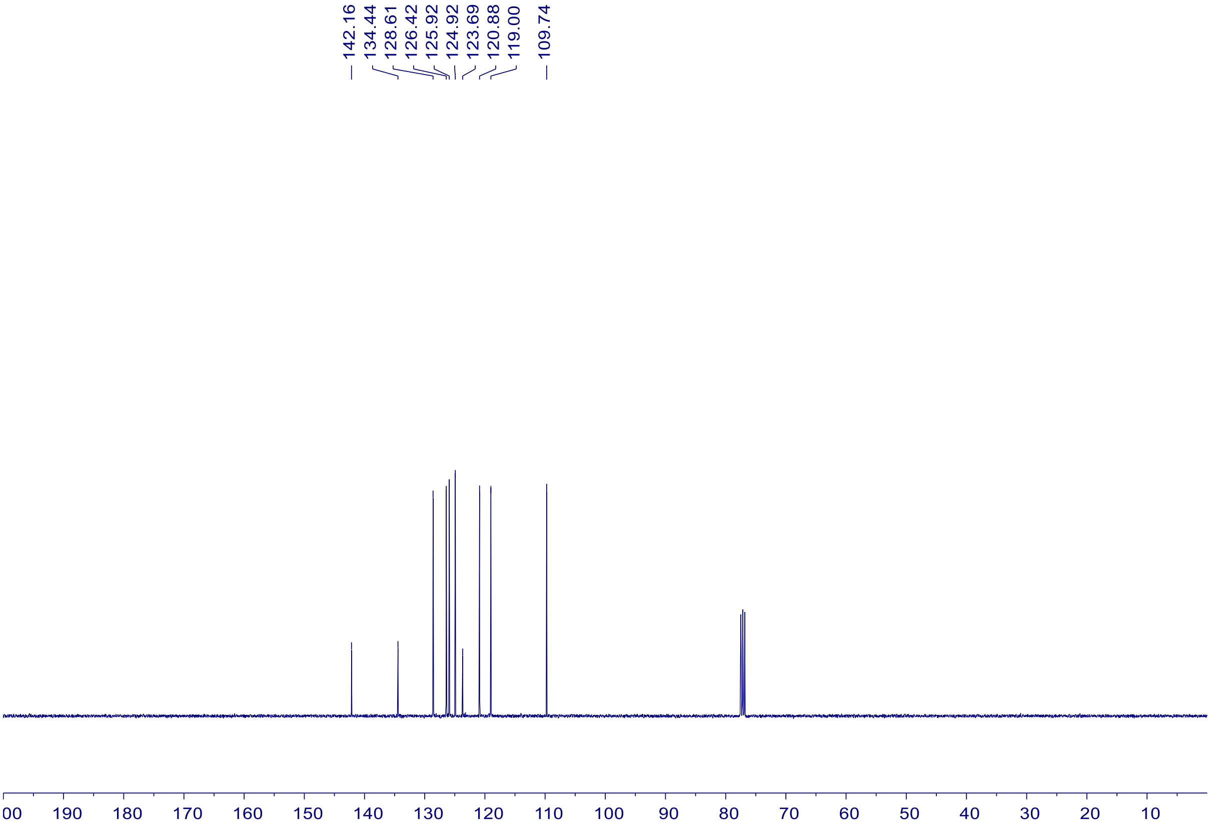

28 – ^{1}H NMR (500 MHz, $CDCl_3$)

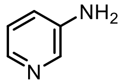

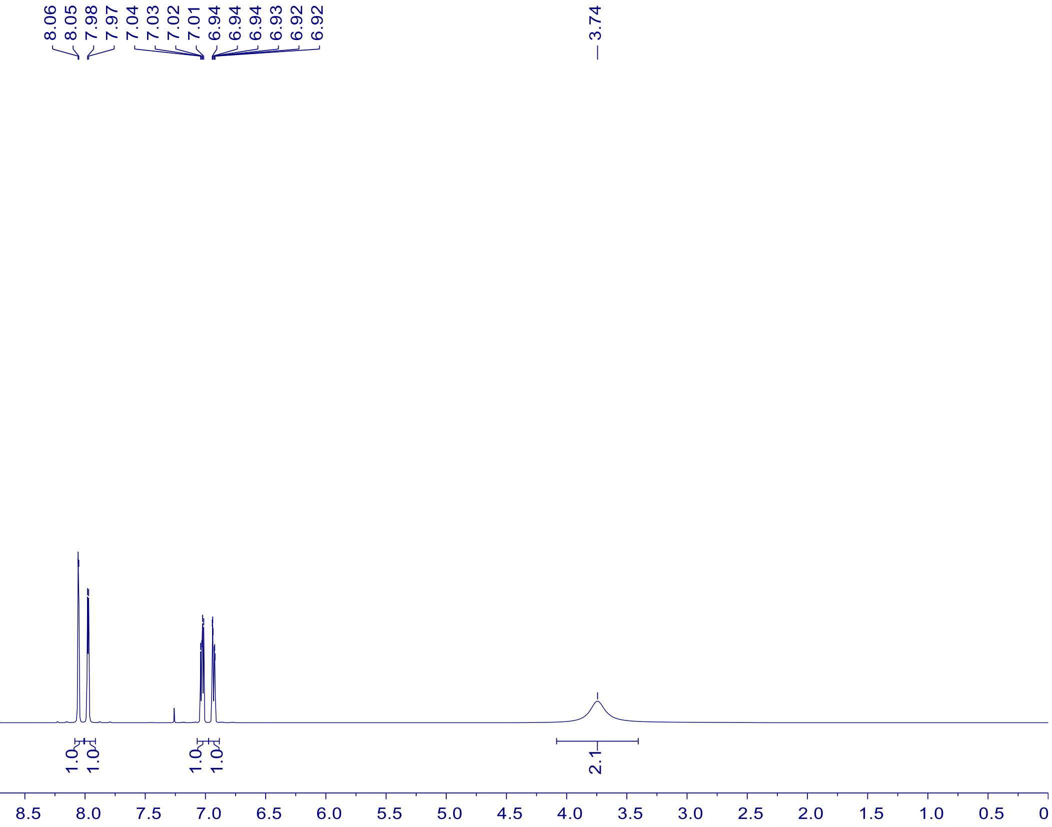

28 – ^{13}C NMR (126 MHz, $CDCl_3$)

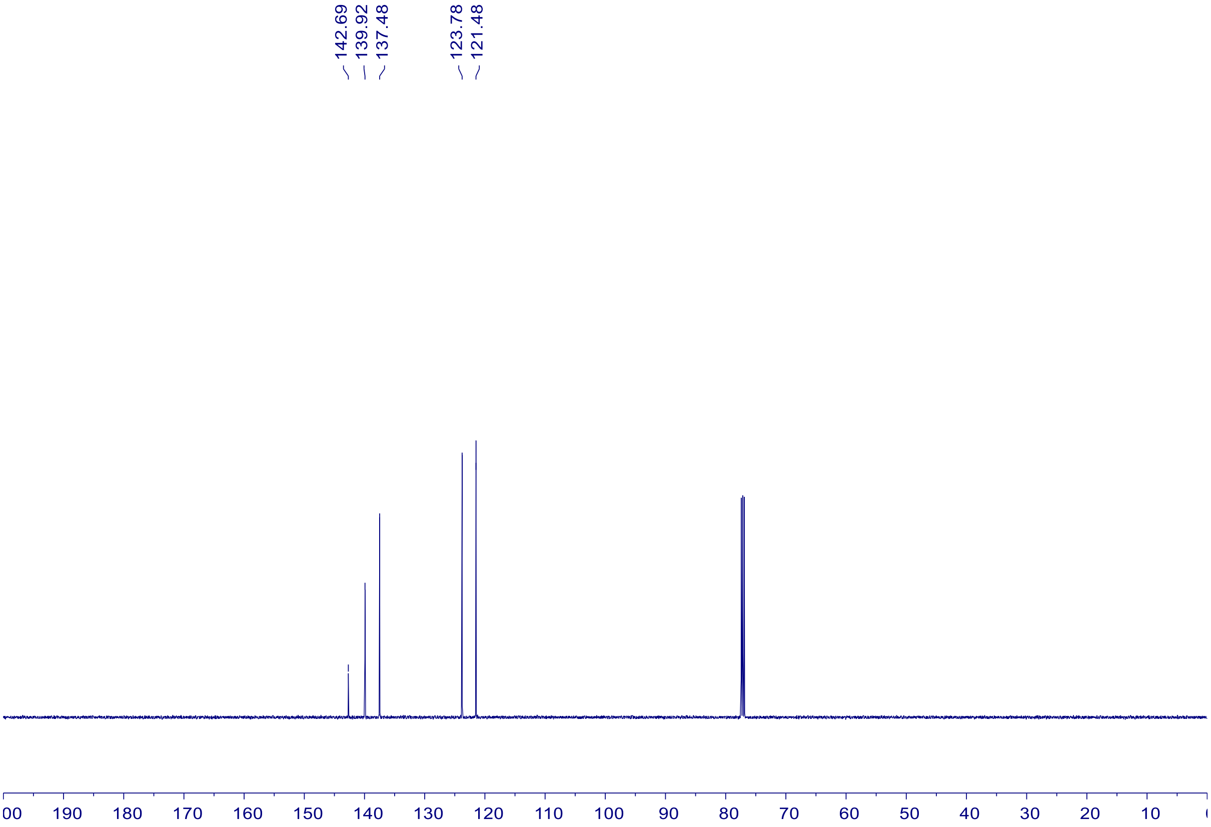

29 – ^{1}H NMR (500 MHz, $CDCl_3$)

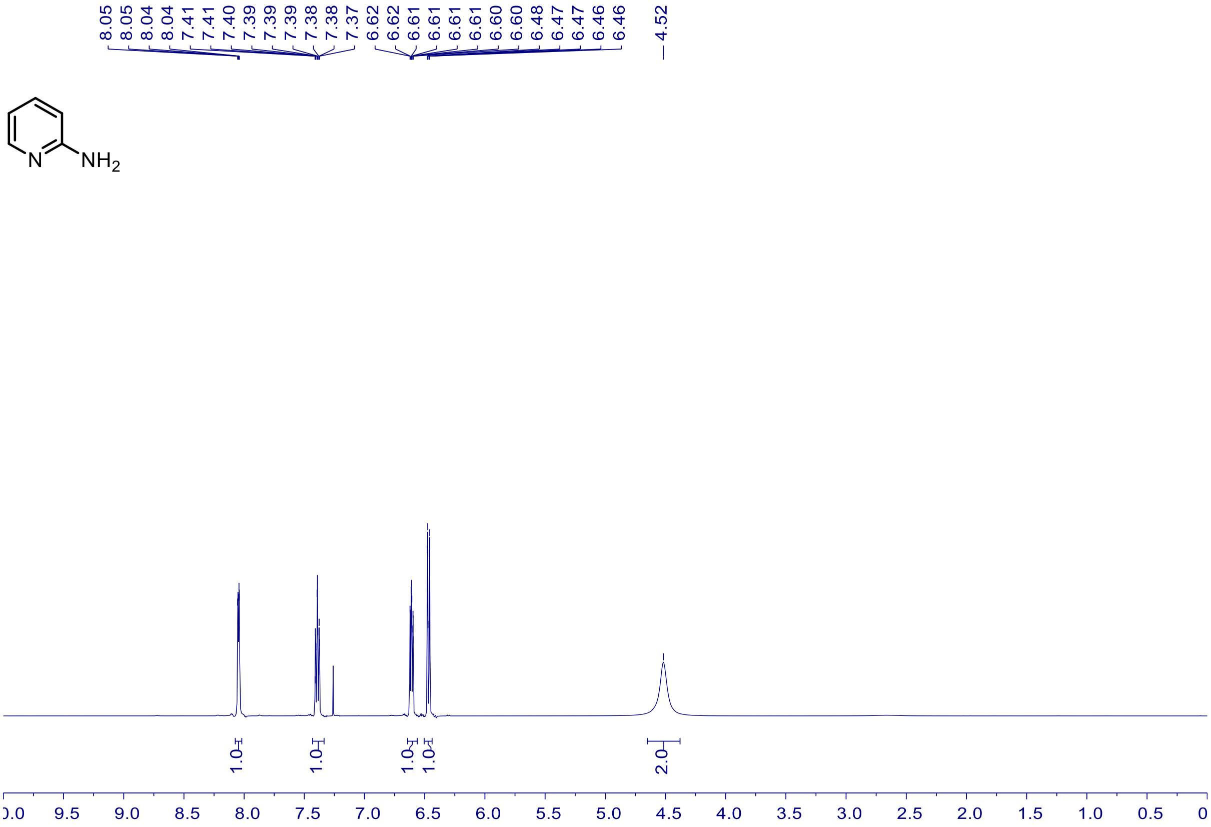

29 – ^{13}C NMR (126 MHz, $CDCl_3$)

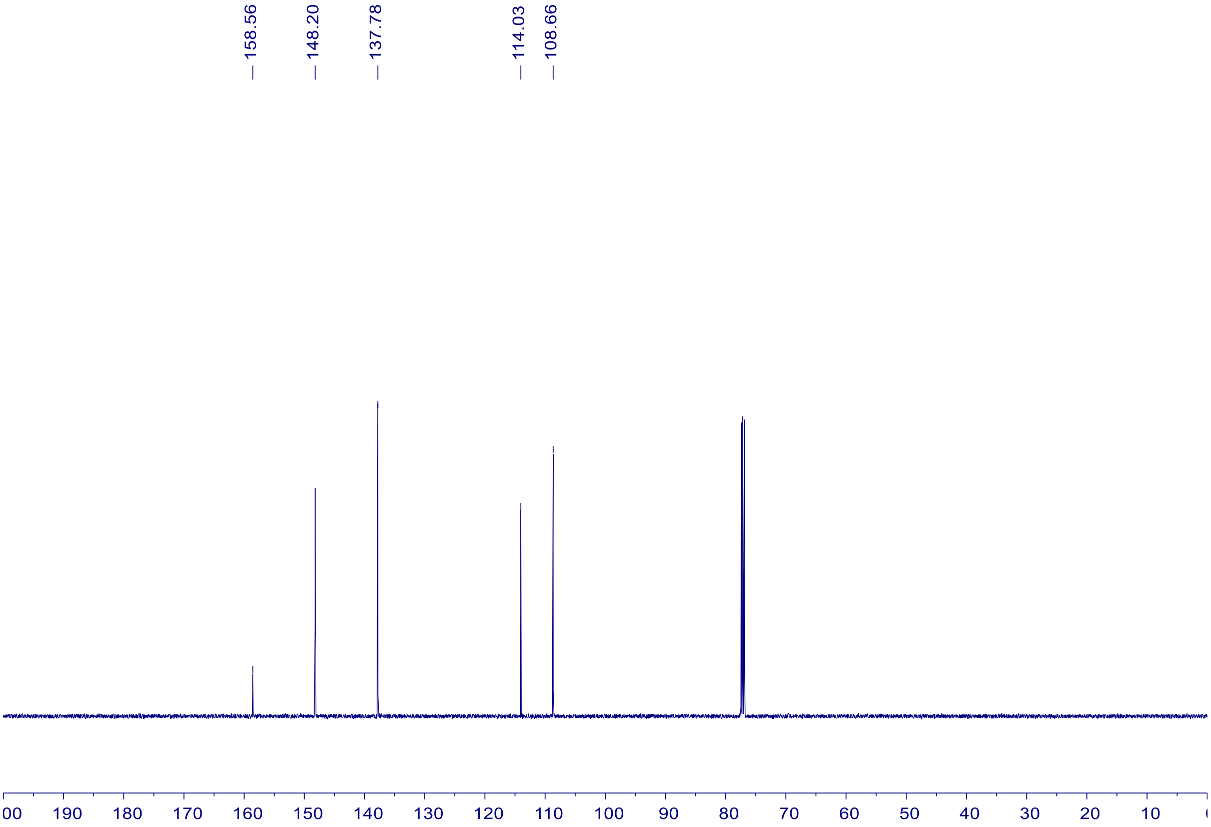

30 – ^{1}H NMR (500 MHz, CDCl$_3$)

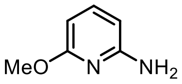

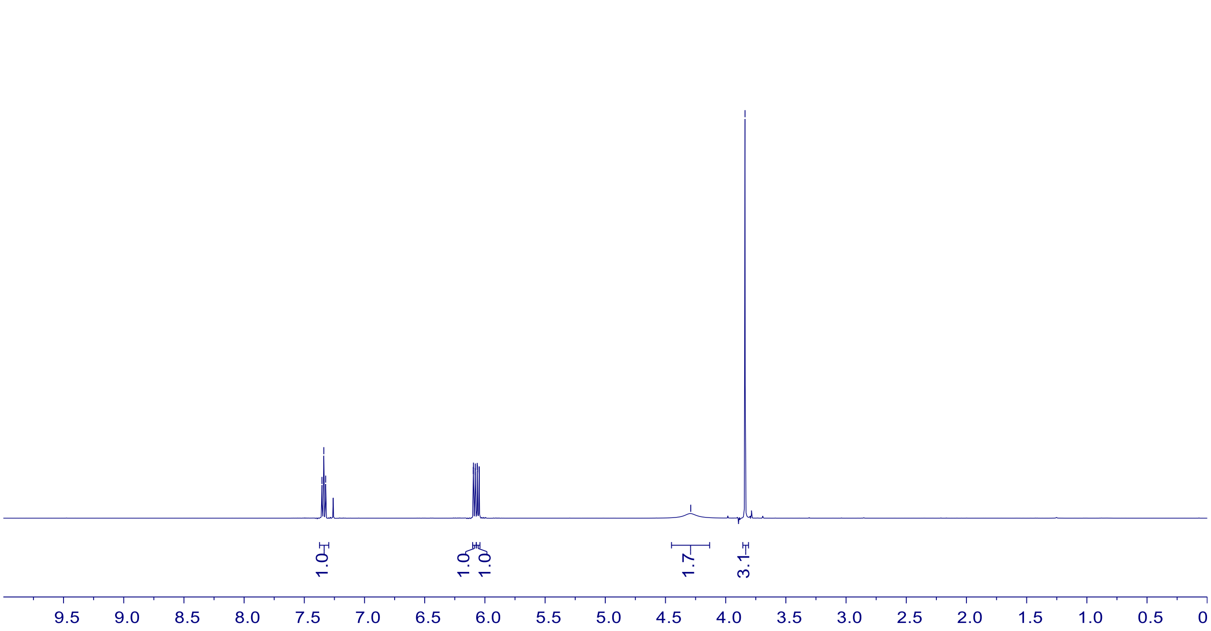

30 – ^{13}C NMR (126 MHz, CDCl$_3$)

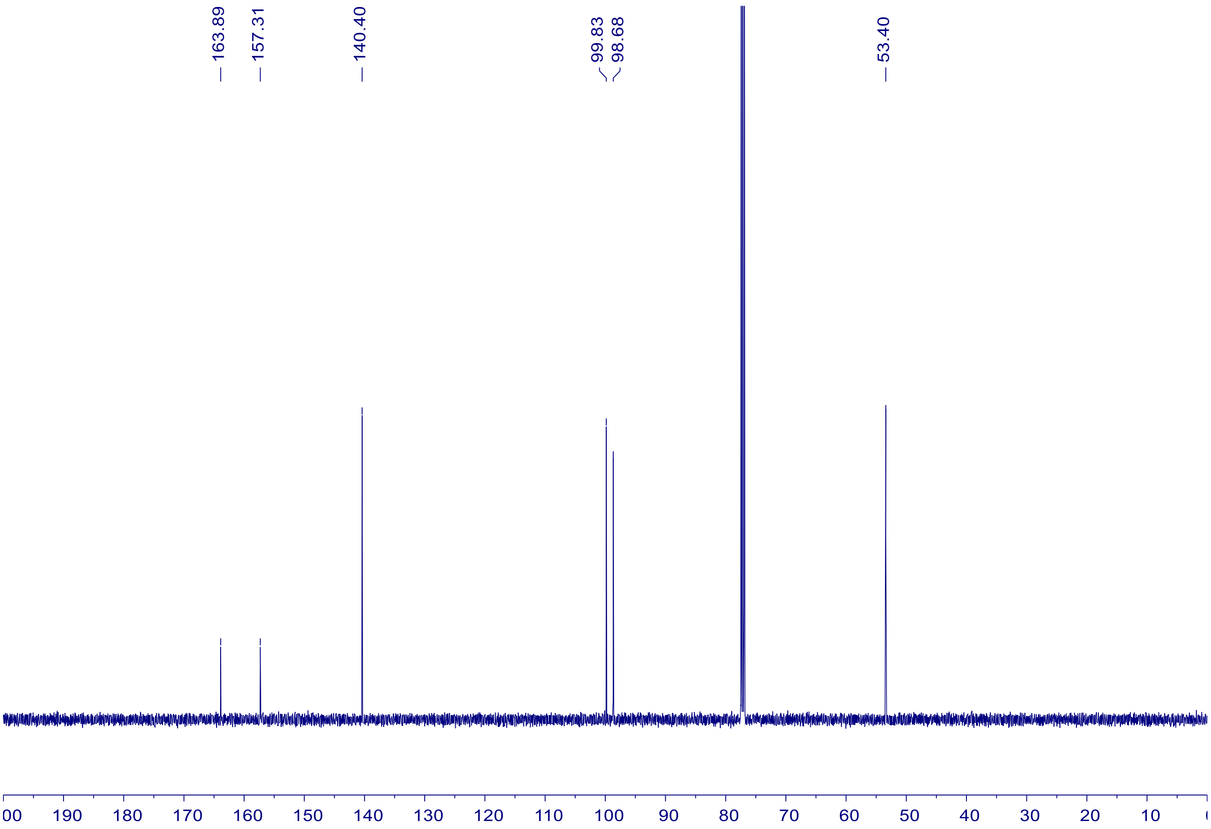

31 – ^{1}H NMR (500 MHz, $CDCl_3$)

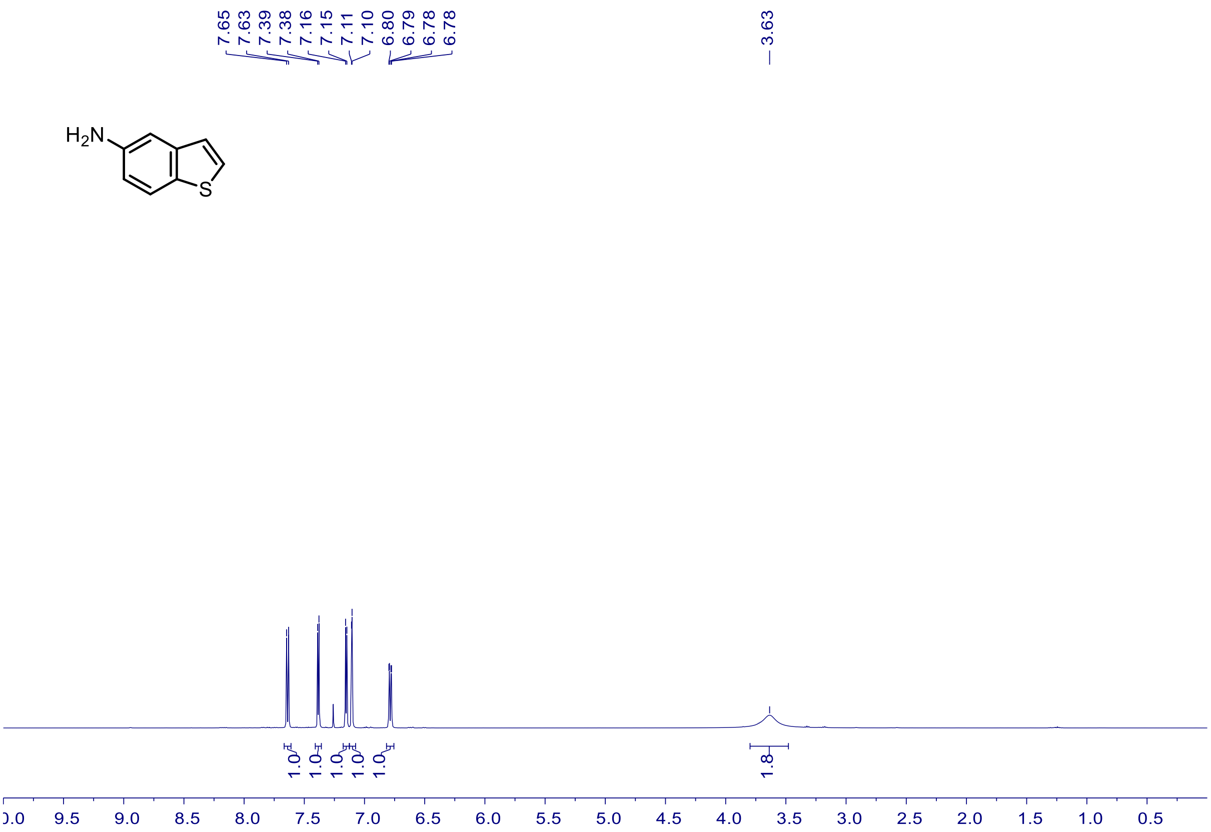

31 – ^{13}C NMR (126 MHz, $CDCl_3$)

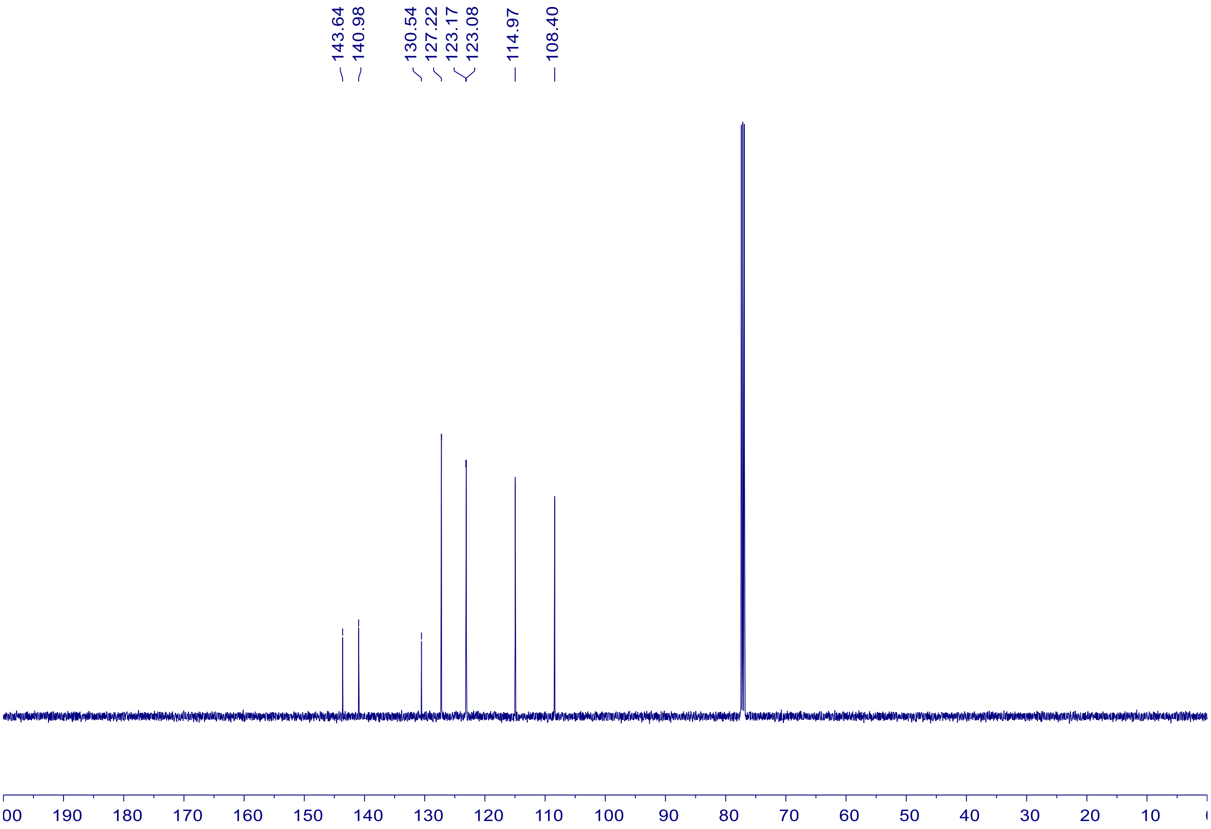

S7 – ^{1}H NMR (400 MHz, MeOH-d_4)

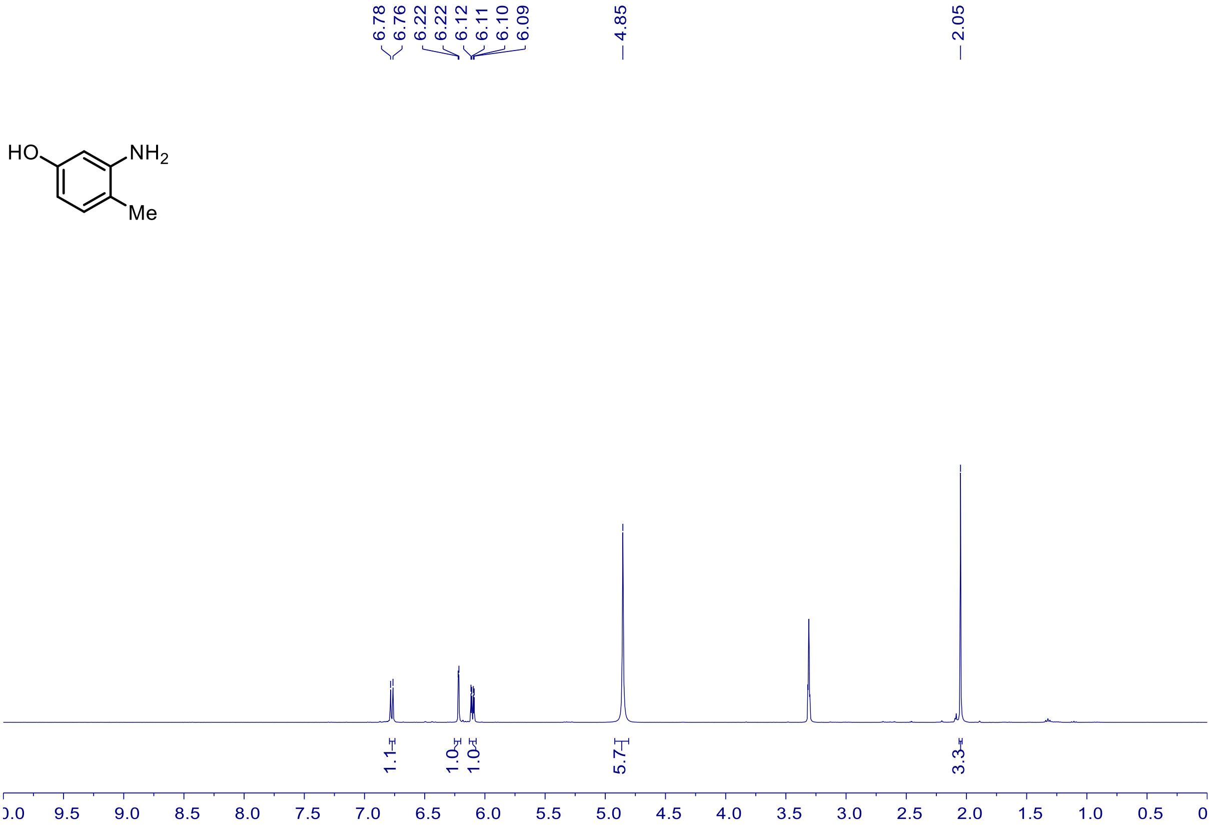

S7 – ^{13}C NMR (101 MHz, MeOH-d_4)

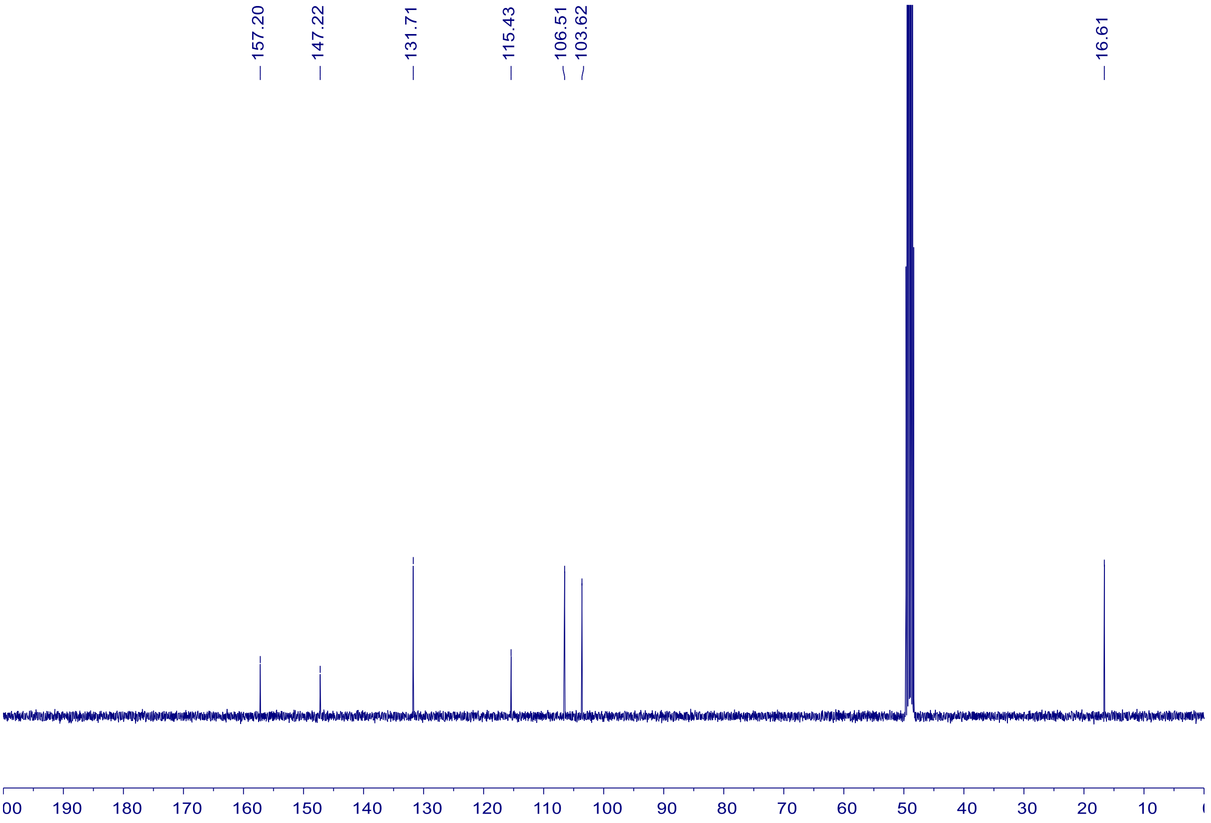

S8 – ^{1}H NMR (500 MHz, $CDCl_3$)

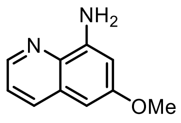

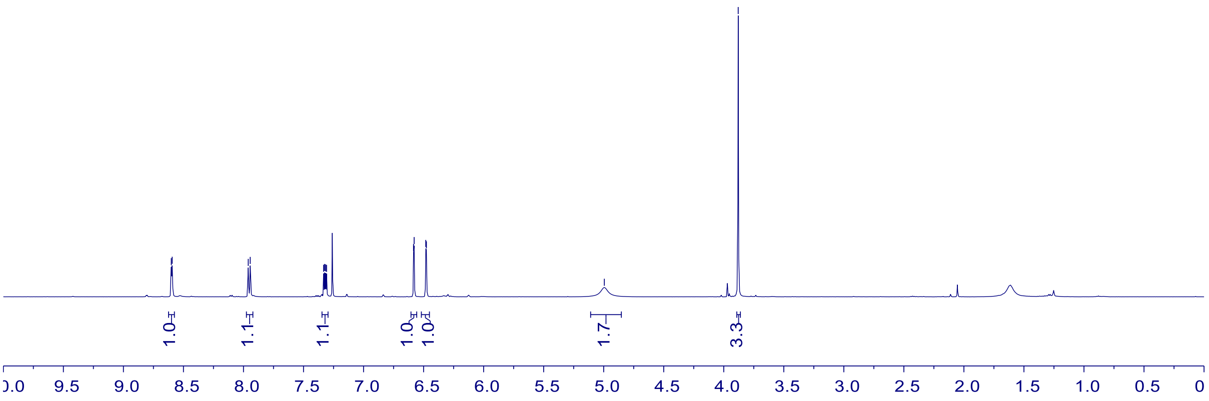

S8 – ^{13}C NMR (126 MHz, $CDCl_3$)

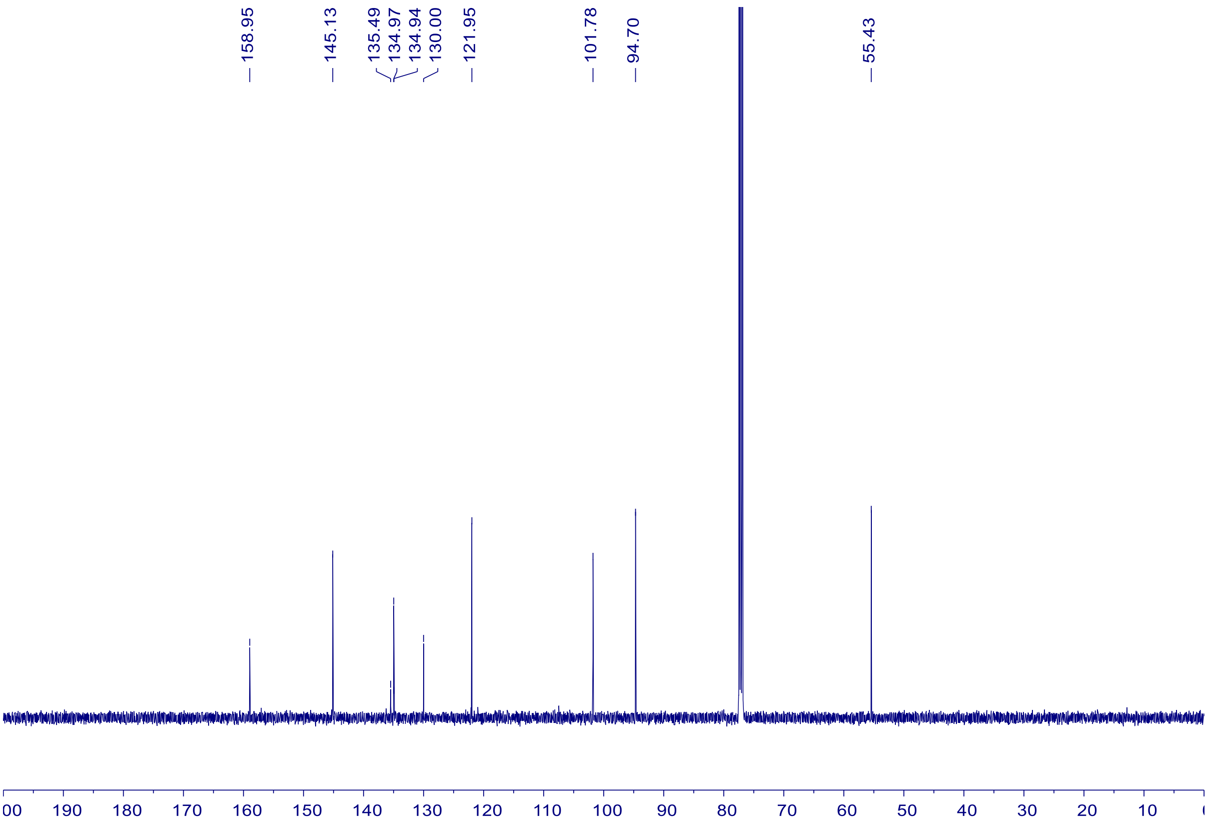

33 – ^{1}H NMR (500 MHz, $CDCl_3$)

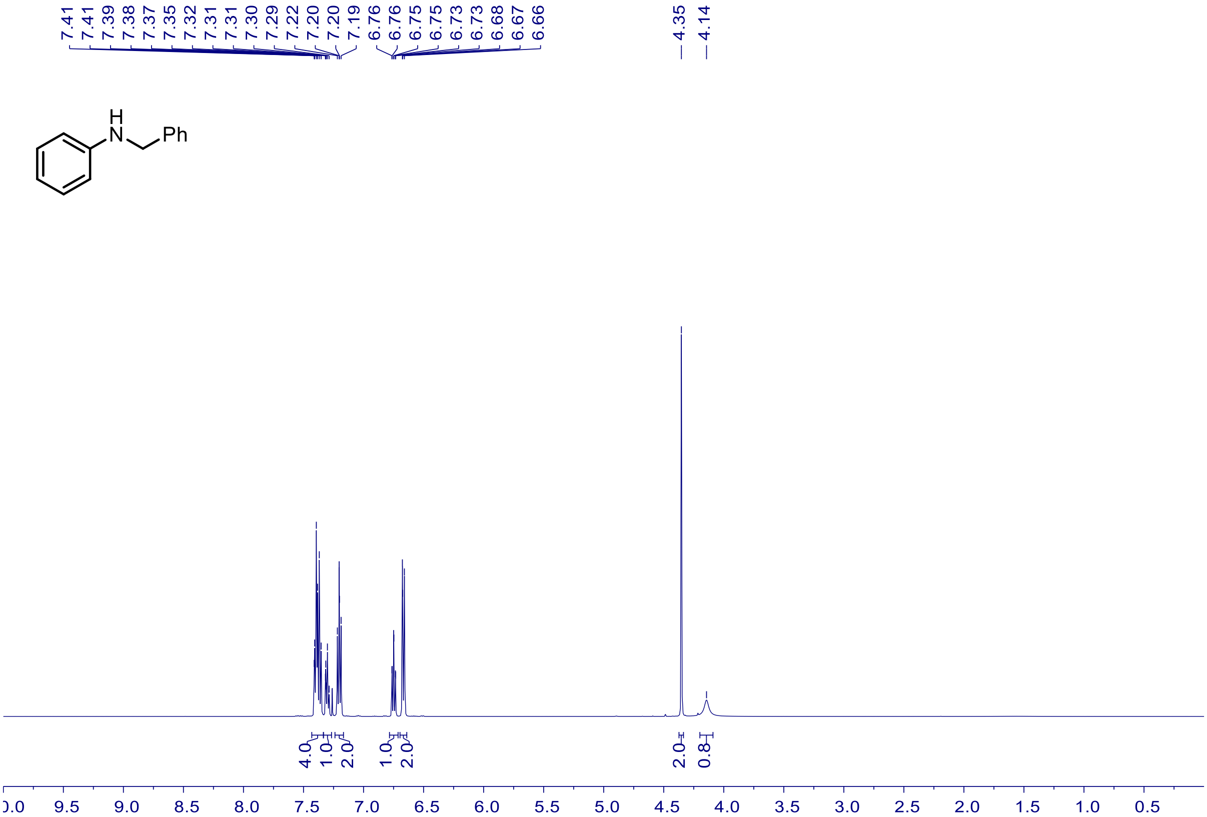

33 – ^{13}C NMR (126 MHz, $CDCl_3$)

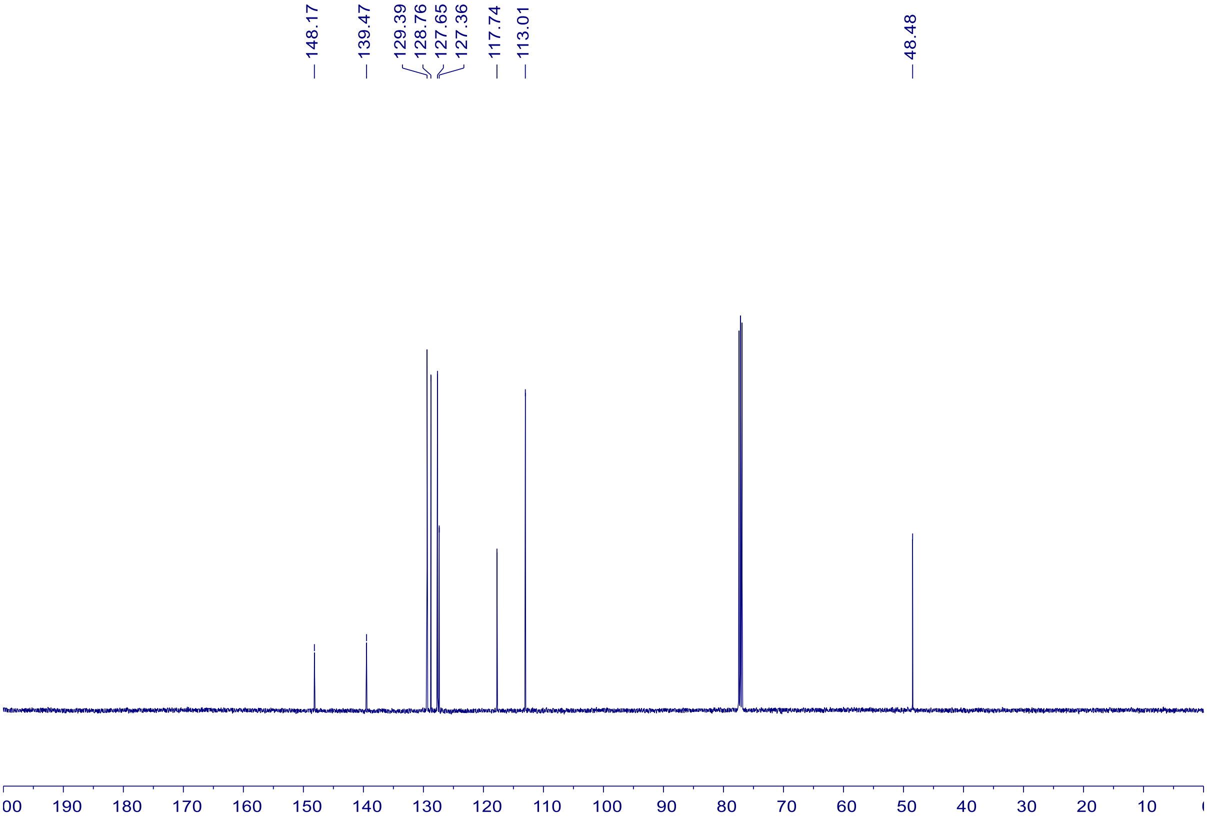

2 – ^{1}H NMR (500 MHz, CDCl$_3$)

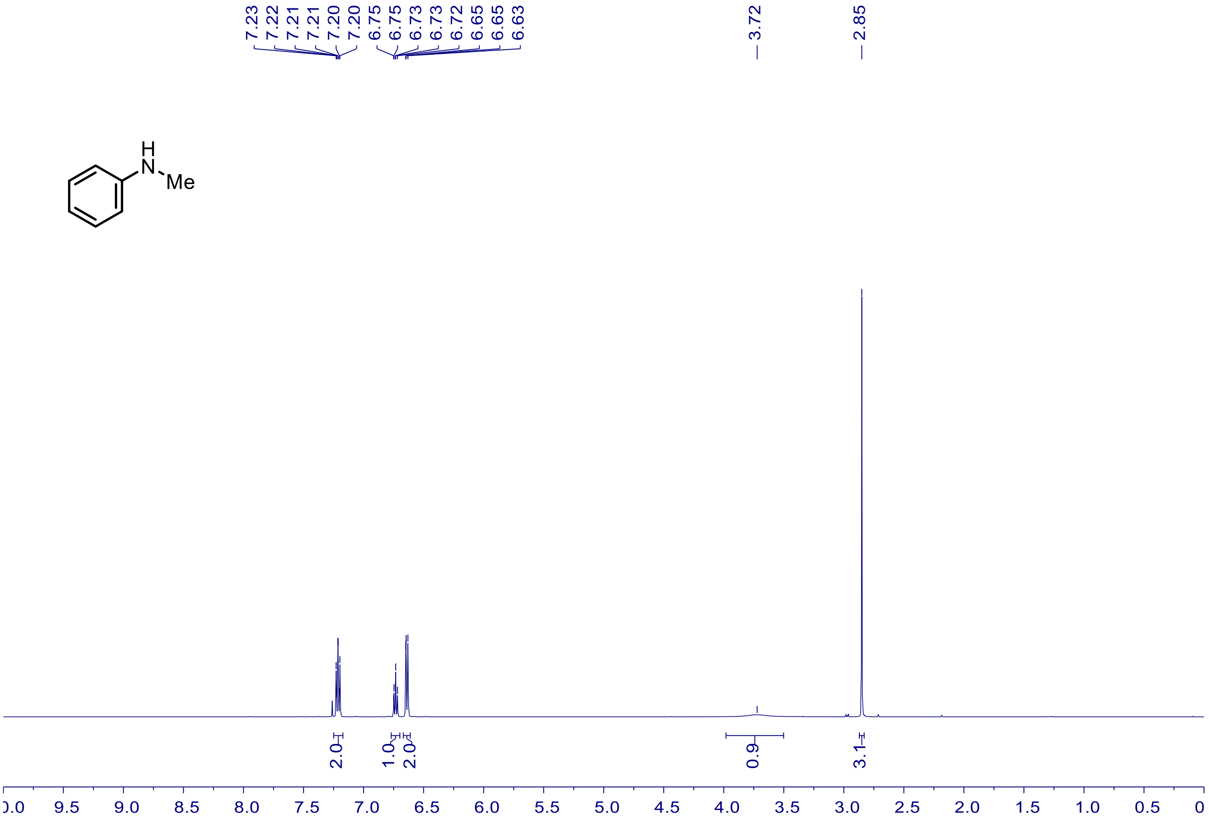

2 – ^{13}C NMR (126 MHz, CDCl$_3$)

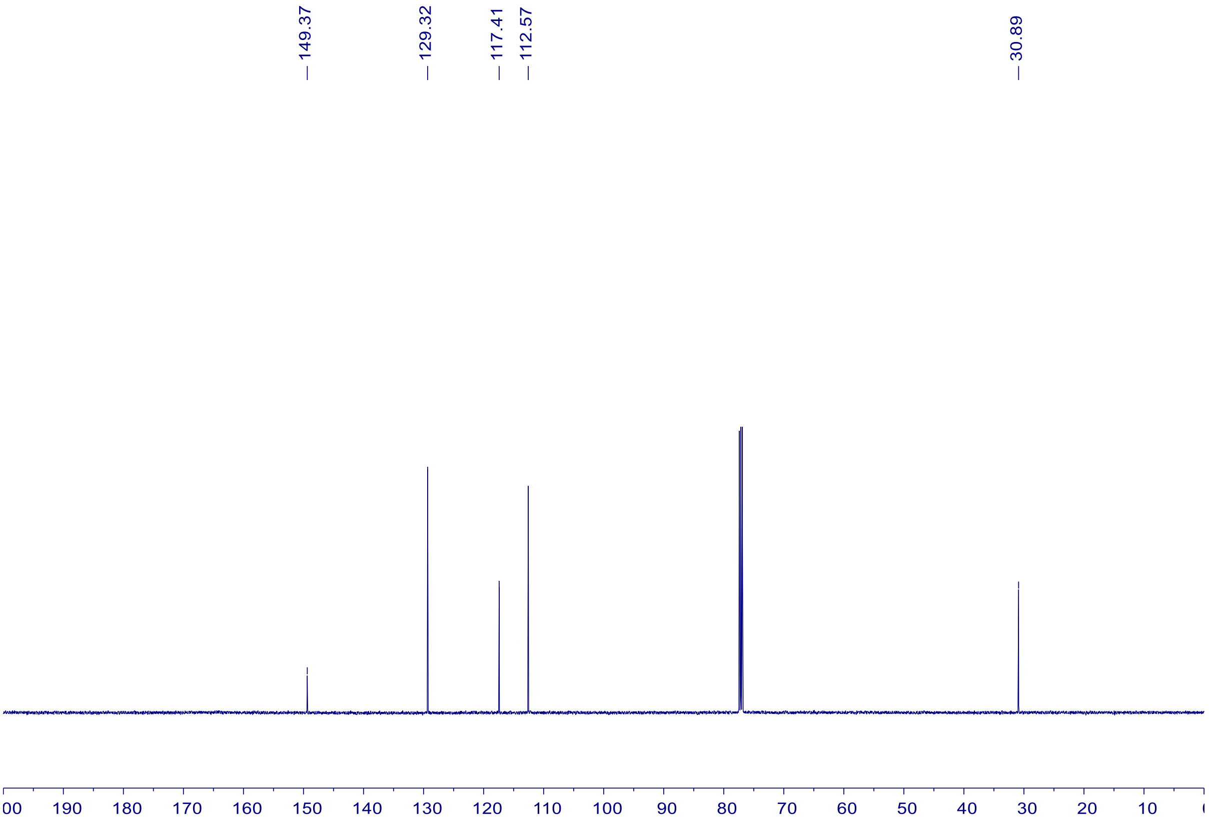

47 – ^{1}H NMR (500 MHz, $CDCl_3$)

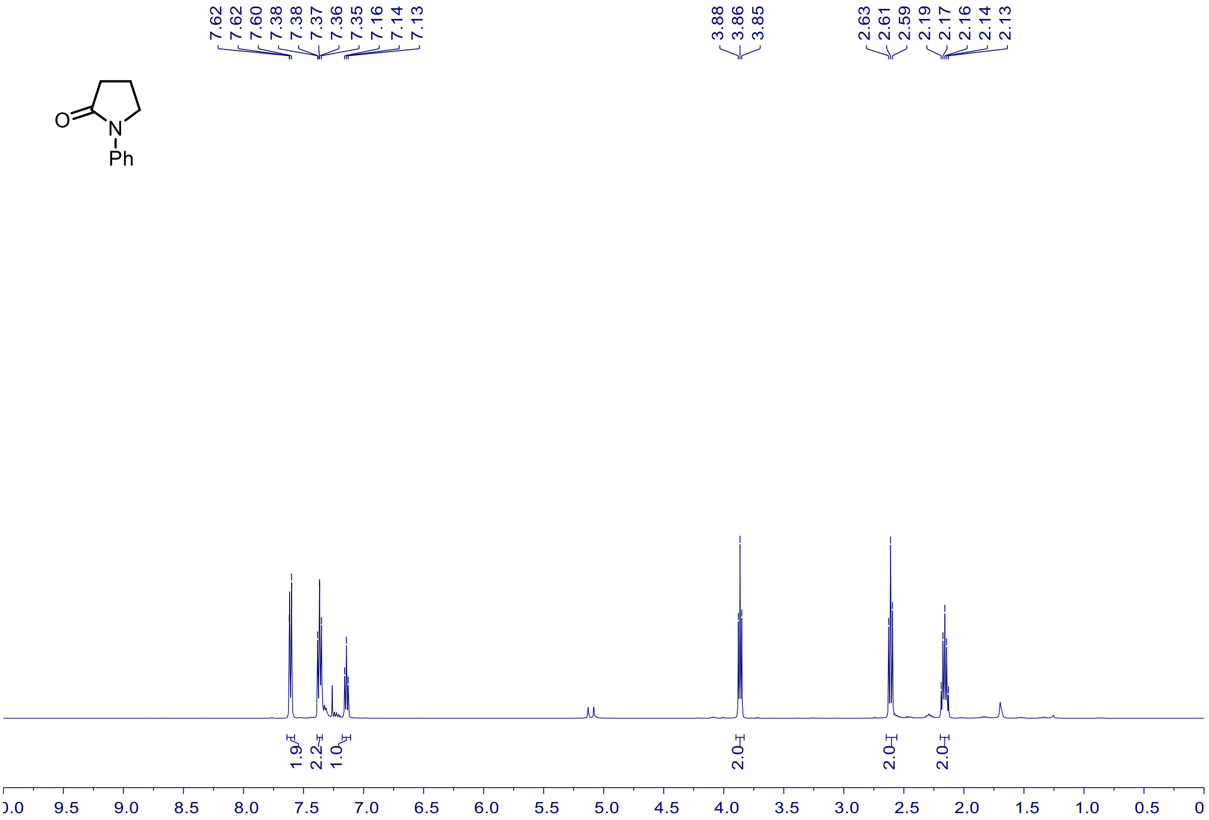

47 – ^{13}C NMR (126 MHz, $CDCl_3$)

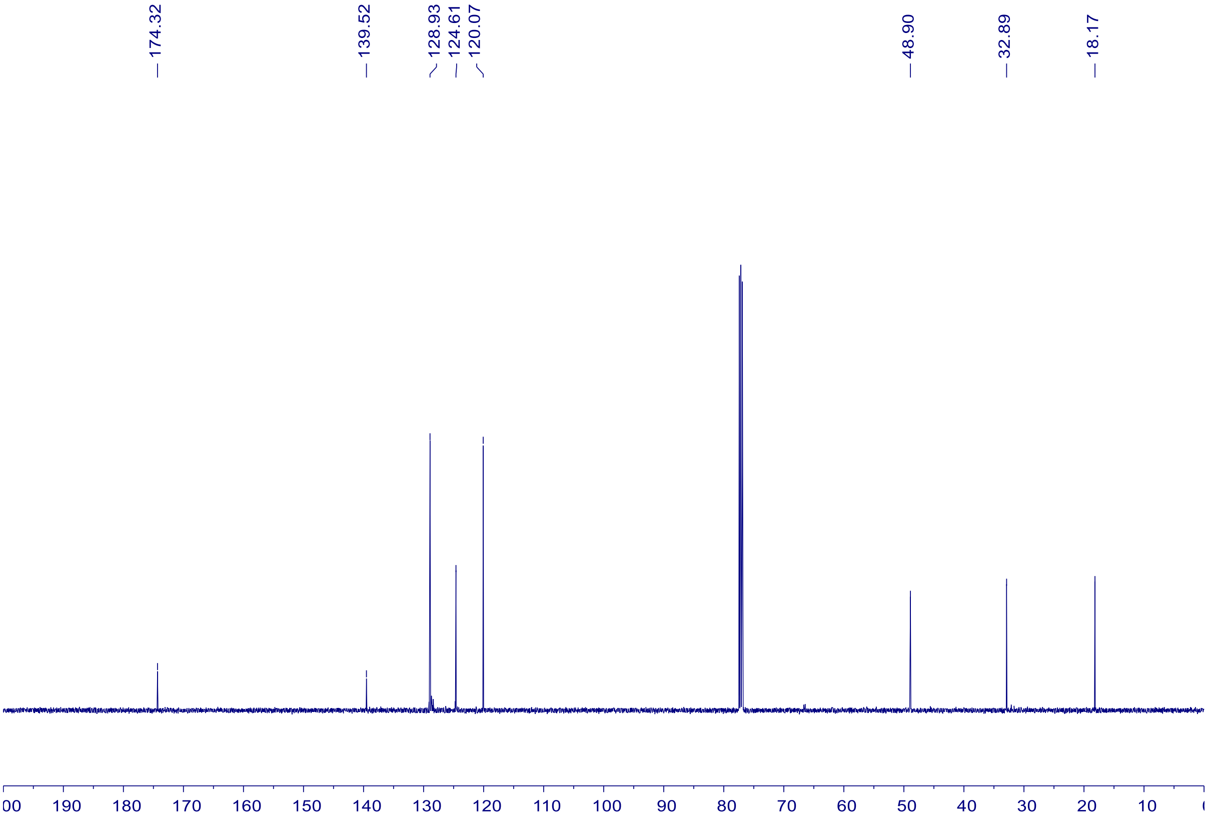

48 – ^{1}H NMR (500 MHz, $CDCl_3$)

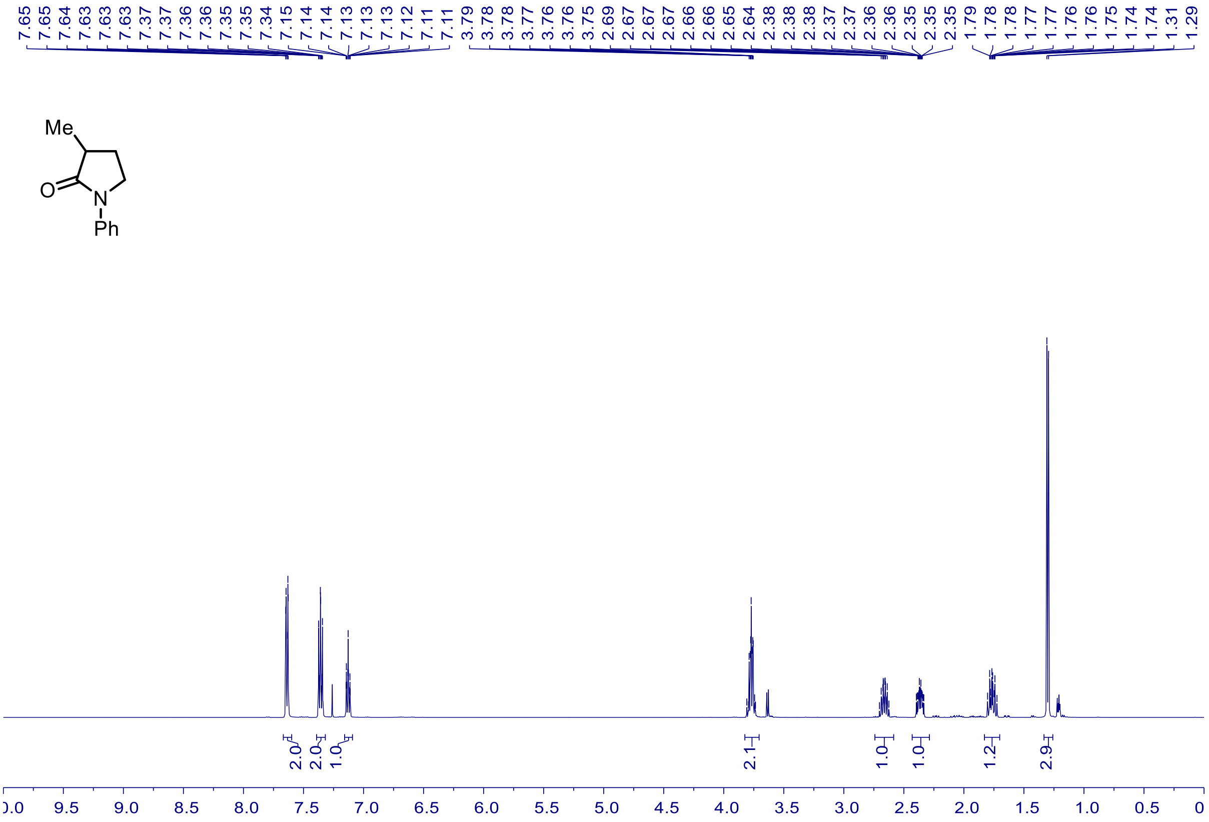

48 – ^{13}C NMR (126 MHz, $CDCl_3$)

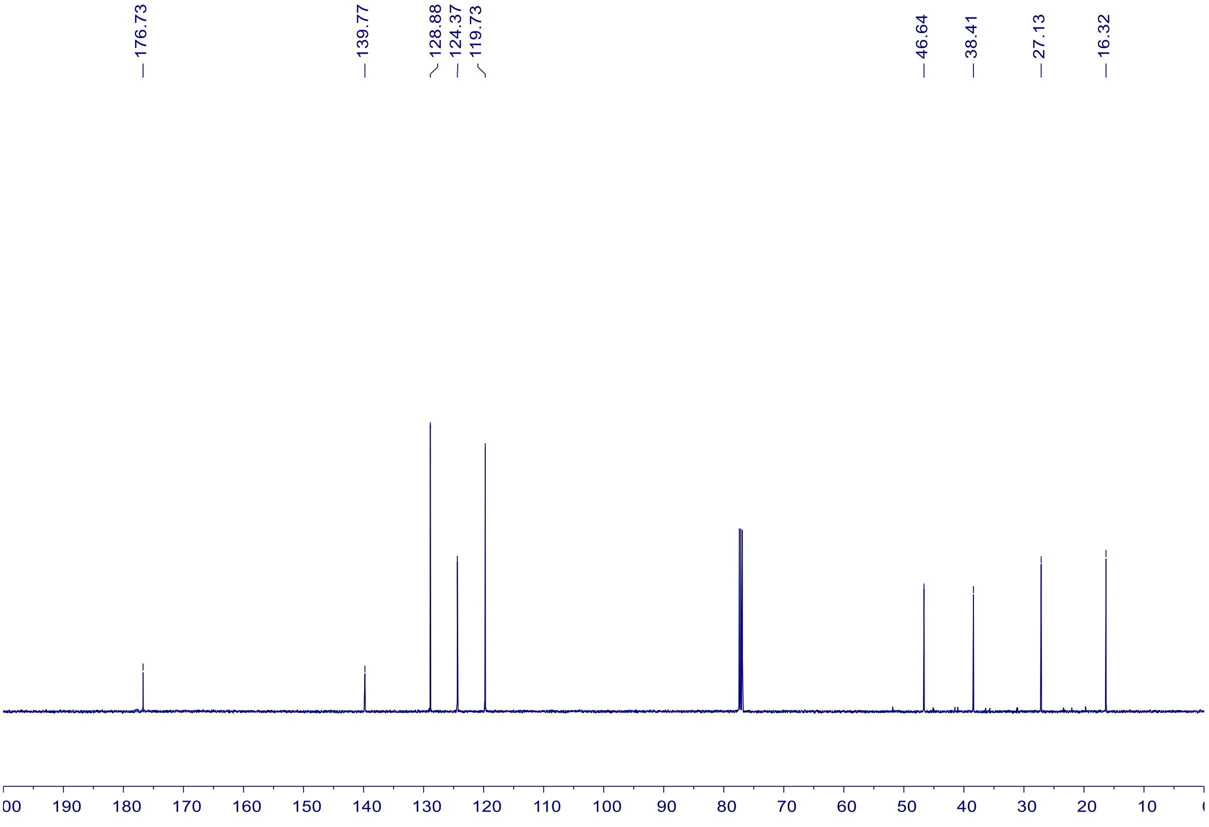

49 – ^{1}H NMR (500 MHz, CDCl$_3$)

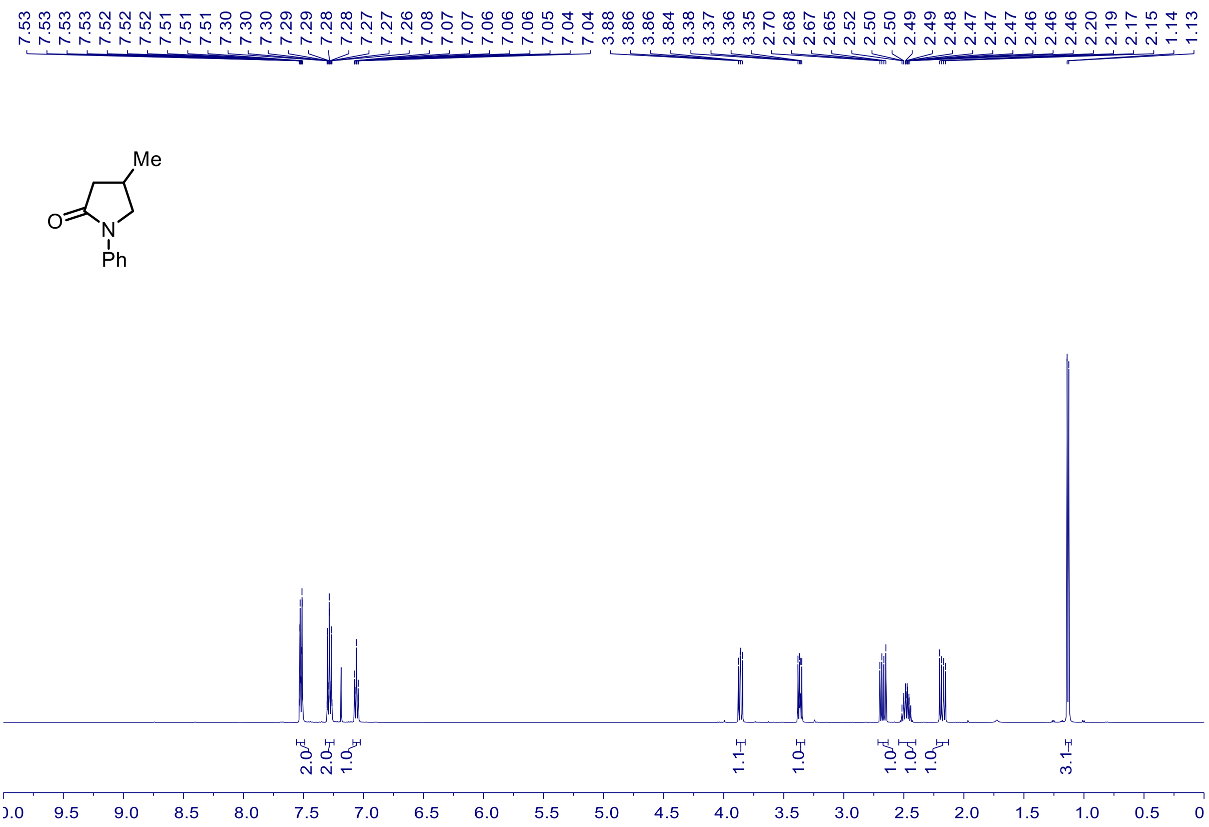

49 – ^{13}C NMR (126 MHz, CDCl$_3$)

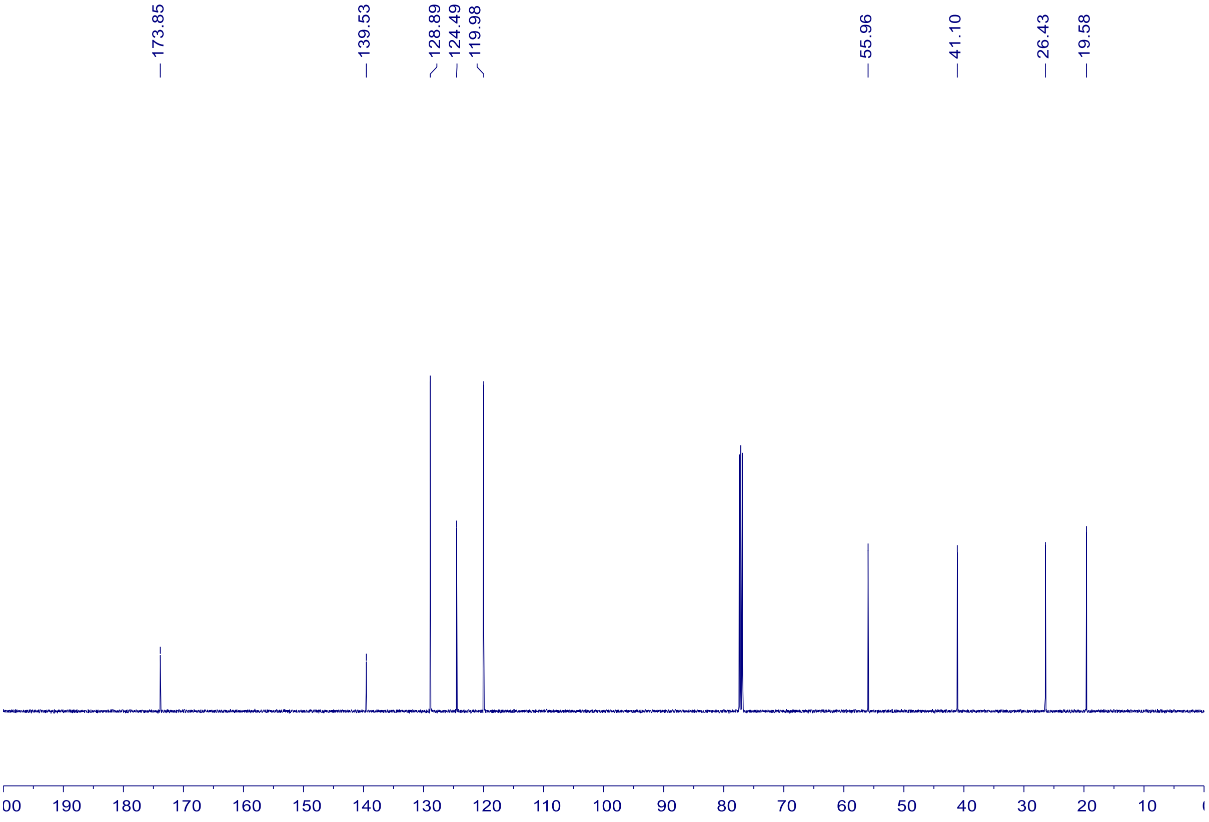

50 – ^{1}H NMR (400 MHz, CDCl$_3$)

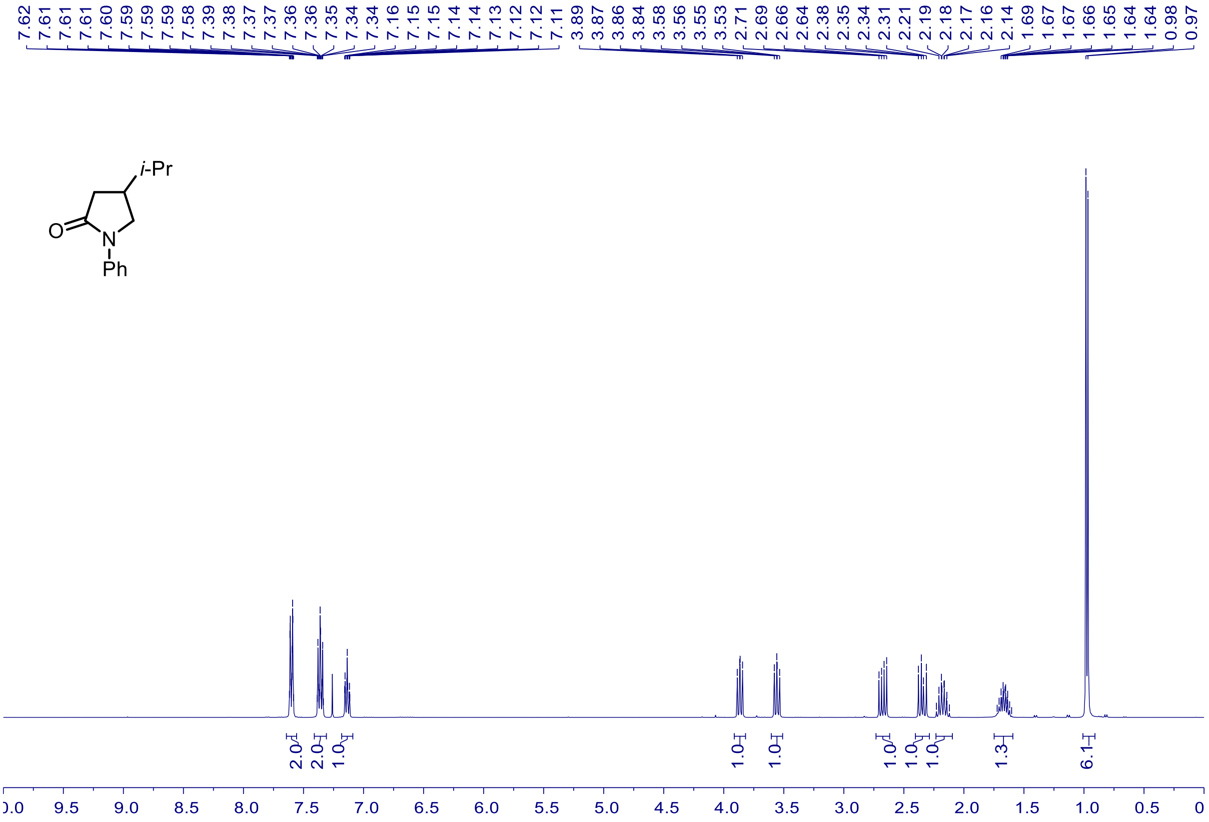

50 – ^{13}C NMR (126 MHz, CDCl$_3$)

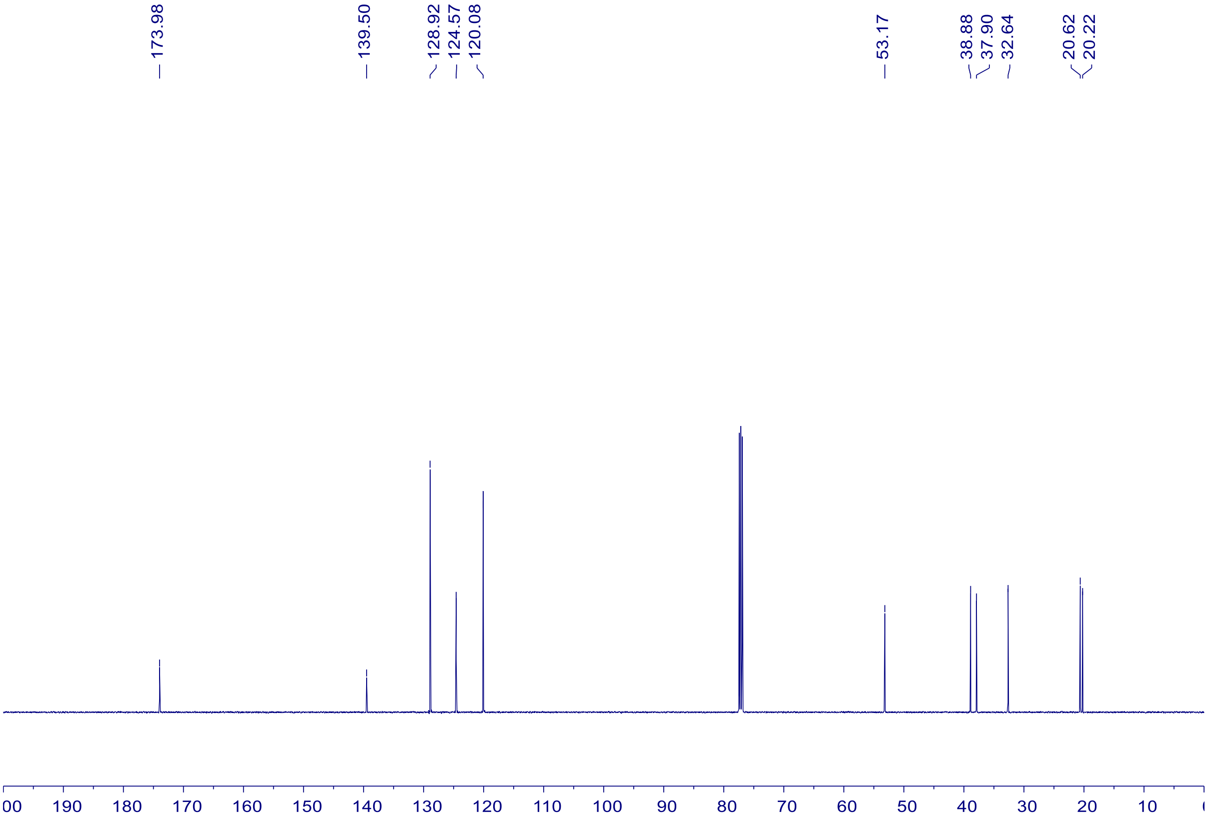

51 – ^{1}H NMR (400 MHz, $CDCl_3$)

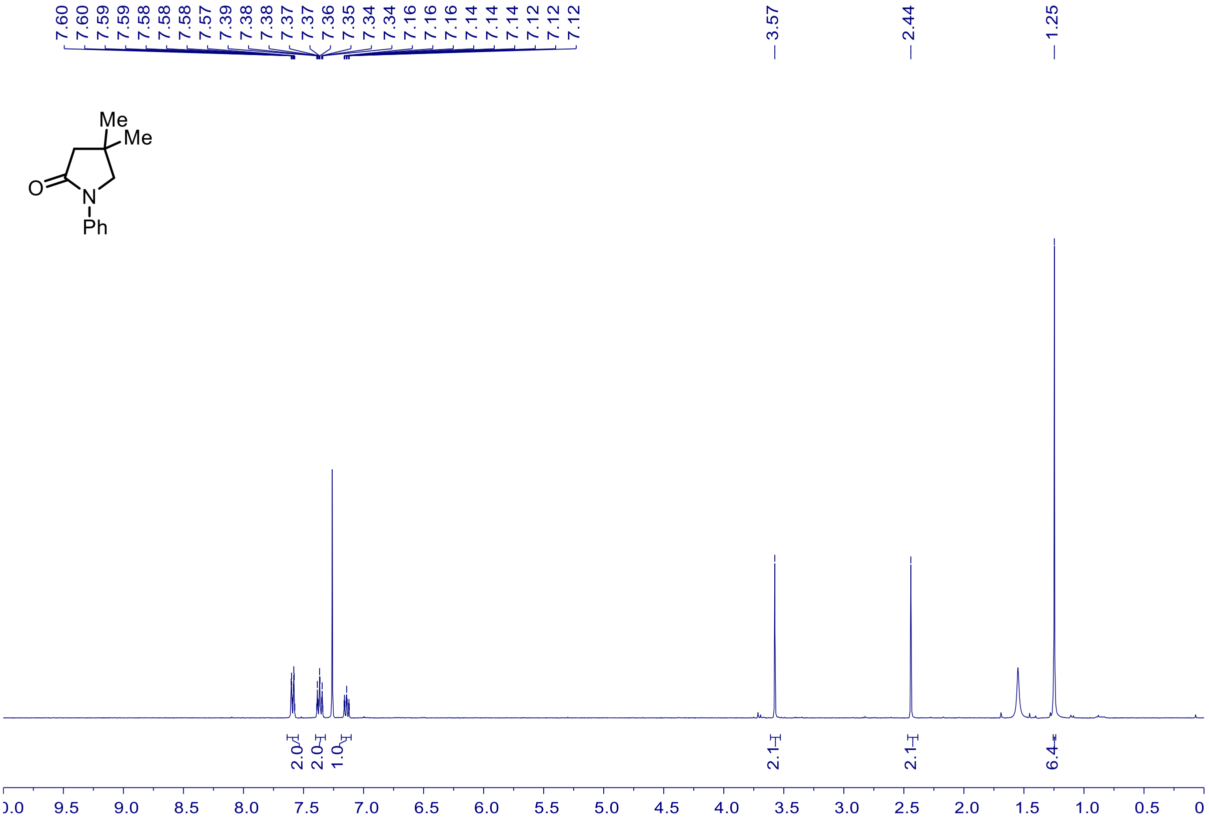

51 – ^{13}C NMR (126 MHz, $CDCl_3$)

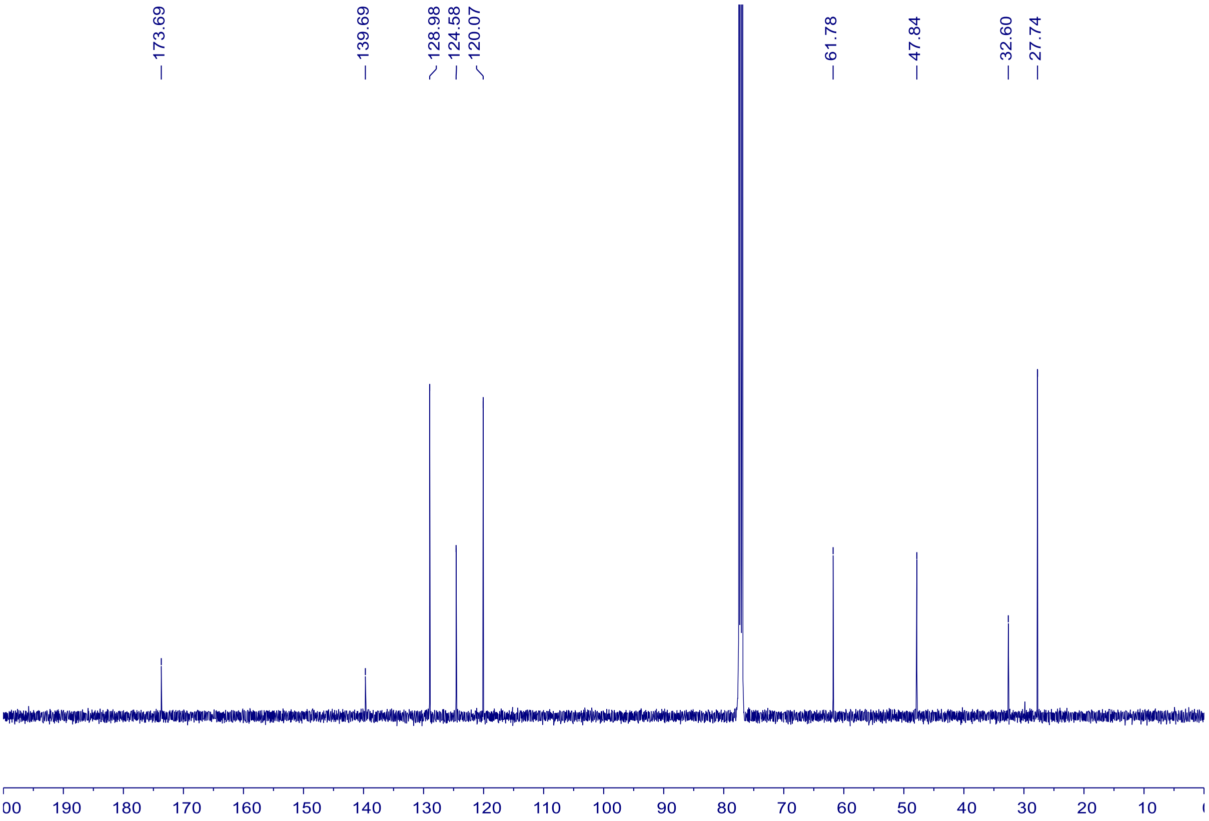

52 – ^{1}H NMR (500 MHz, $CDCl_3$)

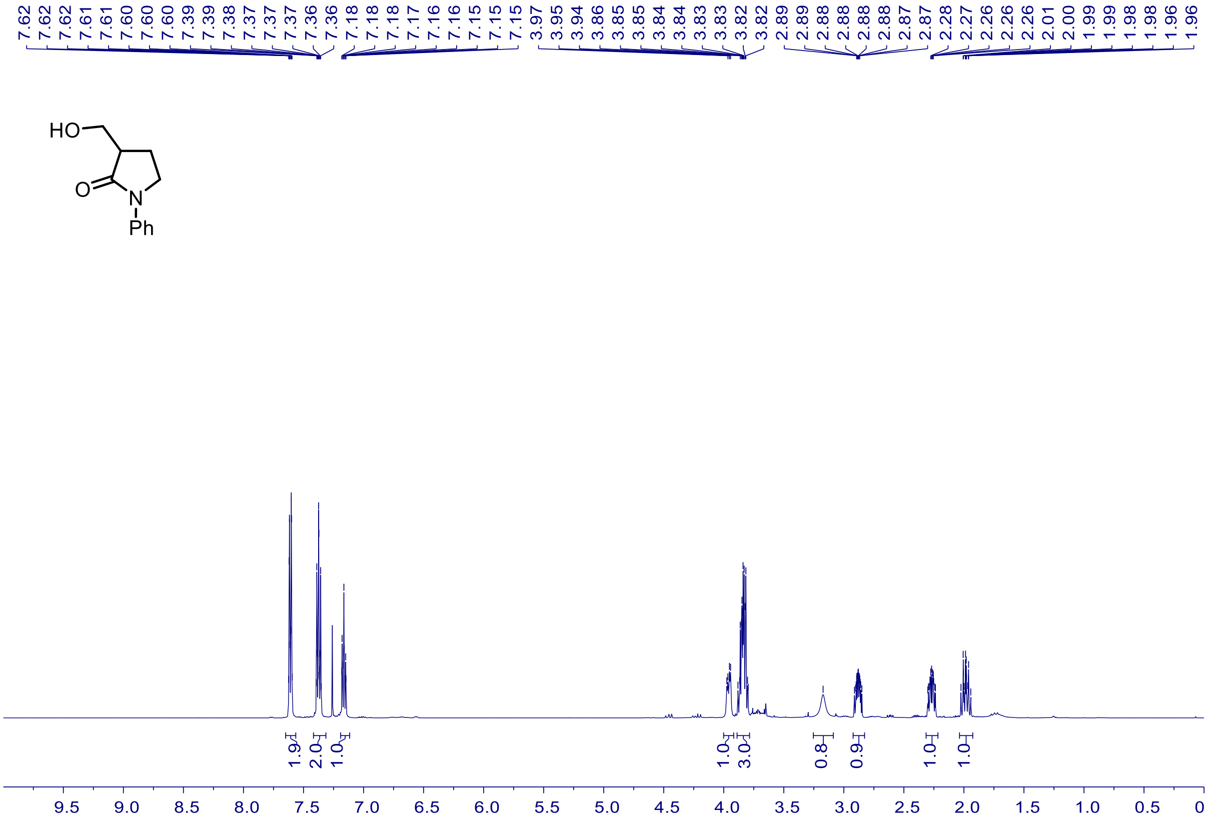

52 – ^{13}C NMR (126 MHz, $CDCl_3$)

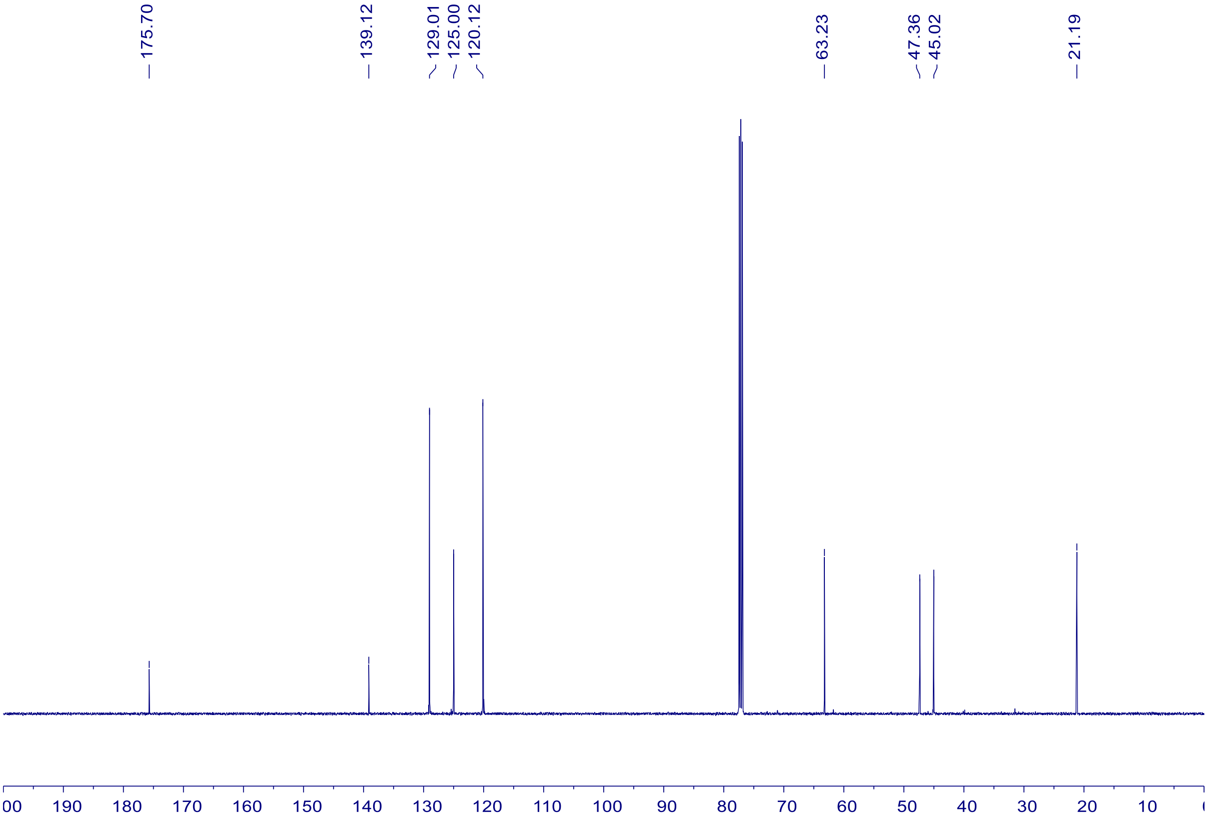

53 – ^{1}H NMR (500 MHz, $CDCl_3$)

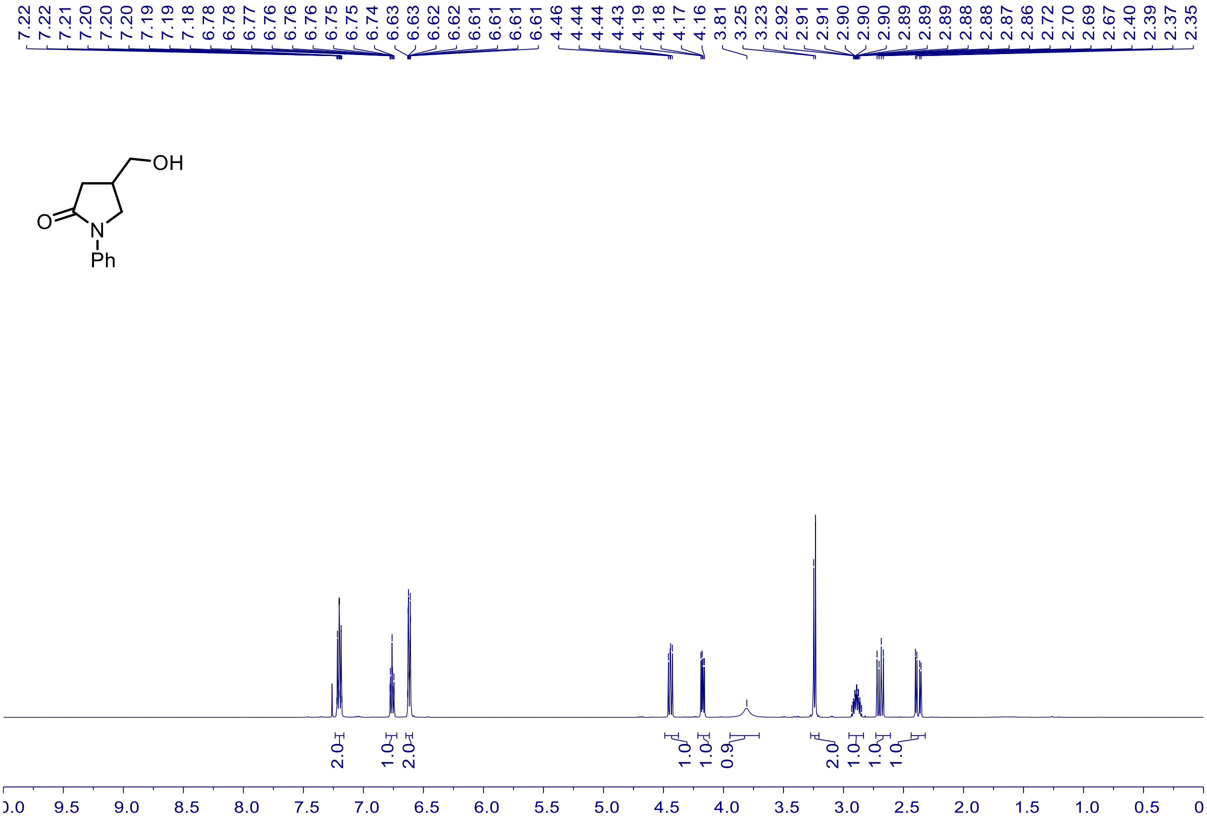

53 – ^{13}C NMR (126 MHz, $CDCl_3$)

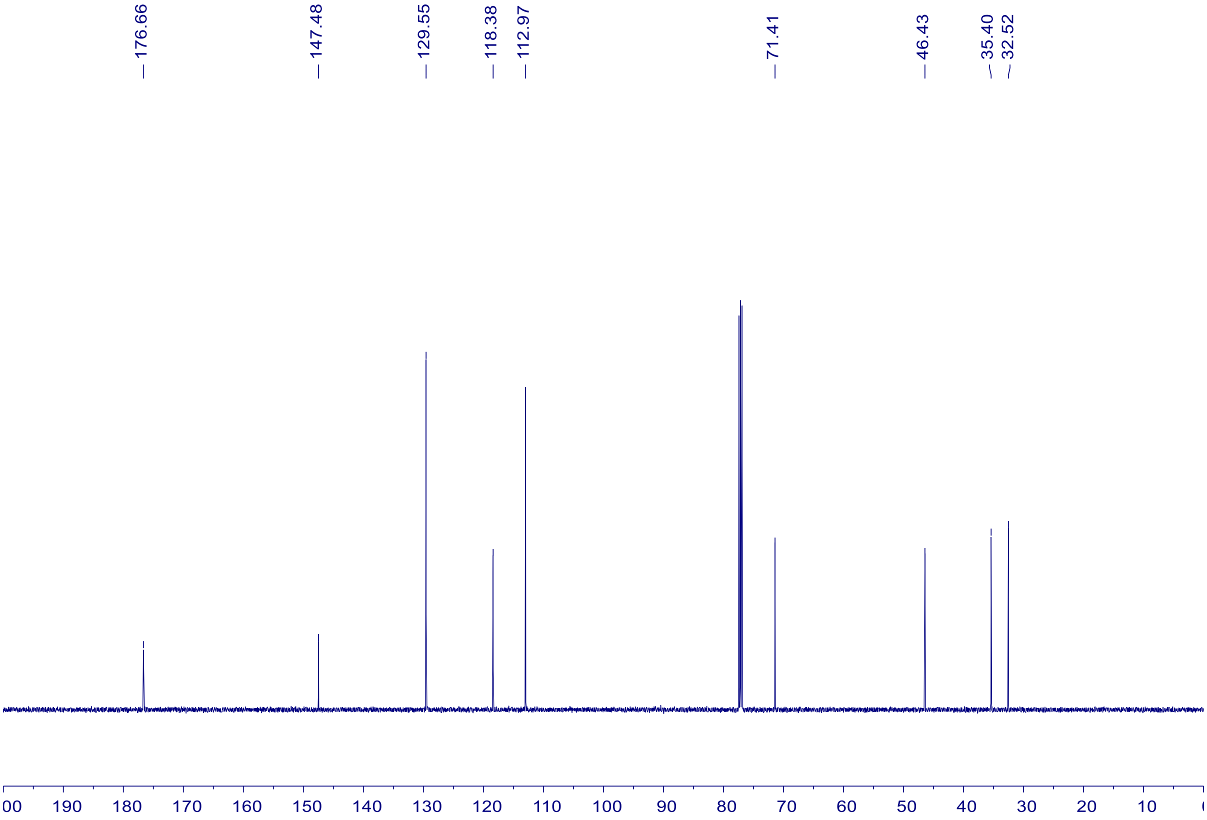

54 – ^{1}H NMR (500 MHz, $CDCl_3$)

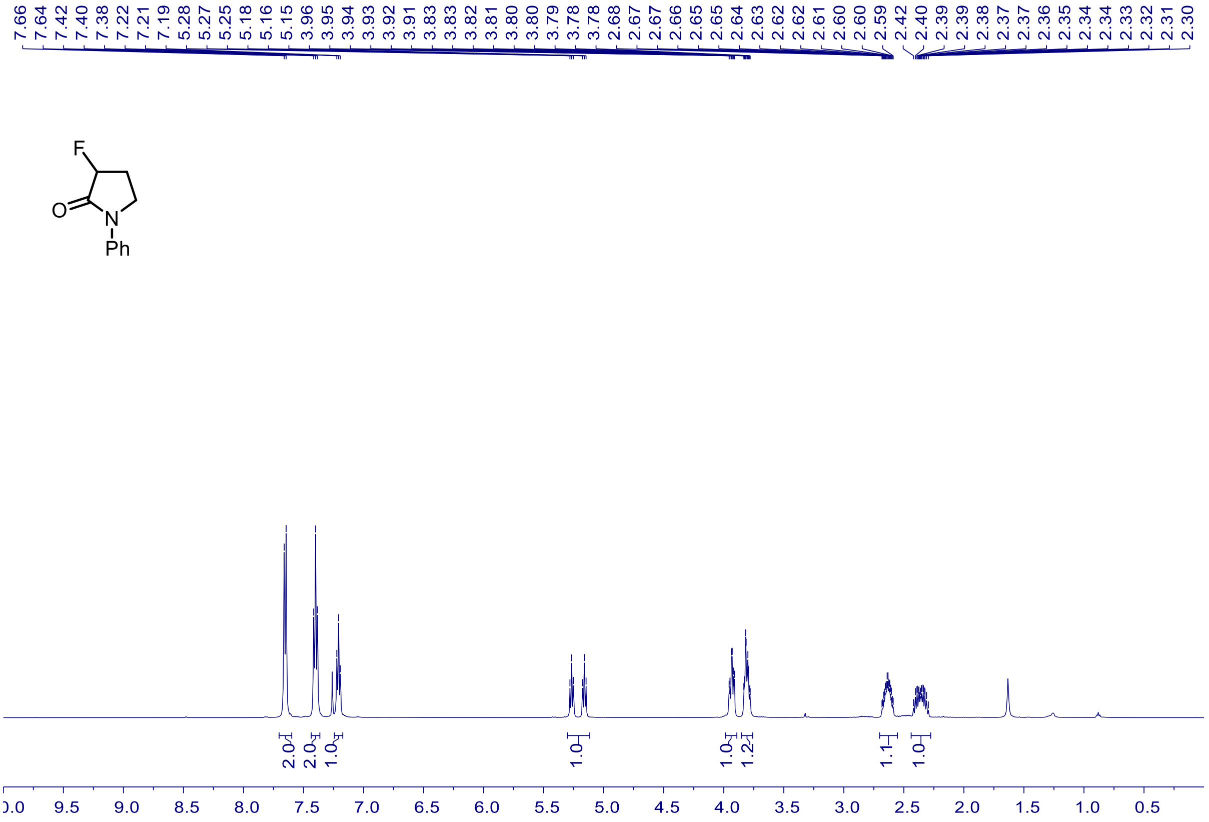

54 – ^{13}C NMR (126 MHz, $CDCl_3$)

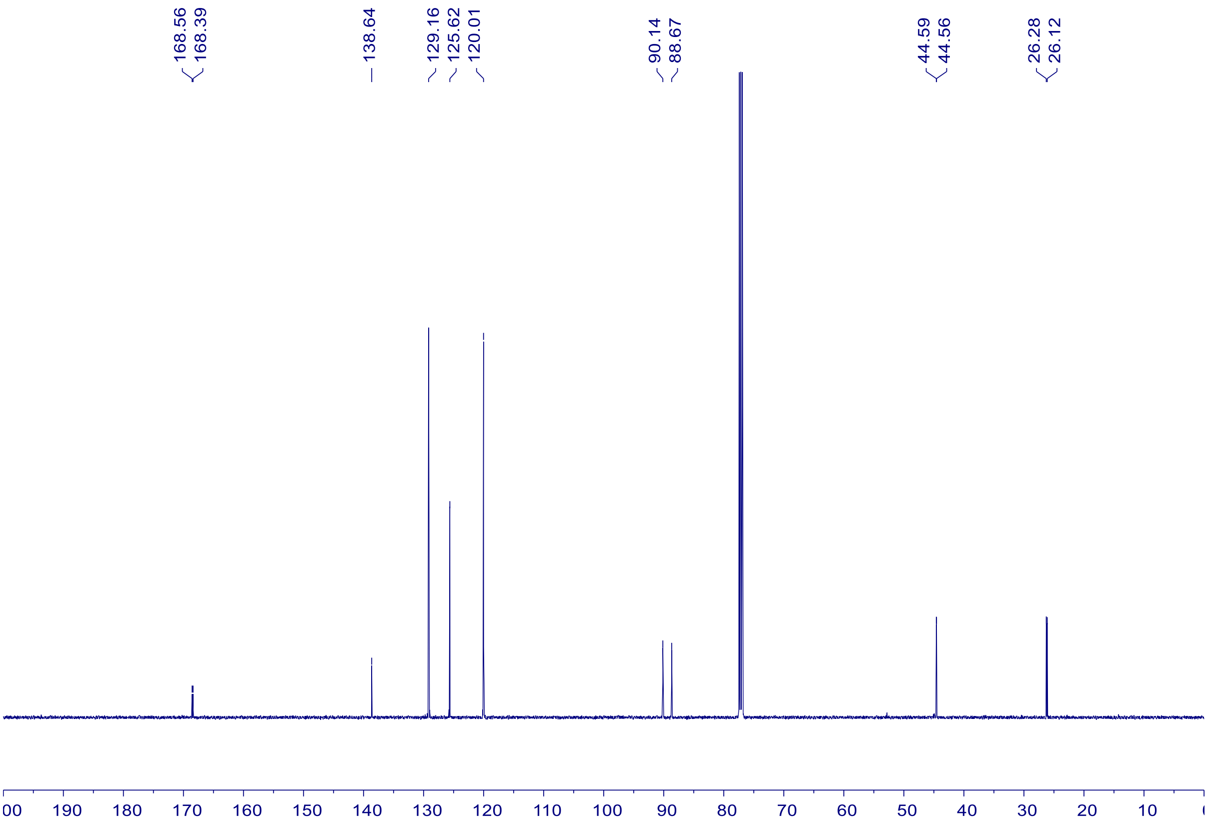

54 – ^{19}F NMR (376 MHz, $CDCl_3$)

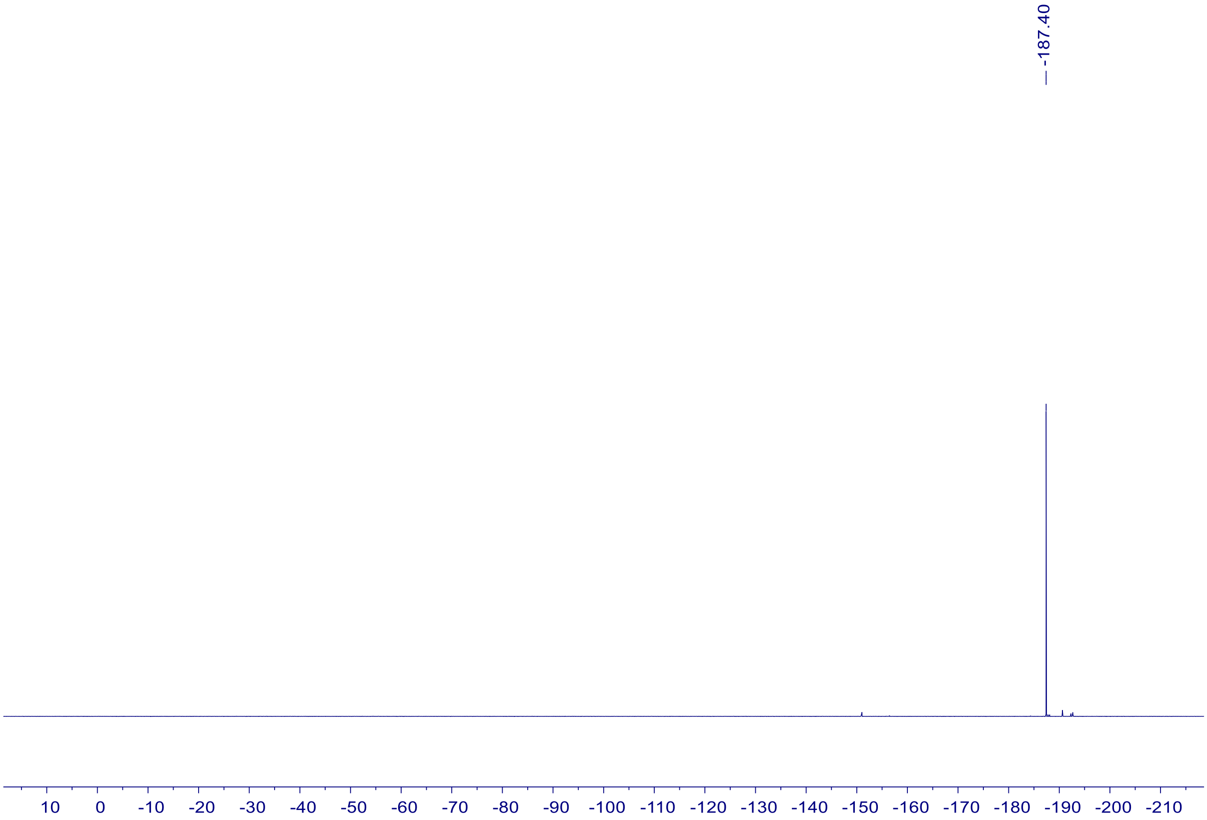

55 – ^{1}H NMR (400 MHz, $CDCl_3$)

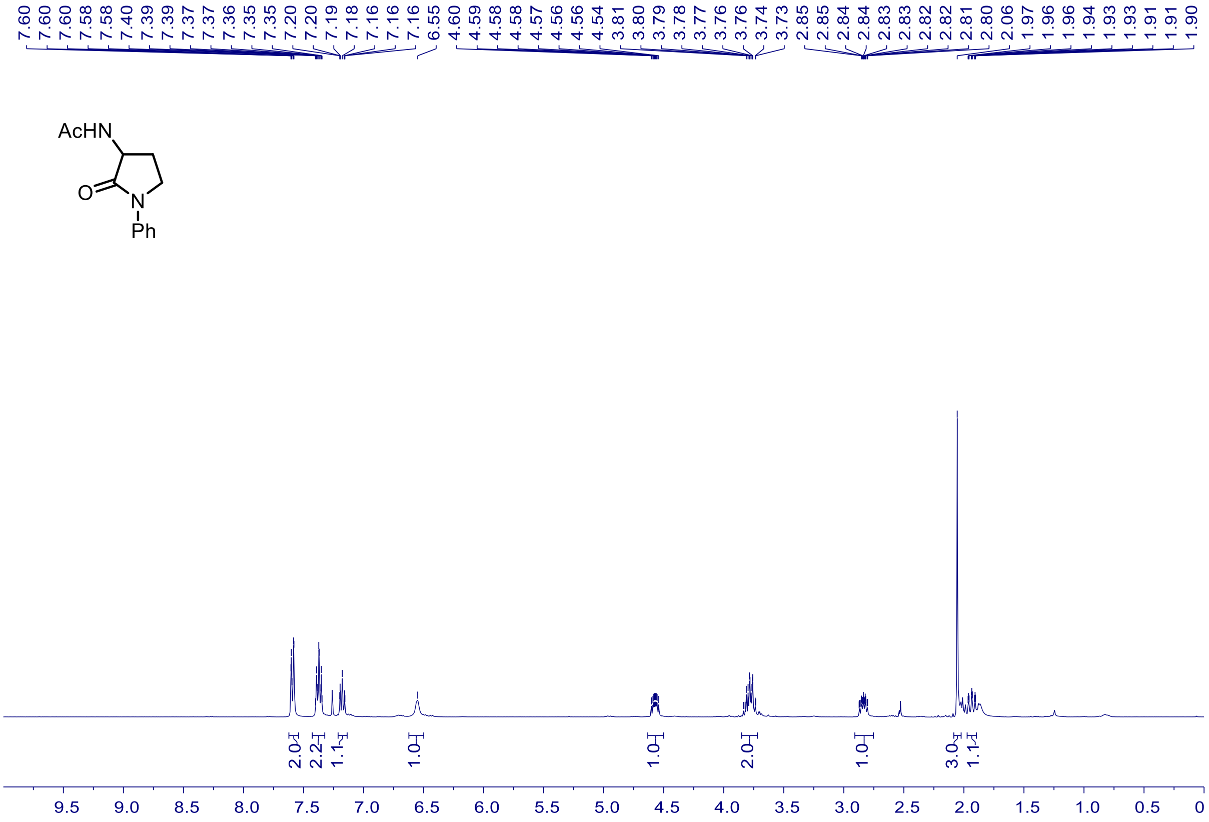

55 – ^{13}C NMR (101 MHz, $CDCl_3$)

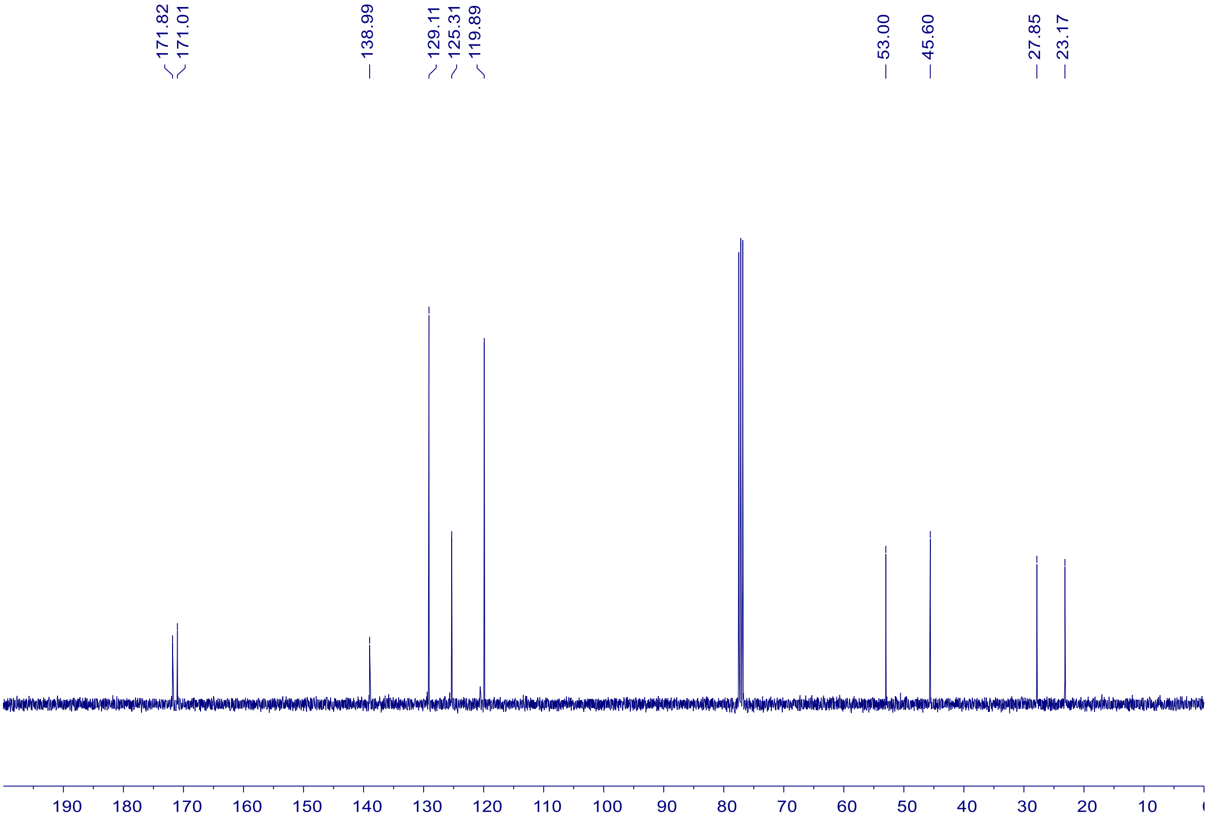

S9 – ^{1}H NMR (400 MHz, $CDCl_3$)

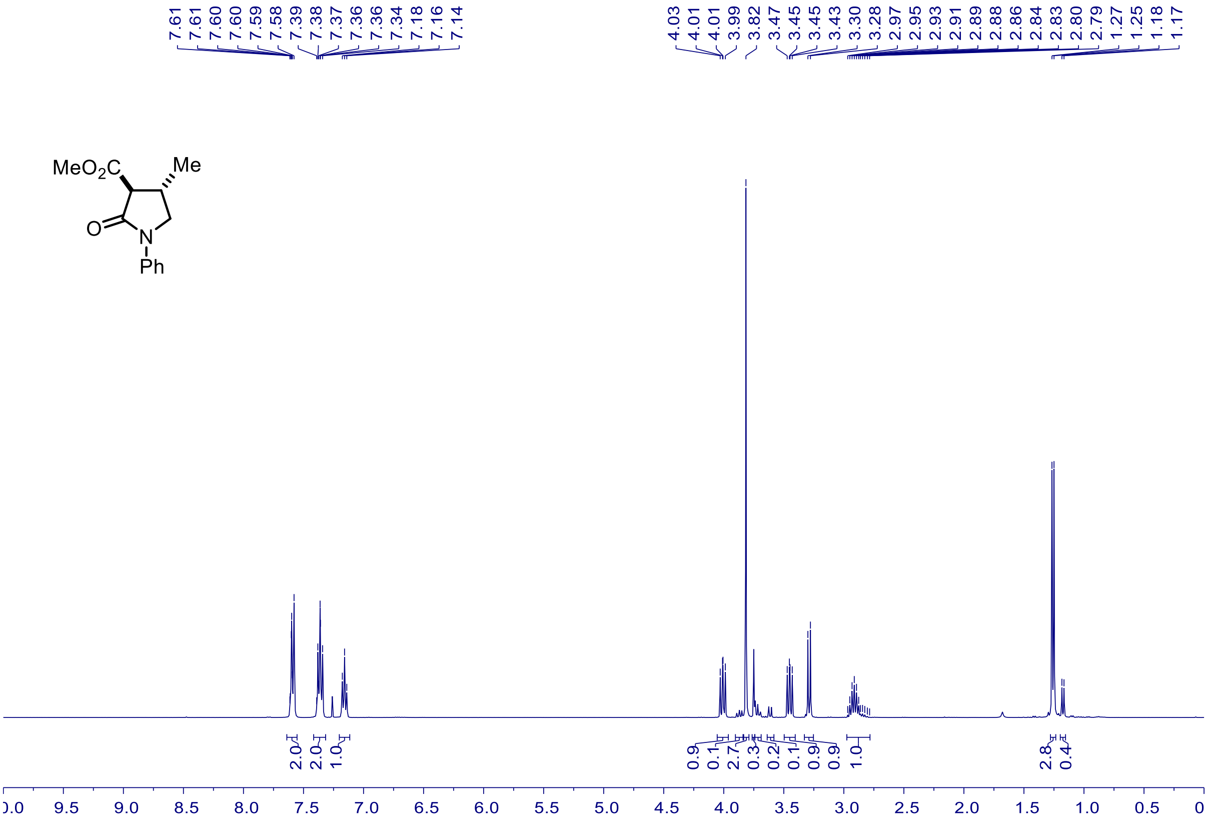

S9 – ^{13}C NMR (101 MHz, $CDCl_3$)

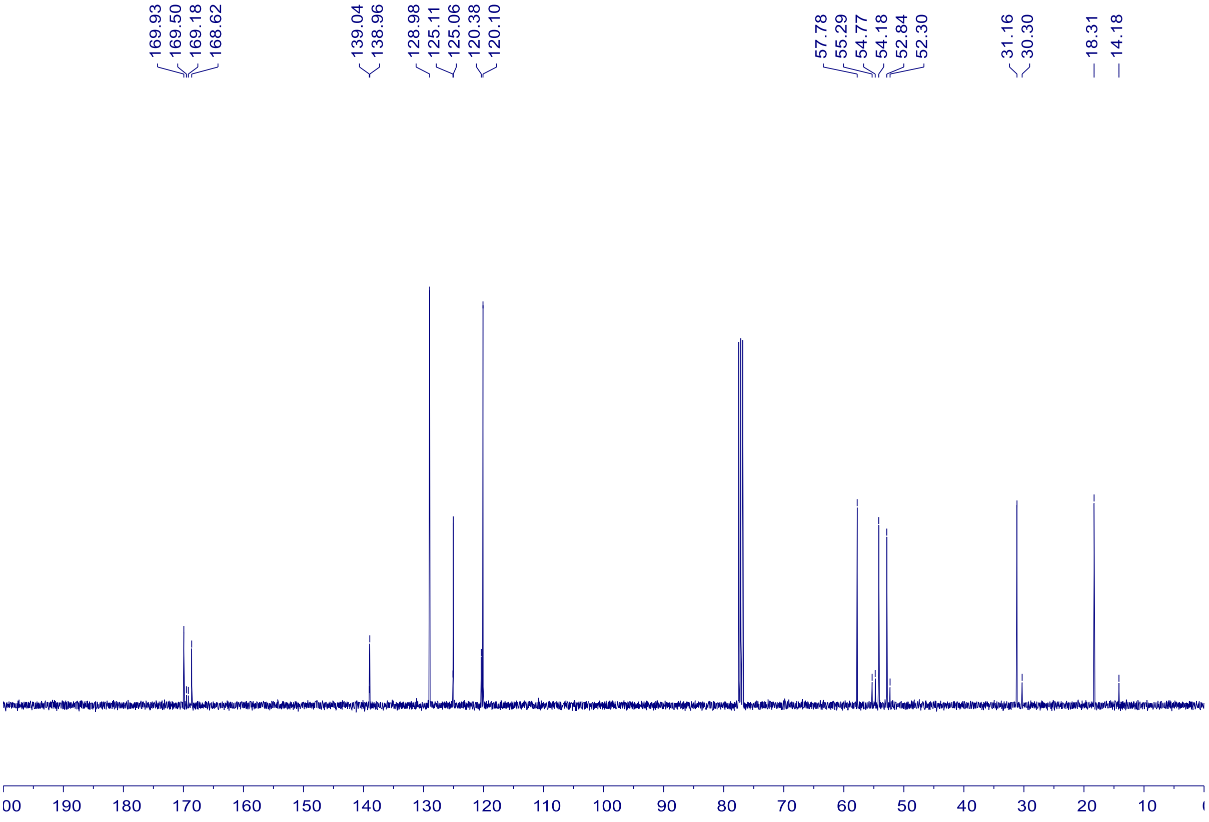

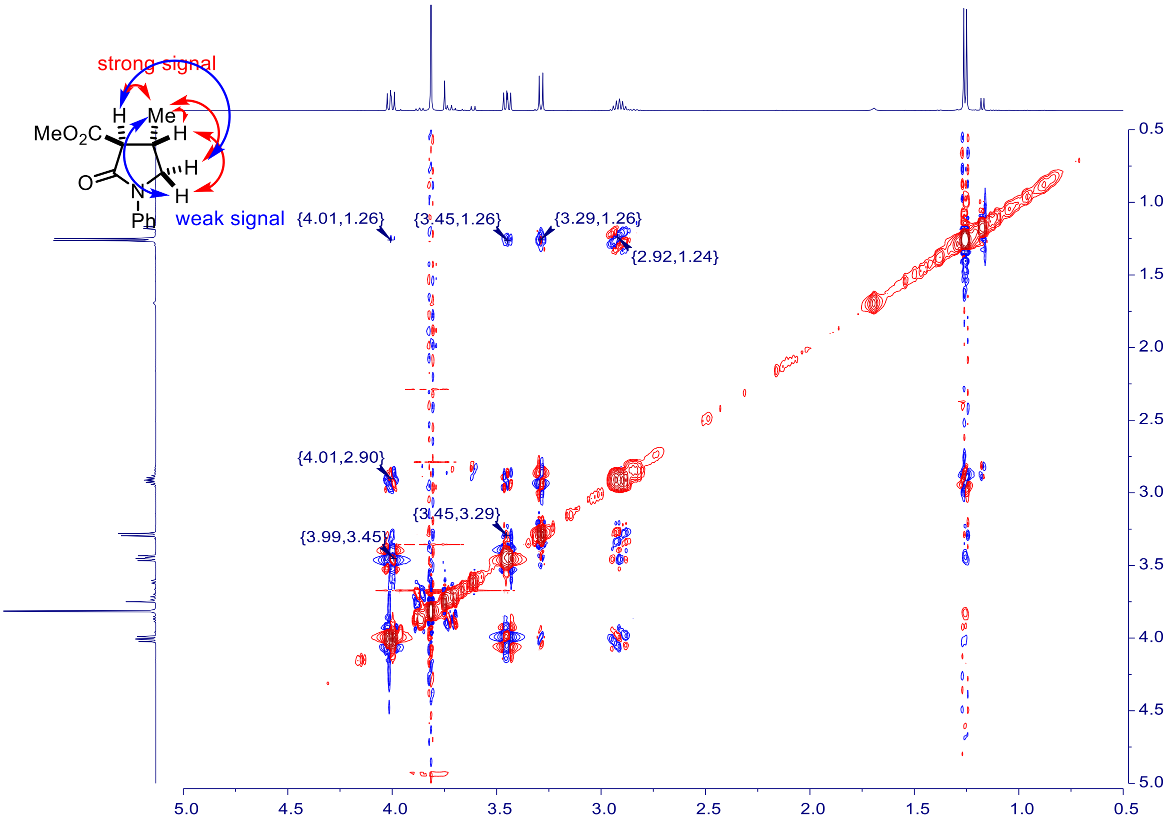
strong signal
weak signal
MeO2C
Me
H
O
N
Ph
{4.01,1.26}
{3.45,1.26}
{3.29,1.26}
{2.92,1.24}
{4.01,2.90}
{3.45,3.29}
{3.99,3.45}

56 – ^{1}H NMR (400 MHz, $CDCl_3$)

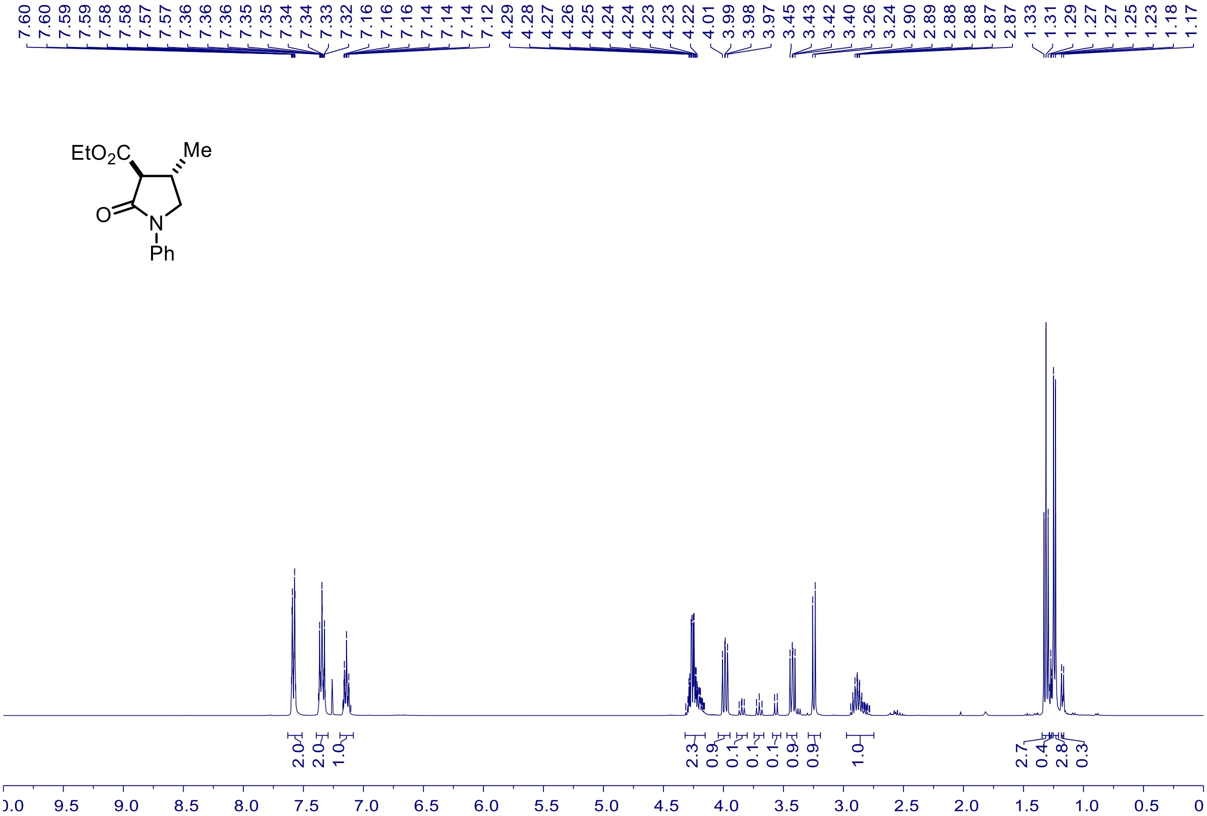

56 – ^{13}C NMR (101 MHz, $CDCl_3$)

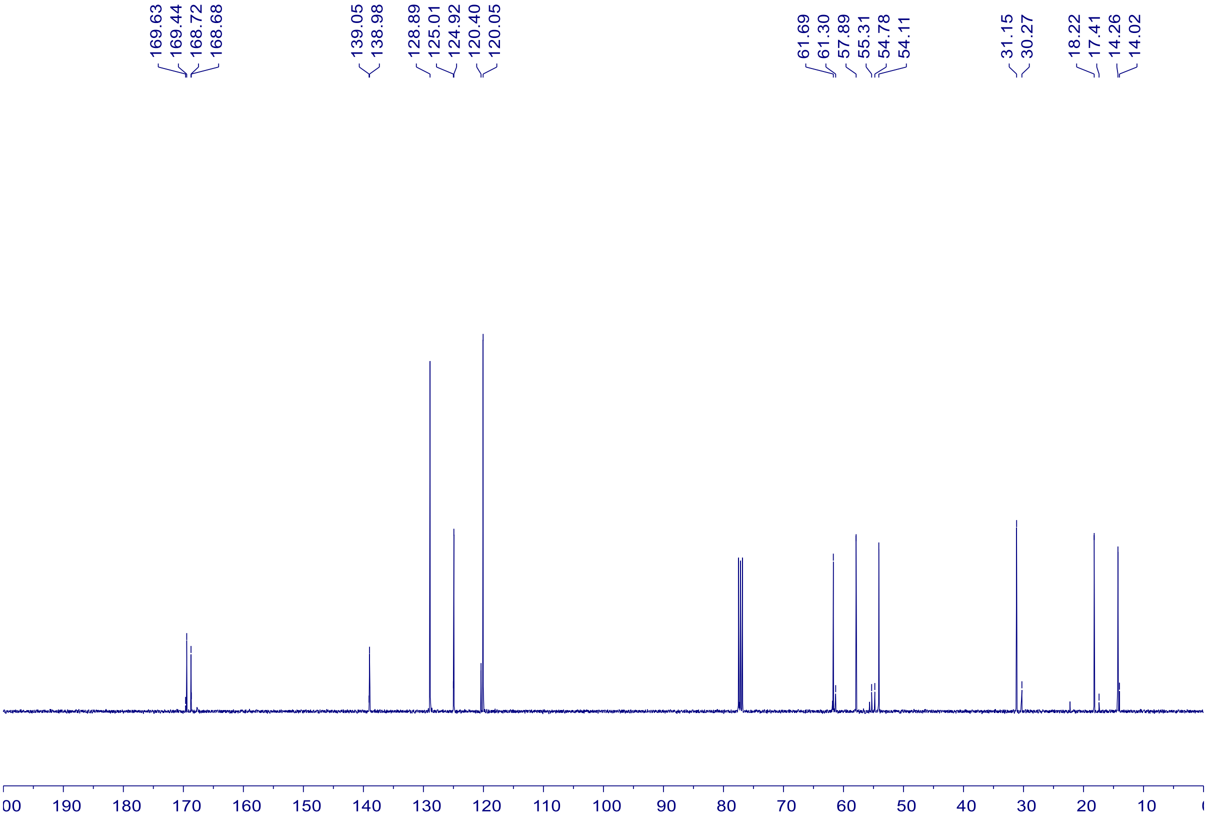

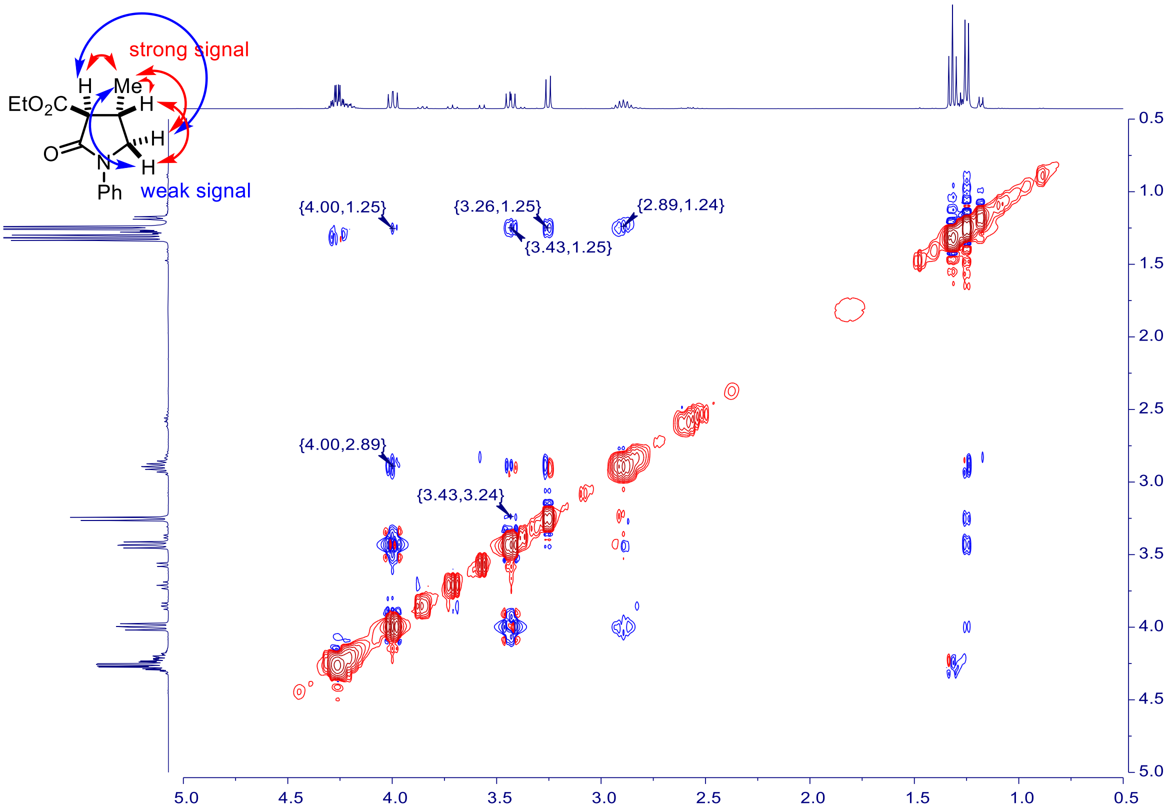
strong signal
weak signal
EtO2C
Me
H
O
N
Ph
{4.00,1.25}
{3.26,1.25}
{3.43,1.25}
{2.89,1.24}
{4.00,2.89}
{3.43,3.24}
0.5
1.0
1.5
2.0
2.5
3.0
3.5
4.0
4.5
5.0

57 – ^{1}H NMR (500 MHz, $CDCl_3$)

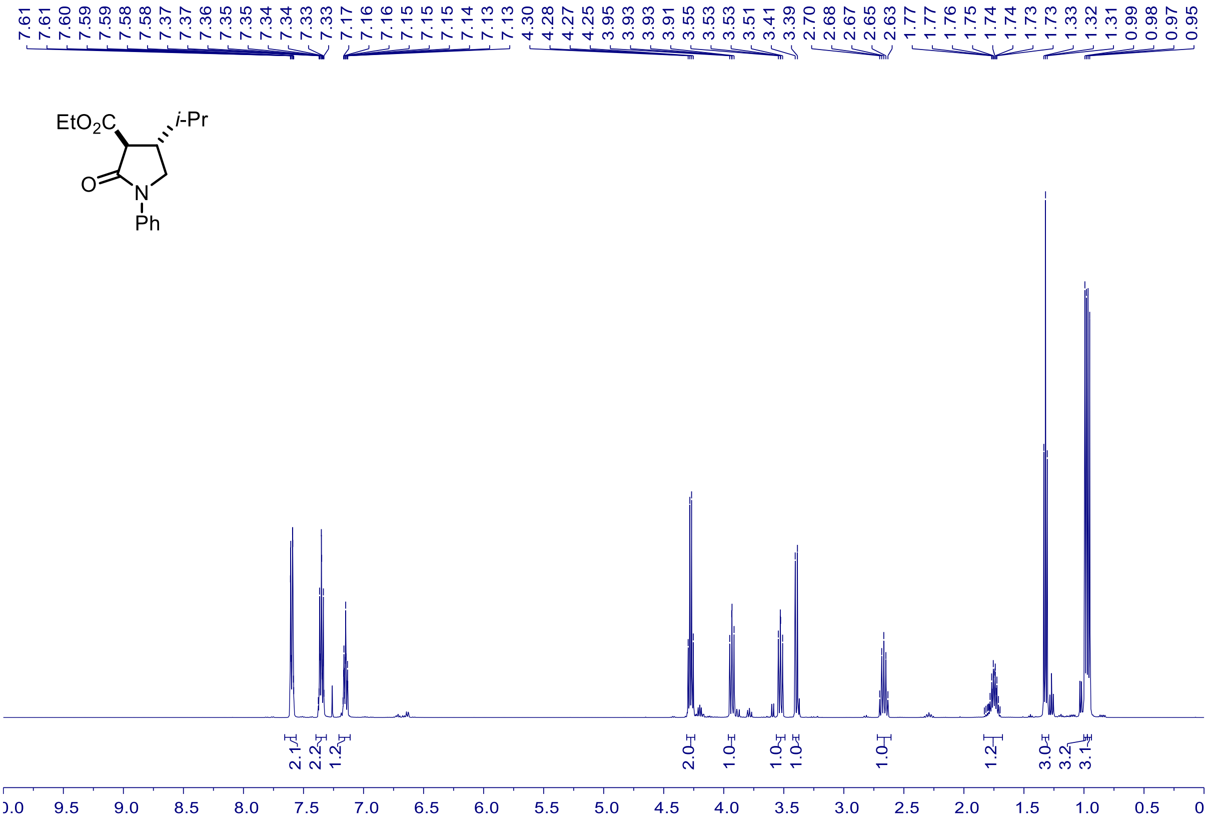

57 – ^{13}C NMR (126 MHz, $CDCl_3$)

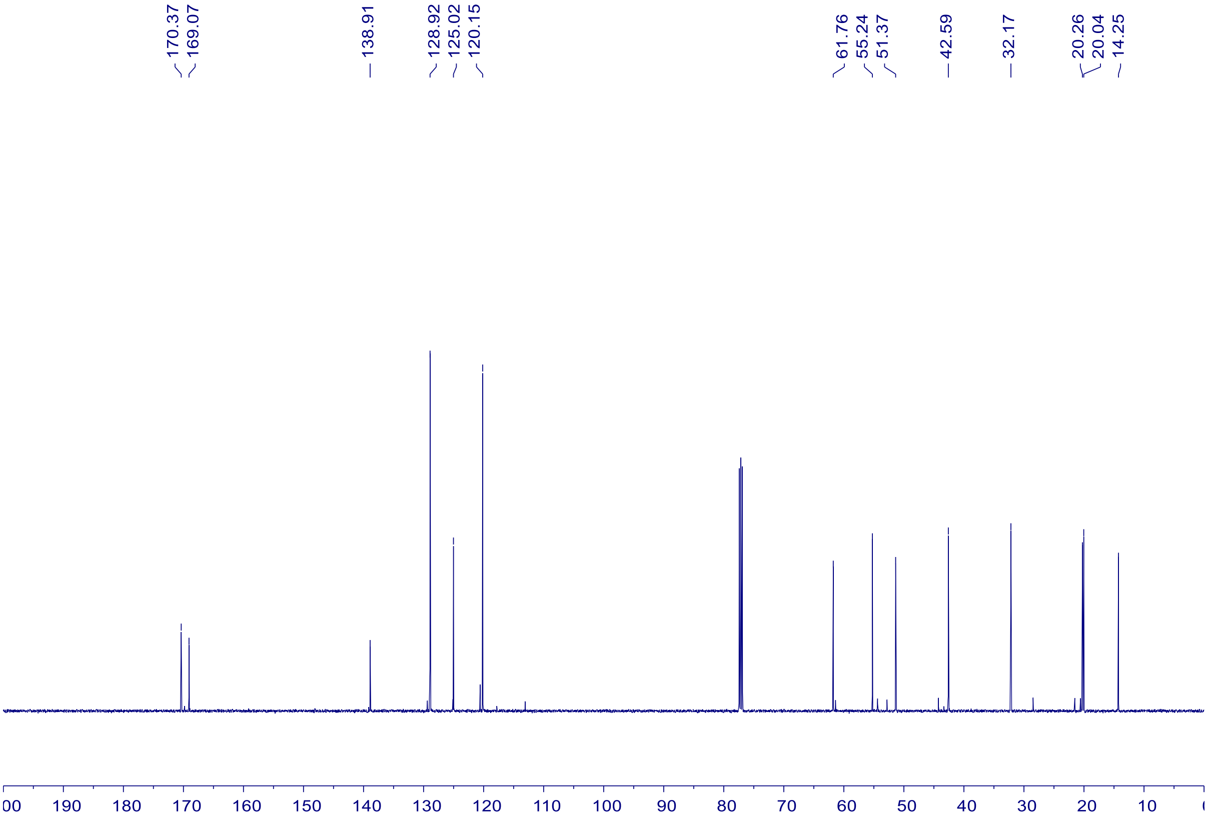

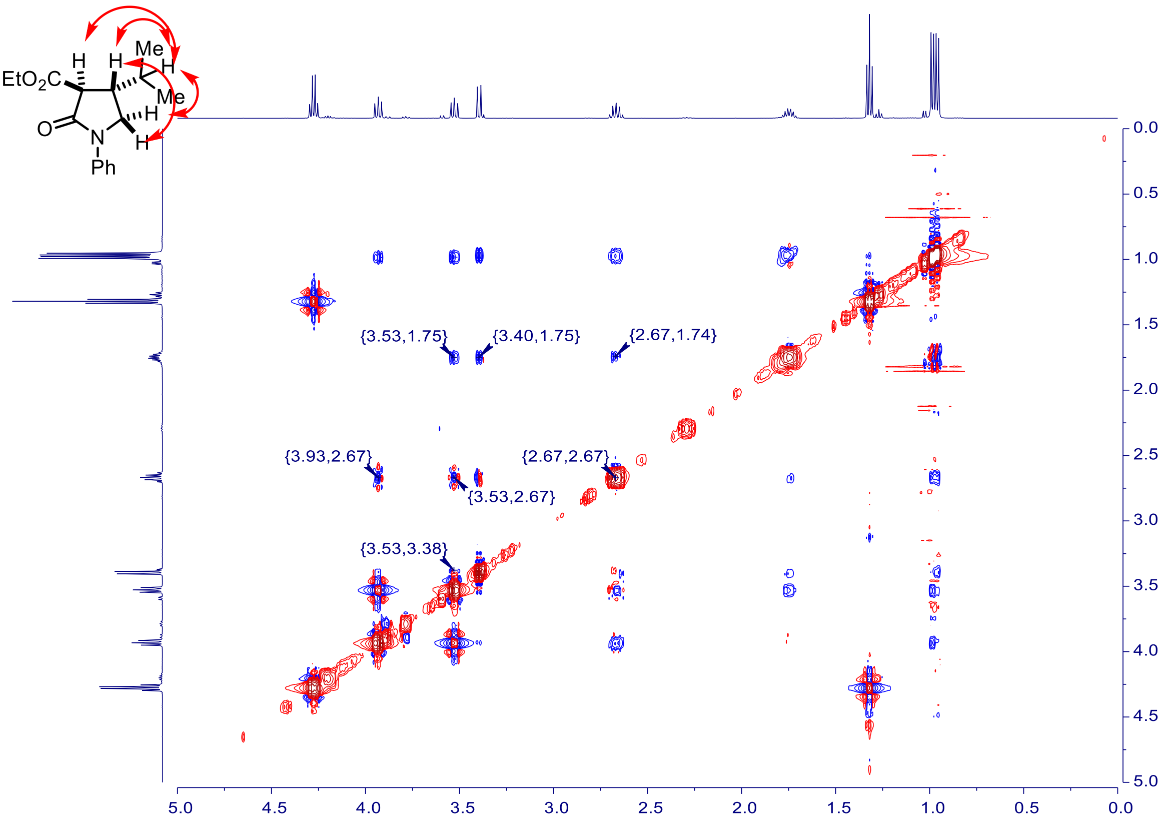
EtO2C
O
N
Ph
Me
Me
{3.53,1.75}
{3.40,1.75}
{2.67,1.74}
{3.93,2.67}
{2.67,2.67}
{3.53,2.67}
{3.53,3.38}

58 – ^{1}H NMR (400 MHz, $CDCl_3$)

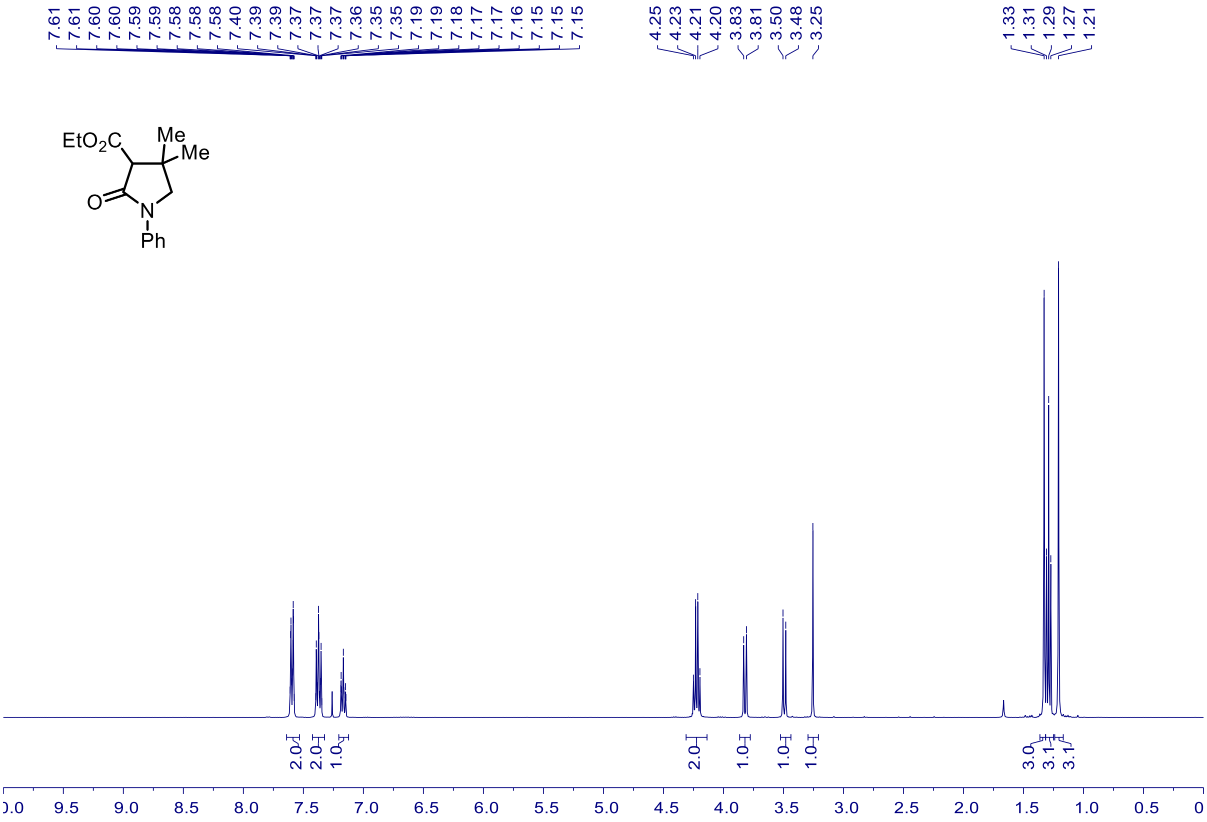

58 – ^{13}C NMR (101 MHz, $CDCl_3$)

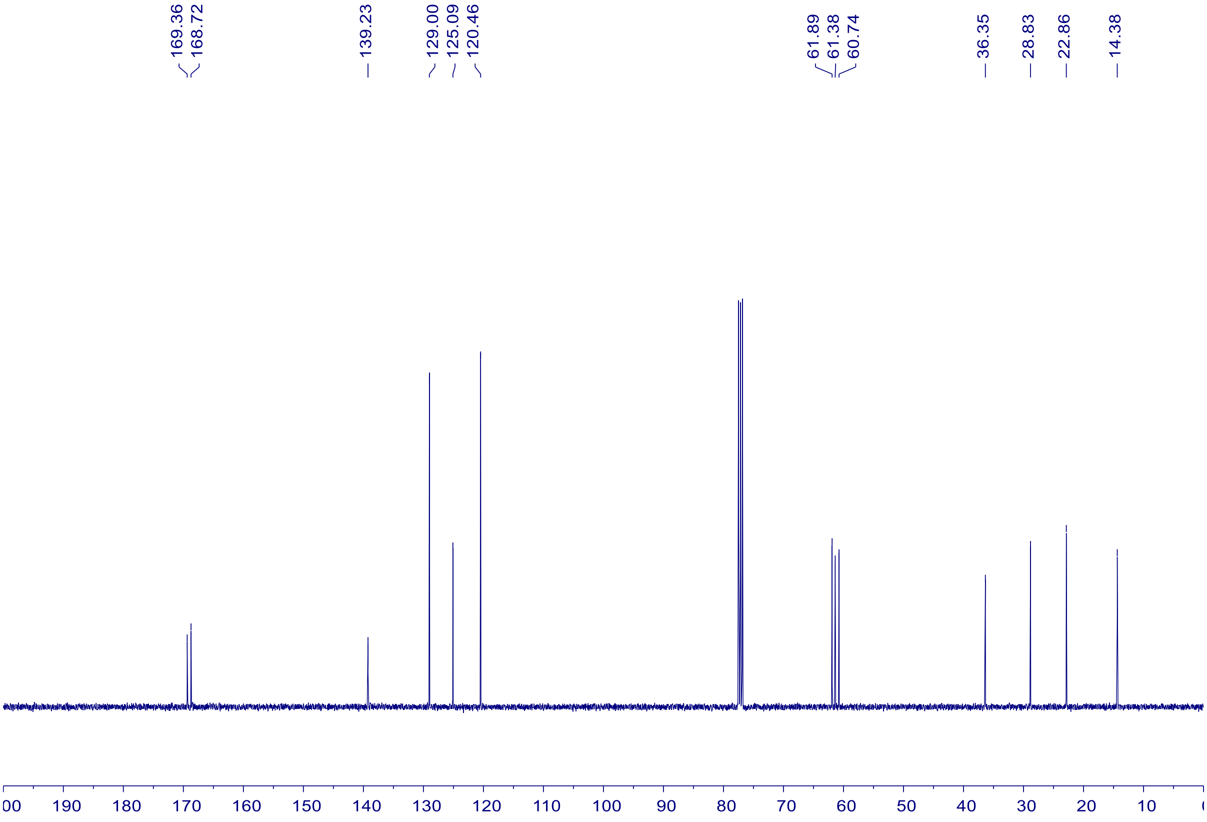

59 – ^{1}H NMR (400 MHz, $CDCl_3$)

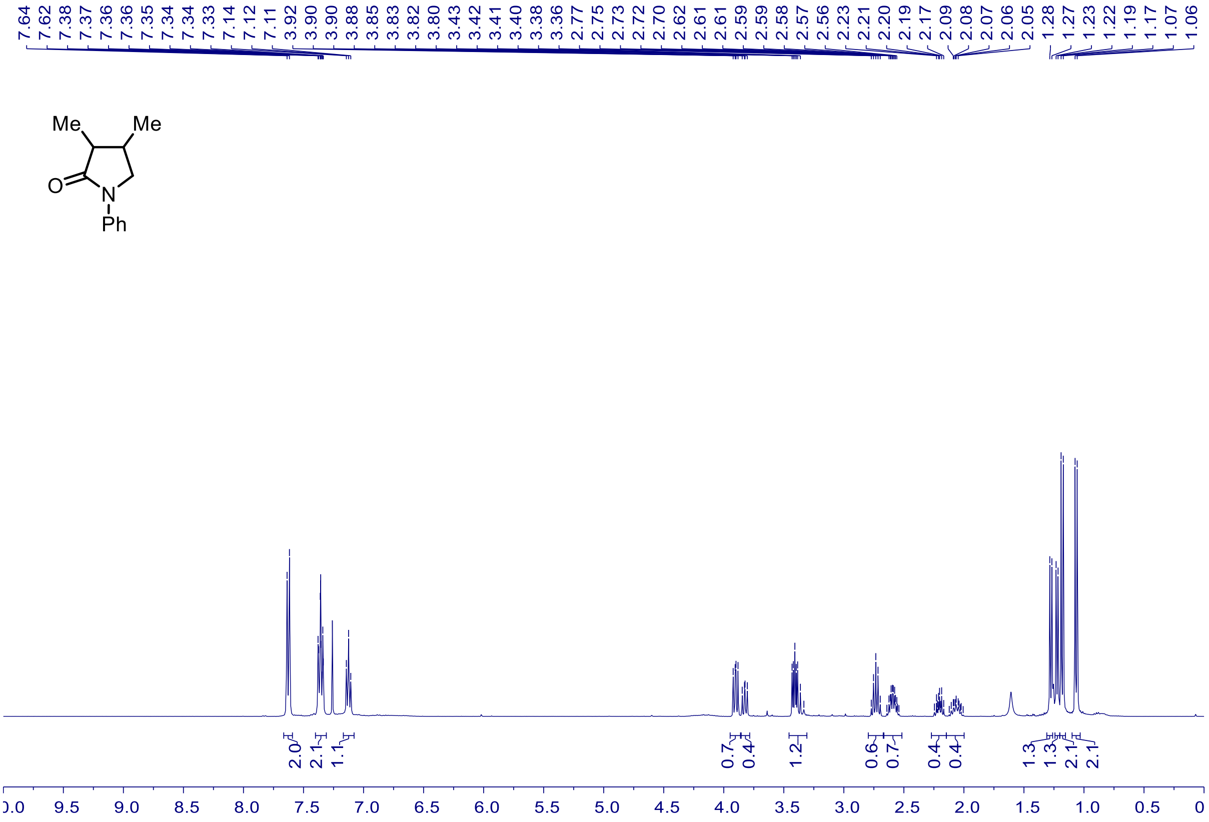

59 – ^{13}C NMR (101 MHz, $CDCl_3$)

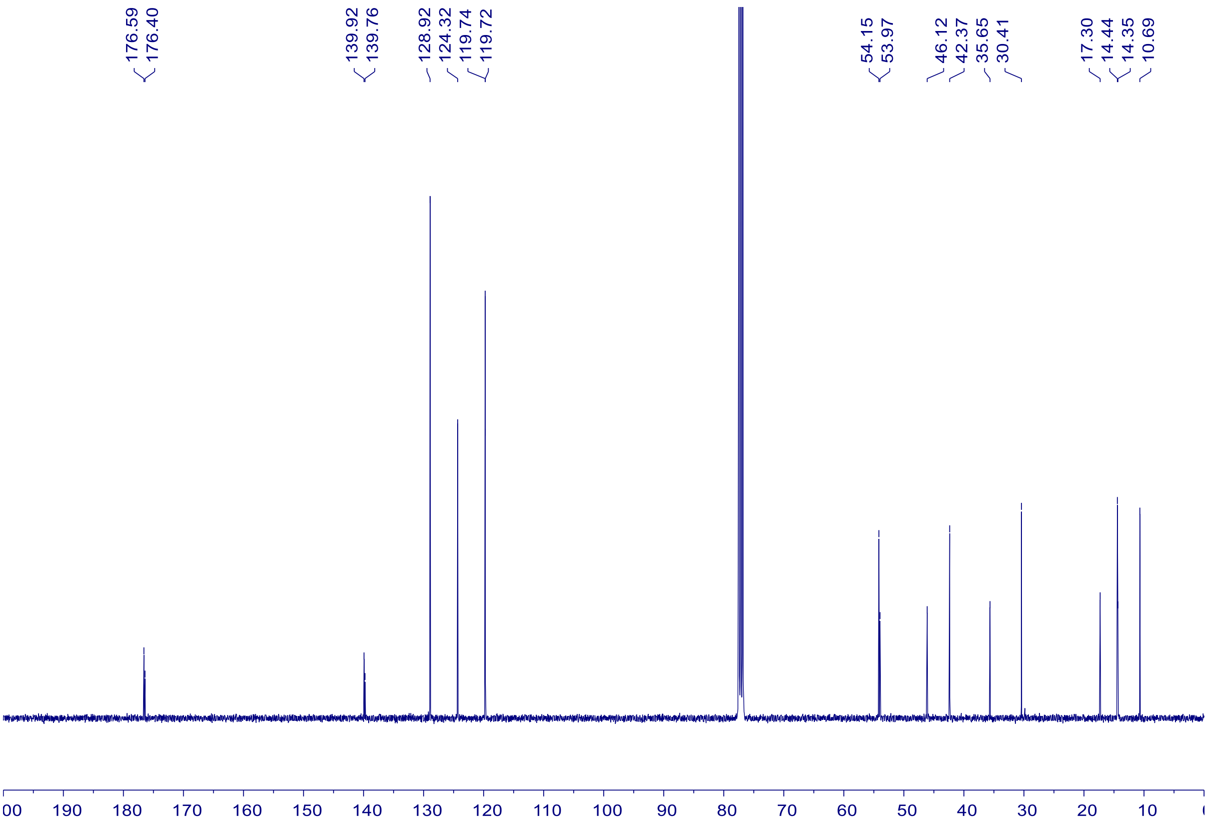

60 – ^{1}H NMR (400 MHz, $CDCl_3$)

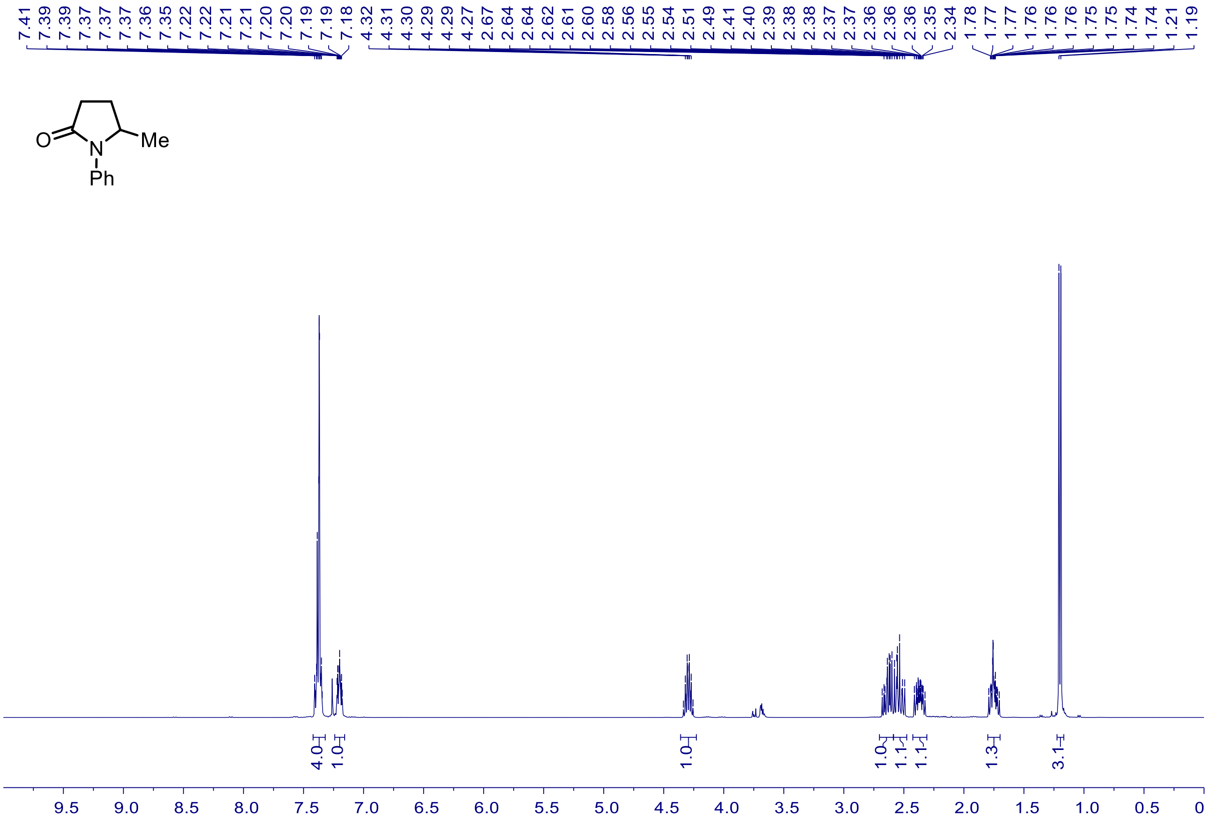

60 – ^{13}C NMR (101 MHz, $CDCl_3$)

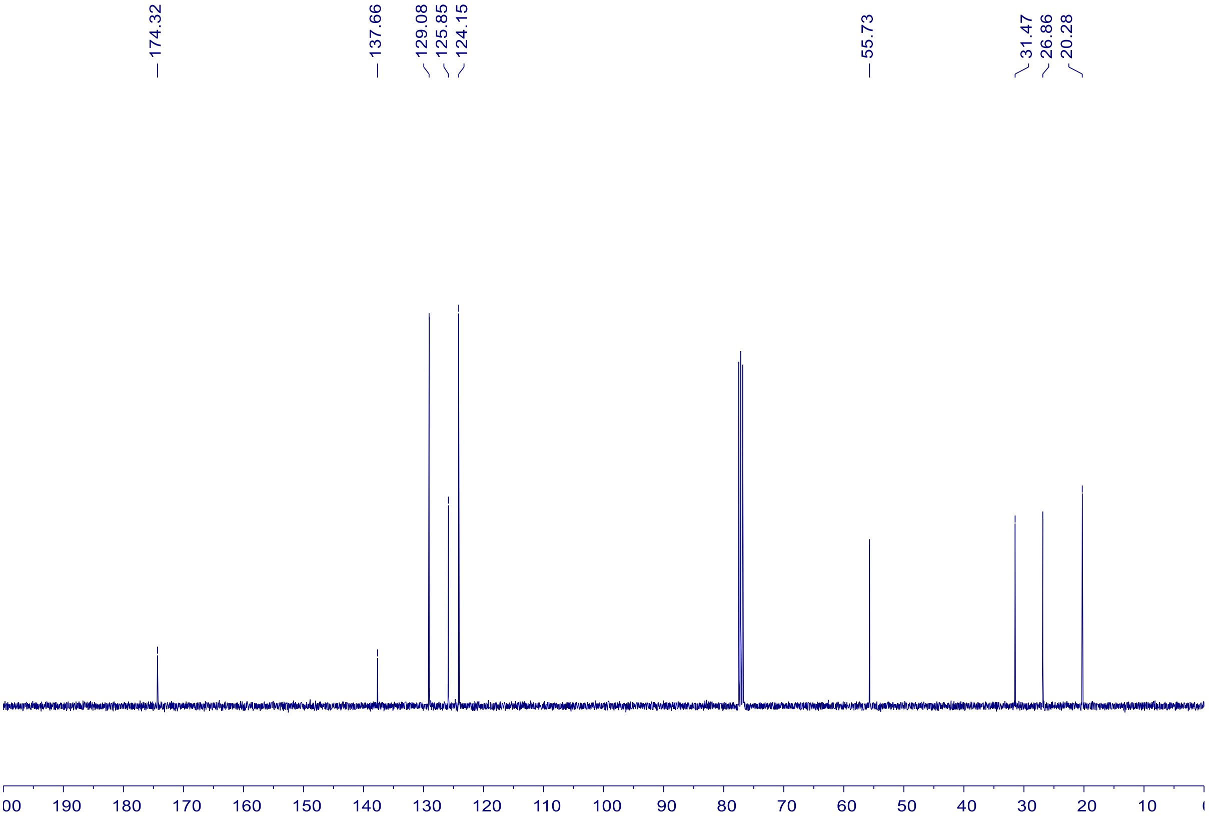

61 *trans* isomer – ^{1}H NMR (500 MHz, $CDCl_3$)

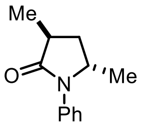

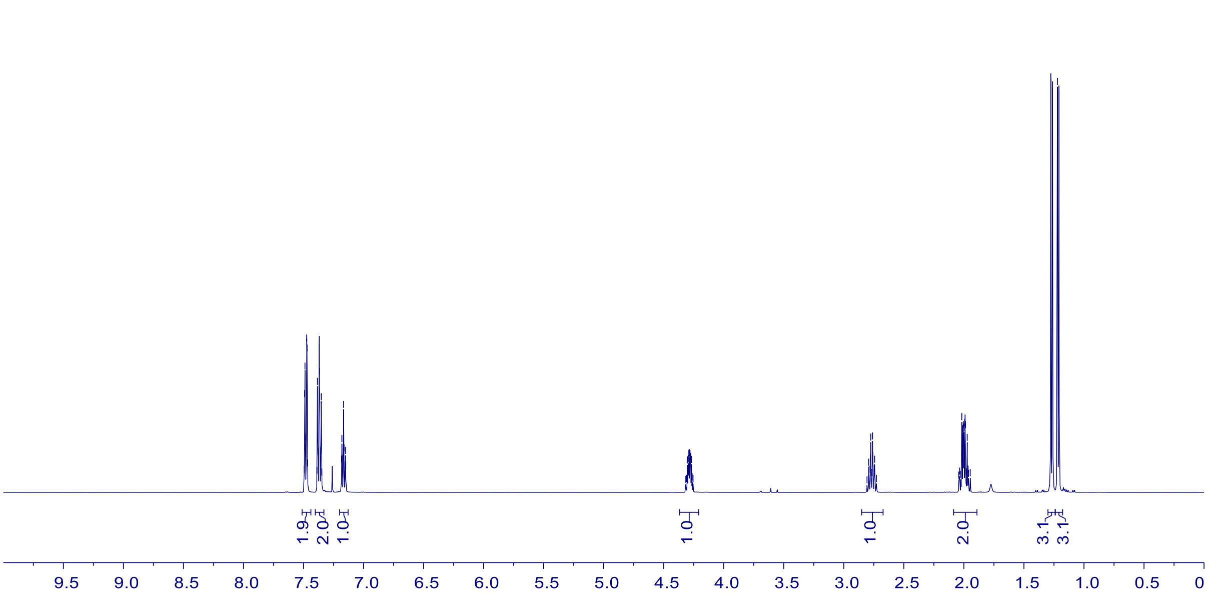

61 *trans* isomer – ^{13}C NMR (126 MHz, $CDCl_3$)

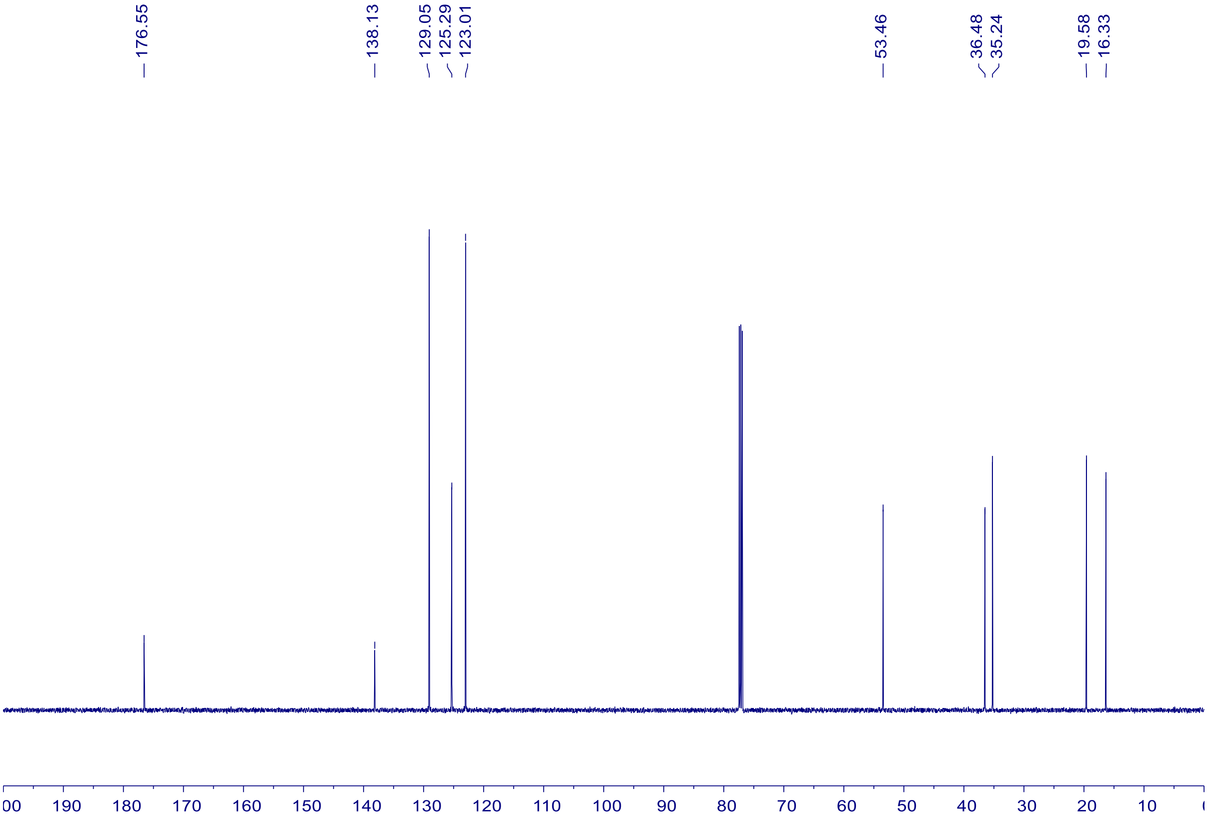

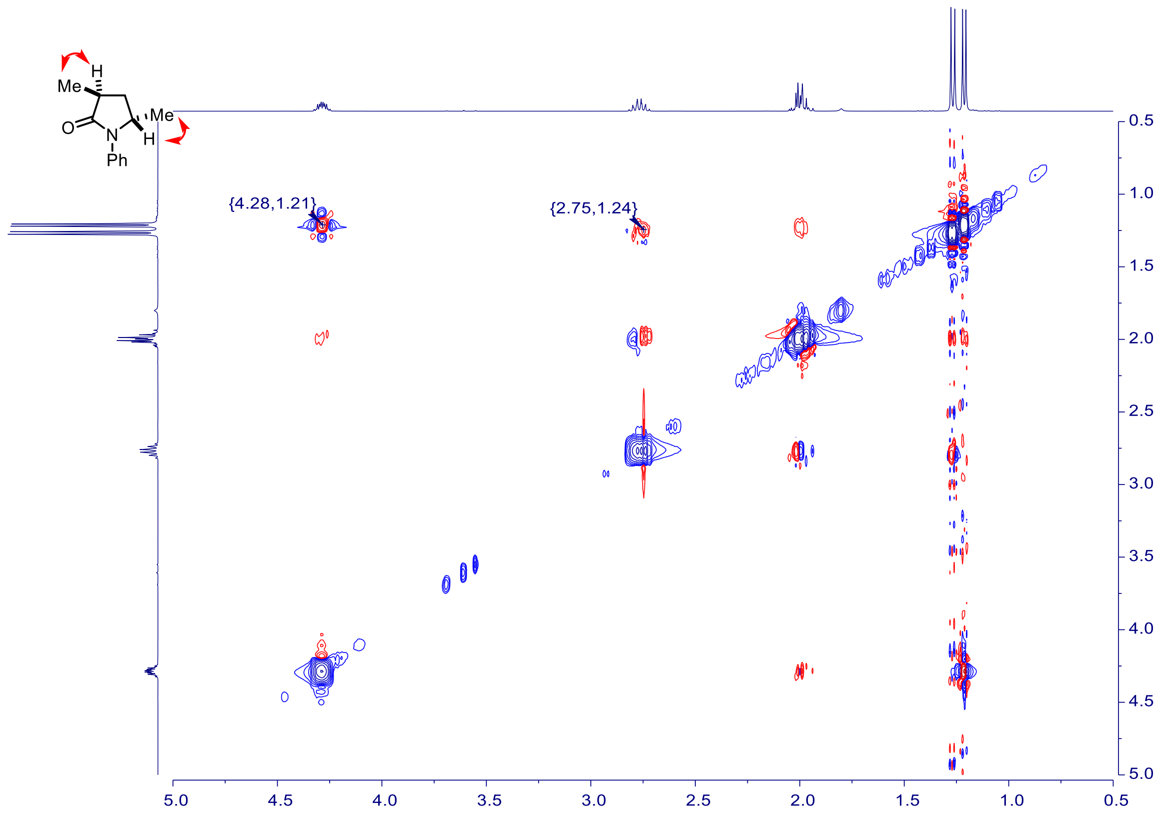

61 *cis* isomer – ^{1}H NMR (500 MHz, $CDCl_3$)

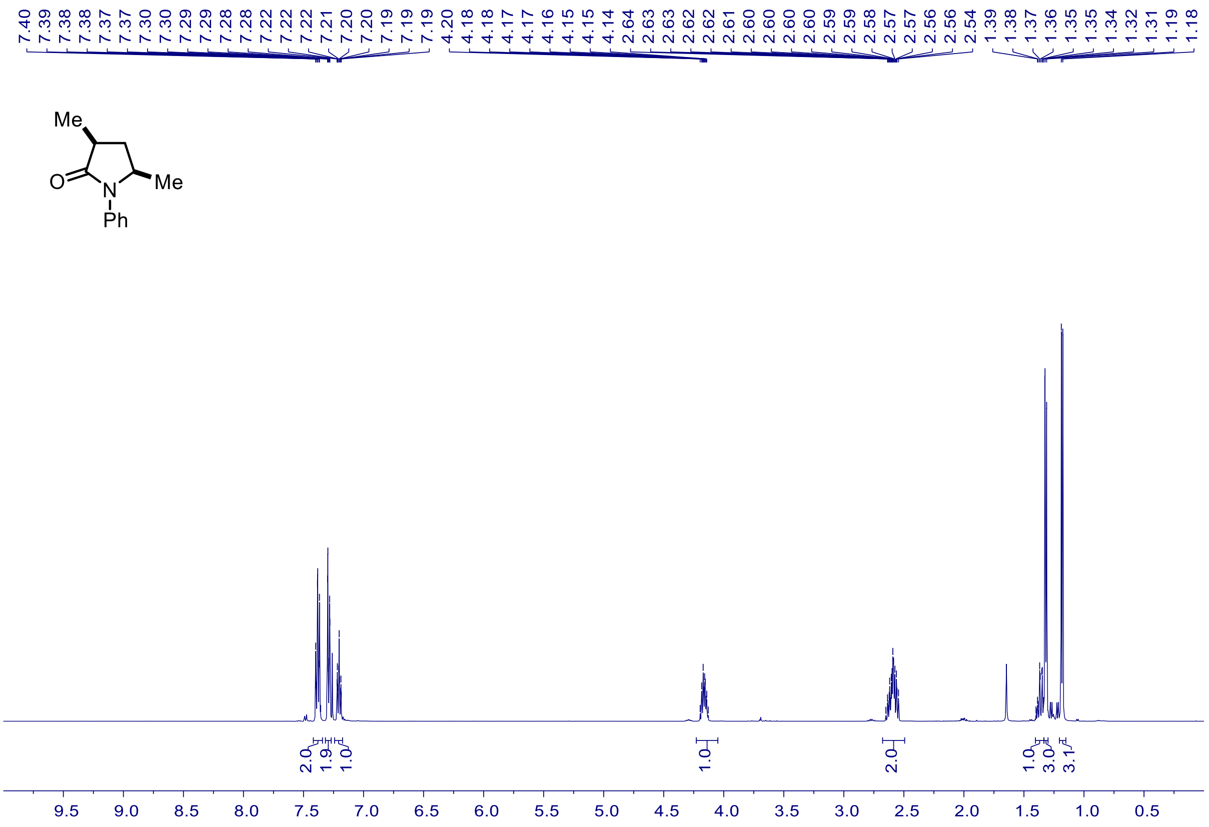

61 *cis* isomer – ^{13}C NMR (126 MHz, $CDCl_3$)

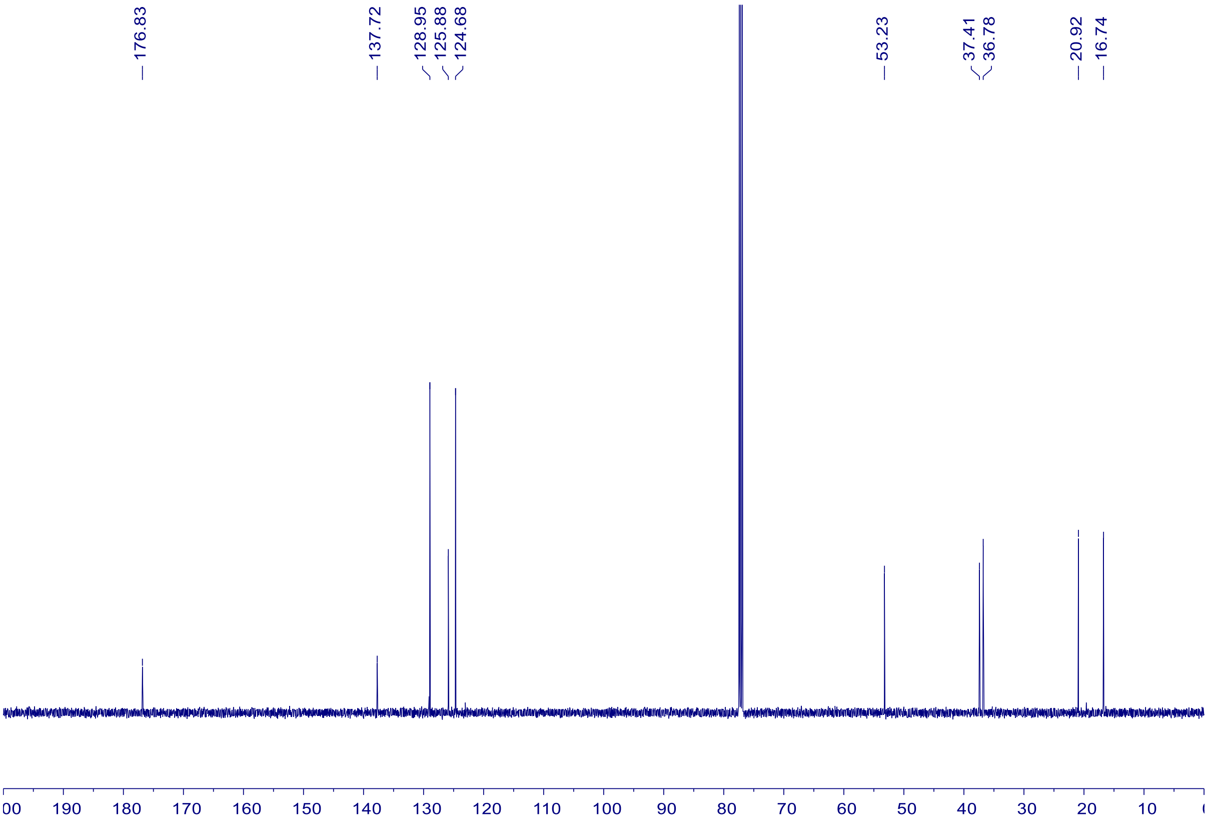

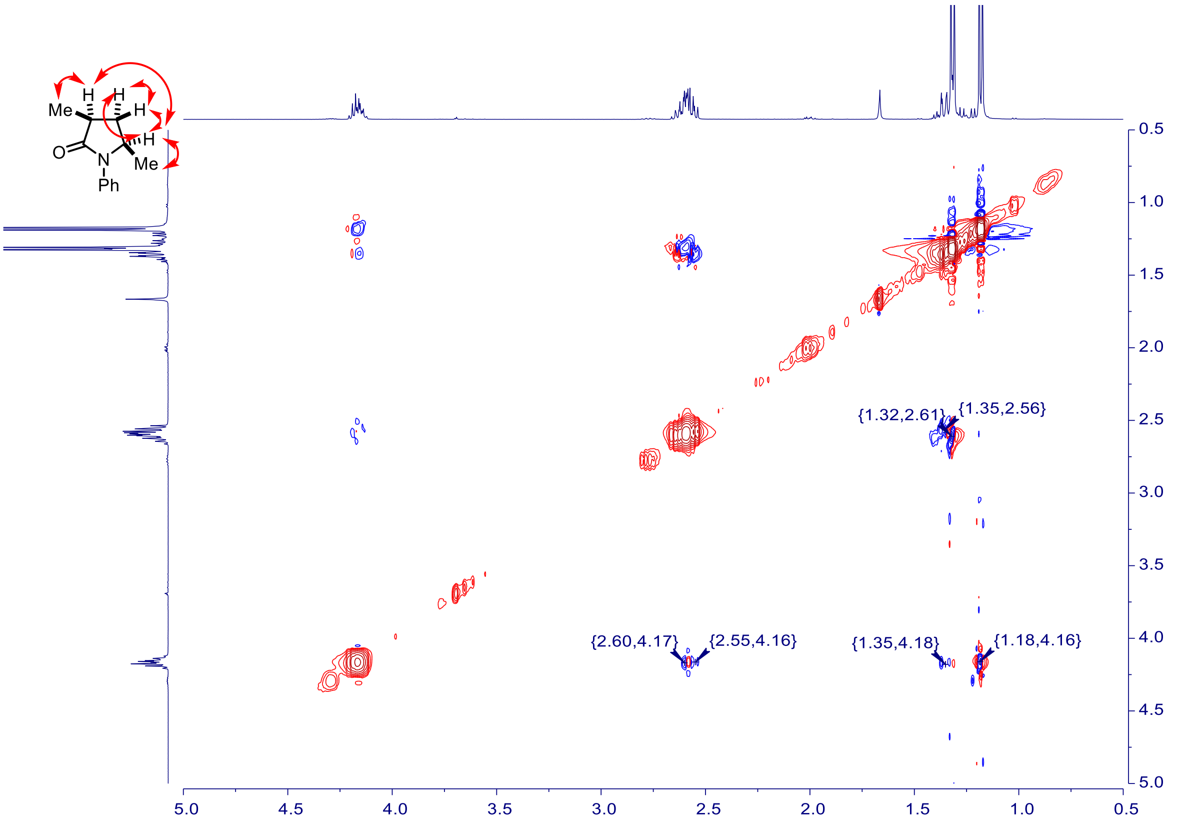

62 *trans-trans* isomer – ^{1}H NMR (500 MHz, $CDCl_3$)

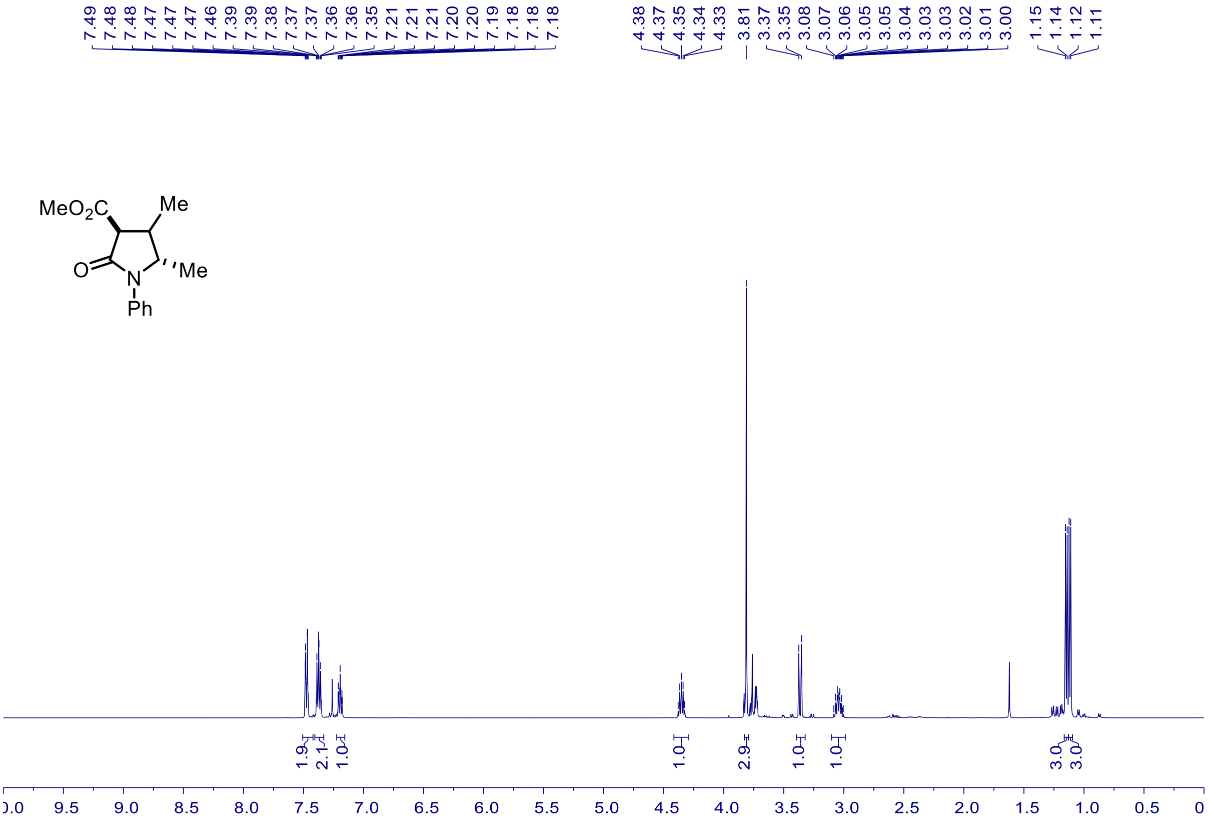

62 *trans-trans* isomer – ^{13}C NMR (126 MHz, $CDCl_3$)

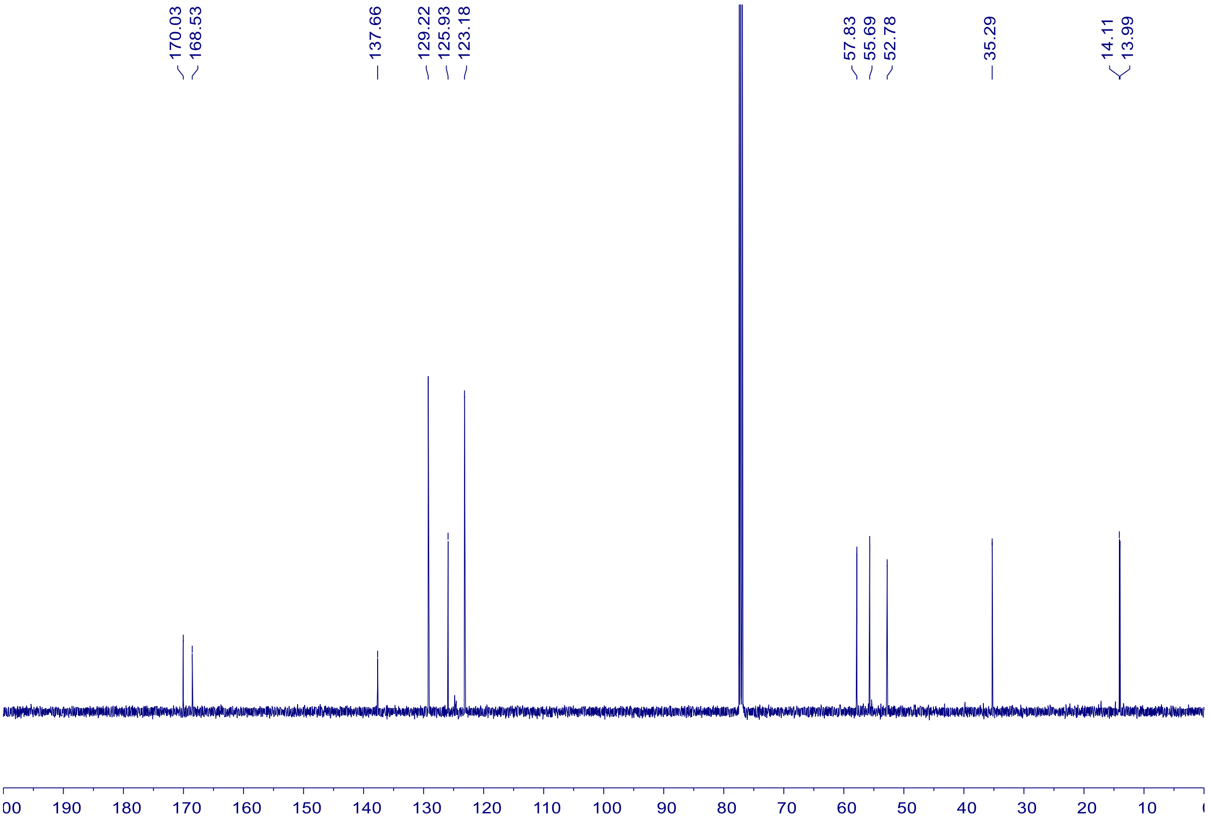

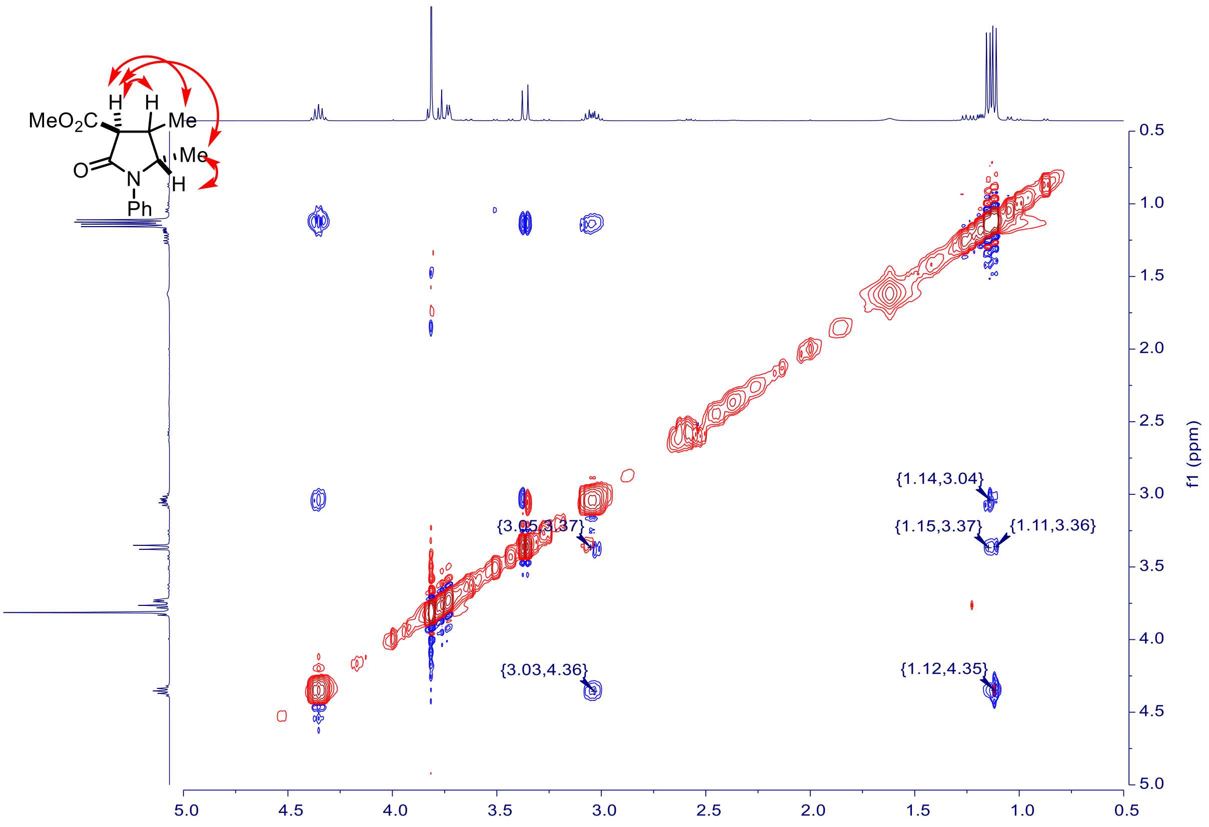

62 *trans-cis* isomer – ^{1}H NMR (500 MHz, $CDCl_3$)

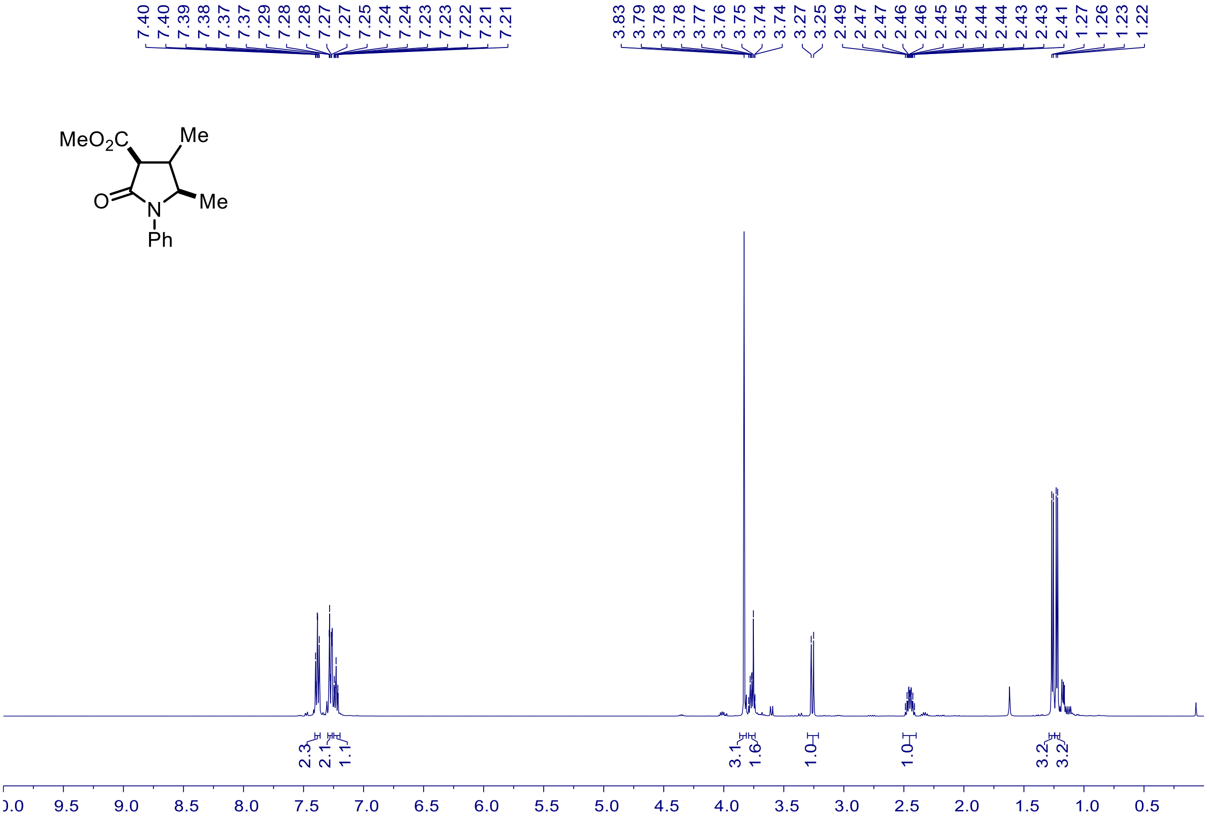

62 *trans-cis* isomer – ^{13}C NMR (126 MHz, $CDCl_3$)

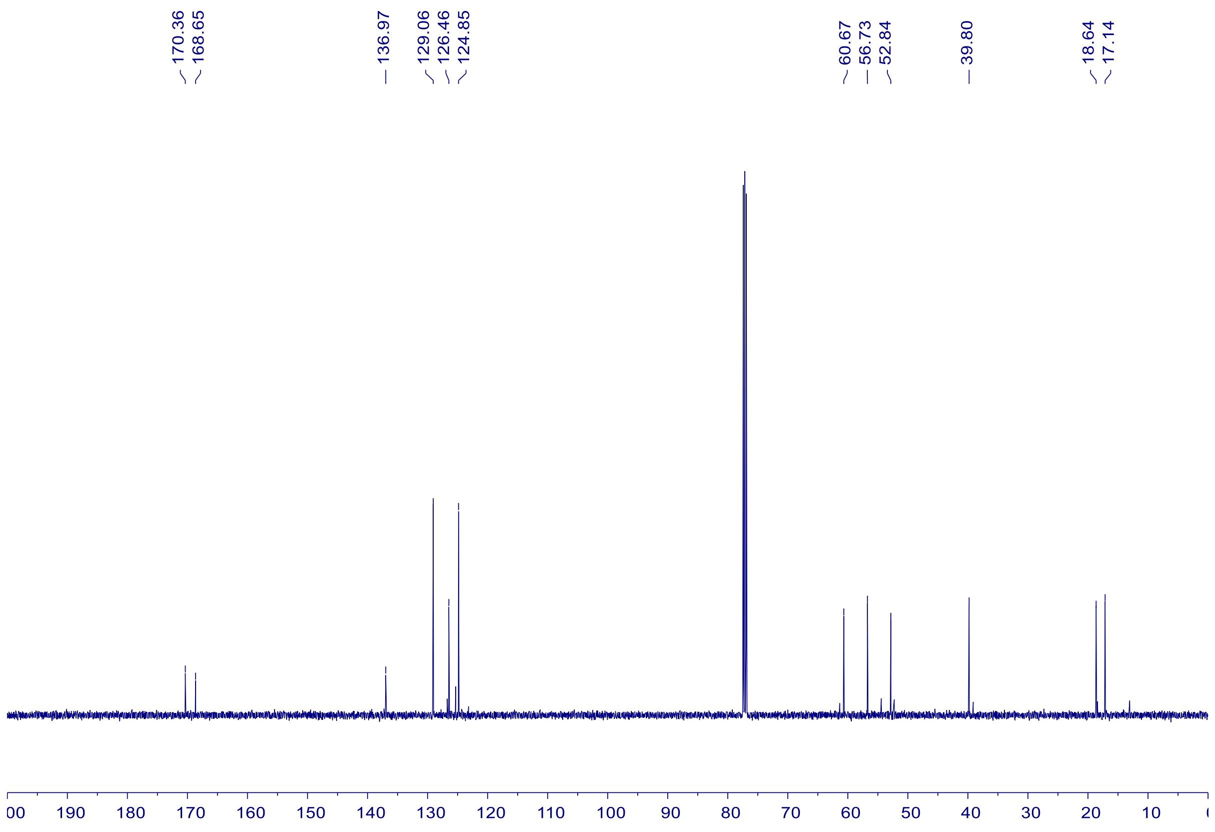

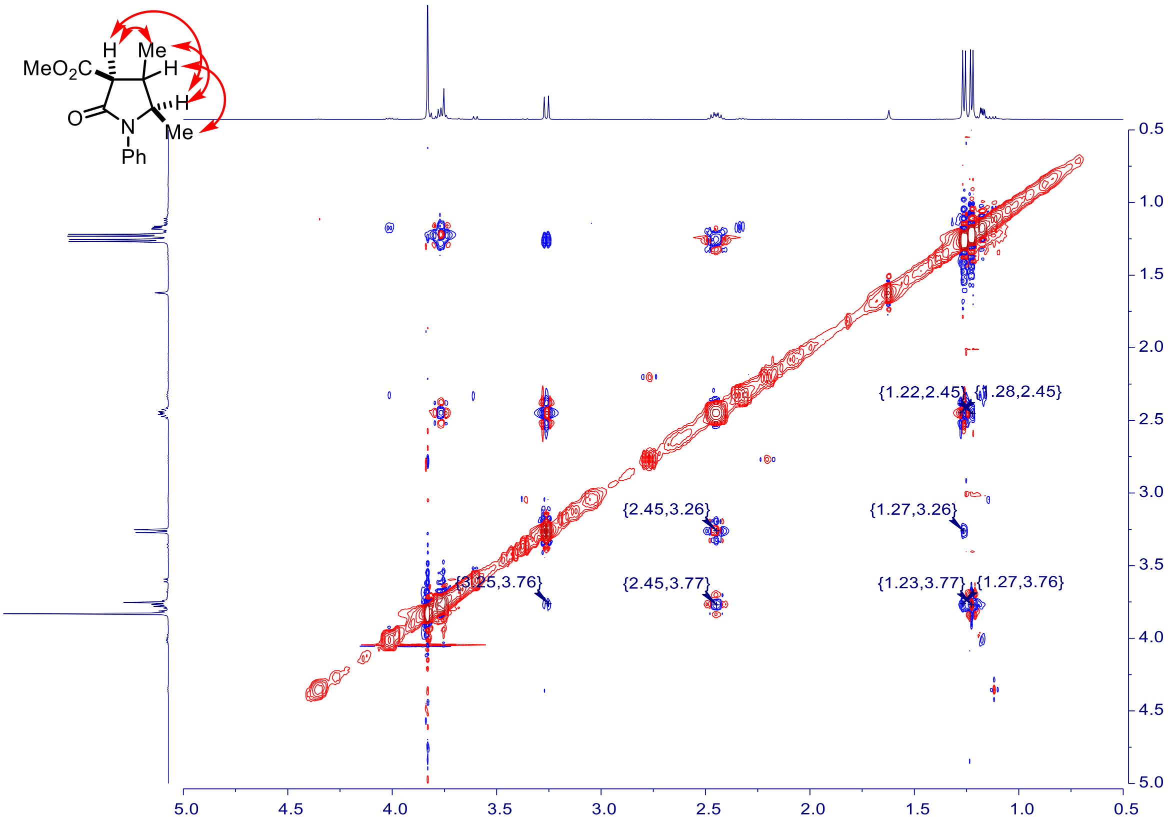

63 *trans* isomer – ^{1}H NMR (500 MHz, $CDCl_3$)

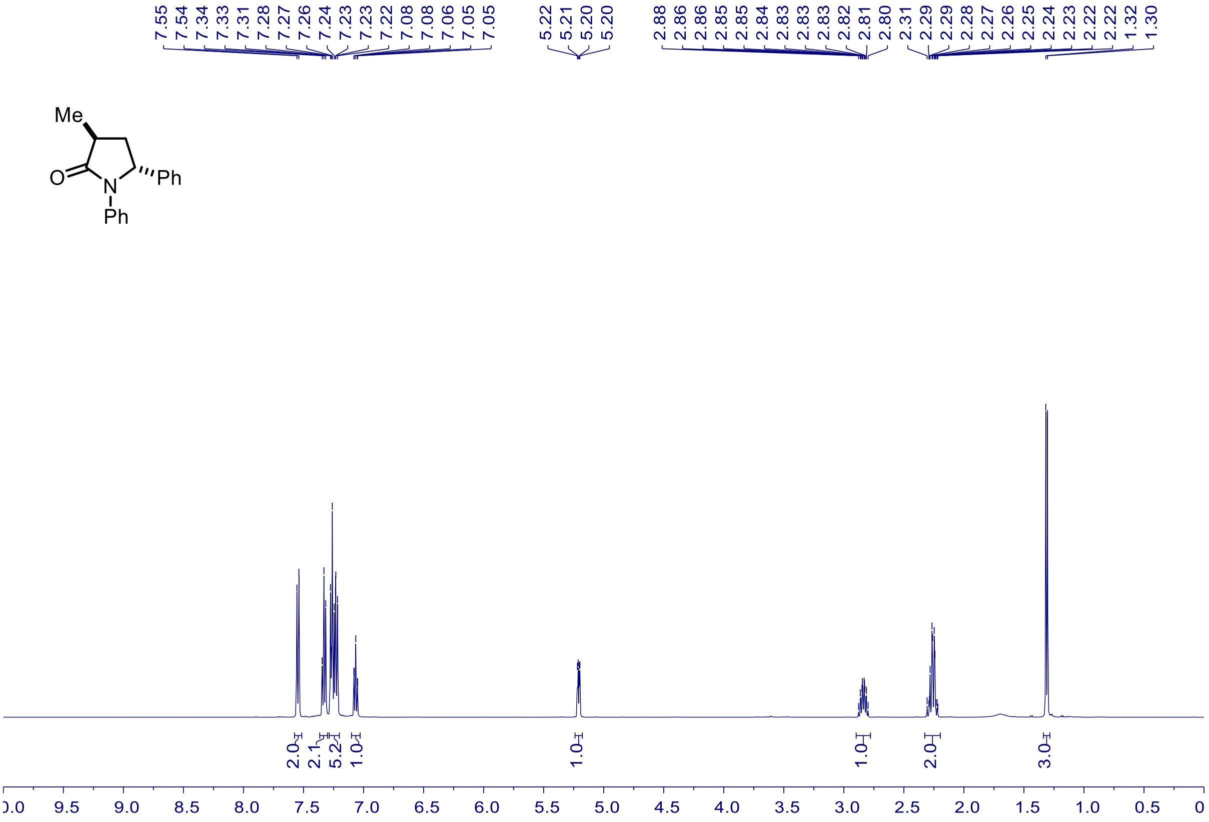

63 *trans* isomer – ^{13}C NMR (126 MHz, $CDCl_3$)

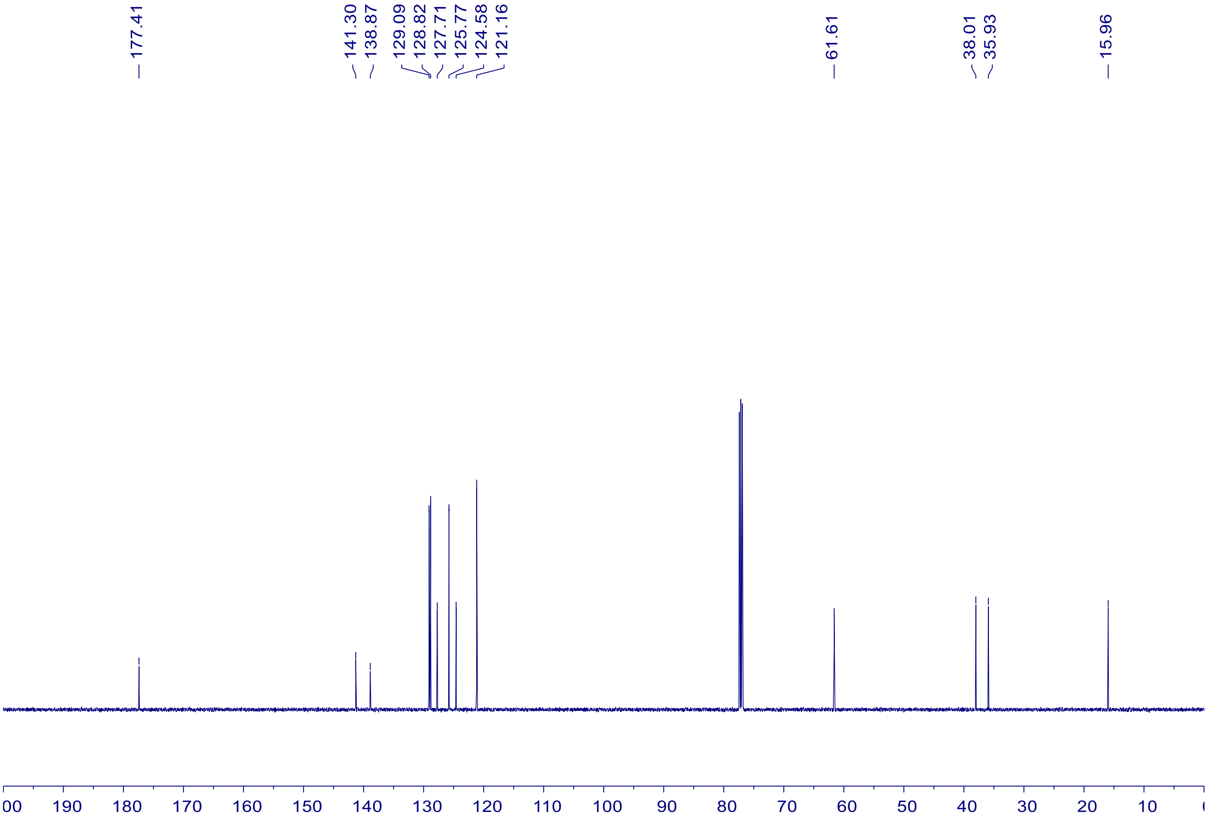

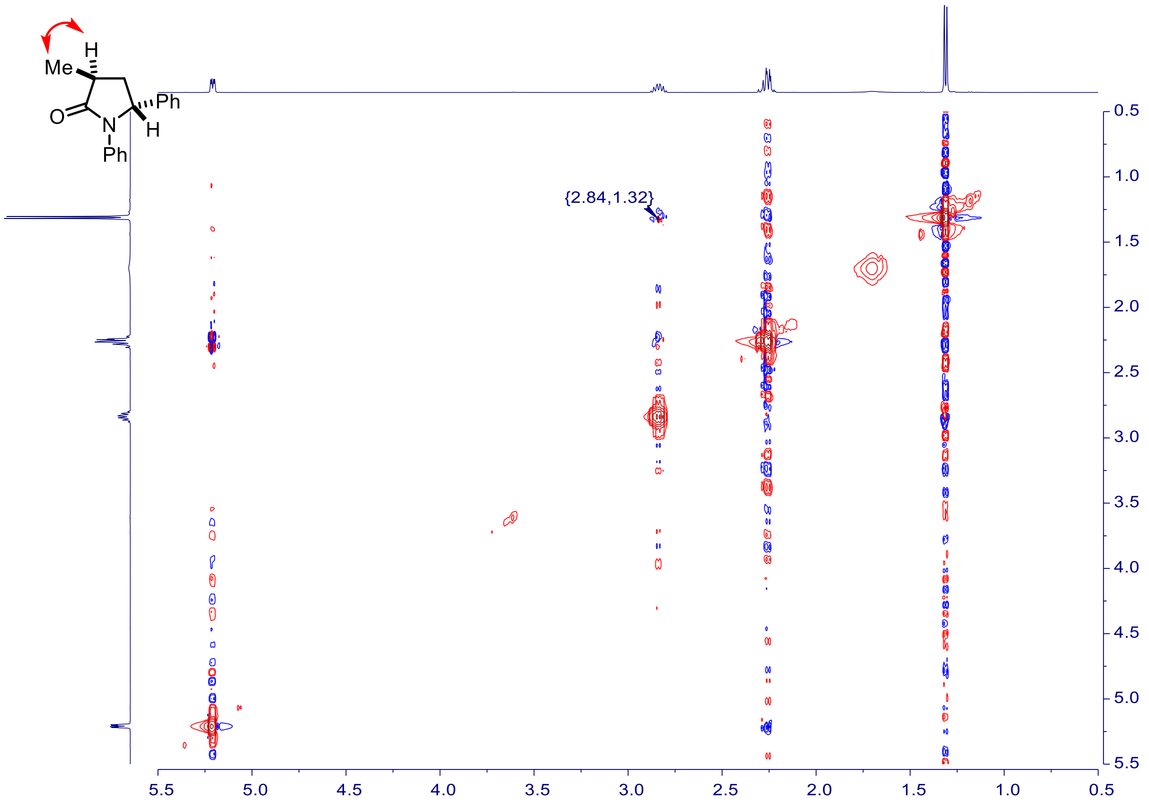

63 *cis* isomer – ^{1}H NMR (500 MHz, $CDCl_3$)

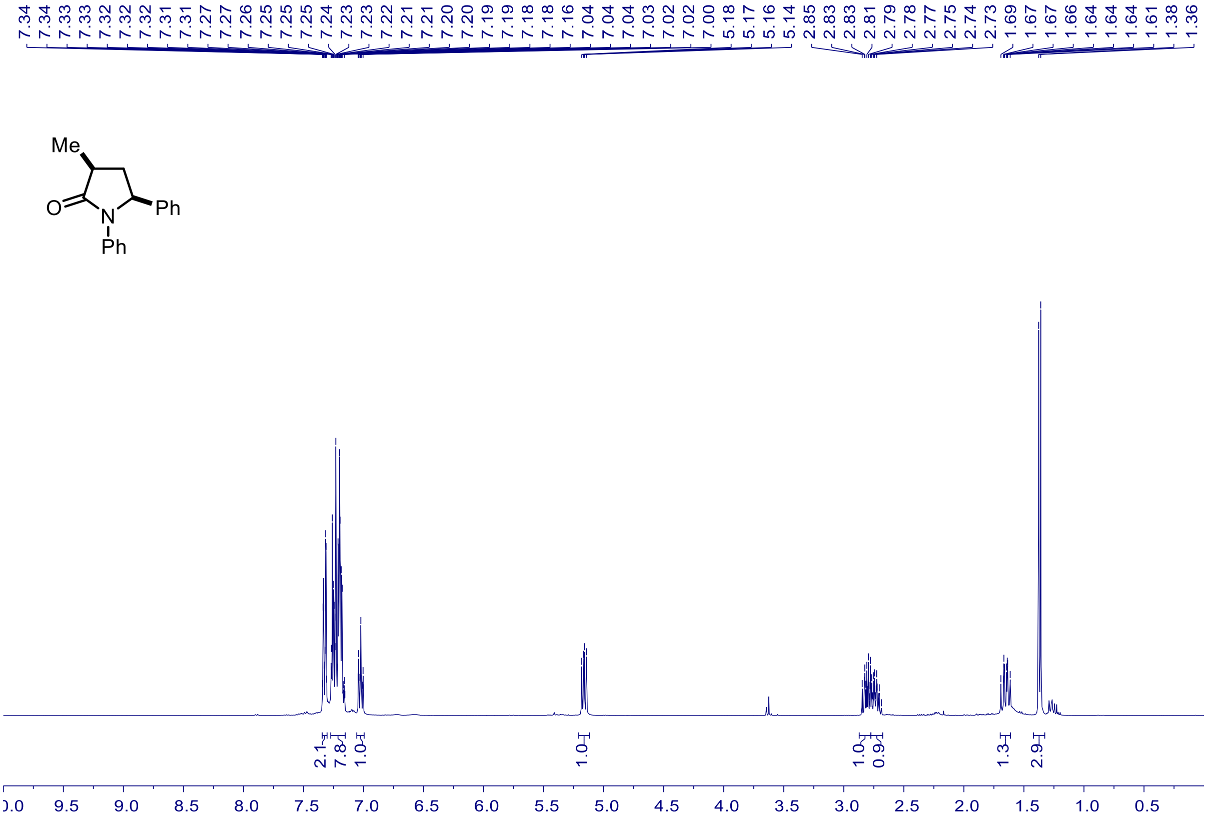

63 *cis* isomer – ^{13}C NMR (101 MHz, $CDCl_3$)

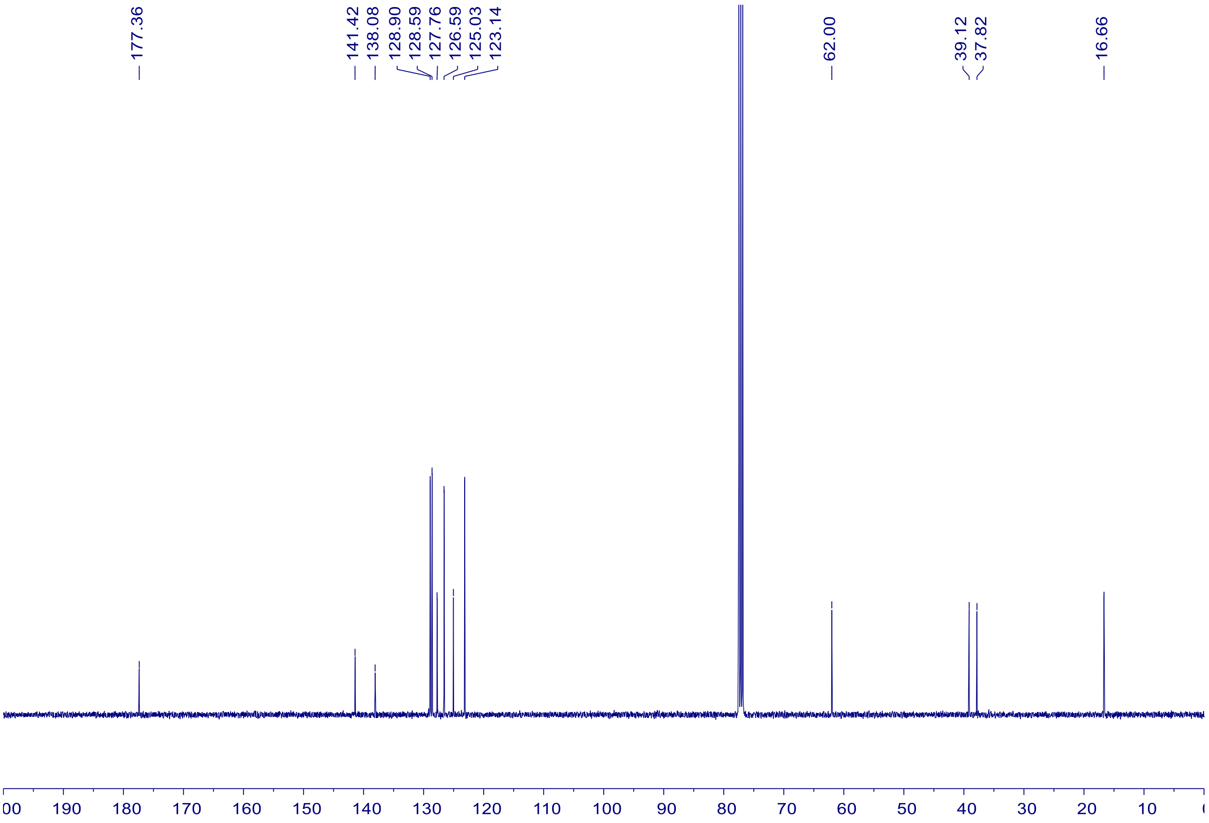

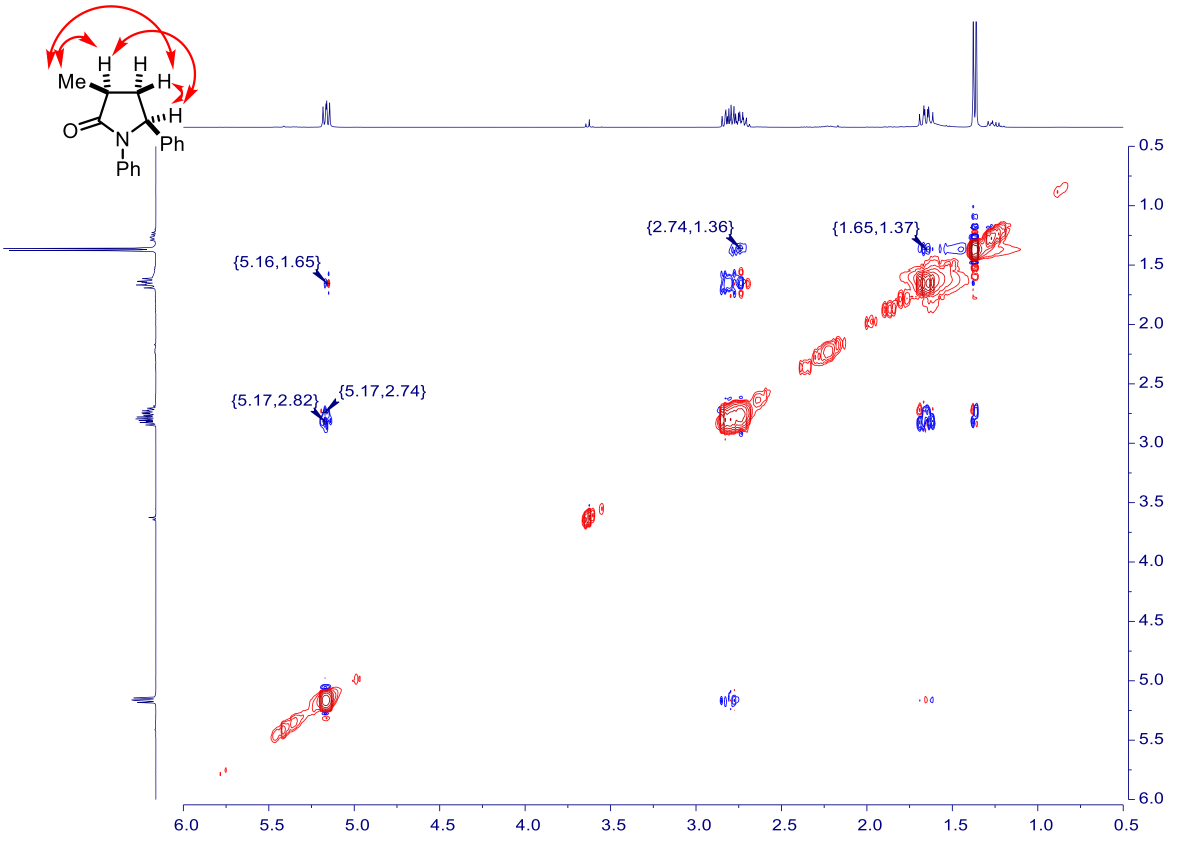

64 *trans* isomer – ^{1}H NMR (400 MHz, $CDCl_3$)

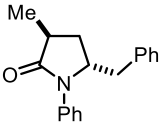

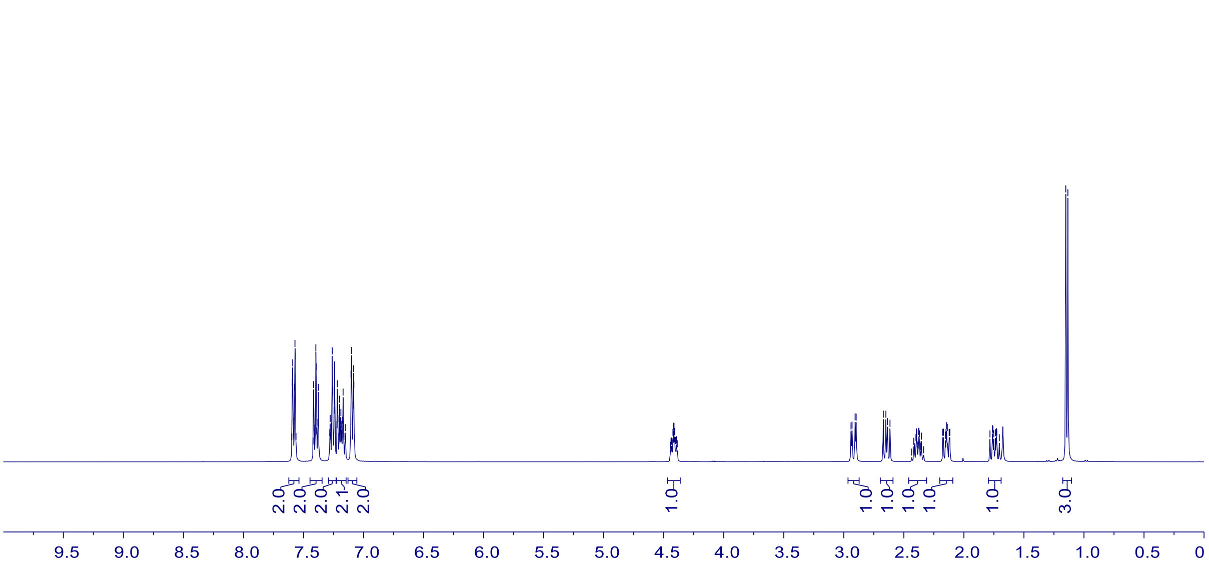

64 *trans* isomer – ^{13}C NMR (101 MHz, $CDCl_3$)

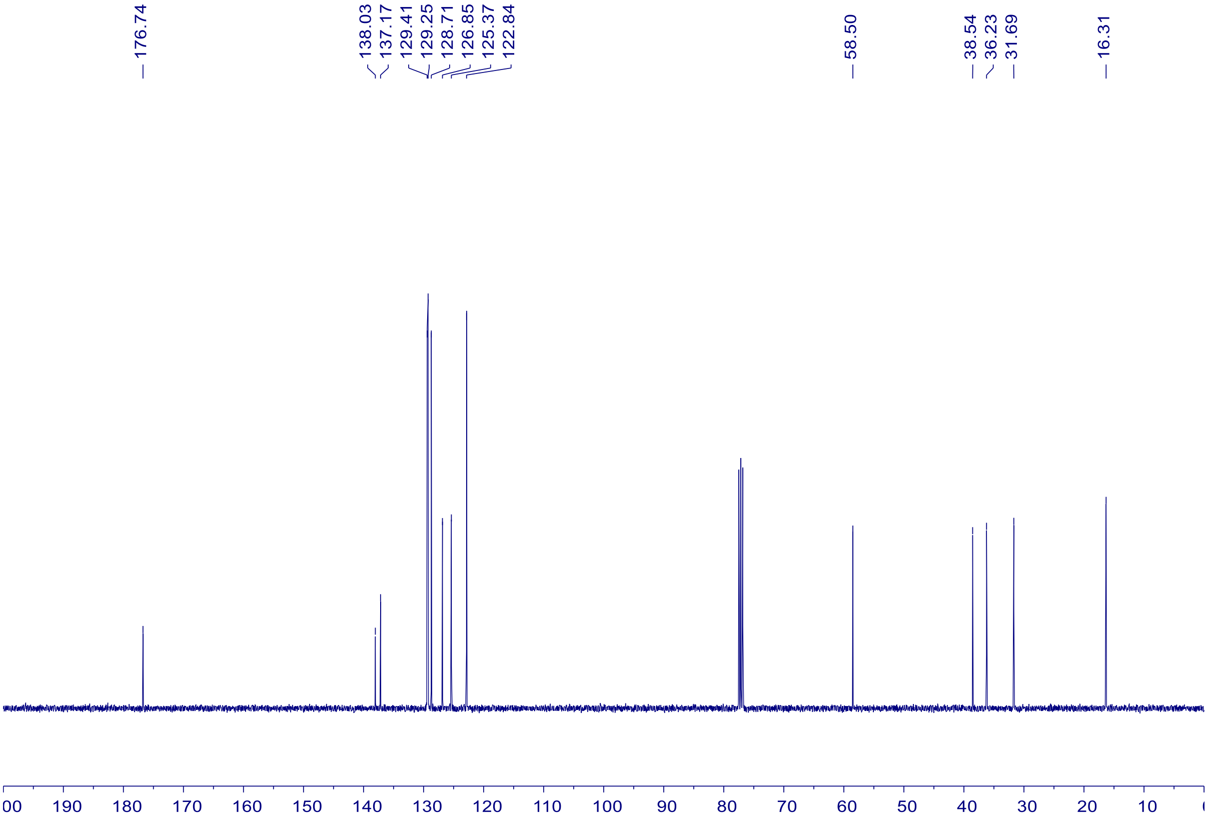

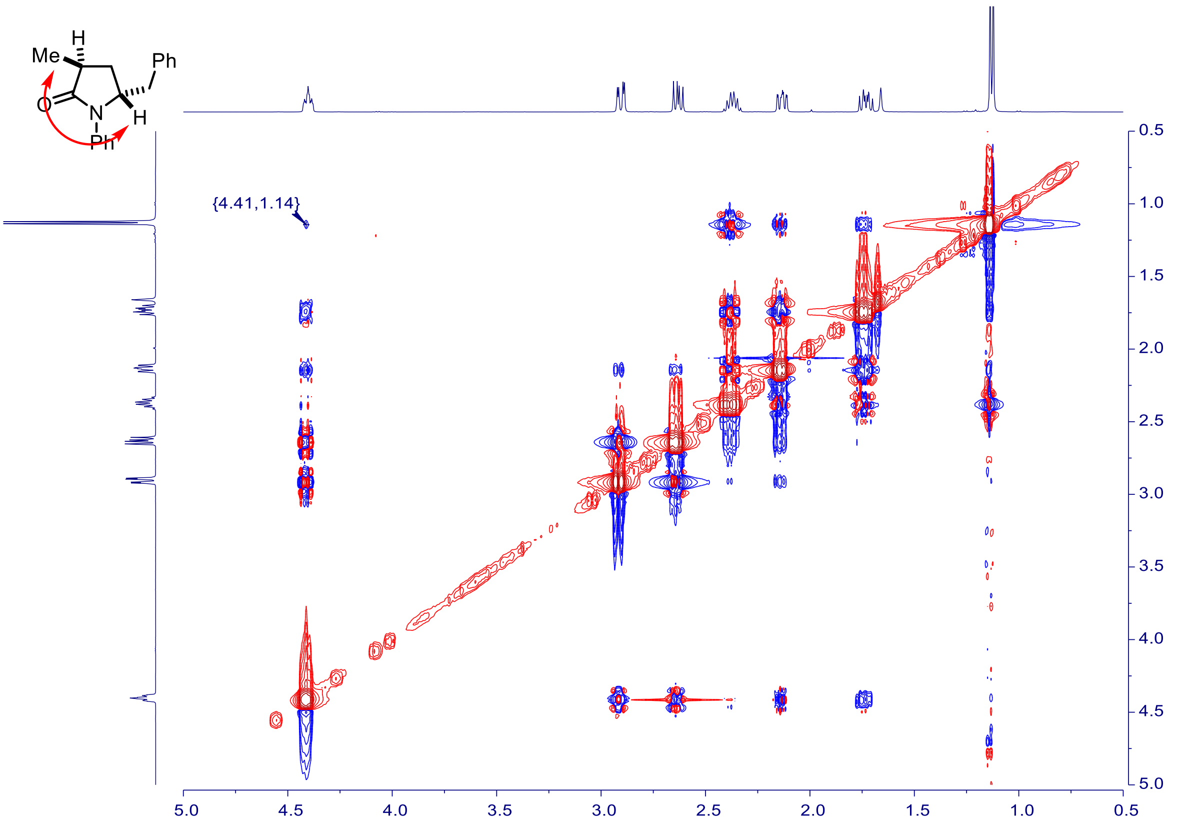

64 *cis* isomer – ^{1}H NMR (500 MHz, $CDCl_3$)

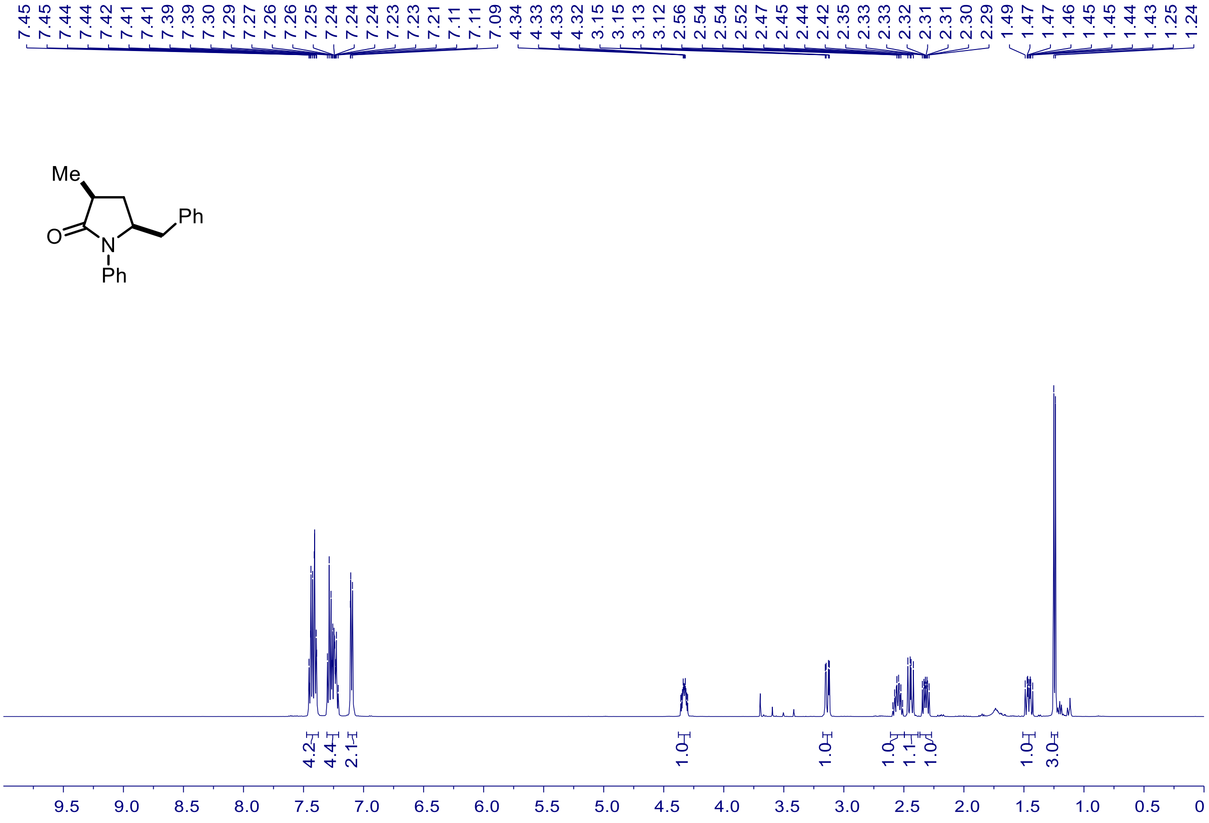

64 *cis* isomer – ^{13}C NMR (126 MHz, $CDCl_3$)

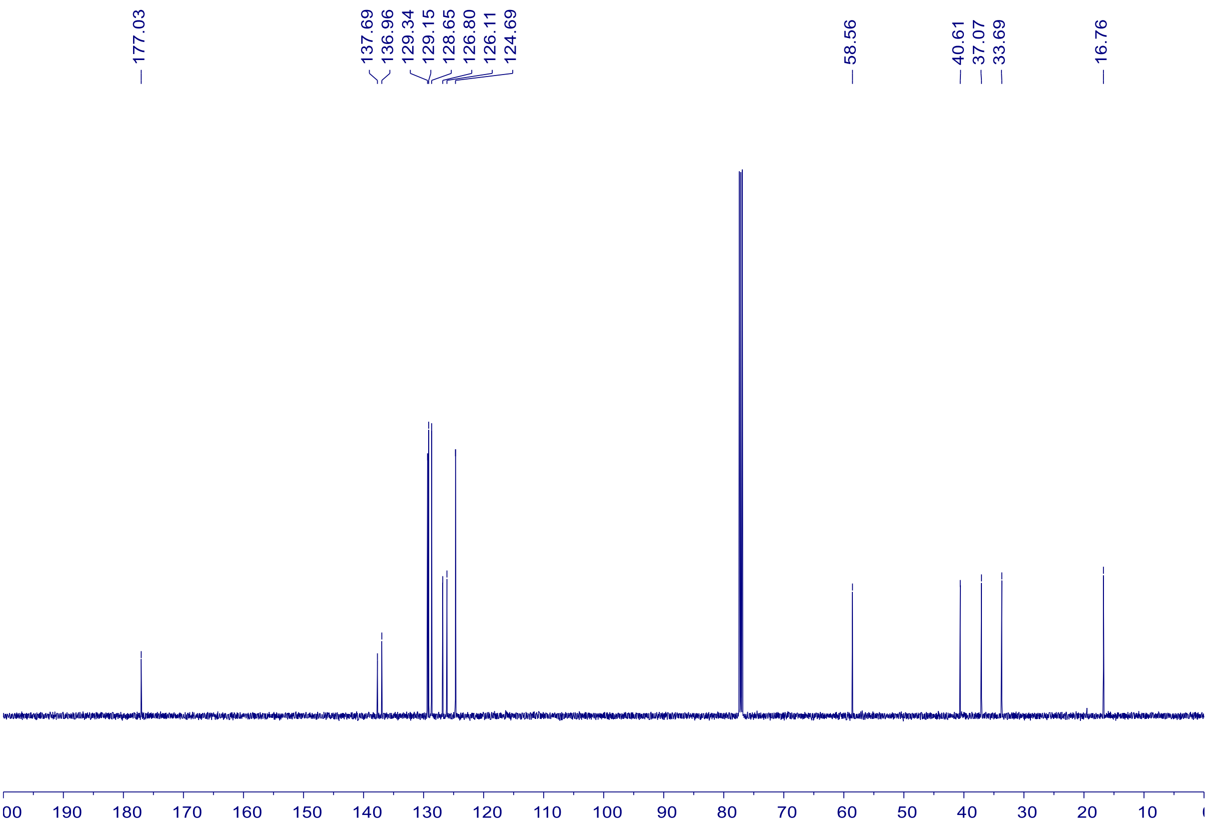

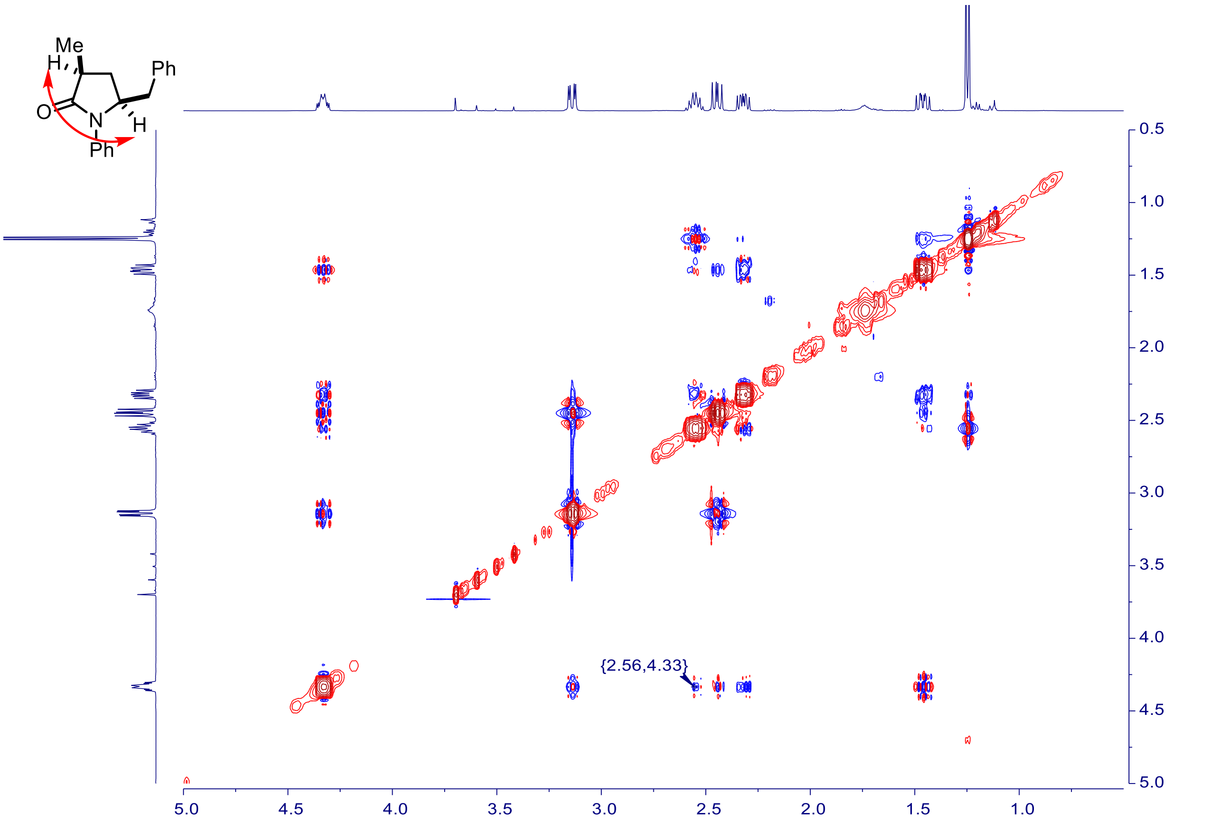

65 *trans* isomer – ^{1}H NMR (500 MHz, $CDCl_3$)

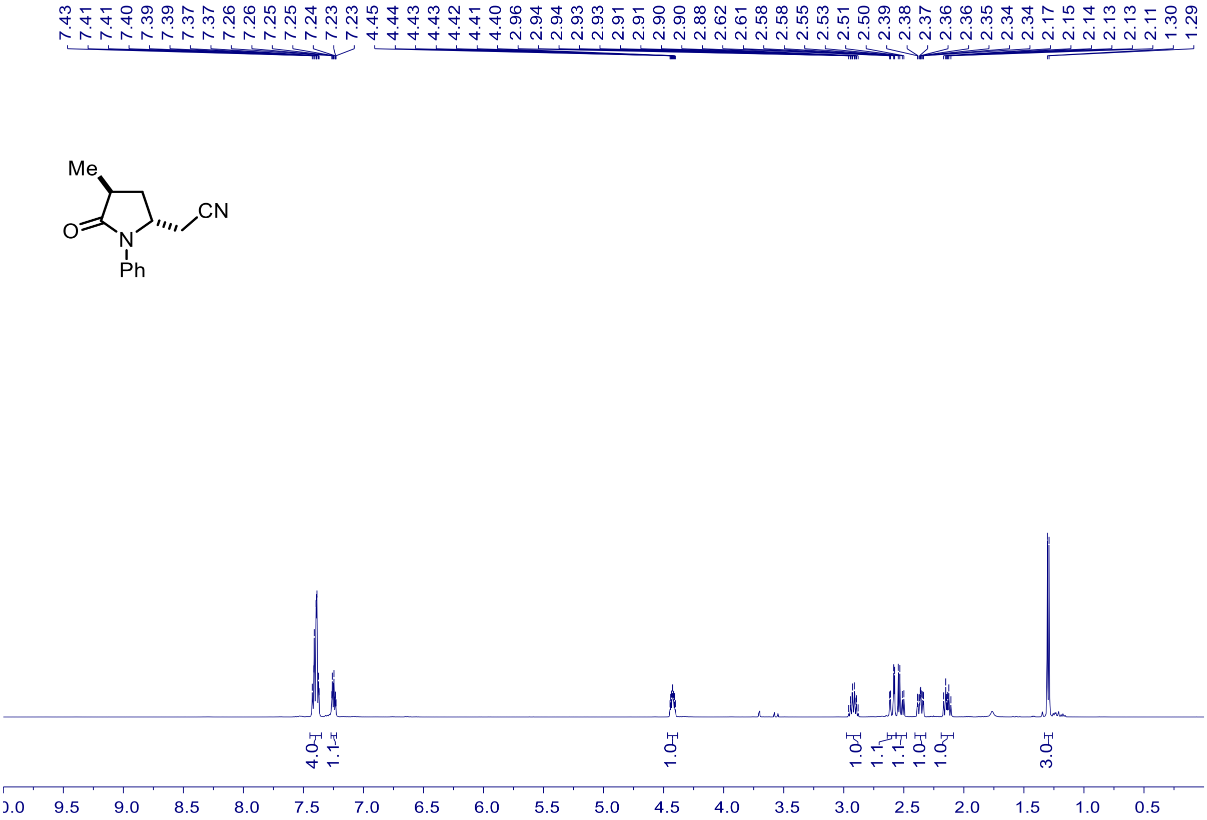

65 *trans* isomer – ^{13}C NMR (126 MHz, $CDCl_3$)

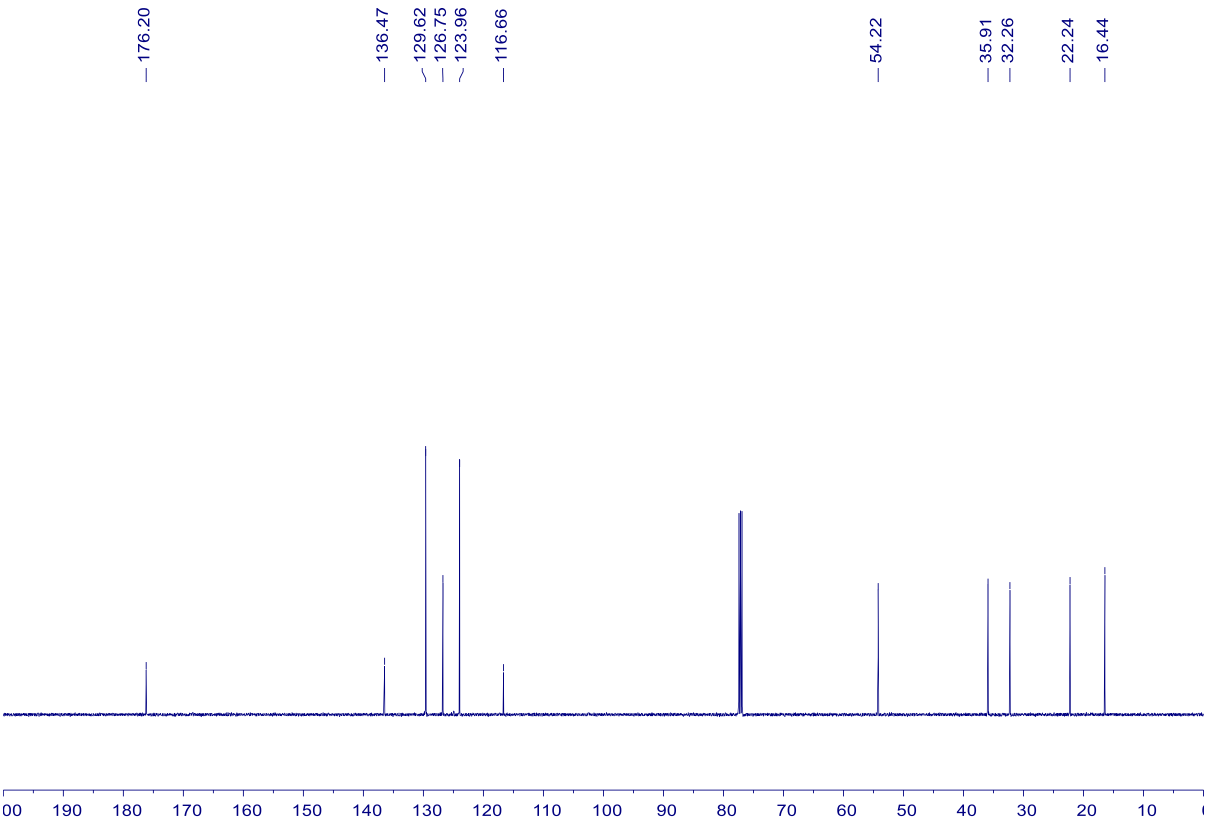

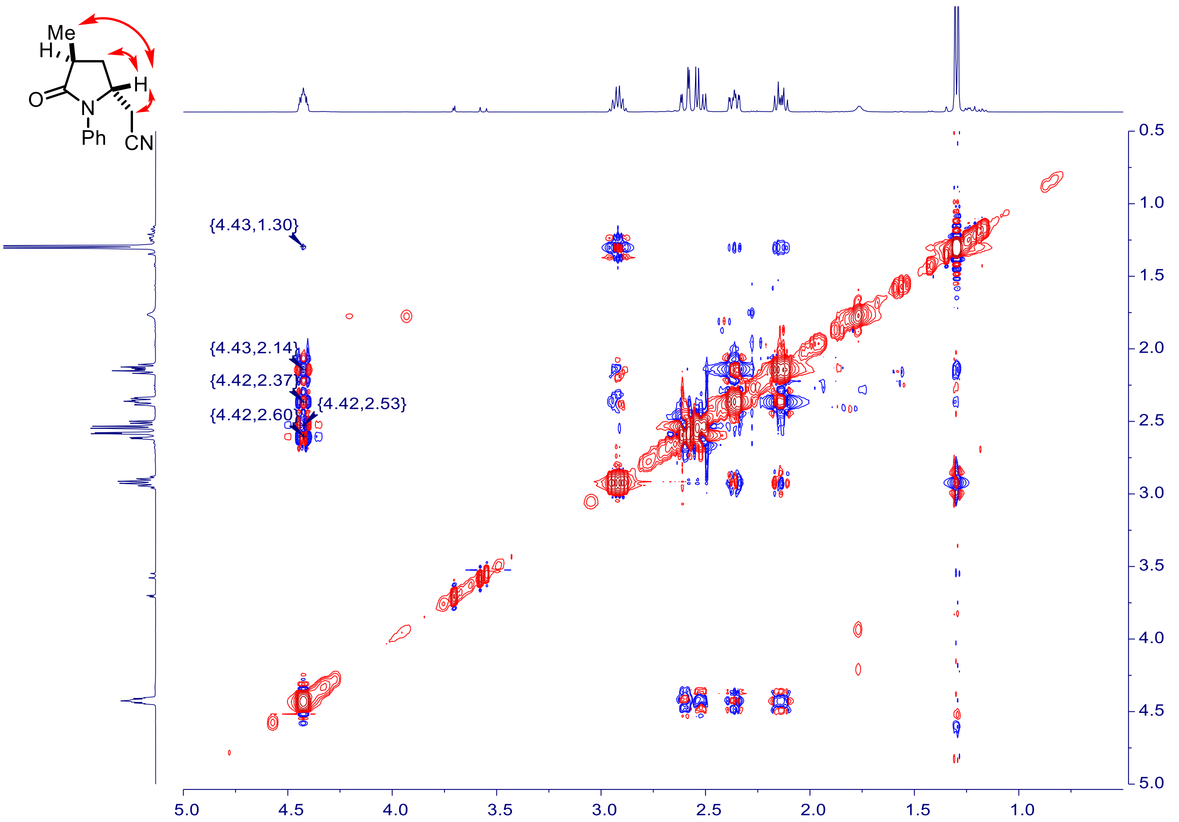

65 *cis* isomer – ^{1}H NMR (500 MHz, $CDCl_3$)

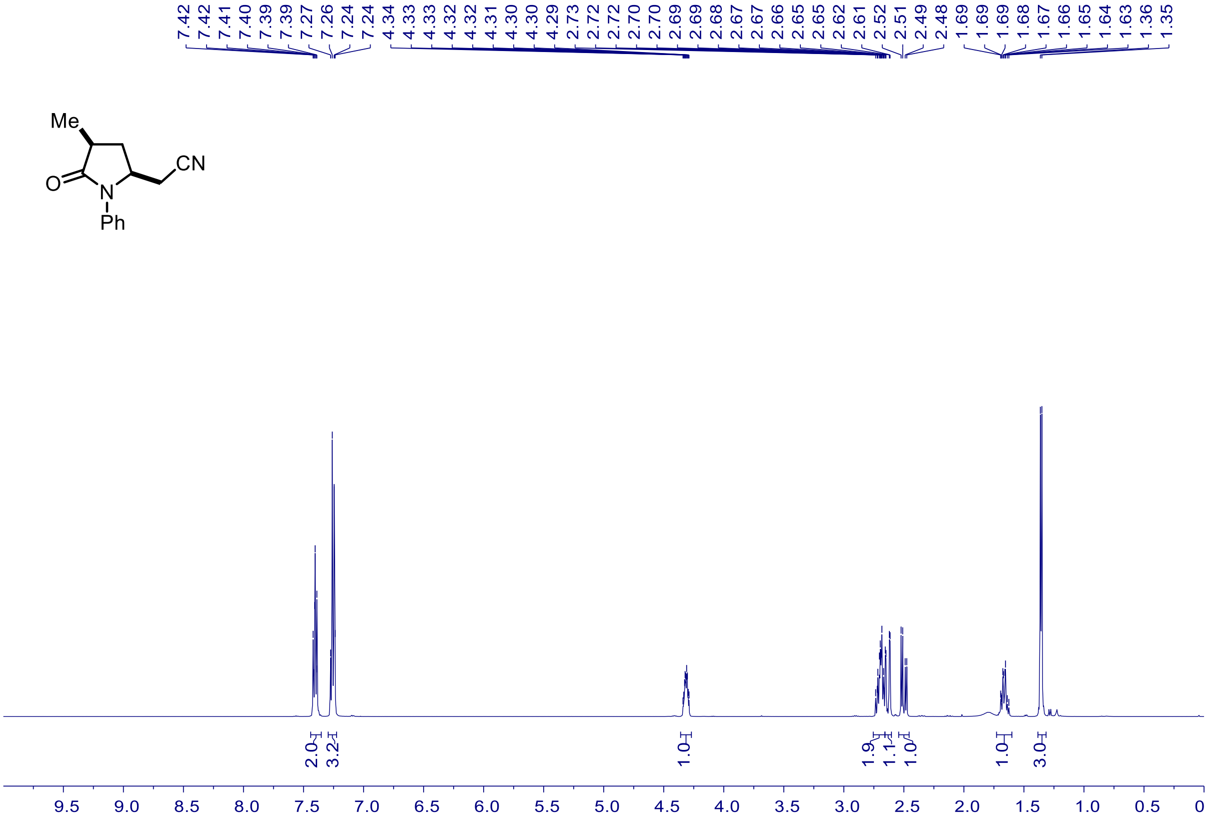

65 *cis* isomer – ^{13}C NMR (126 MHz, $CDCl_3$)

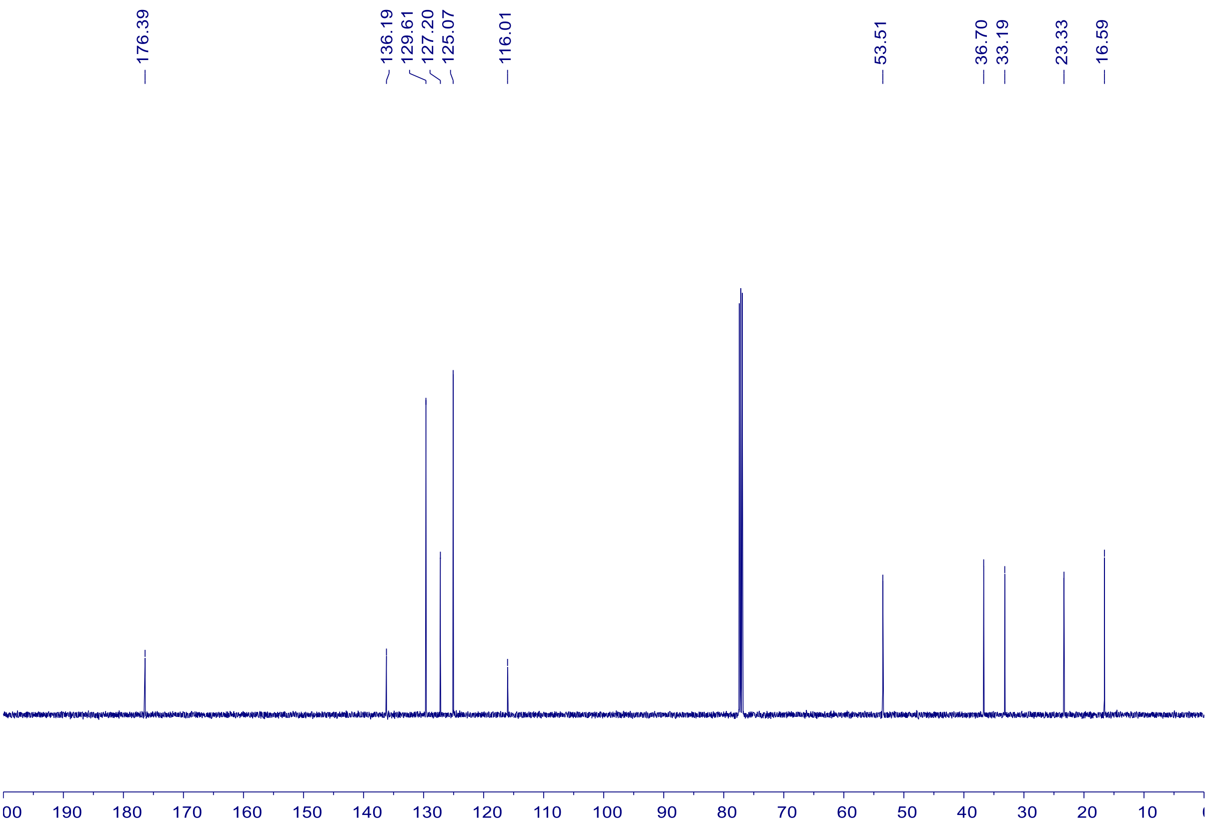

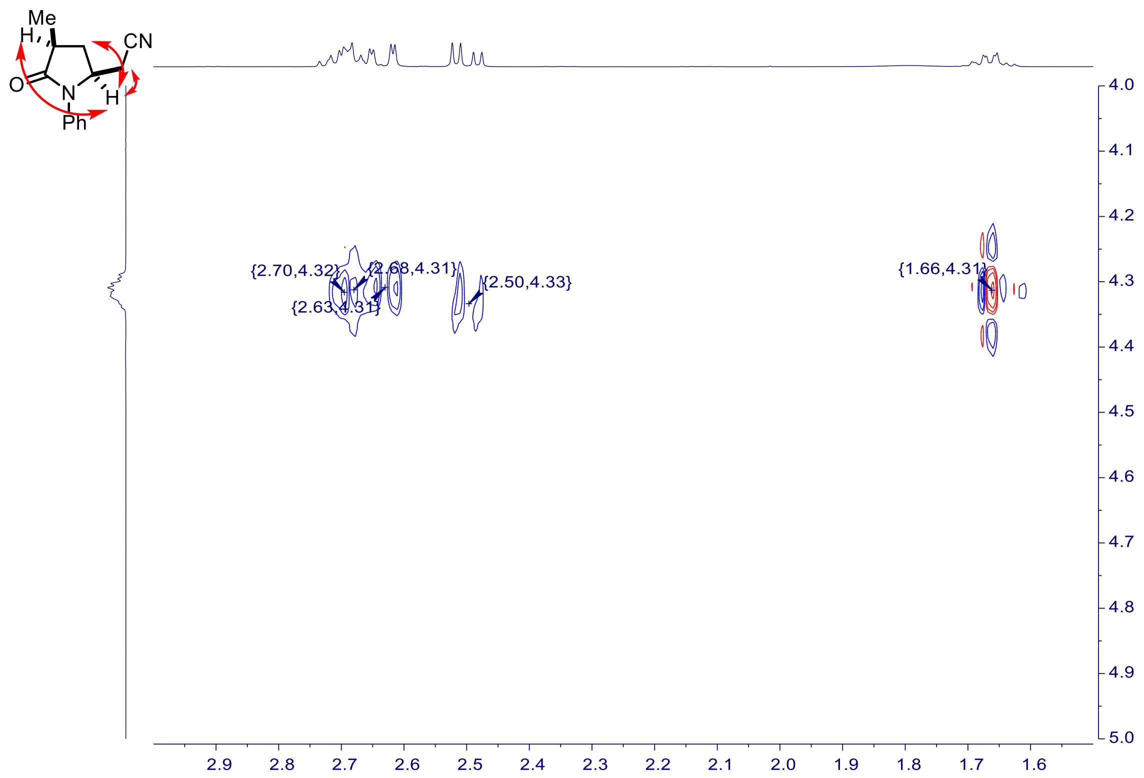

66 – ^{1}H NMR (500 MHz, $CDCl_3$)

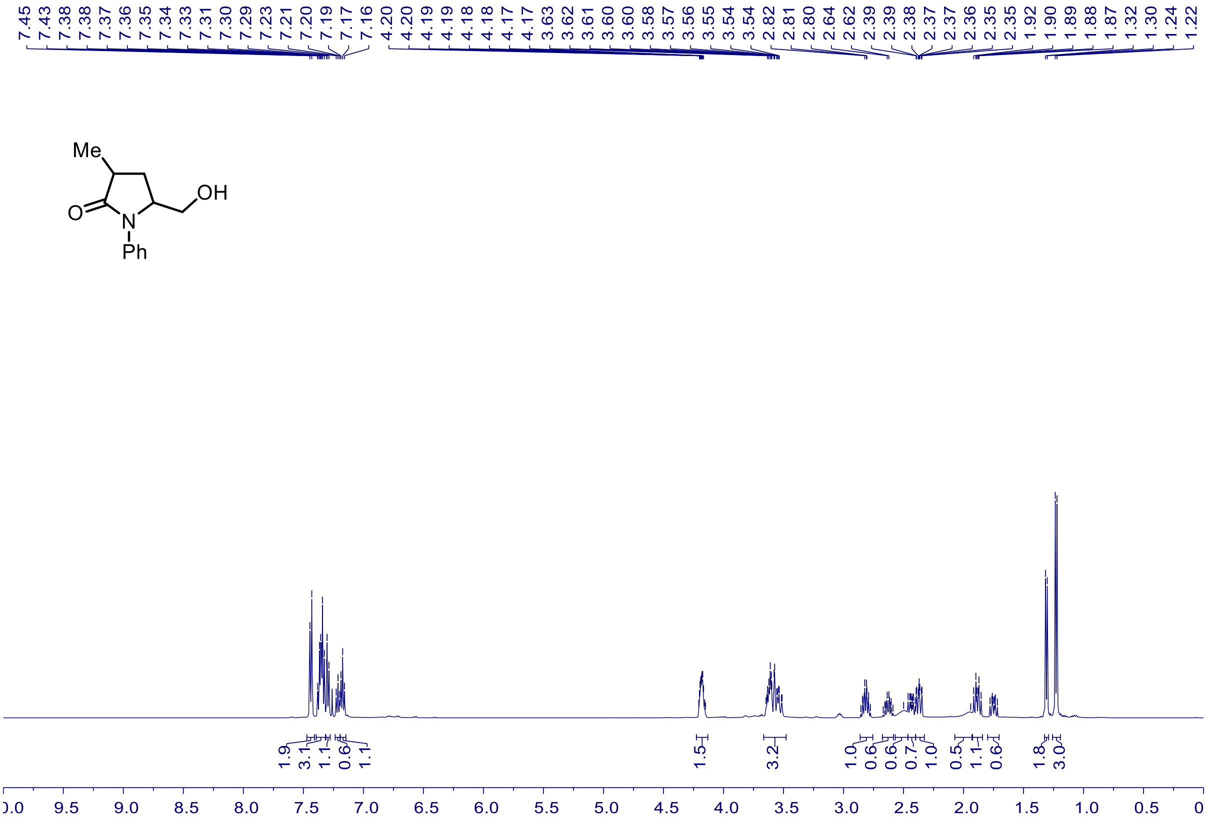

66 – ^{13}C NMR (126 MHz, $CDCl_3$)

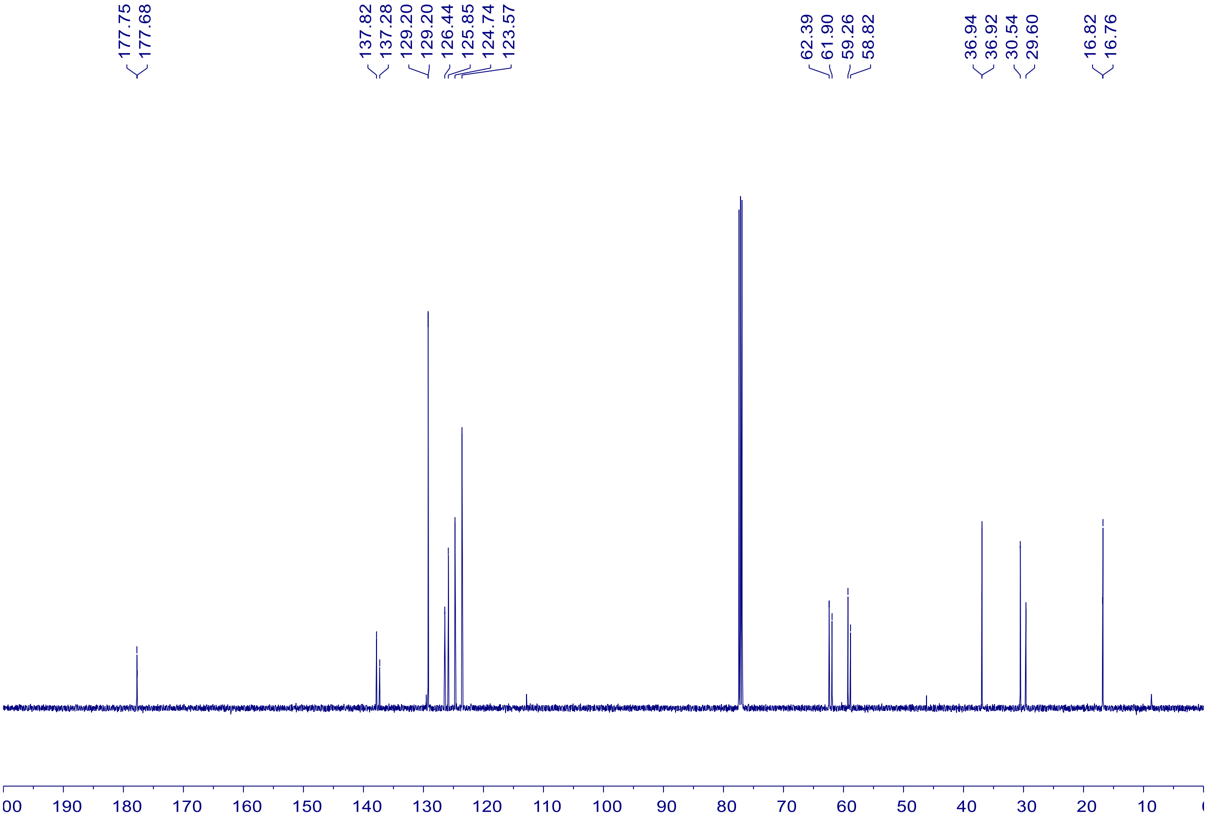

67 *trans* isomer – ^{1}H NMR (500 MHz, $CDCl_3$)

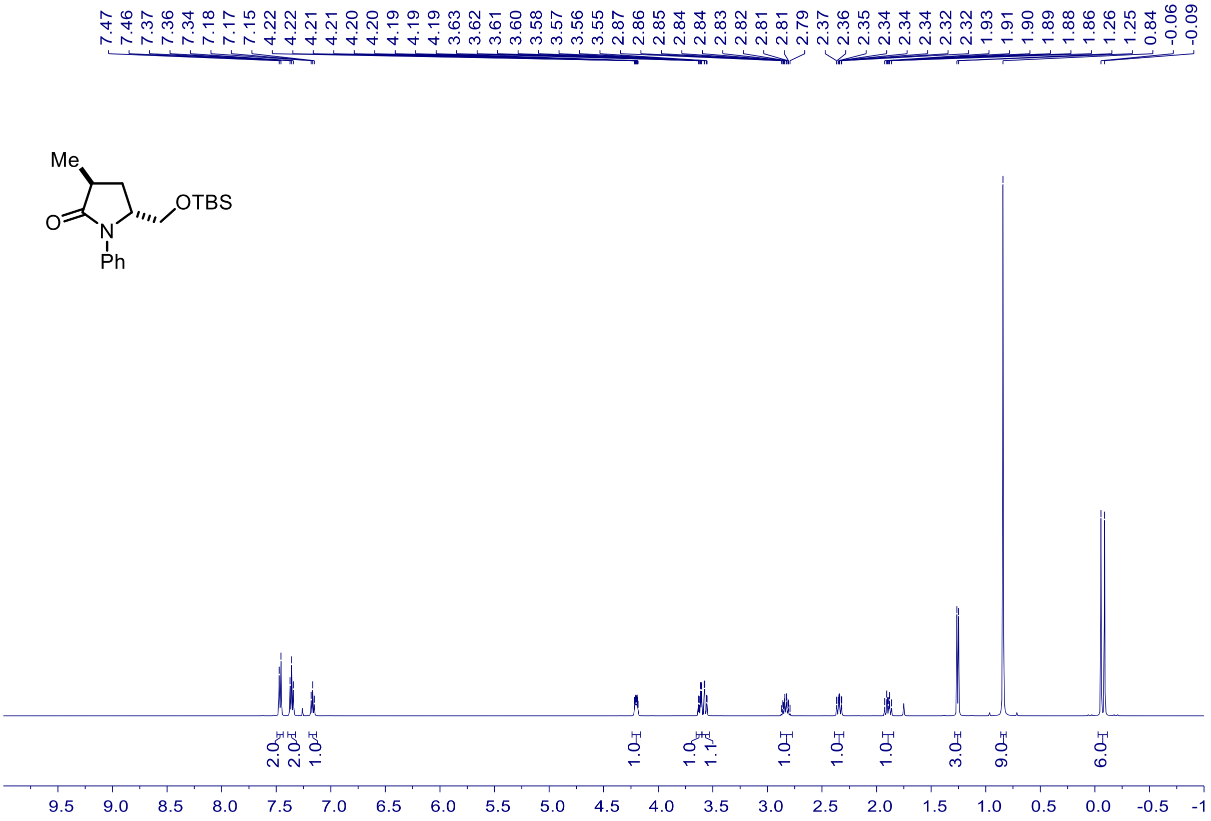

67 *trans* isomer – ^{13}C NMR (126 MHz, $CDCl_3$)

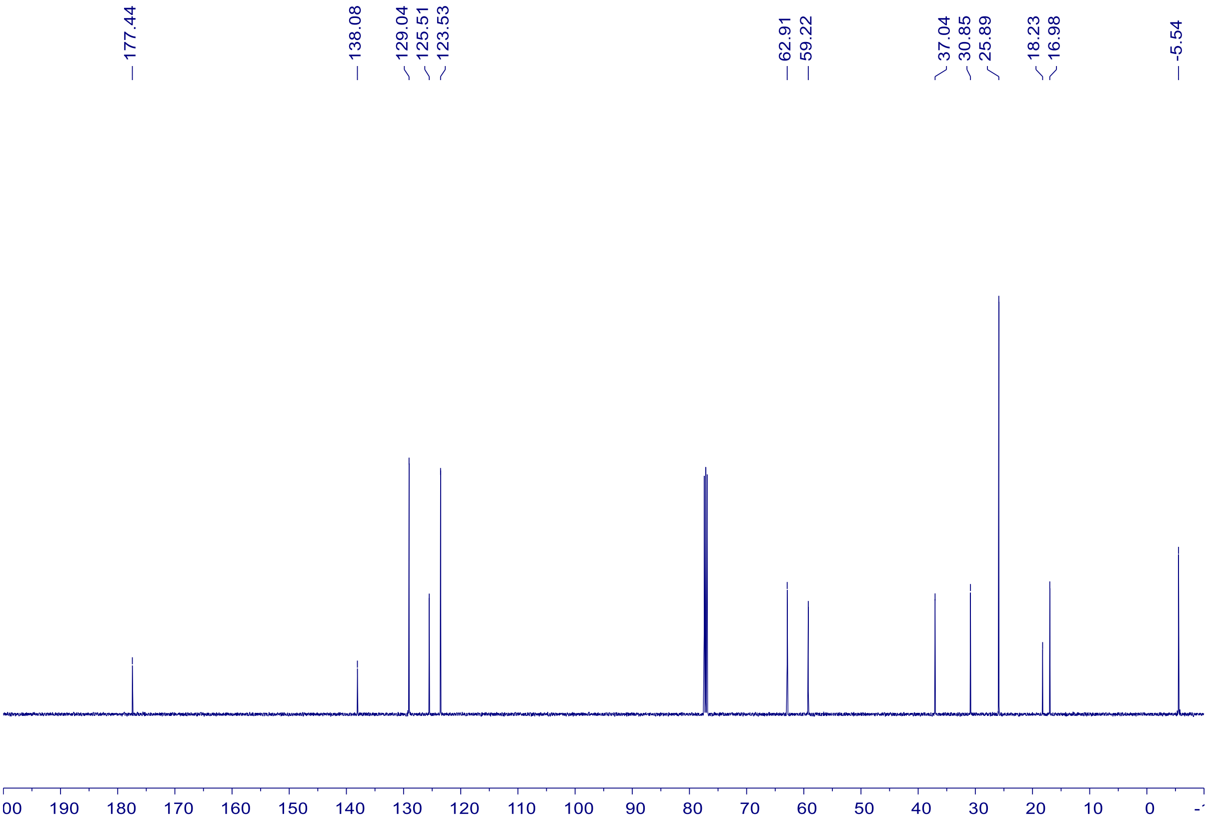

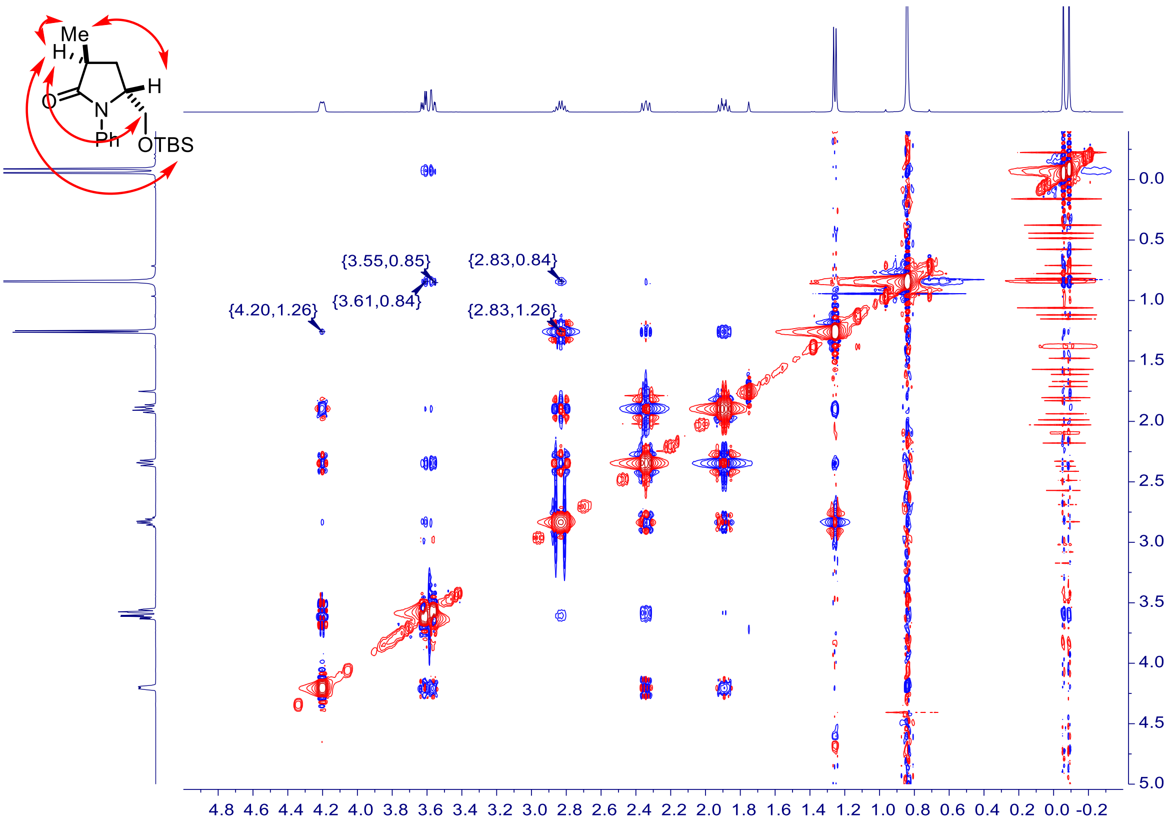

67 *cis* isomer – ^{1}H NMR (500 MHz, $CDCl_3$)

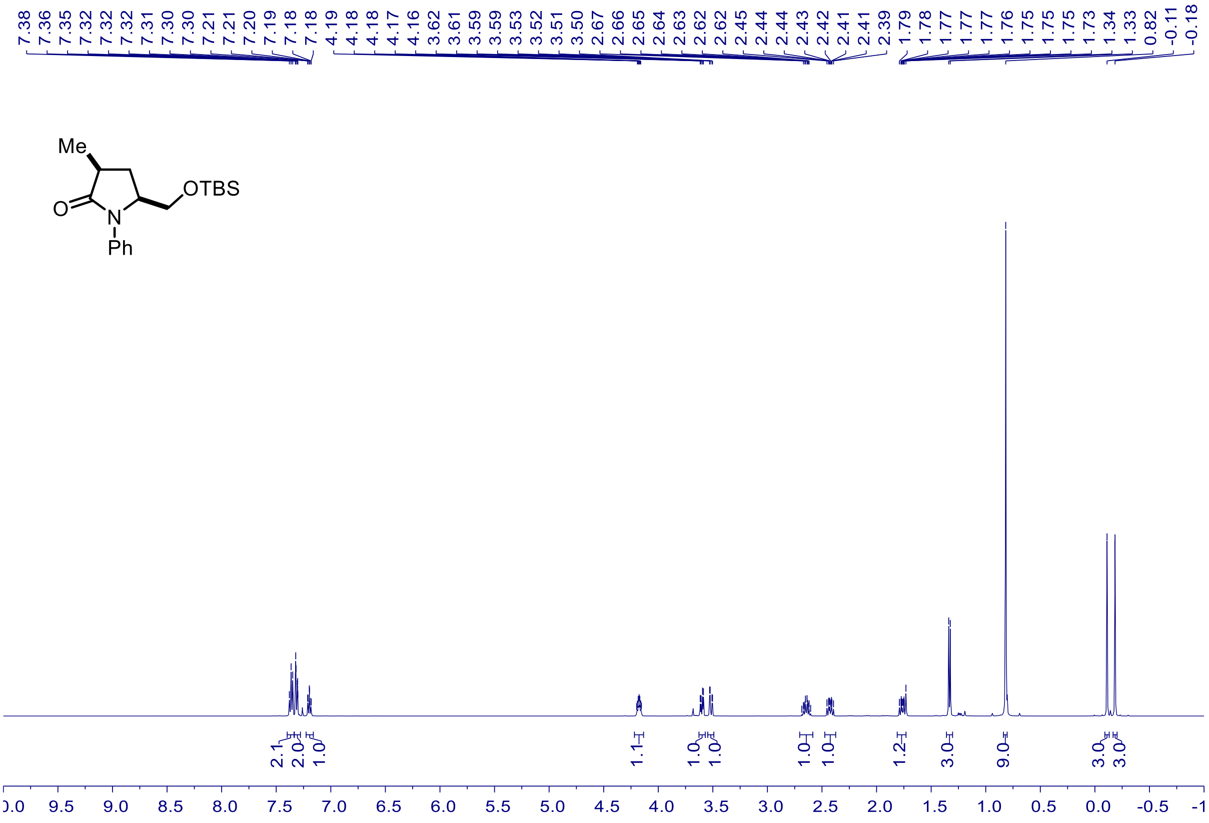

67 *cis* isomer – ^{13}C NMR (126 MHz, CDCl$_3$)

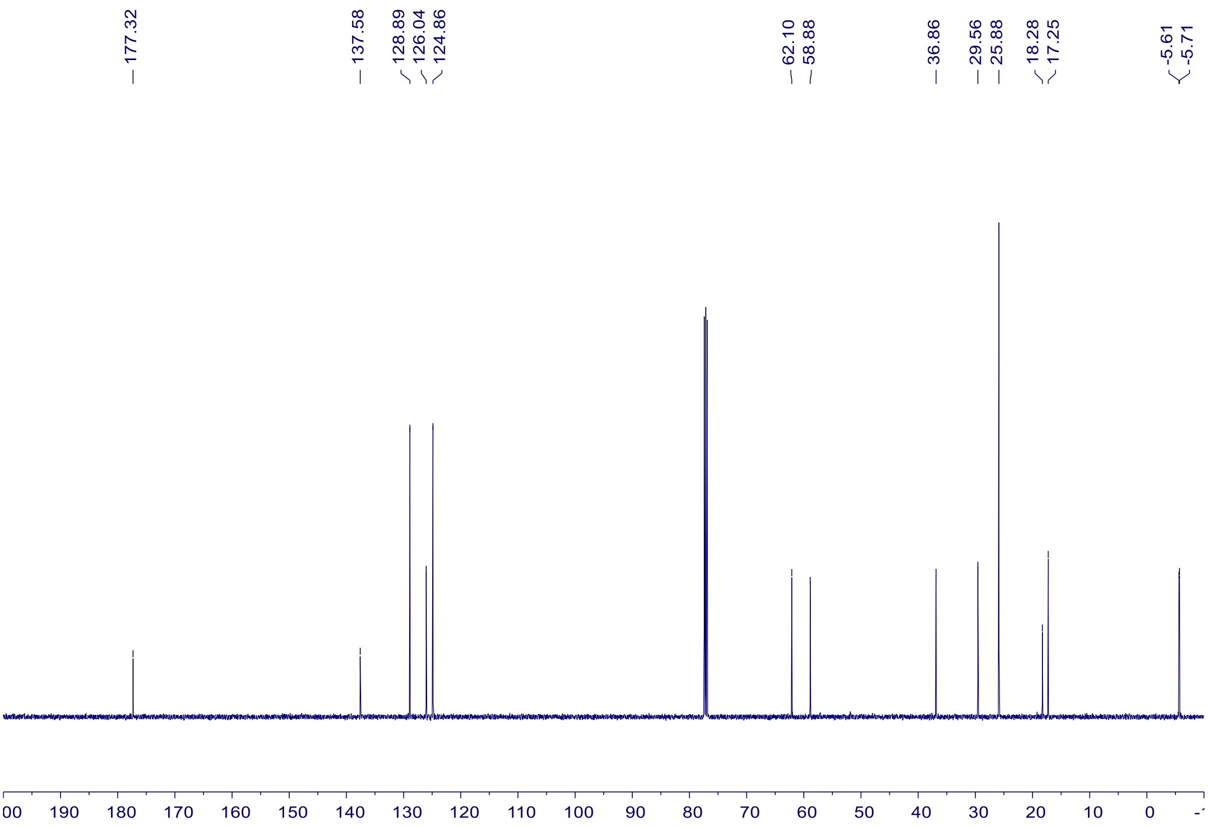

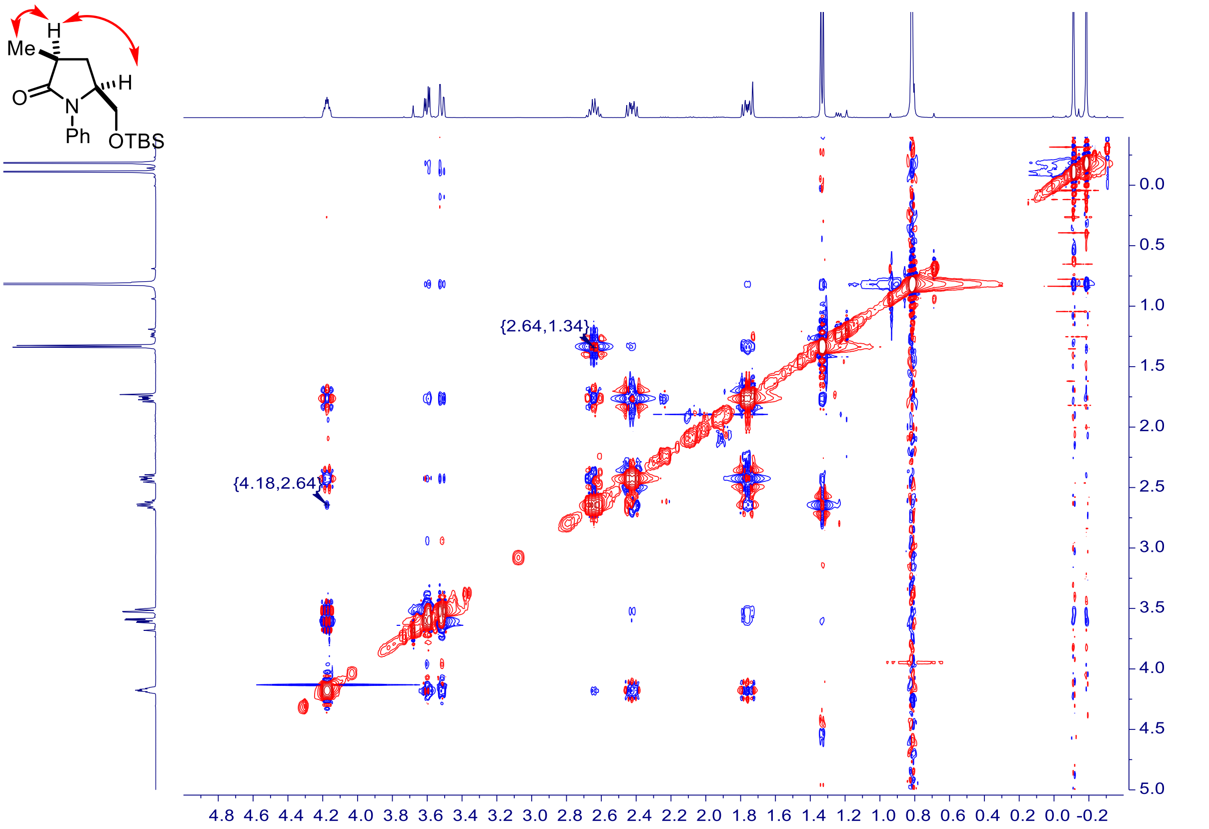

68 *trans* isomer – ^{1}H NMR (500 MHz, $CDCl_3$)

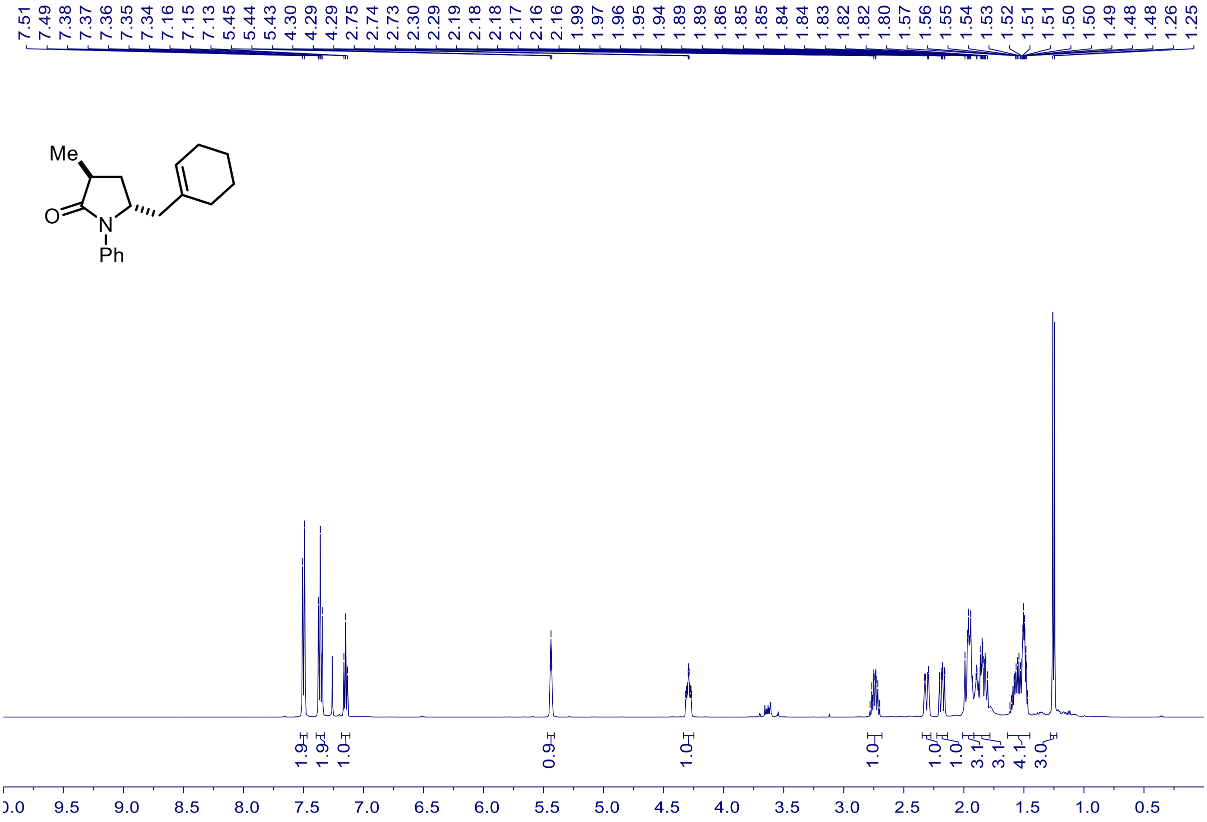

68 *trans* isomer – ^{13}C NMR (126 MHz, $CDCl_3$)

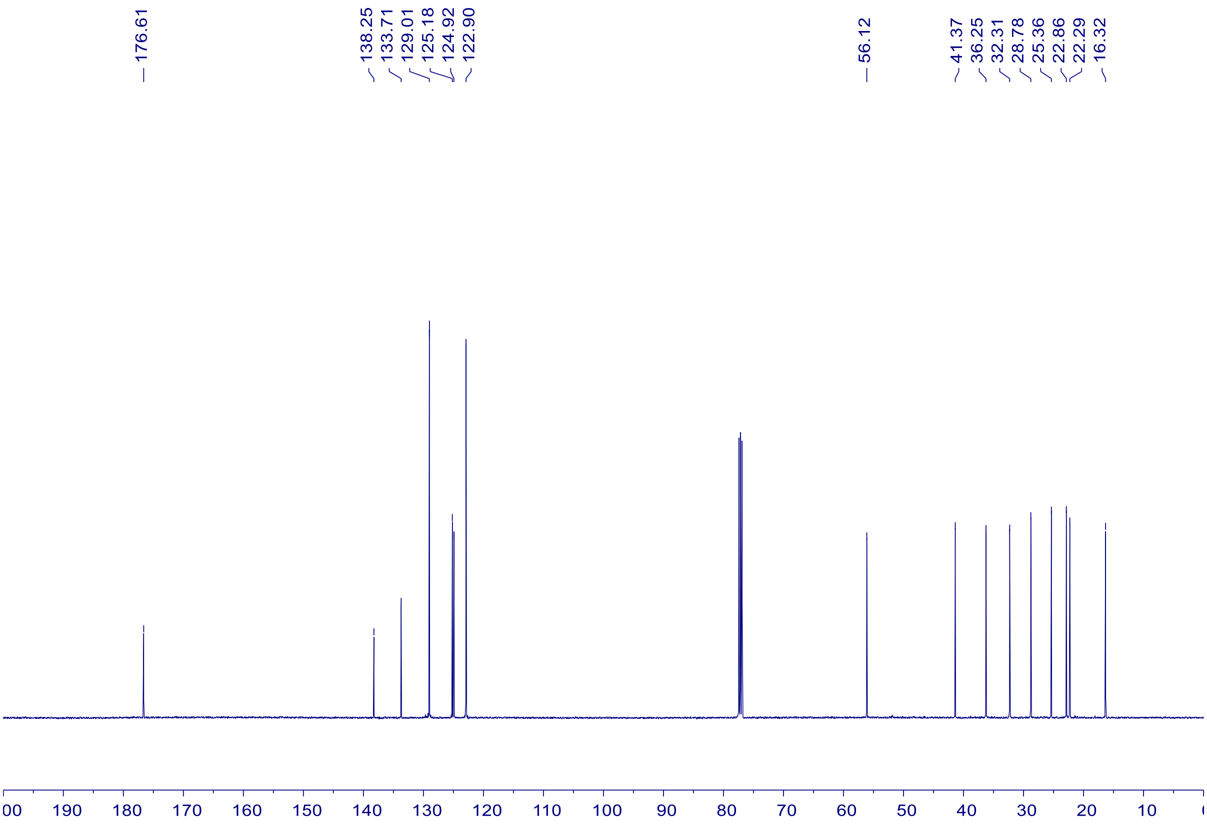

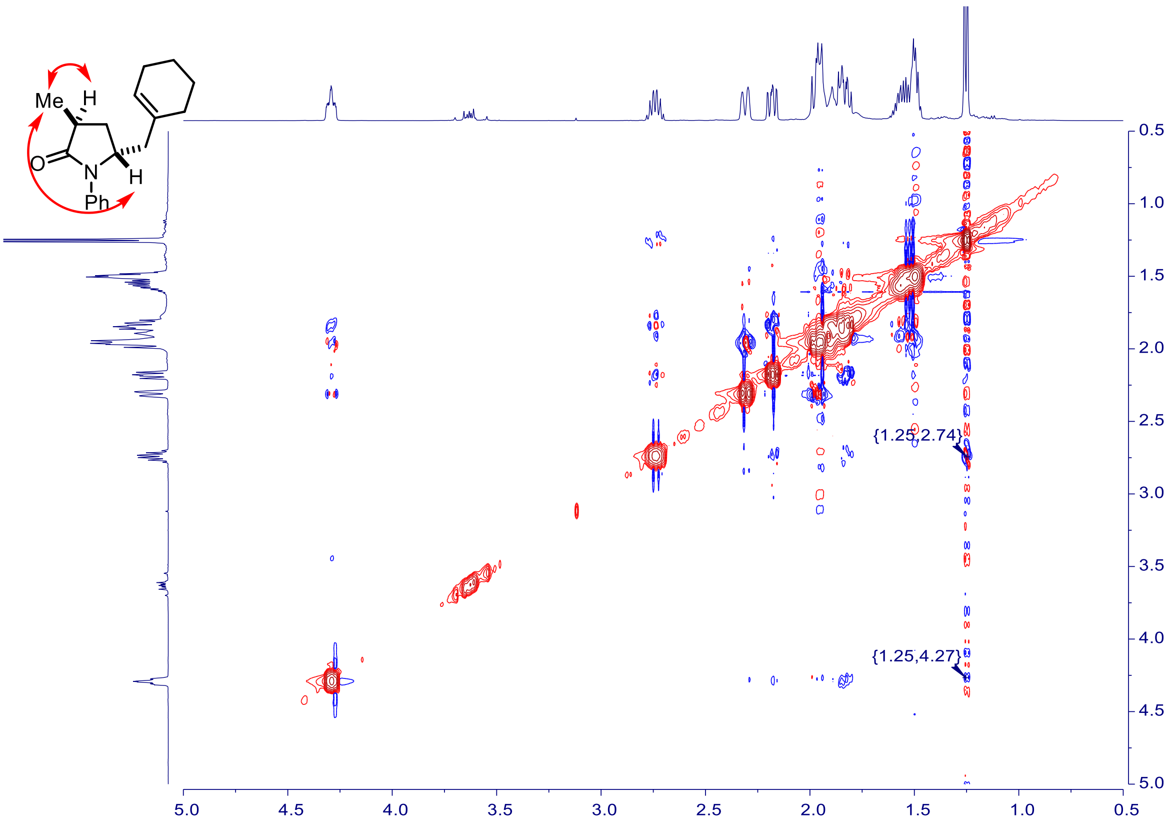

68 *cis* isomer – ^{1}H NMR (500 MHz, $CDCl_3$)

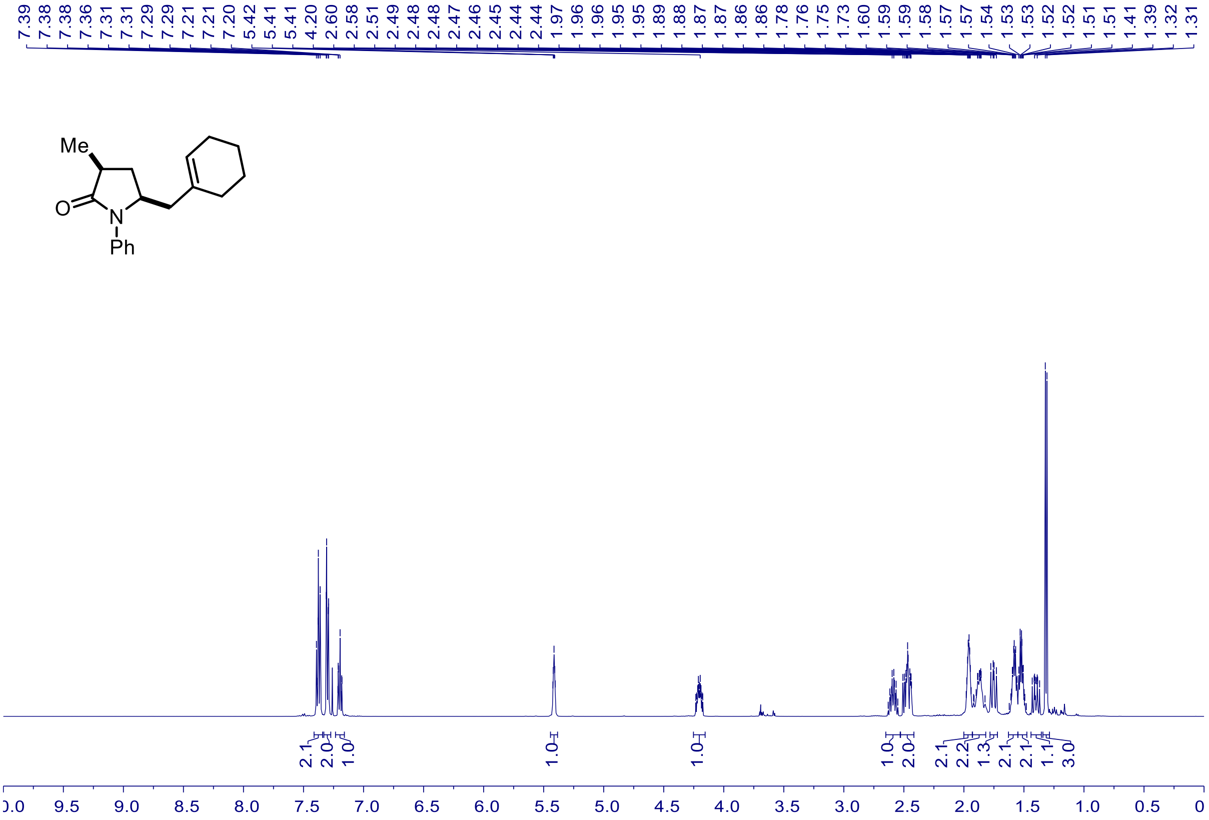

68 *cis* isomer – ^{13}C NMR (126 MHz, $CDCl_3$)

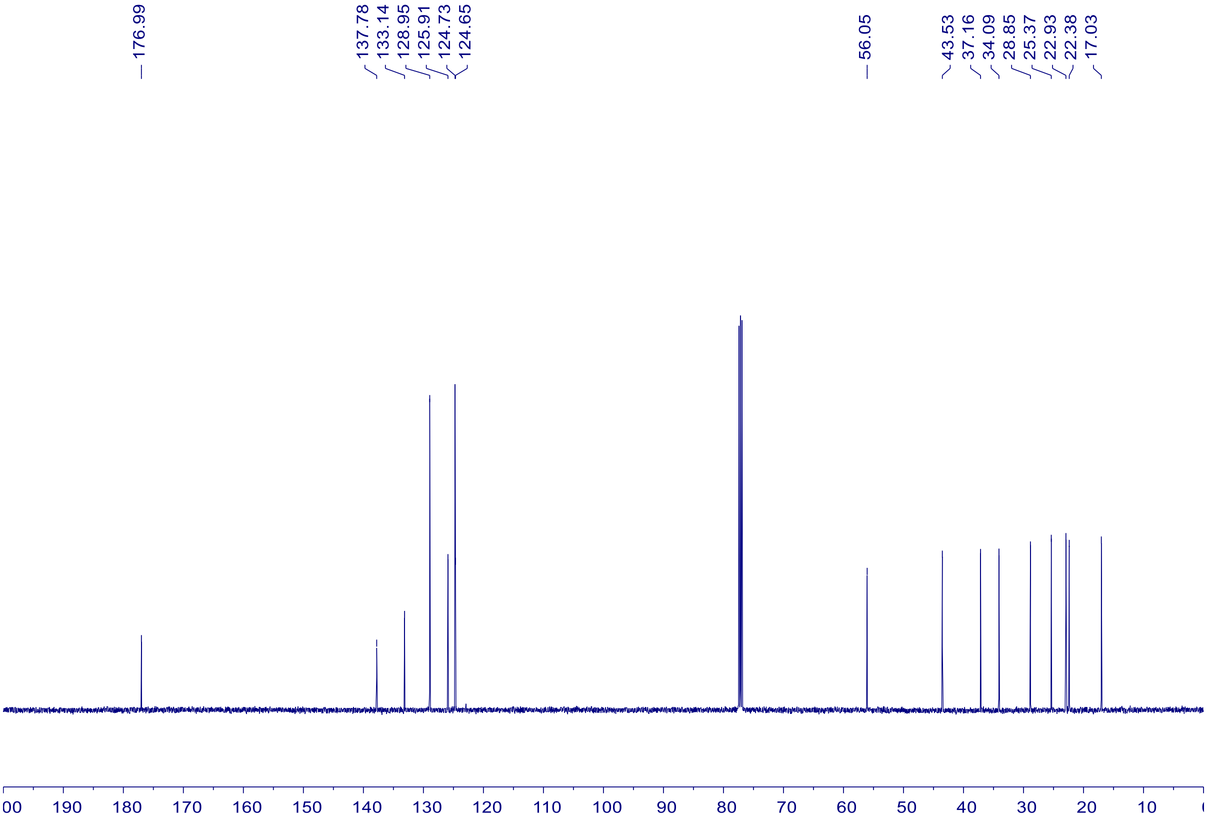

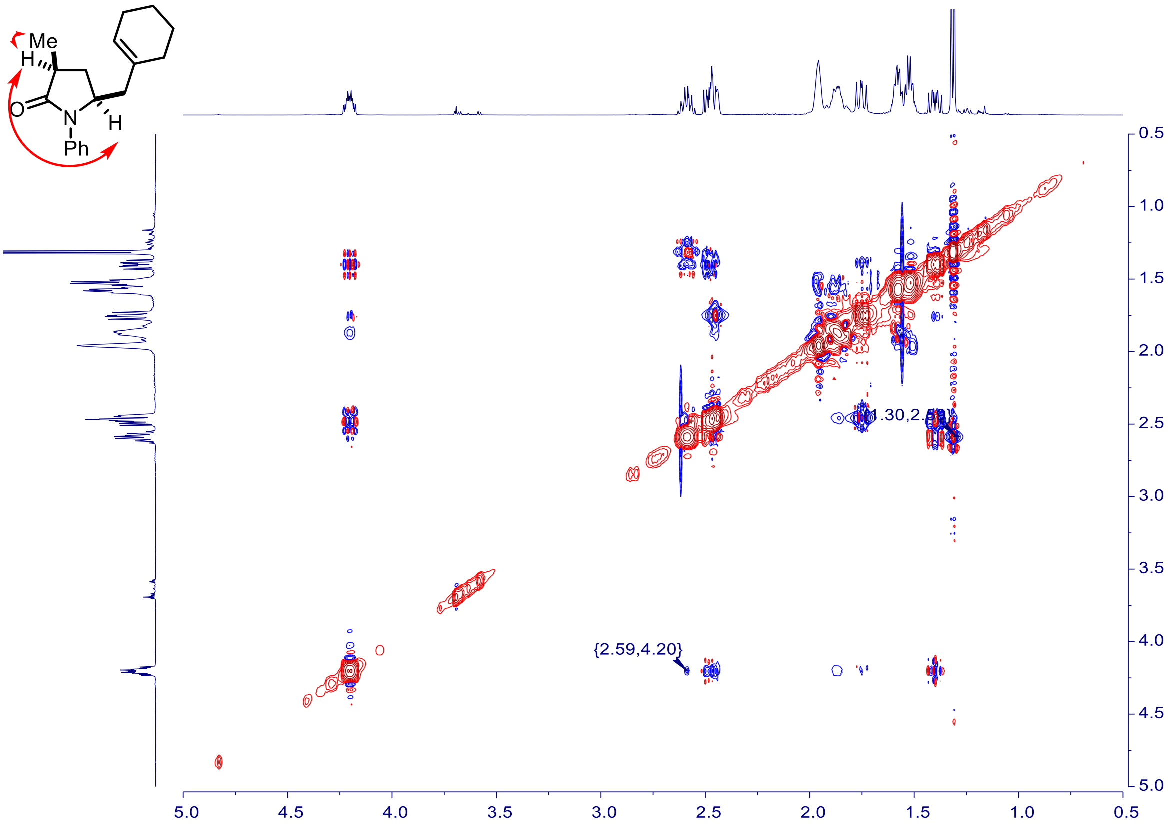

69 *trans* isomer – ^{1}H NMR (500 MHz, $CDCl_3$)

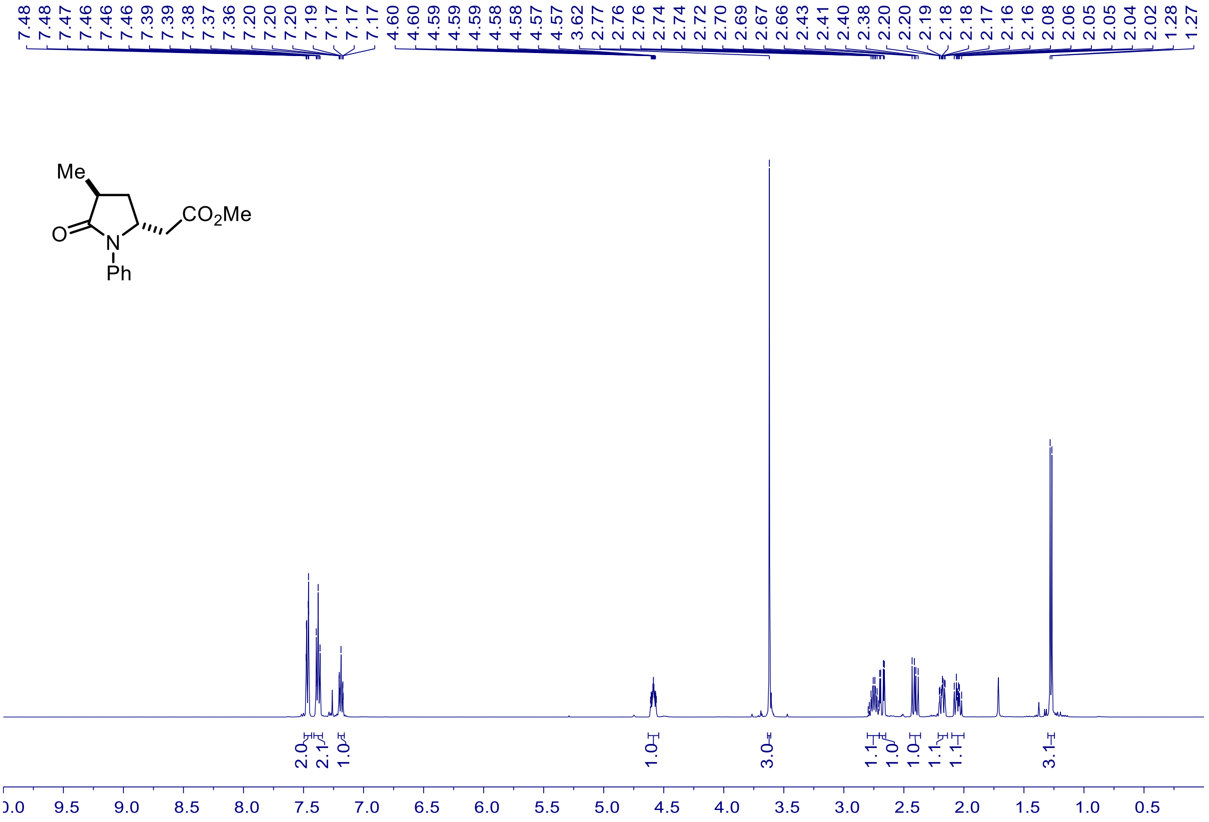

69 *trans* isomer – ^{13}C NMR (126 MHz, $CDCl_3$)

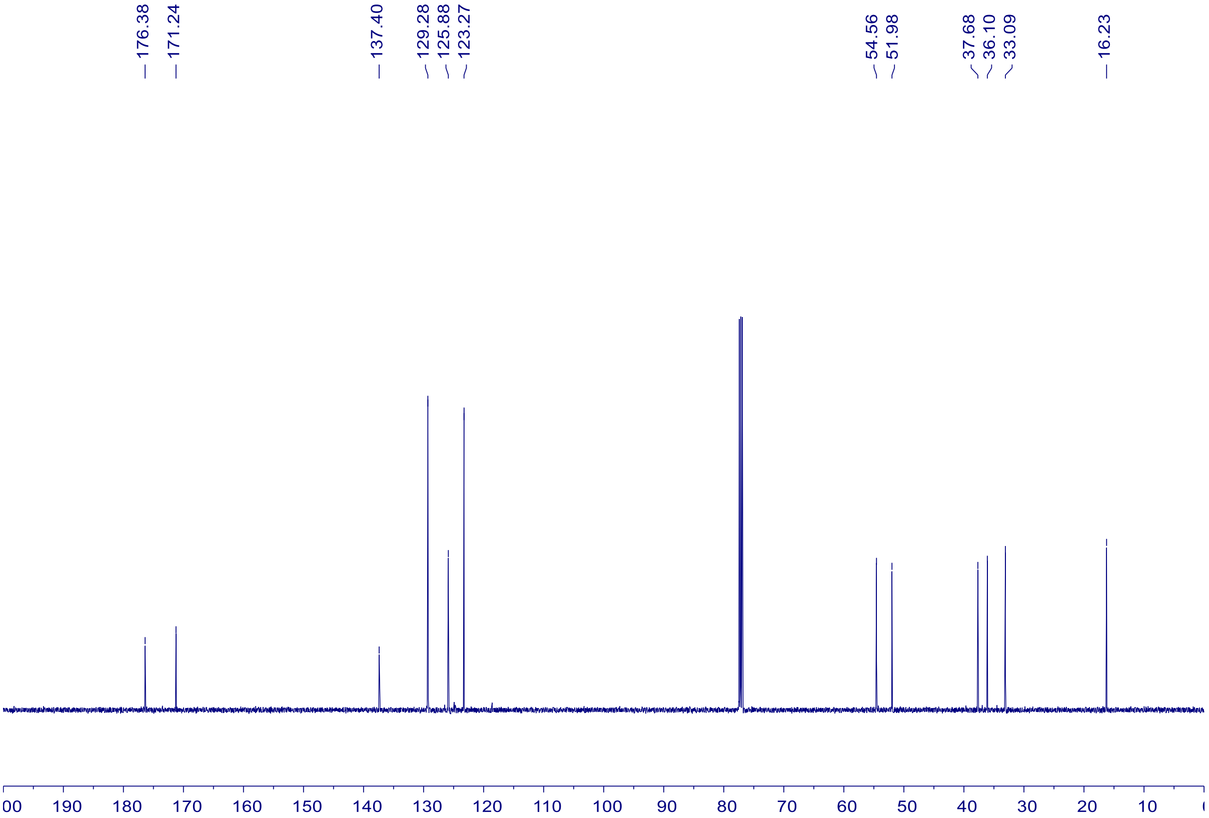

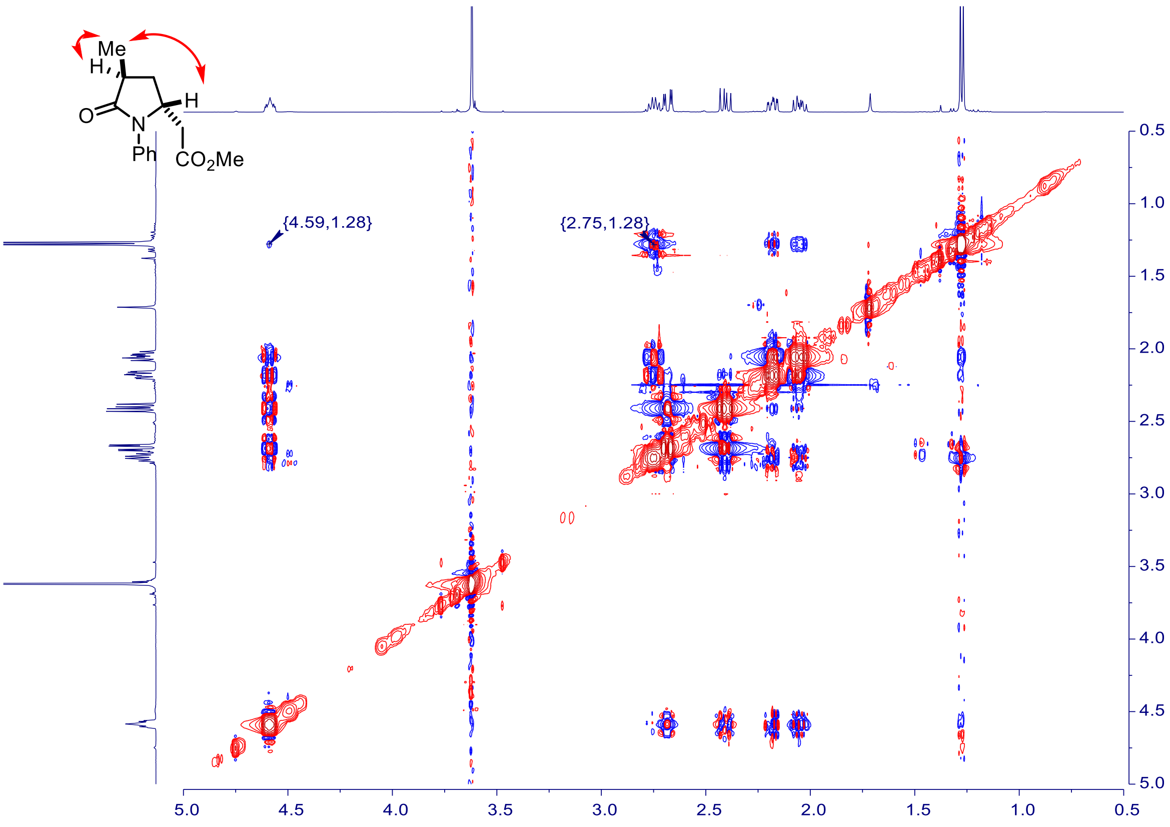

69 *cis* isomer – ^{1}H NMR (500 MHz, $CDCl_3$)

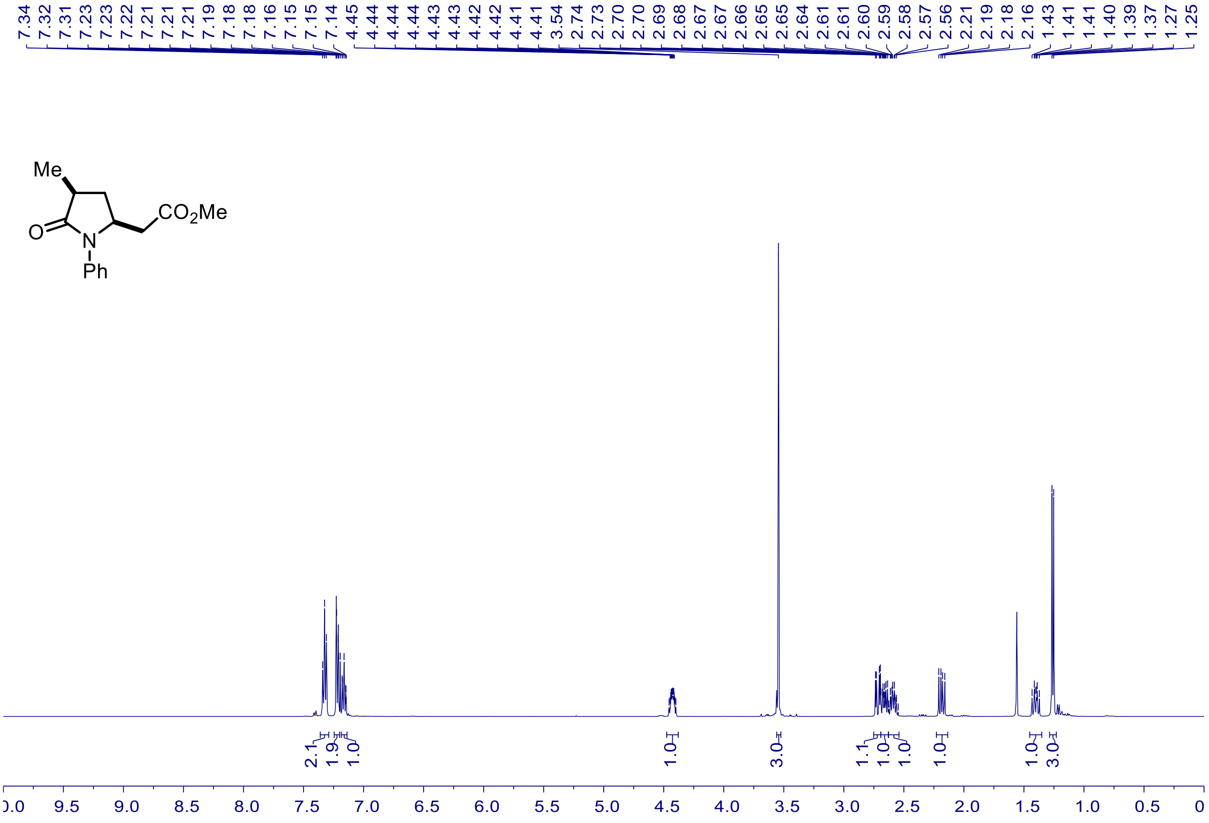

69 *cis* isomer – ^{13}C NMR (126 MHz, $CDCl_3$)

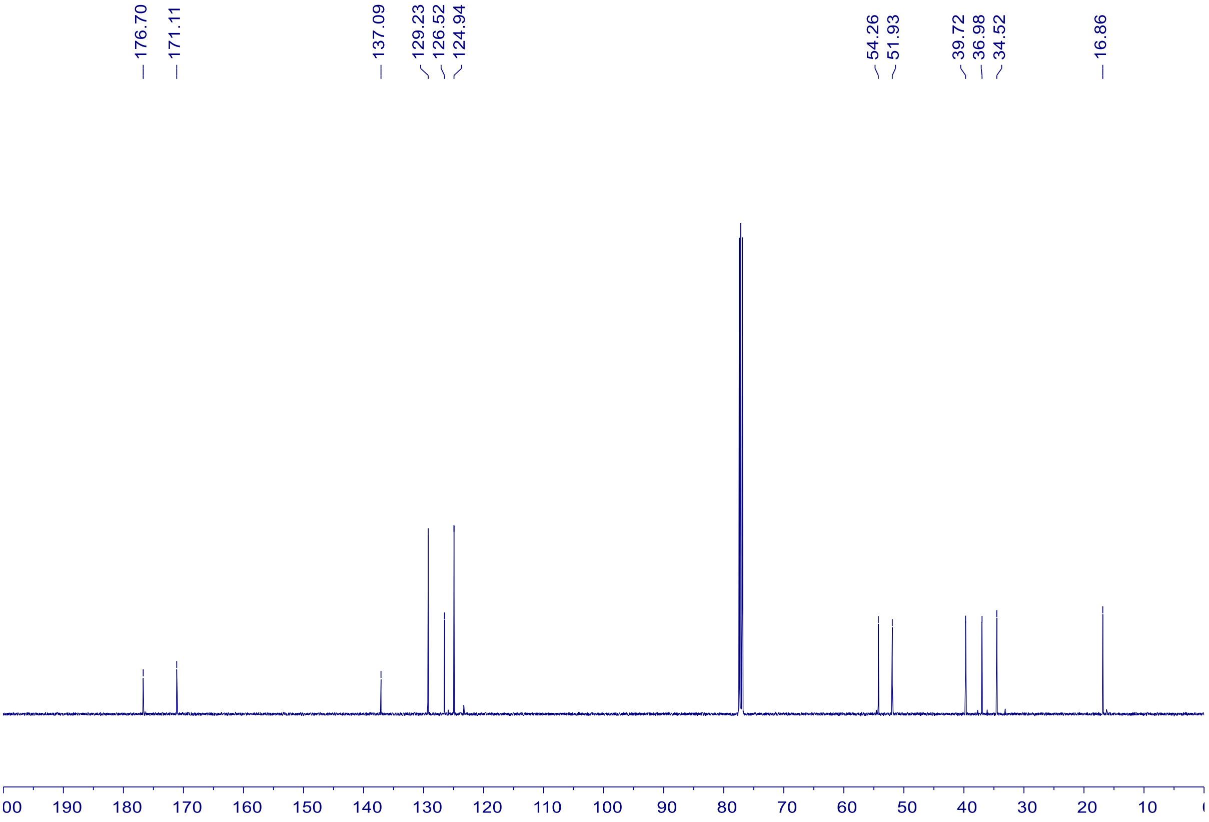

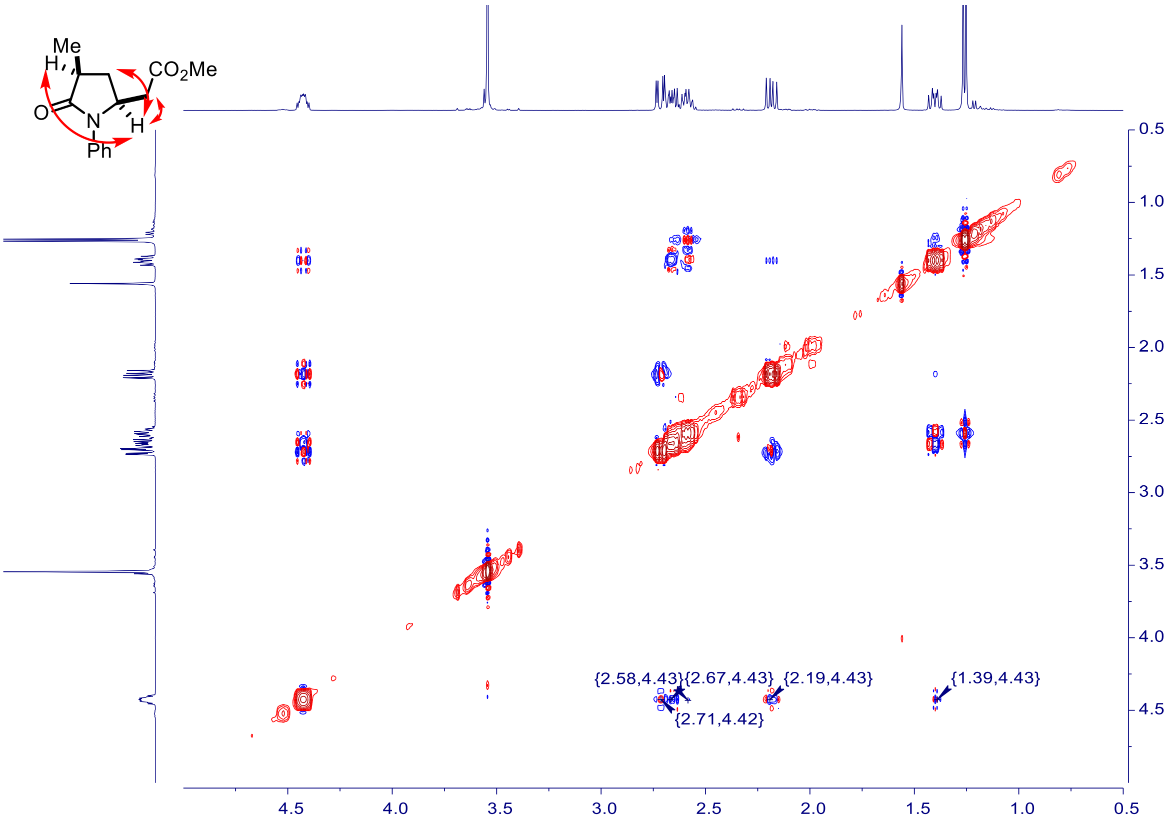

71 – ^{1}H NMR (400 MHz, CDCl$_3$)

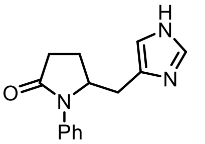

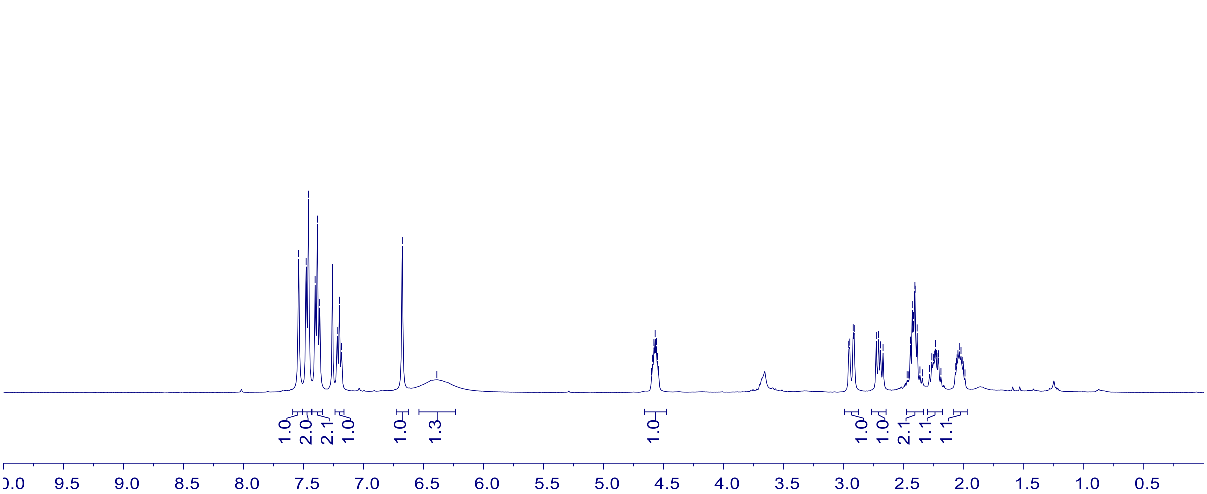

71 – ^{13}C NMR (101 MHz, CDCl$_3$)

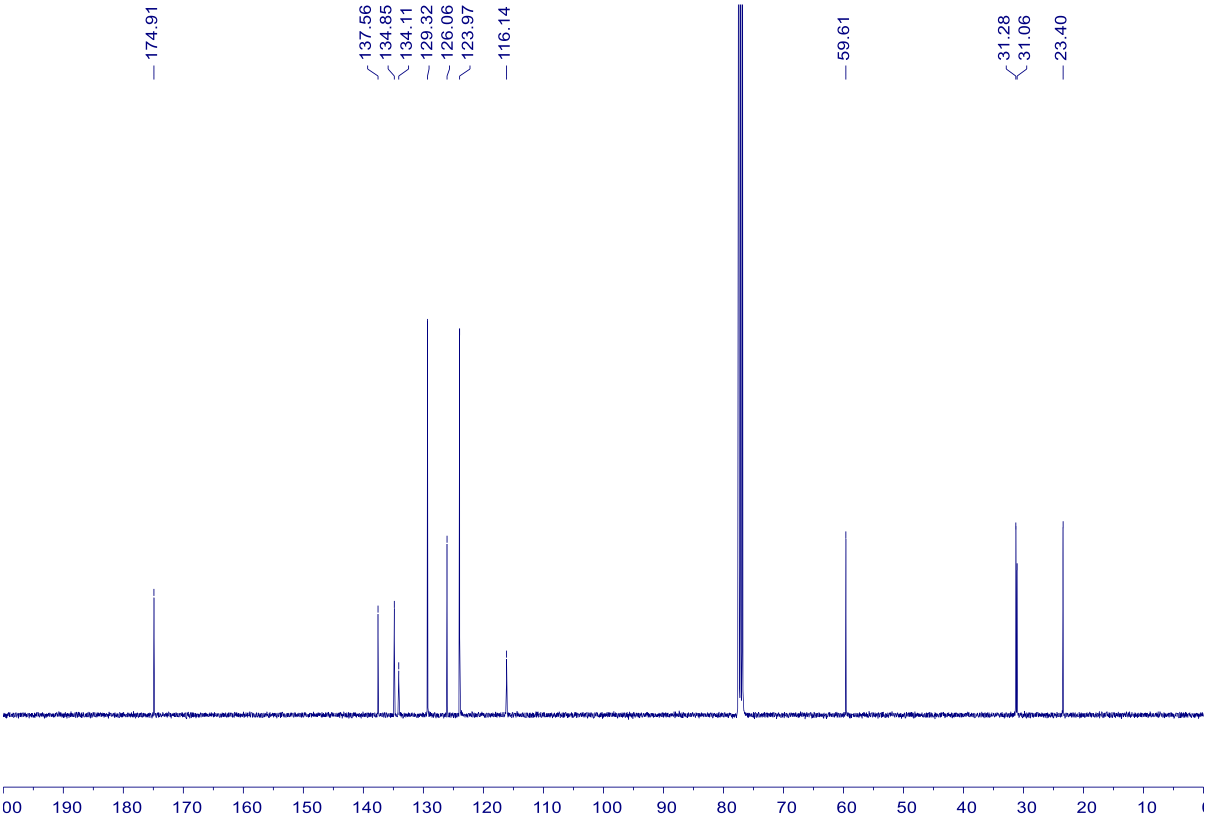

72 – ^{1}H NMR (500 MHz, $CDCl_3$)

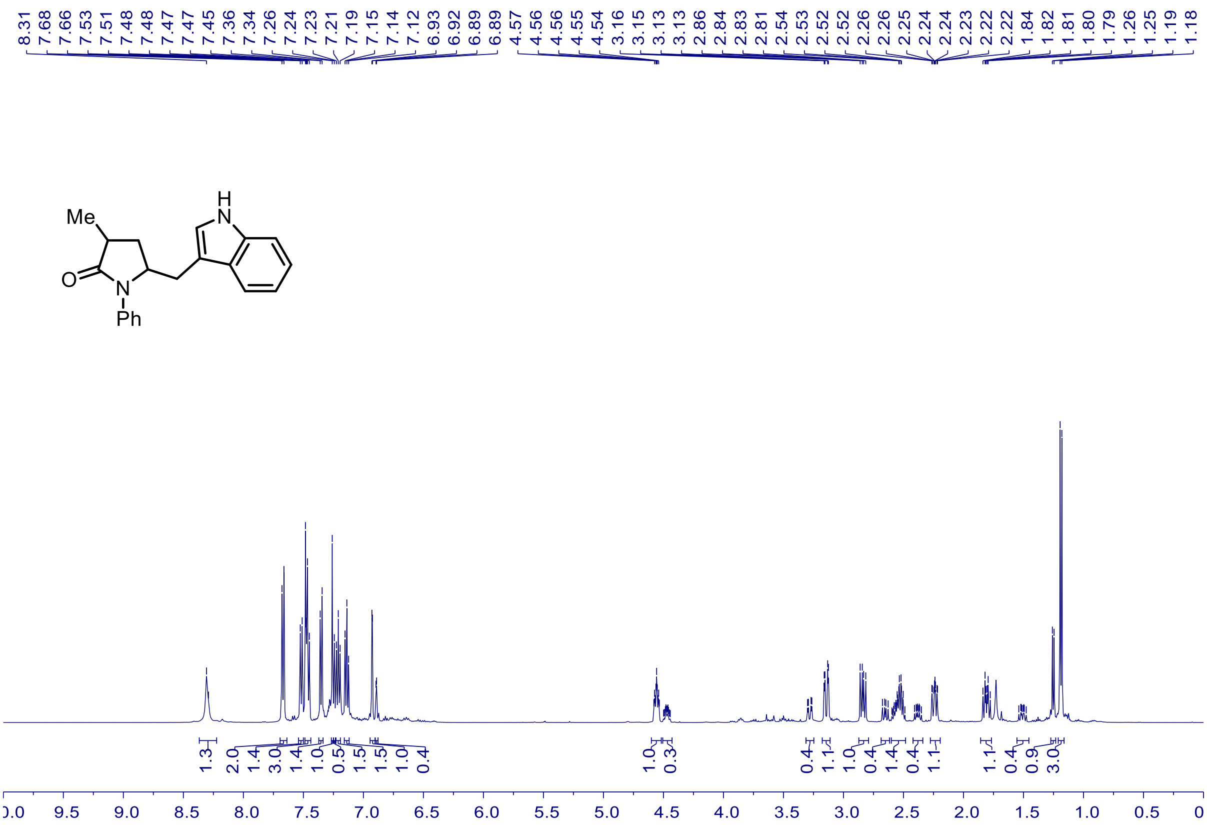

72 – ^{13}C NMR (126 MHz, $CDCl_3$)

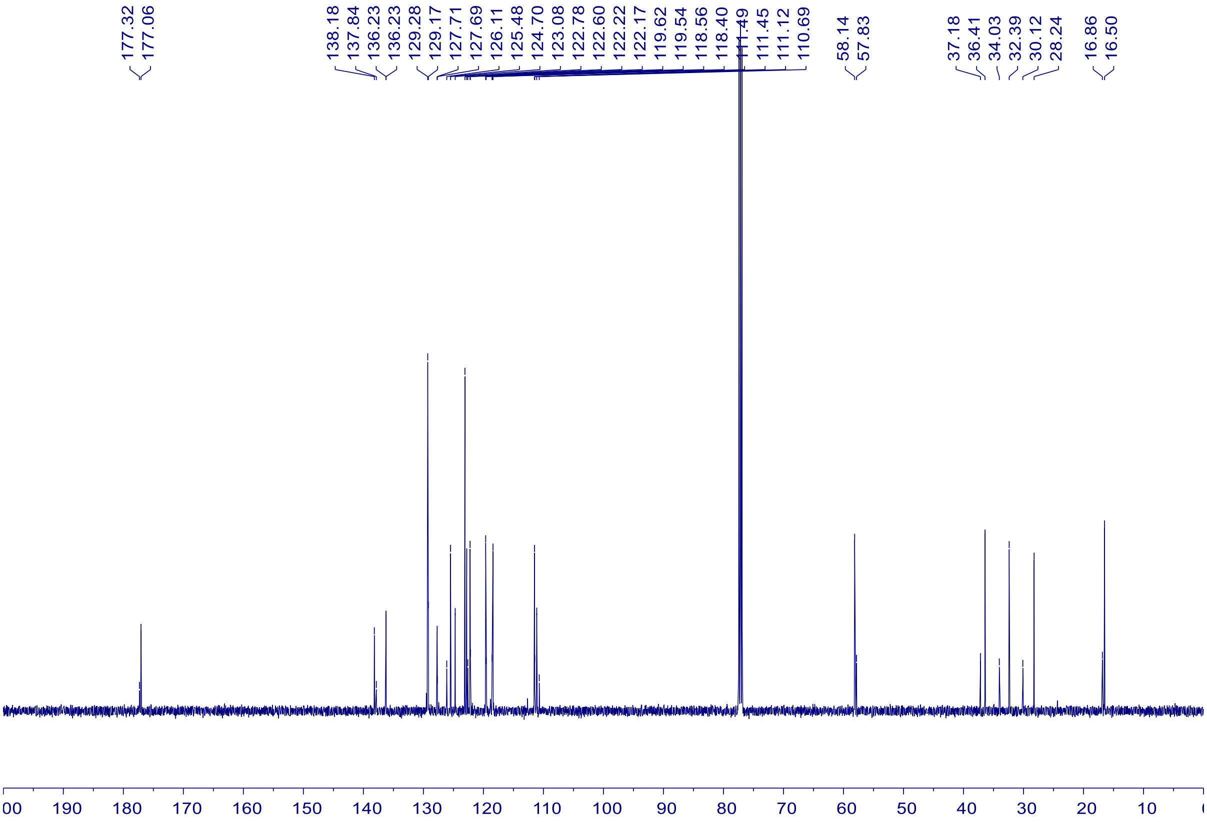

73 *cis* isomer – ^{1}H NMR (400 MHz, $CDCl_3$)

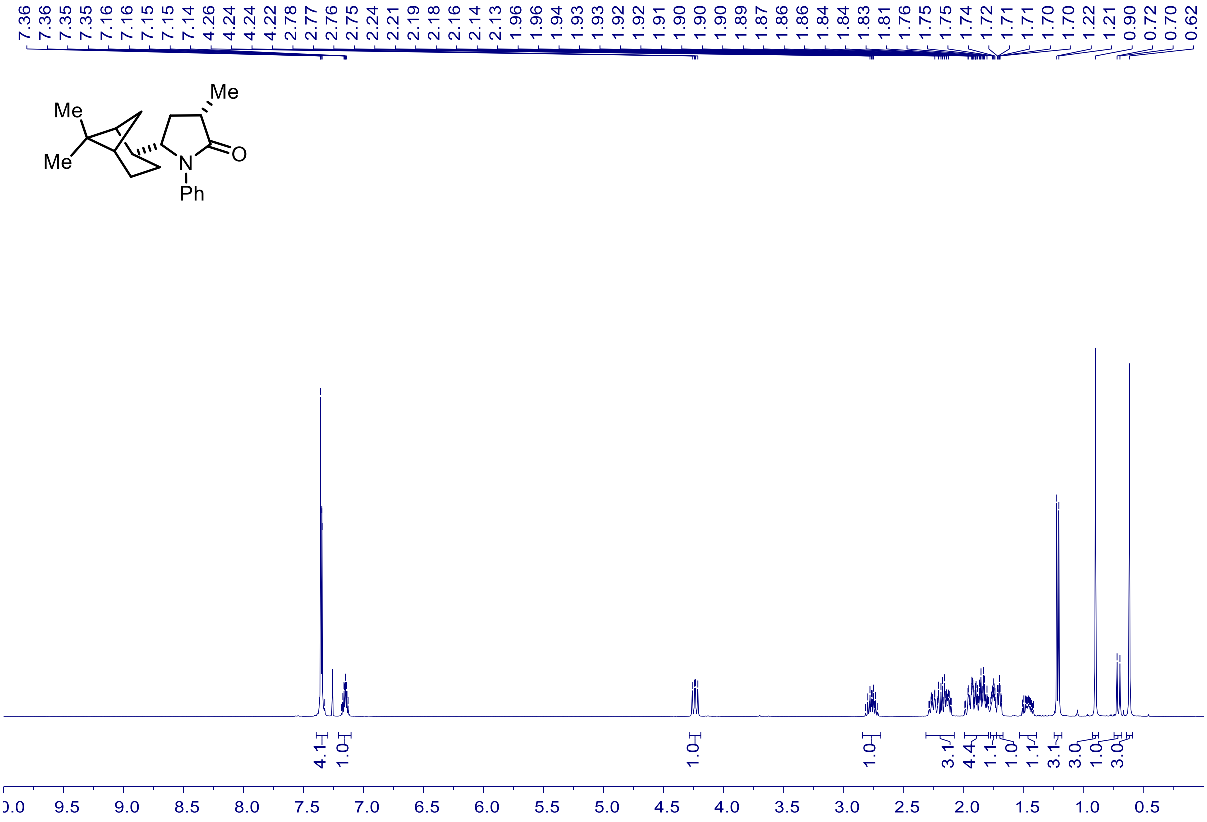

73 *cis* isomer – ^{13}C NMR (101 MHz, $CDCl_3$)

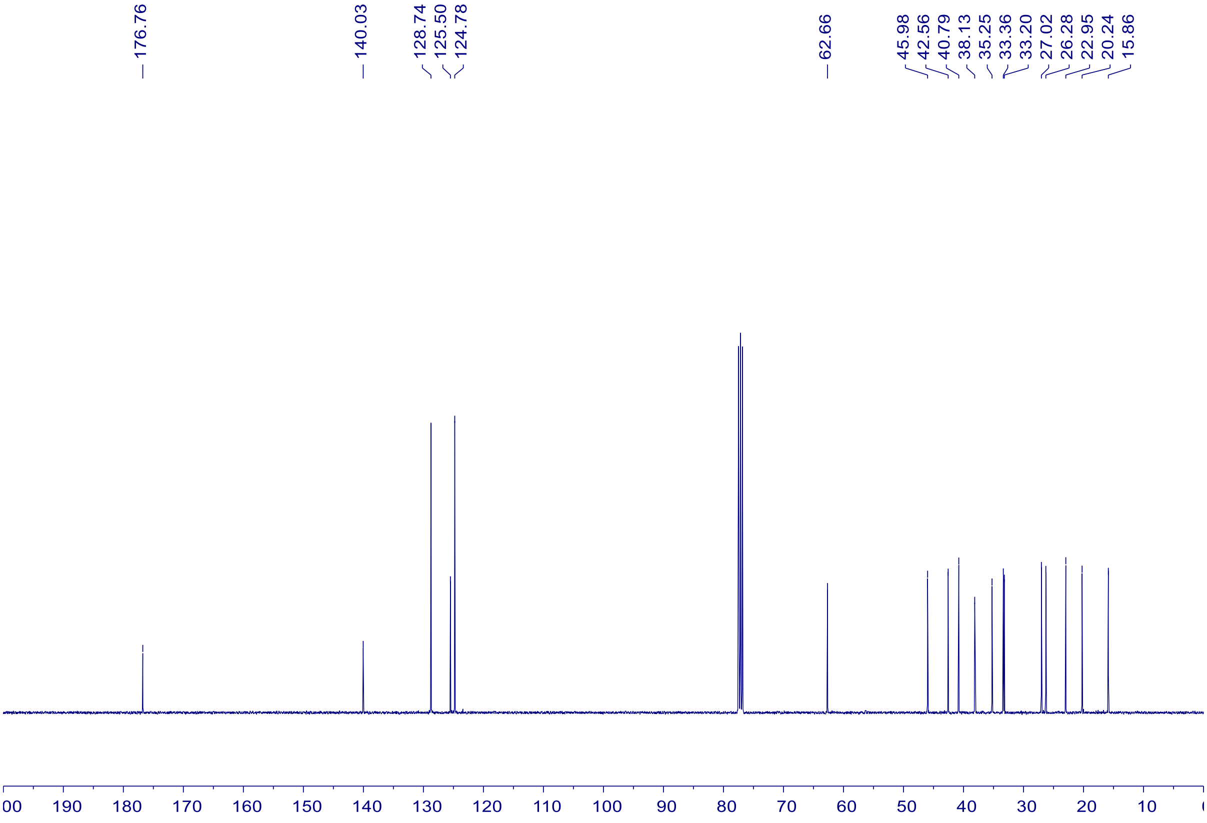

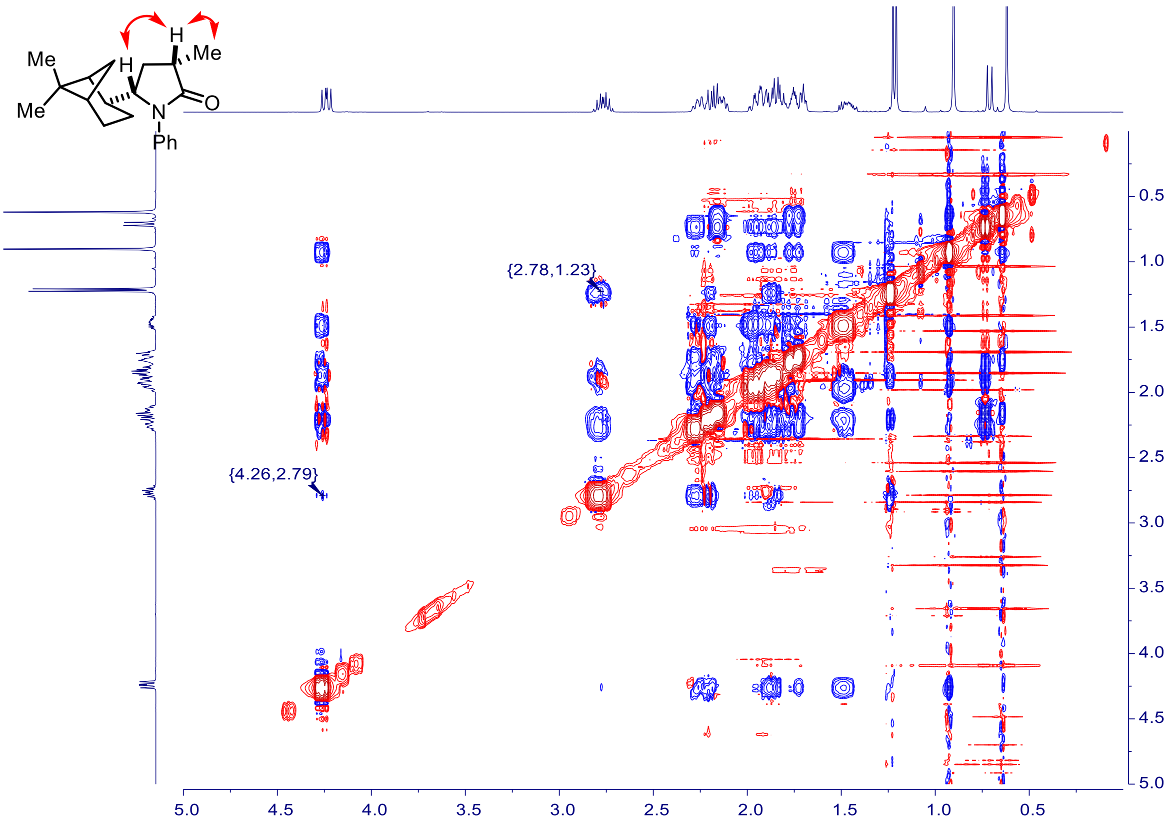

73 *trans* isomer – ^{1}H NMR (400 MHz, $CDCl_3$)

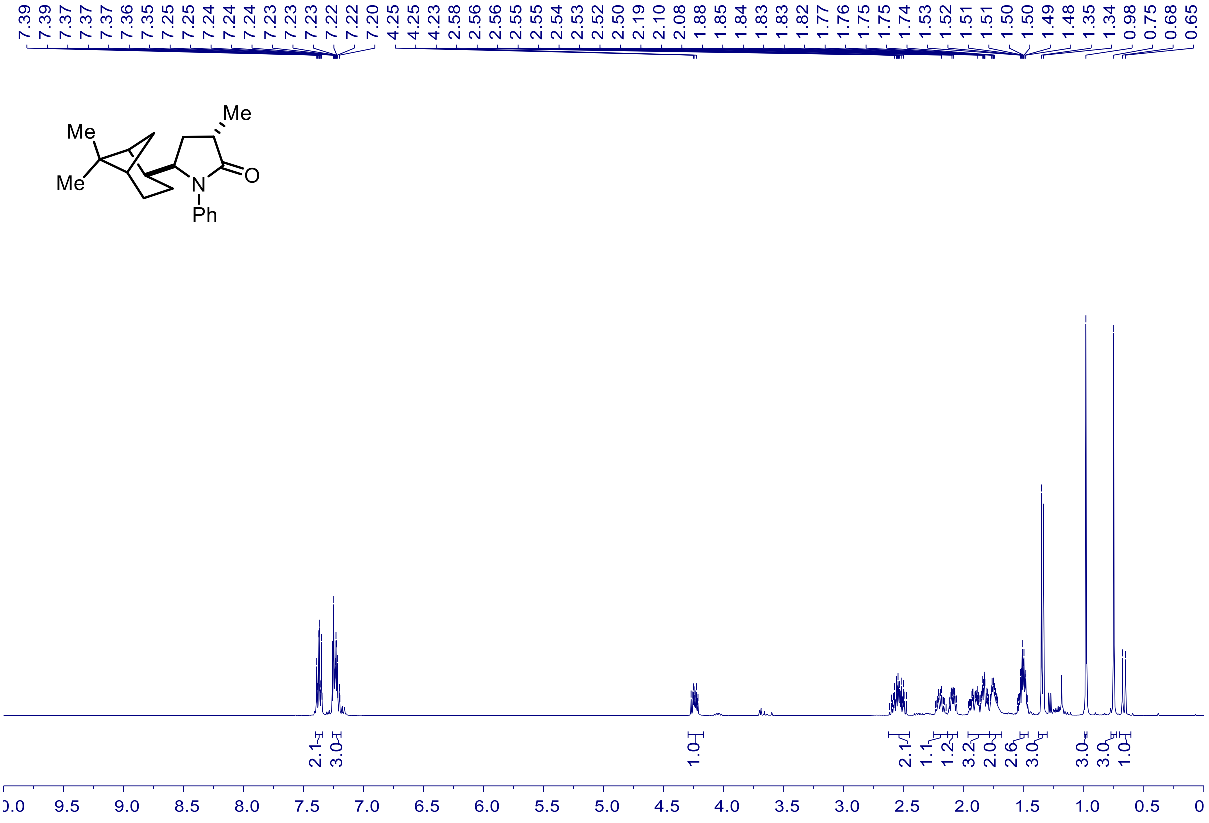

73 *trans* isomer – ^{13}C NMR (101 MHz, $CDCl_3$)

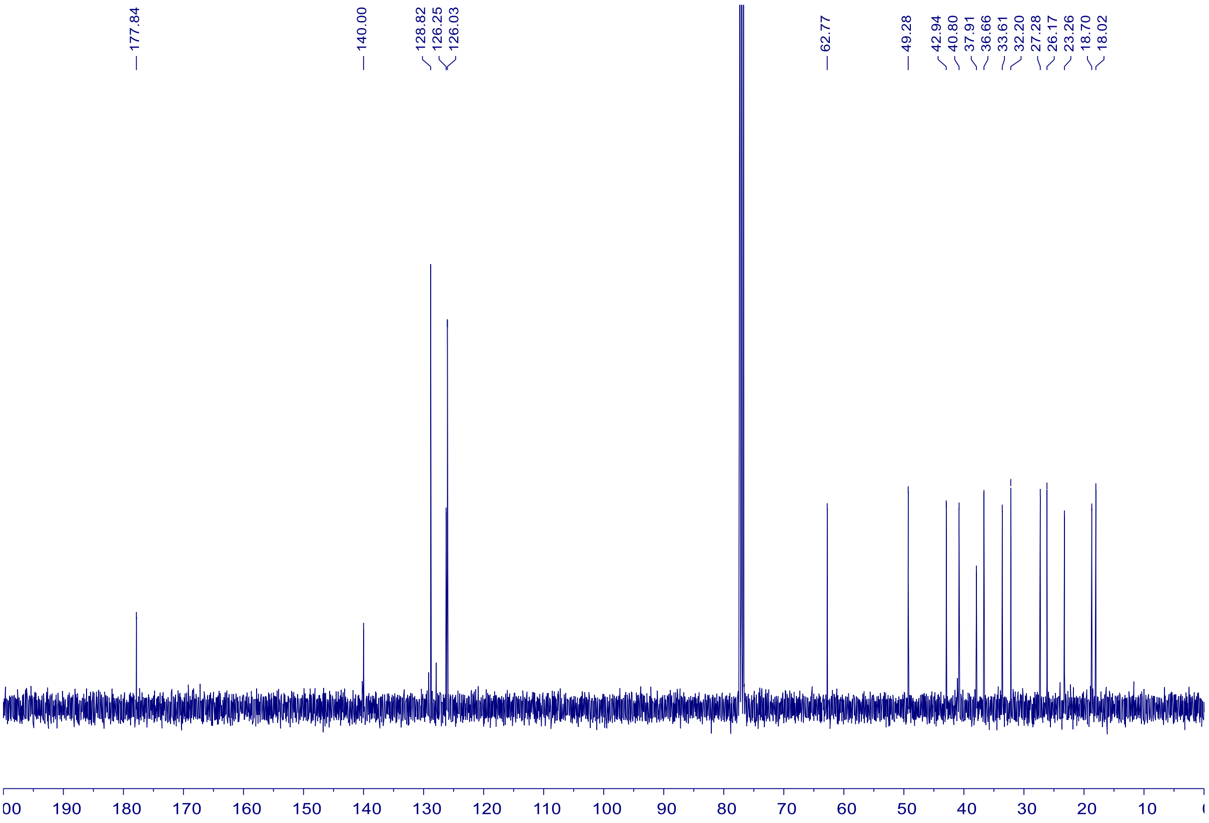

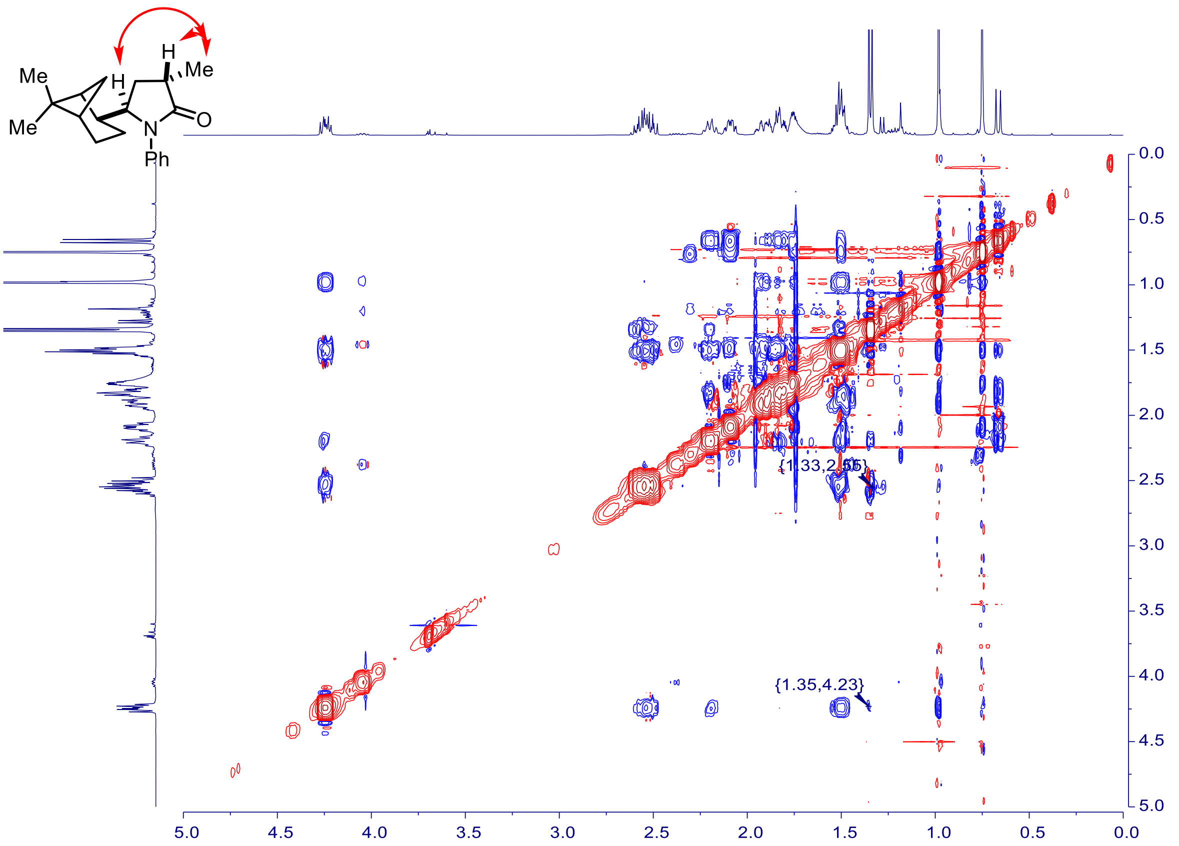

74 – ^{1}H NMR (400 MHz, $CDCl_3$)

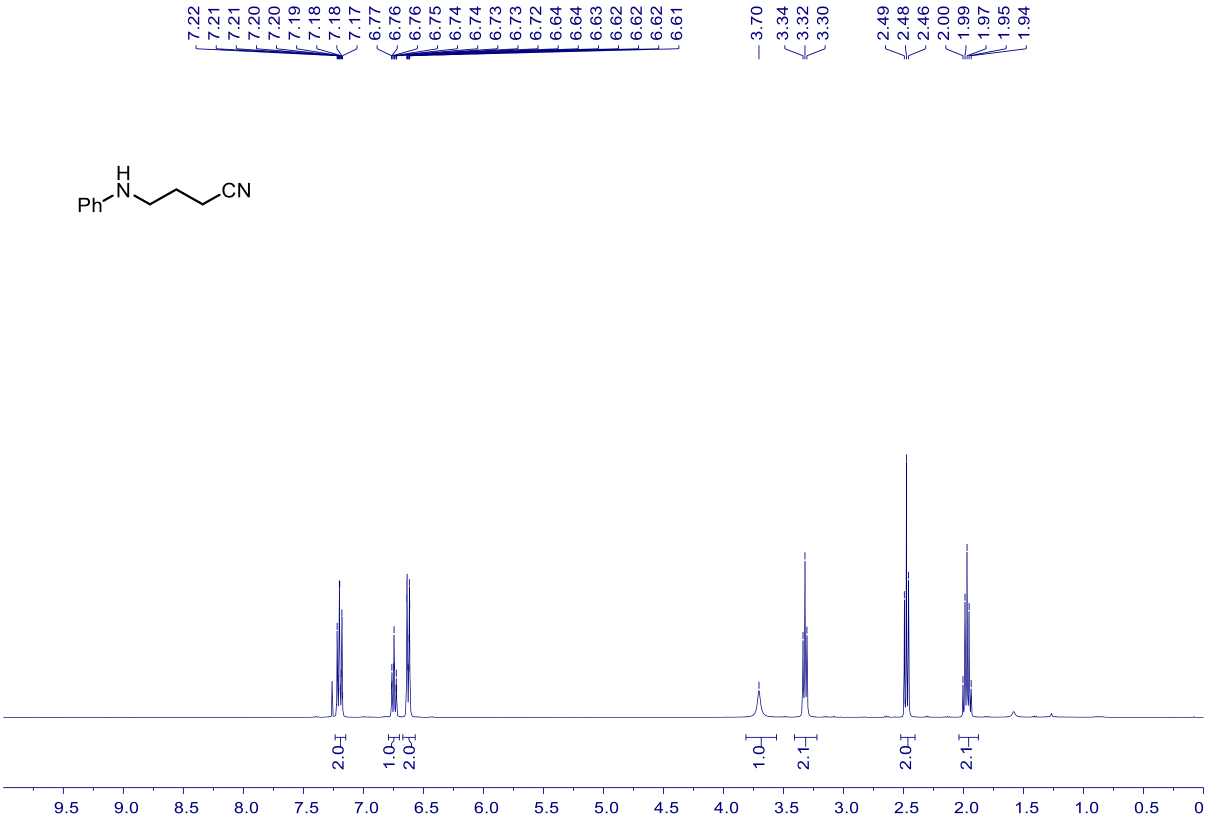

74 – ^{13}C NMR (101 MHz, $CDCl_3$)

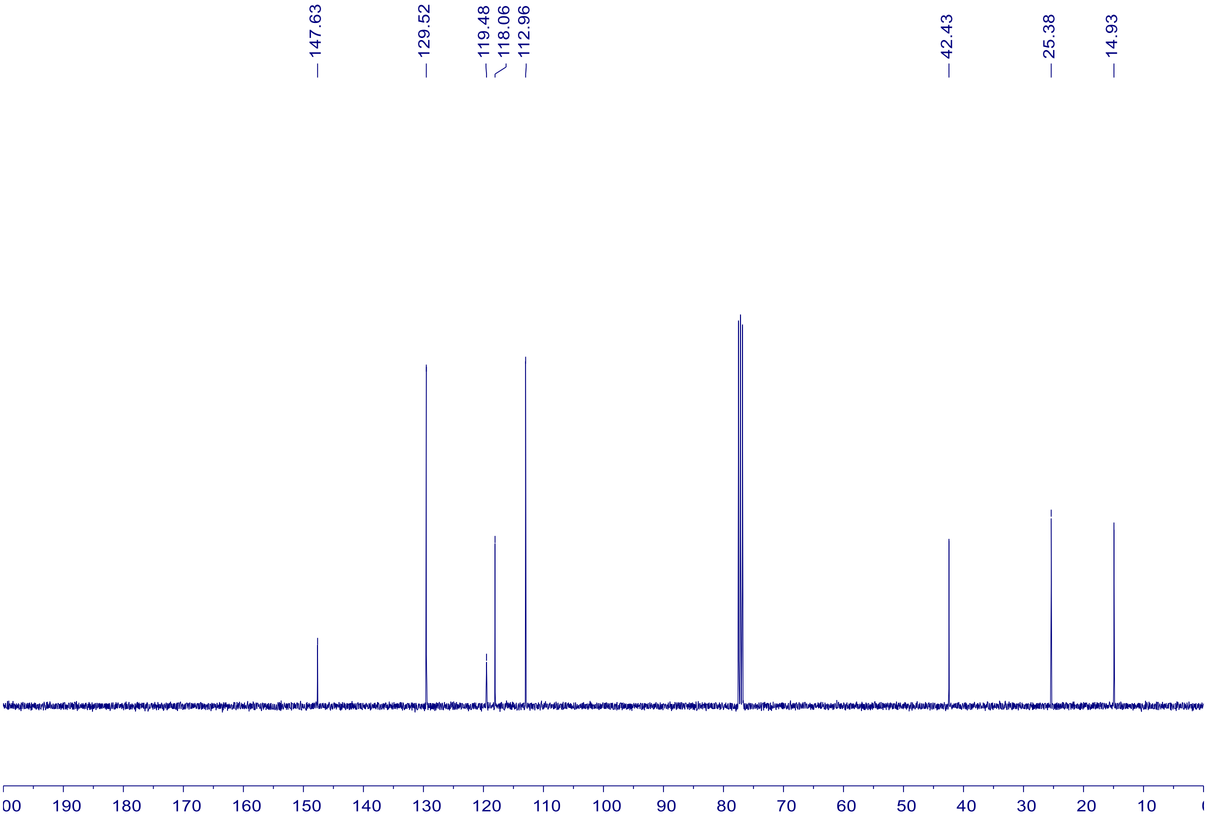

75 – ^{1}H NMR (500 MHz, $CDCl_3$)

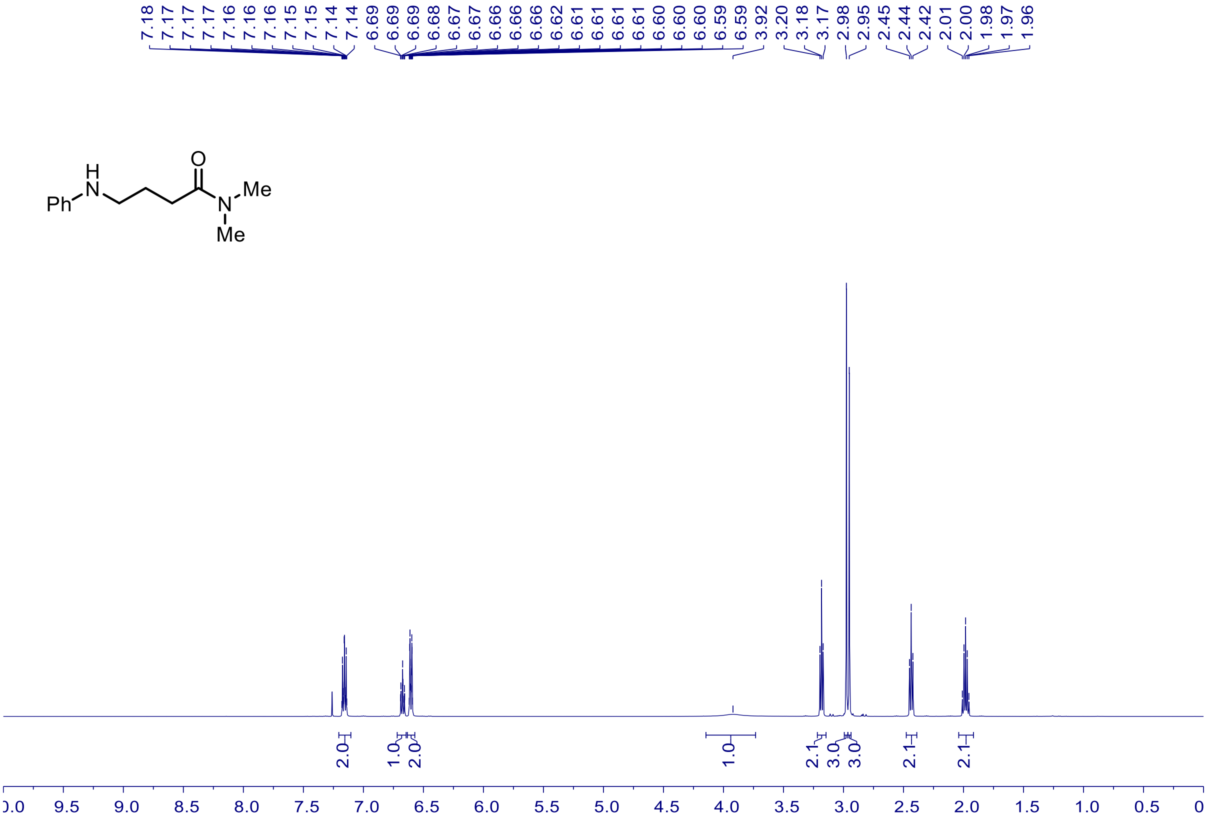

75 – ^{13}C NMR (126 MHz, $CDCl_3$)

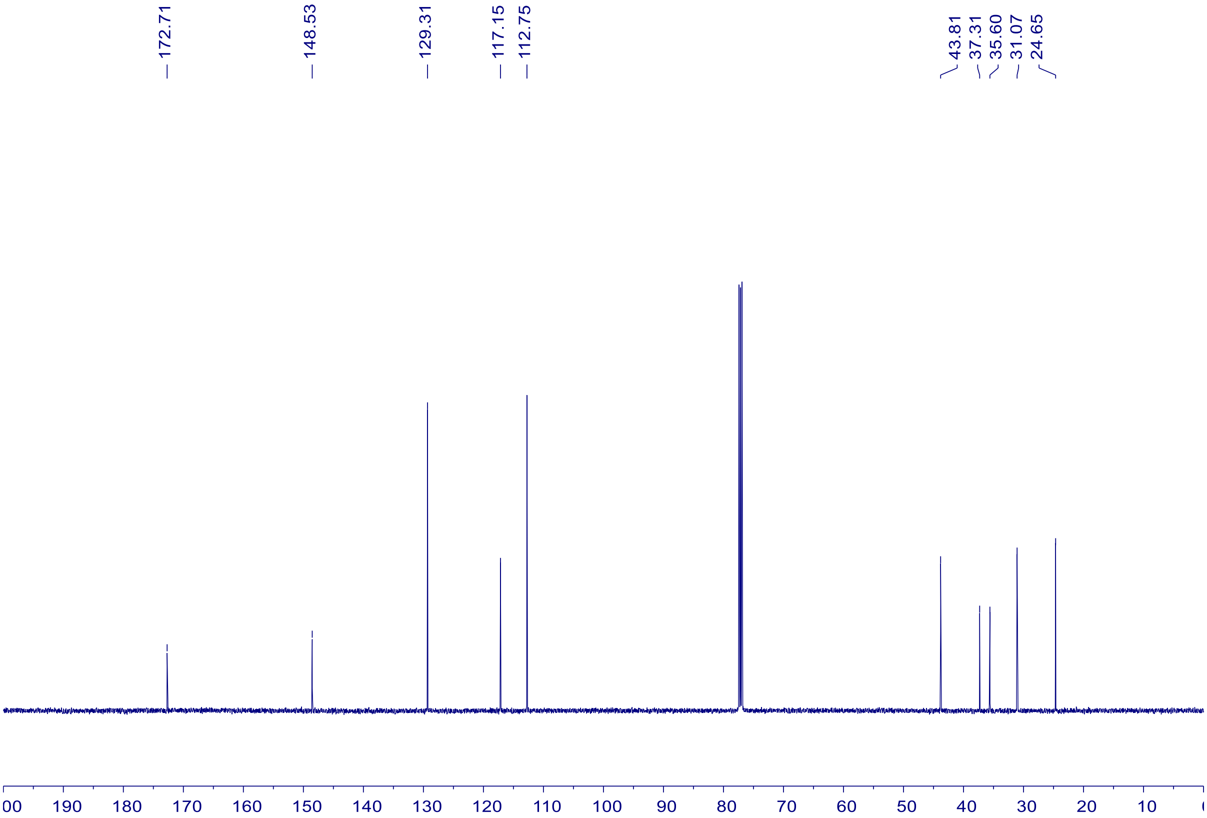

76 – ^{1}H NMR (500 MHz, $CDCl_3$)

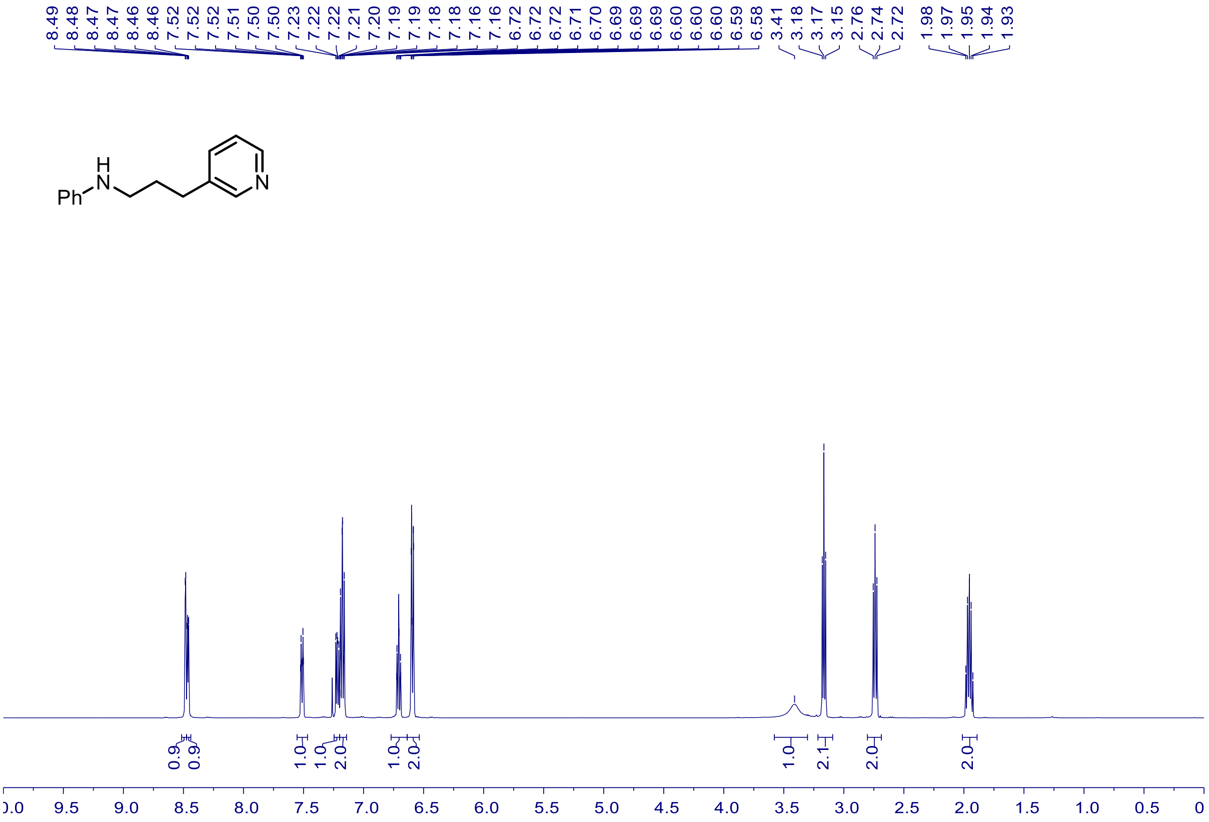

76 – ^{13}C NMR (126 MHz, $CDCl_3$)

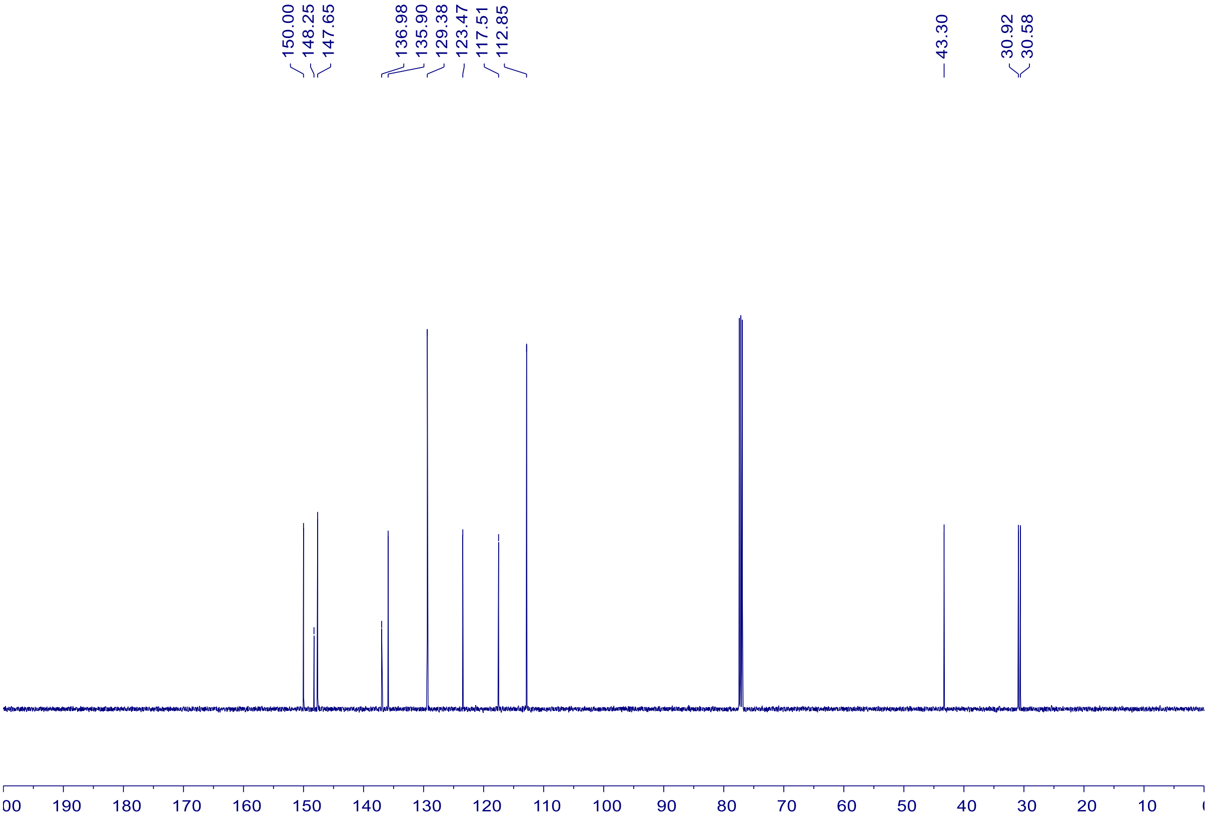

77 – ^{1}H NMR (500 MHz, $CDCl_3$)

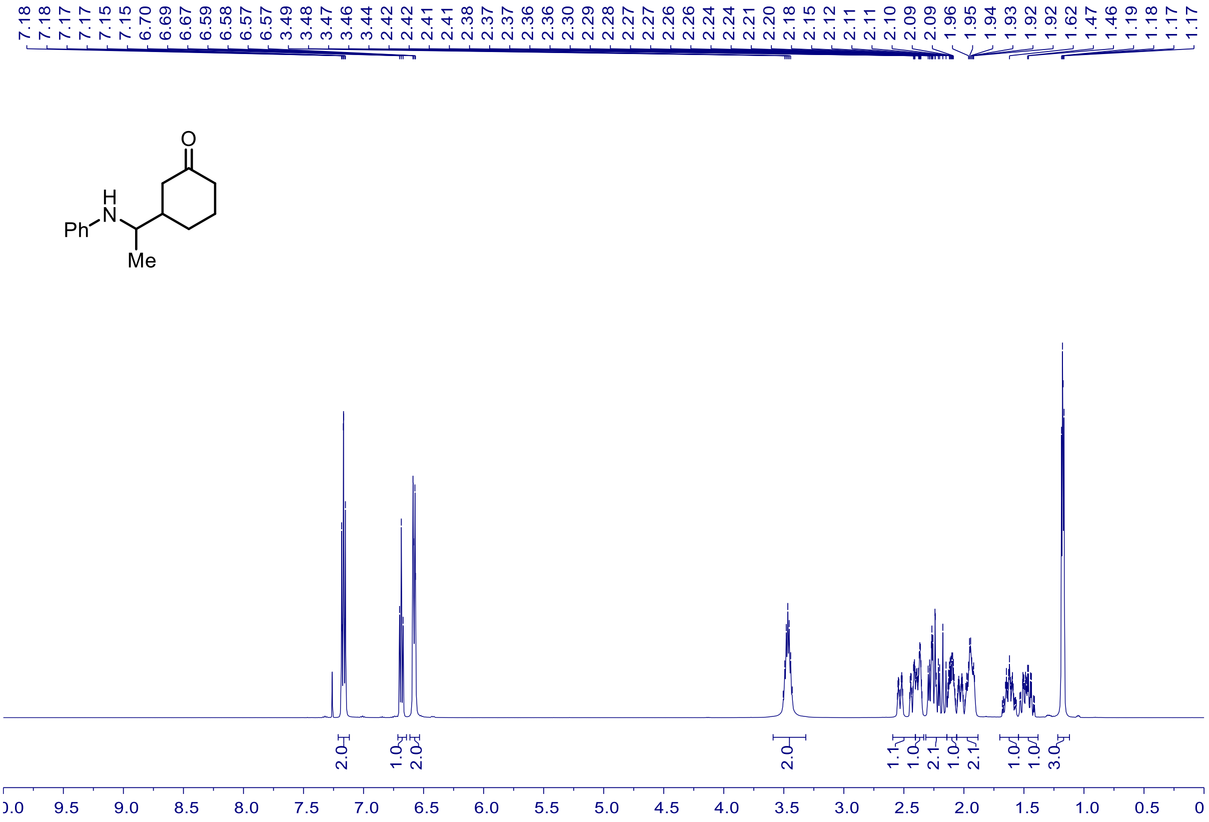

77 – ^{13}C NMR (126 MHz, $CDCl_3$)

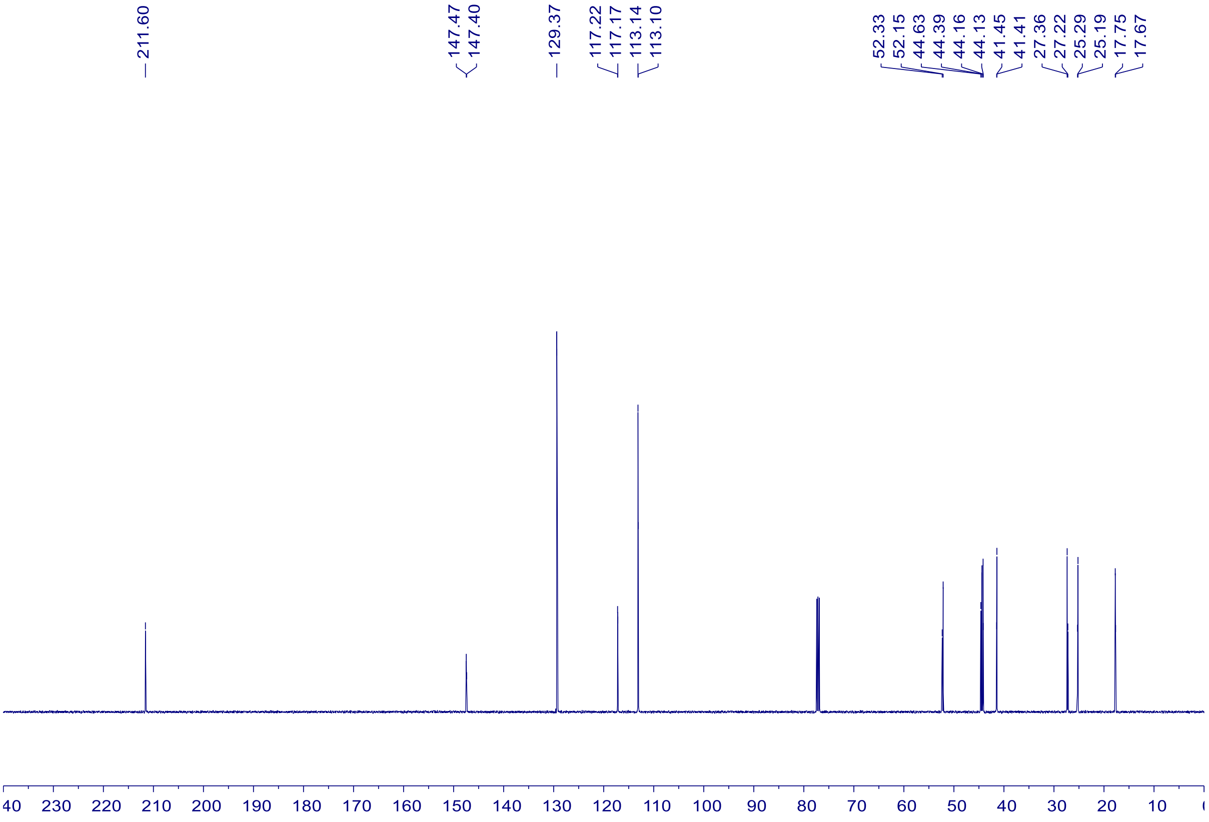

78 – ^{1}H NMR (400 MHz, $CDCl_3$)

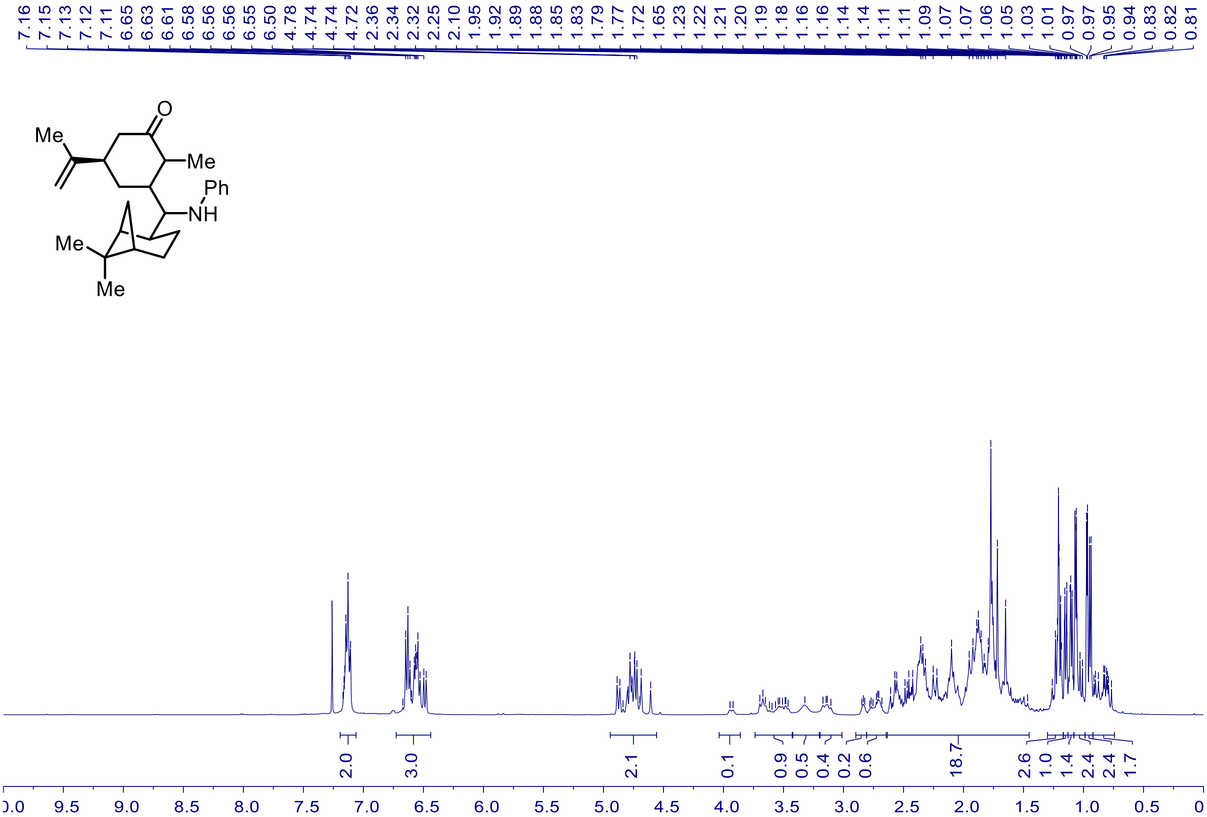

78 – ^{13}C NMR (101 MHz, $CDCl_3$)

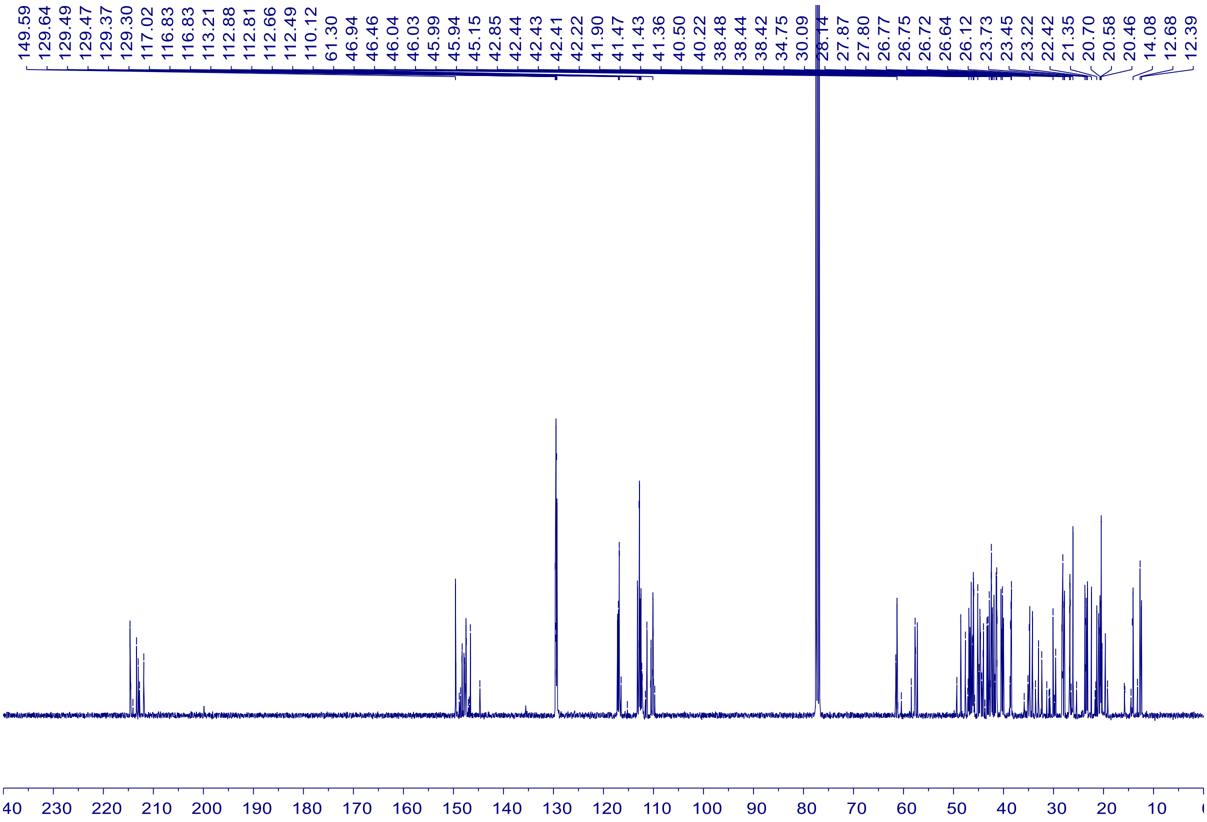

S10 – ^{1}H NMR (500 MHz, $CDCl_3$)

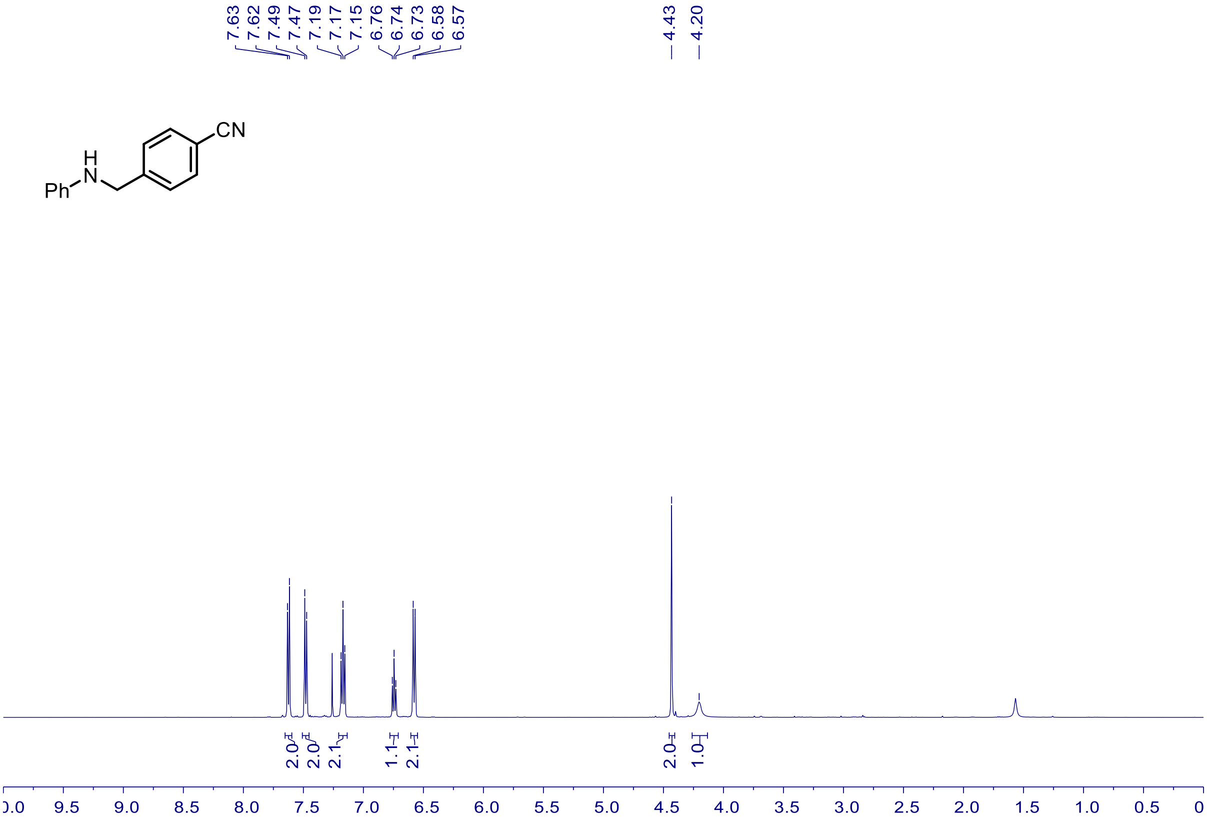

S10 – ^{13}C NMR (126 MHz, $CDCl_3$)

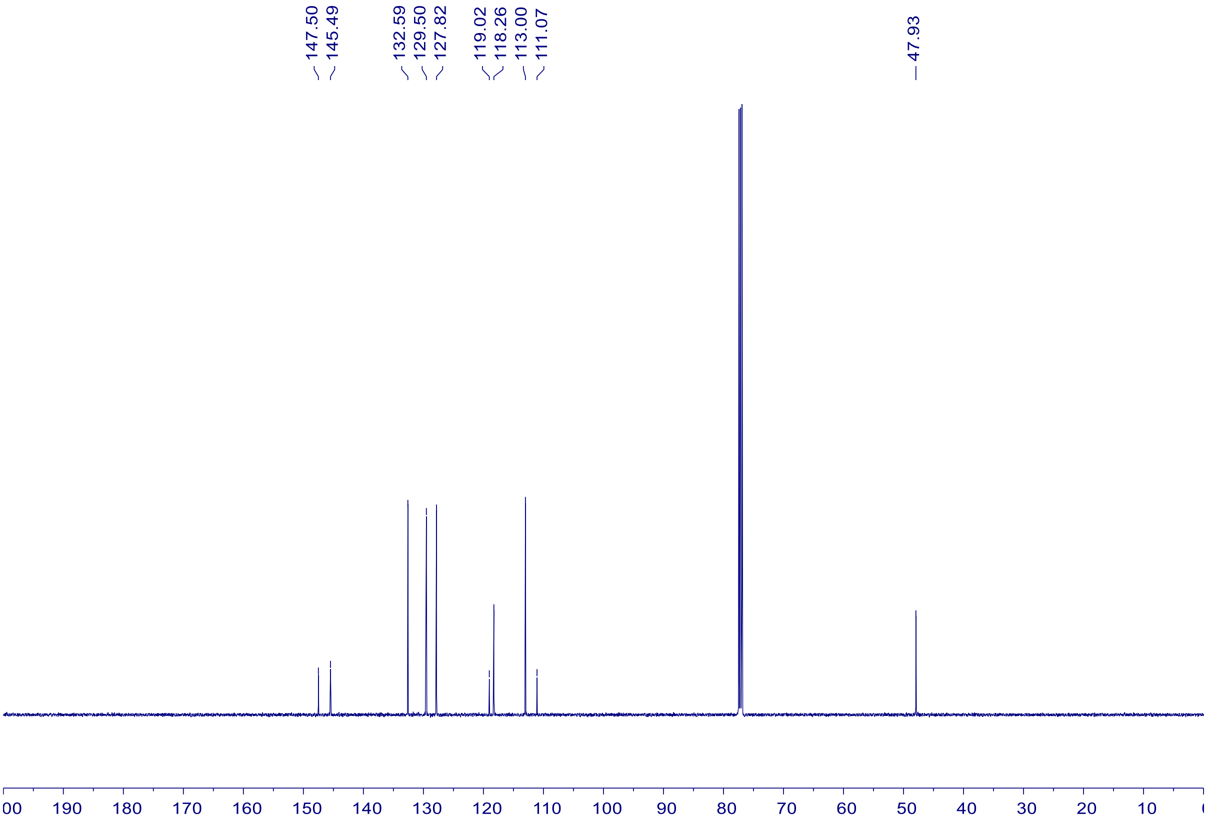

79 – ^{1}H NMR (400 MHz, $CDCl_3$)

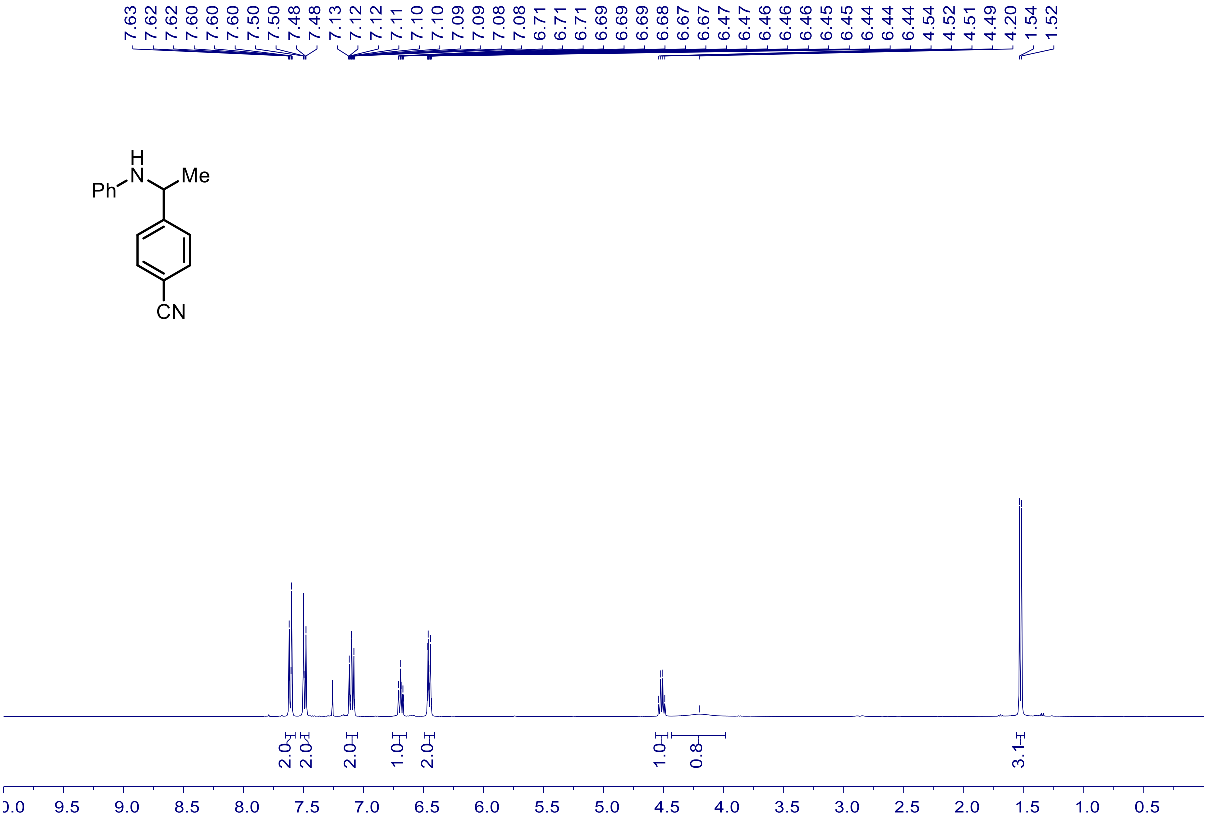

79 – ^{13}C NMR (101 MHz, $CDCl_3$)

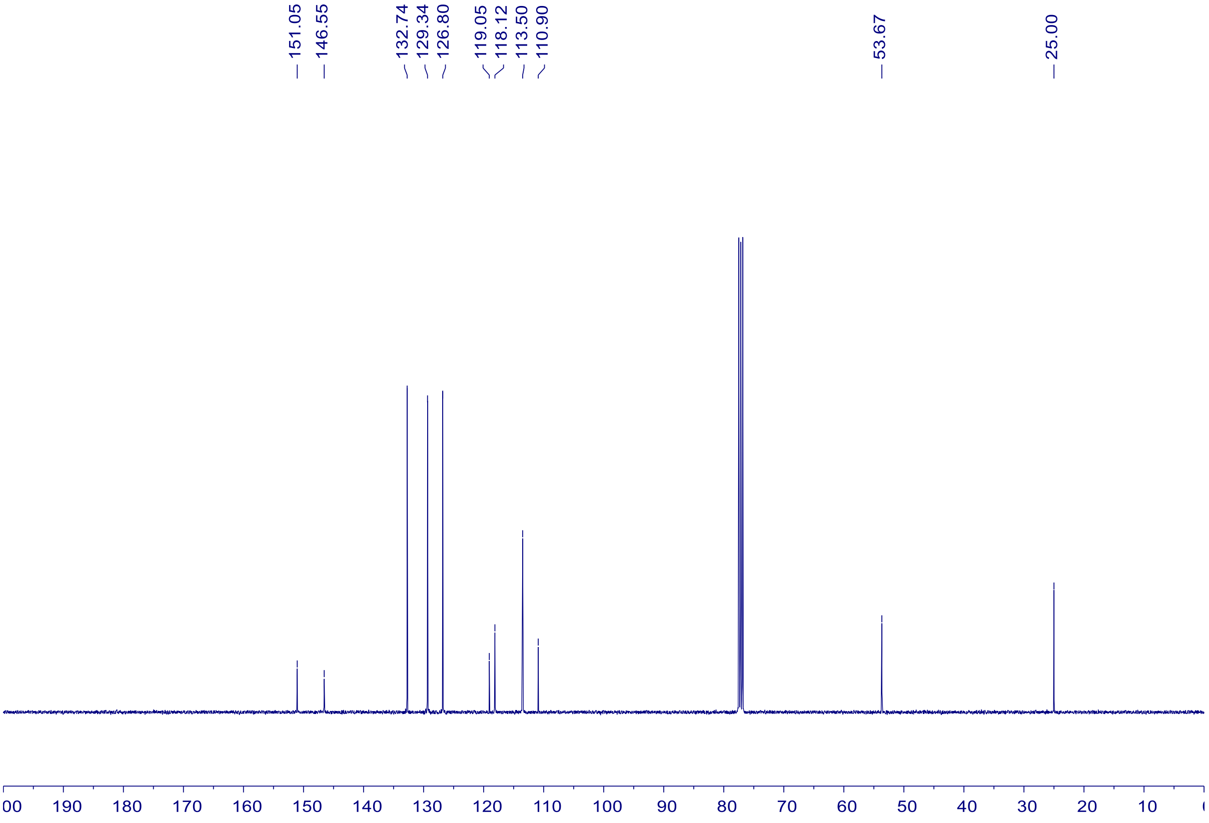

80 – ^{1}H NMR (500 MHz, $CDCl_3$)

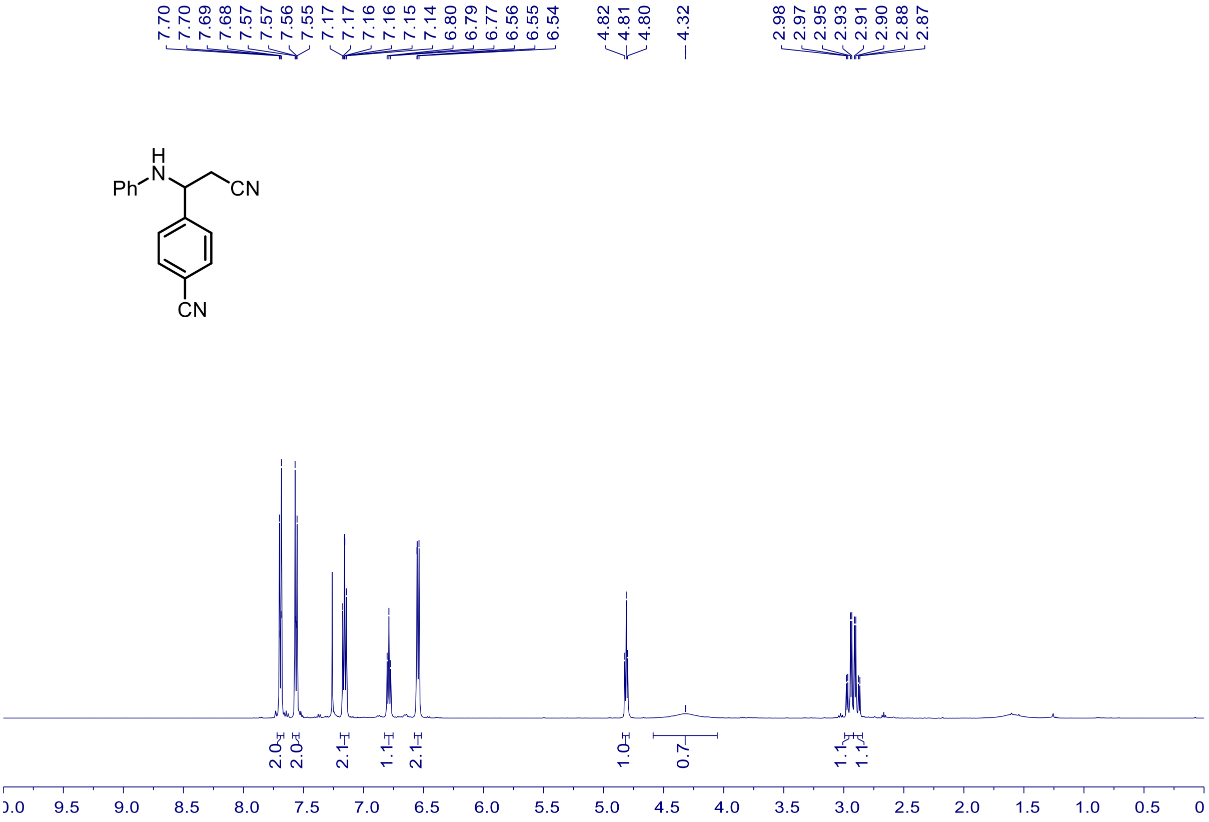

80 – ^{13}C NMR (101 MHz, $CDCl_3$)

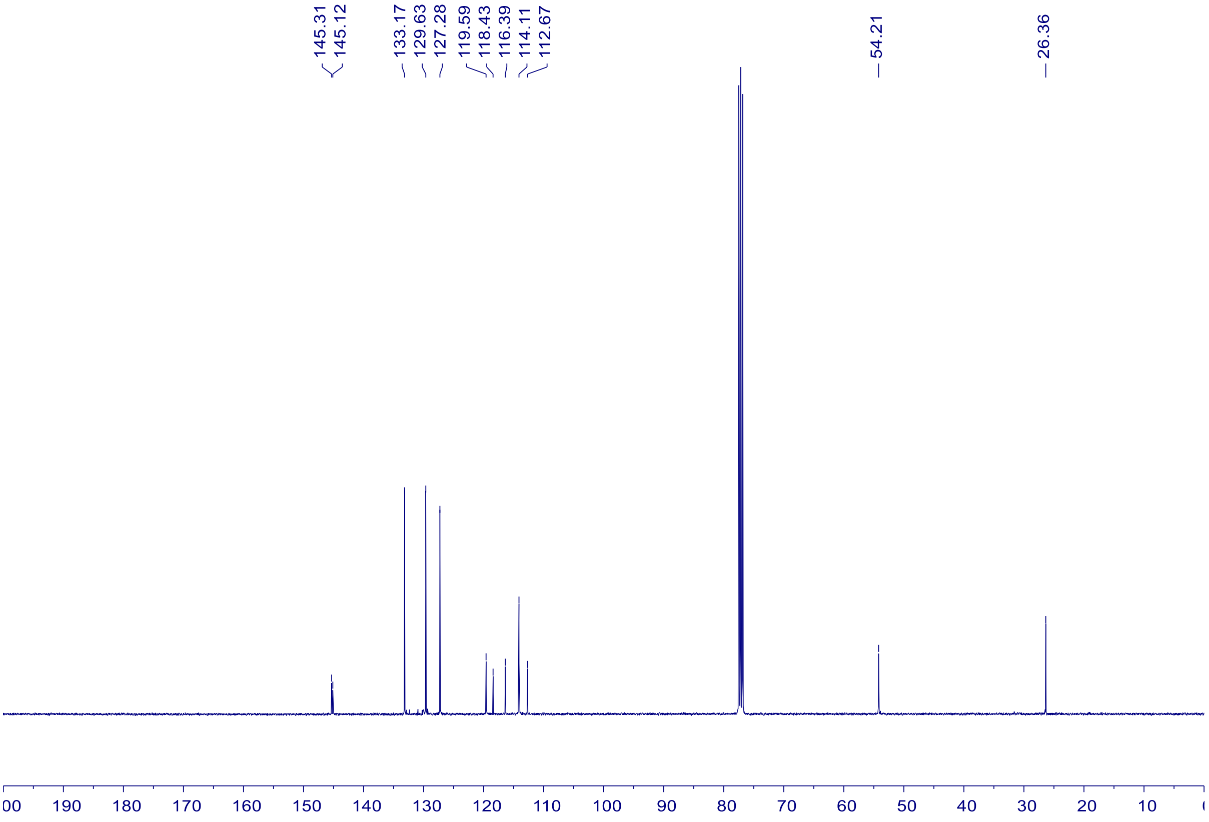

81 – ^{1}H NMR (400 MHz, $CDCl_3$)

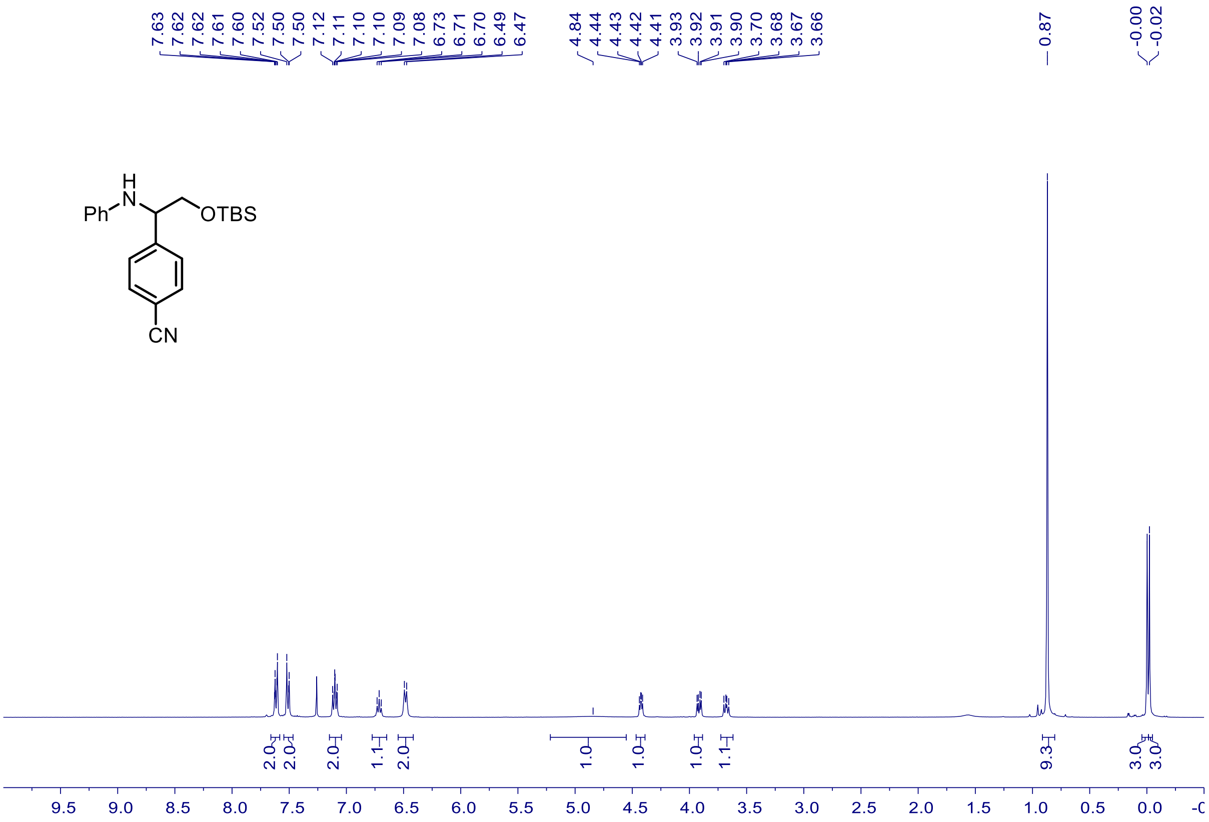

81 – ^{13}C NMR (101 MHz, $CDCl_3$)

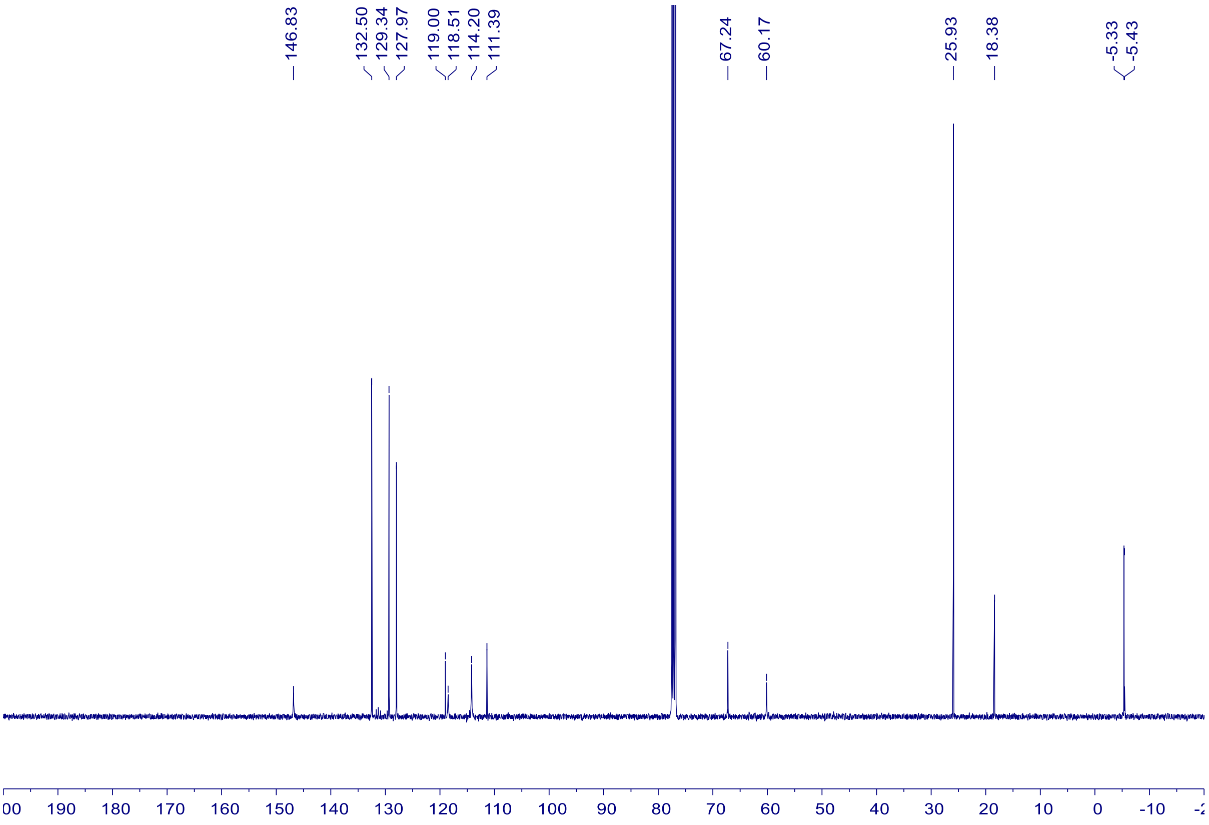

82 – ^{1}H NMR (500 MHz, $CDCl_3$)

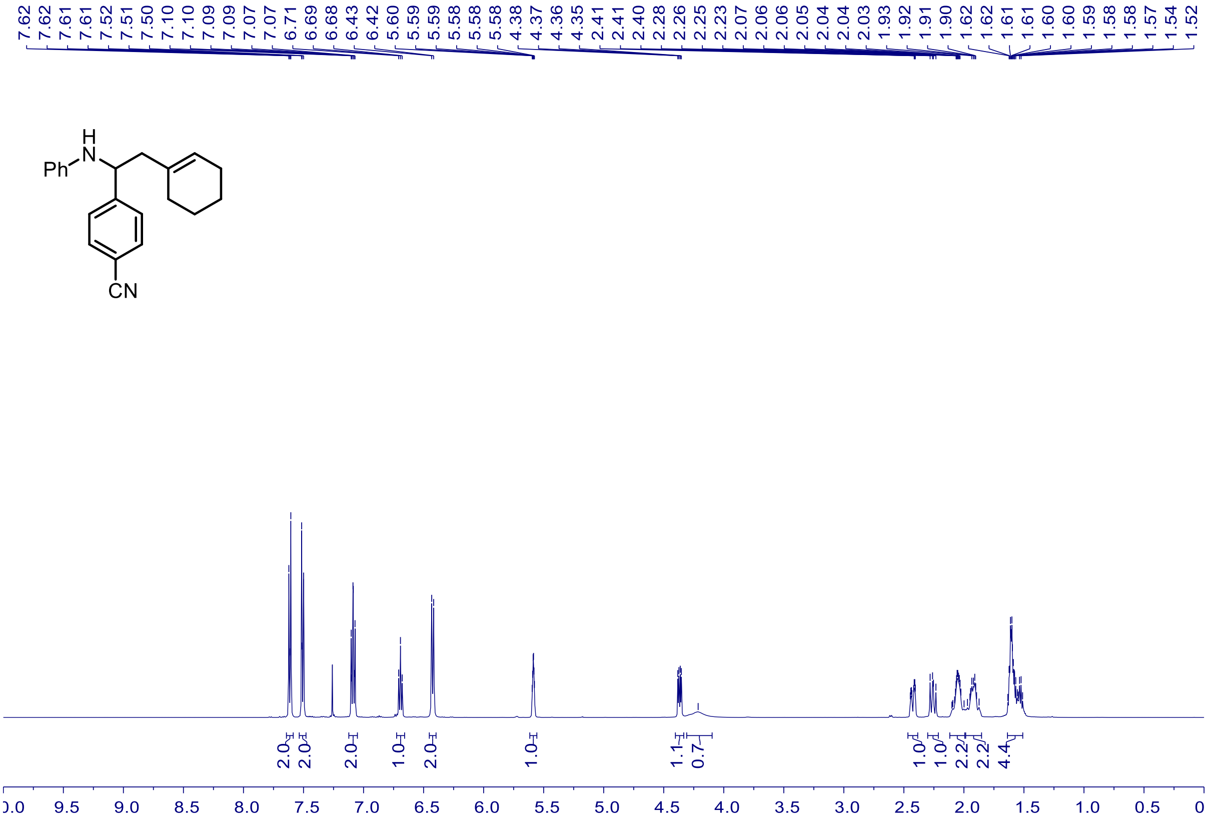

82 – ^{13}C NMR (126 MHz, $CDCl_3$)

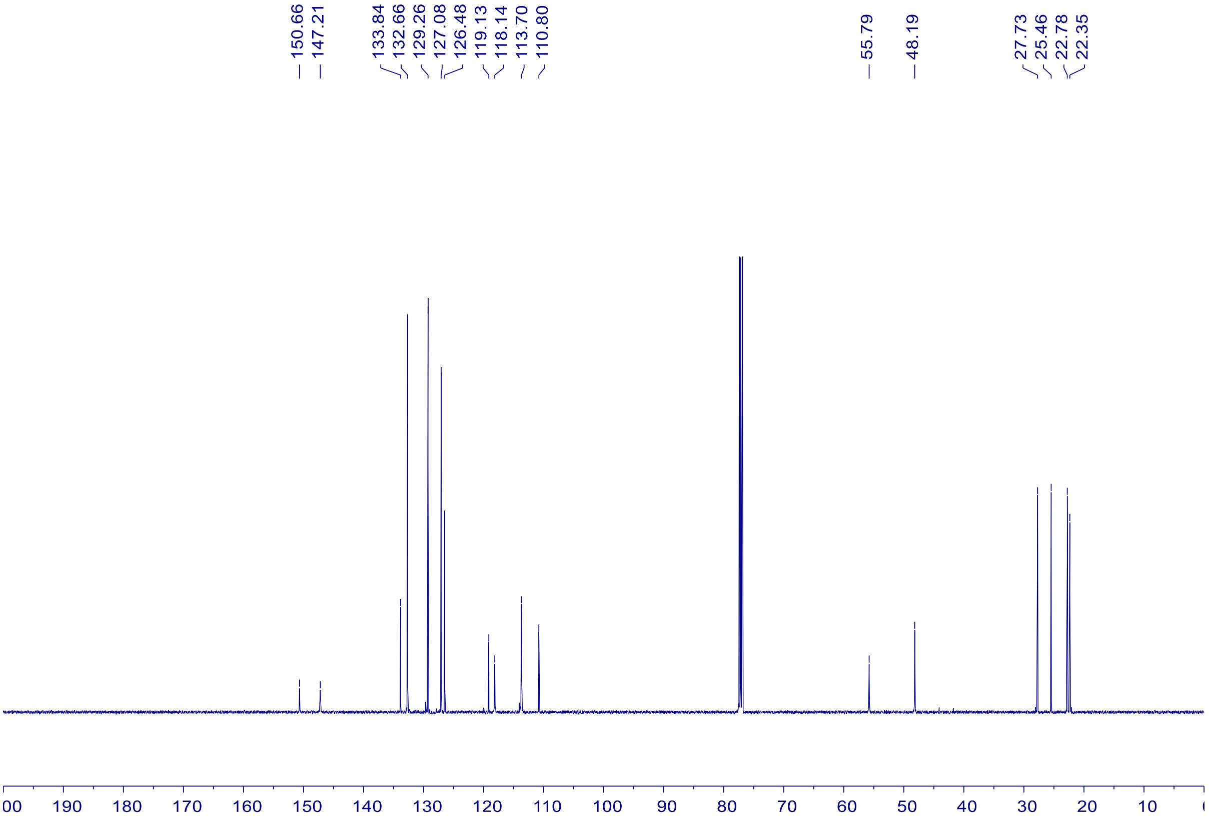

83 – ^{1}H NMR (400 MHz, $CDCl_3$)

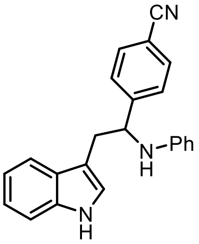

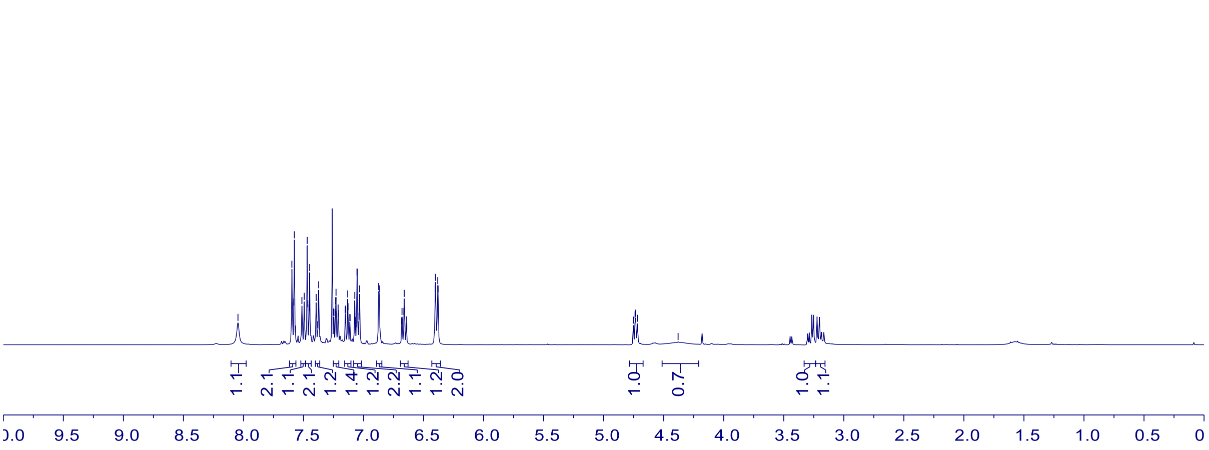

83 – ^{13}C NMR (101 MHz, $CDCl_3$)

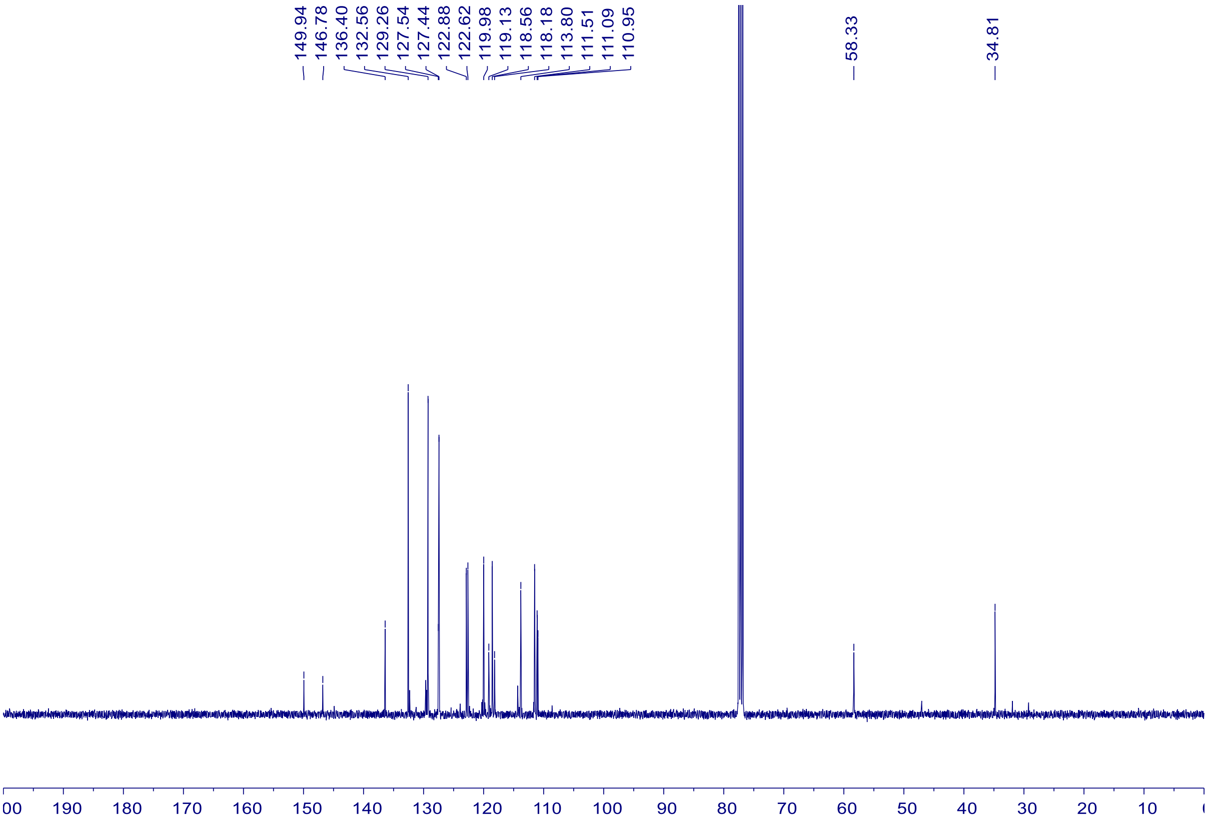

84 – ^{1}H NMR (400 MHz, CDCl$_3$)

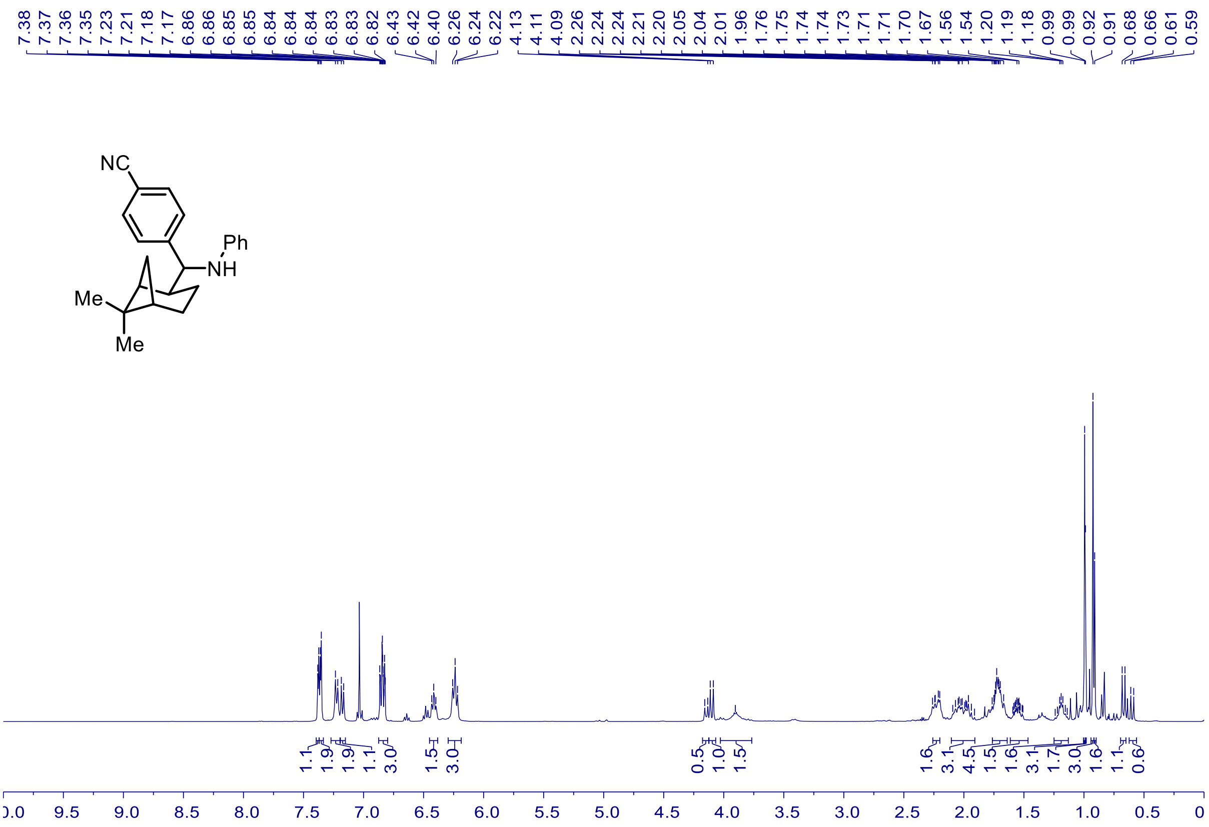

84 – ^{13}C NMR (126 MHz, CDCl$_3$)

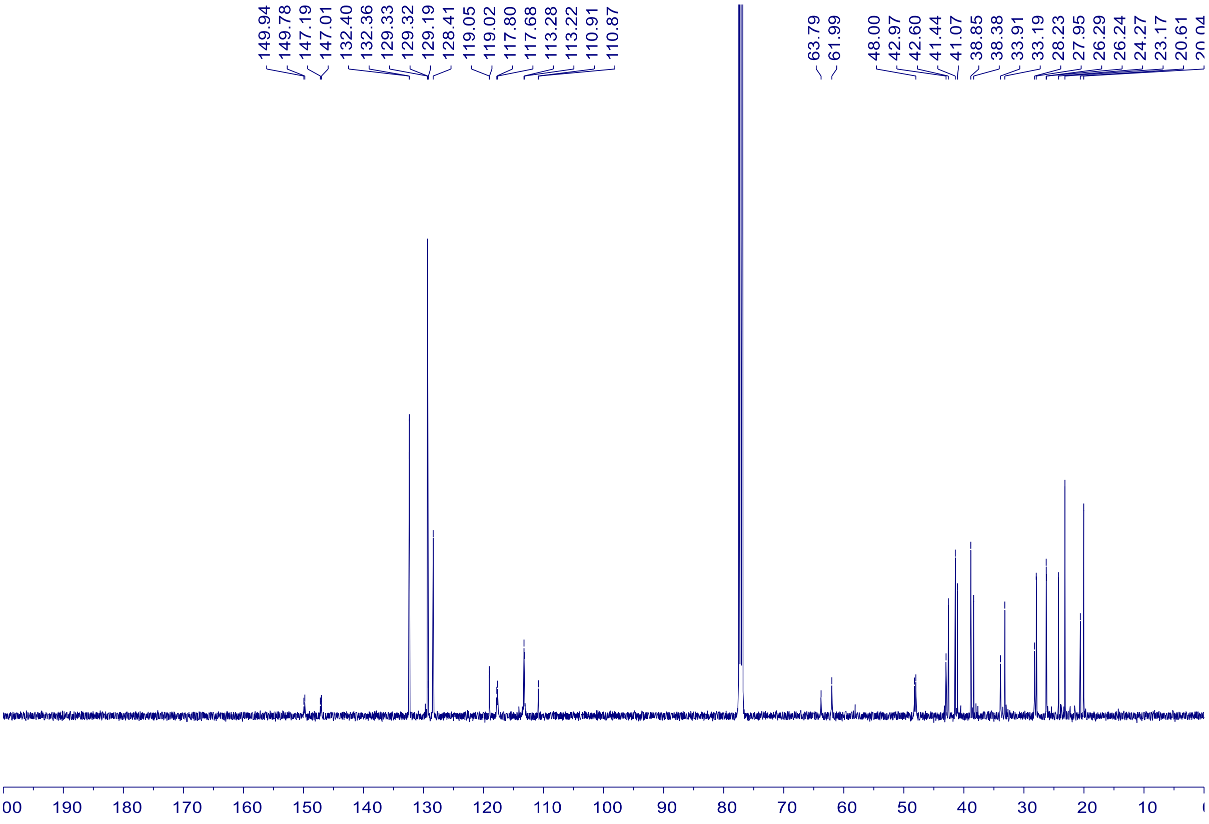

85 – ^{1}H NMR (400 MHz, $CDCl_3$)

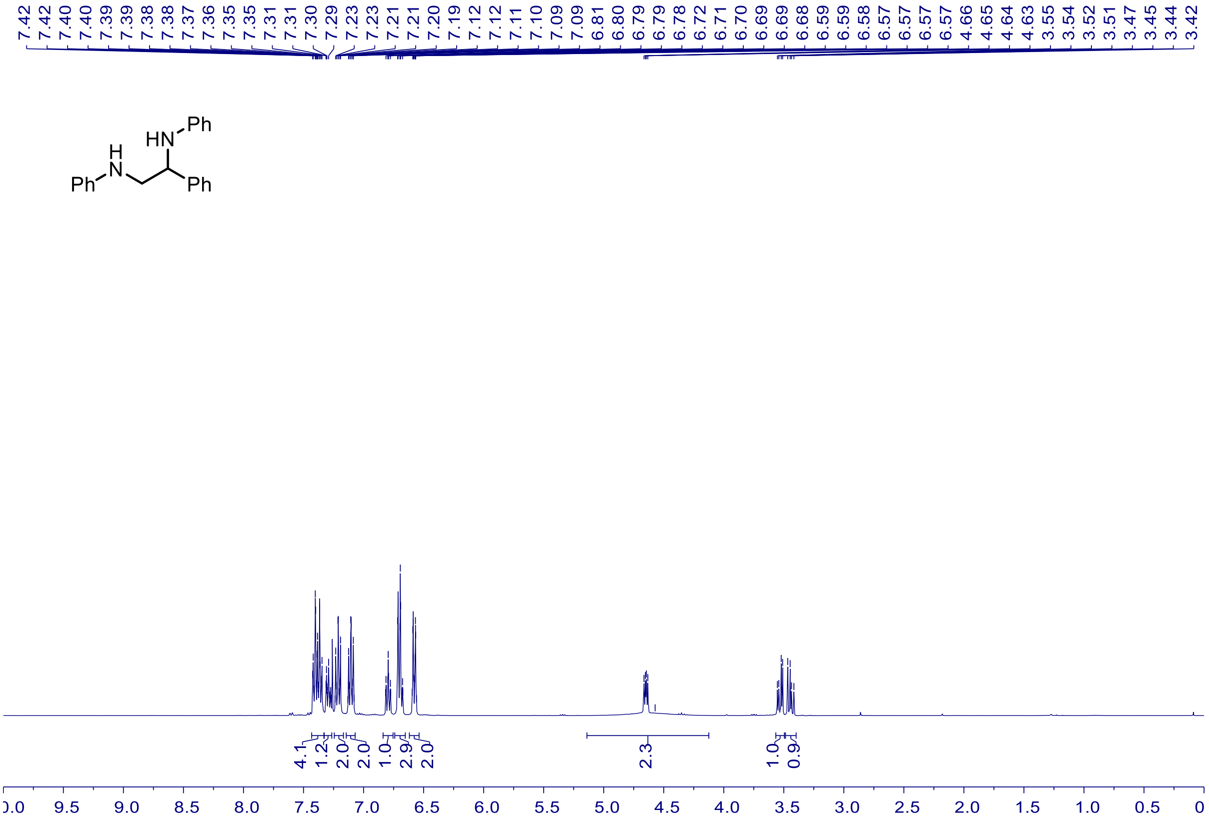

85 – ^{13}C NMR (101 MHz, $CDCl_3$)

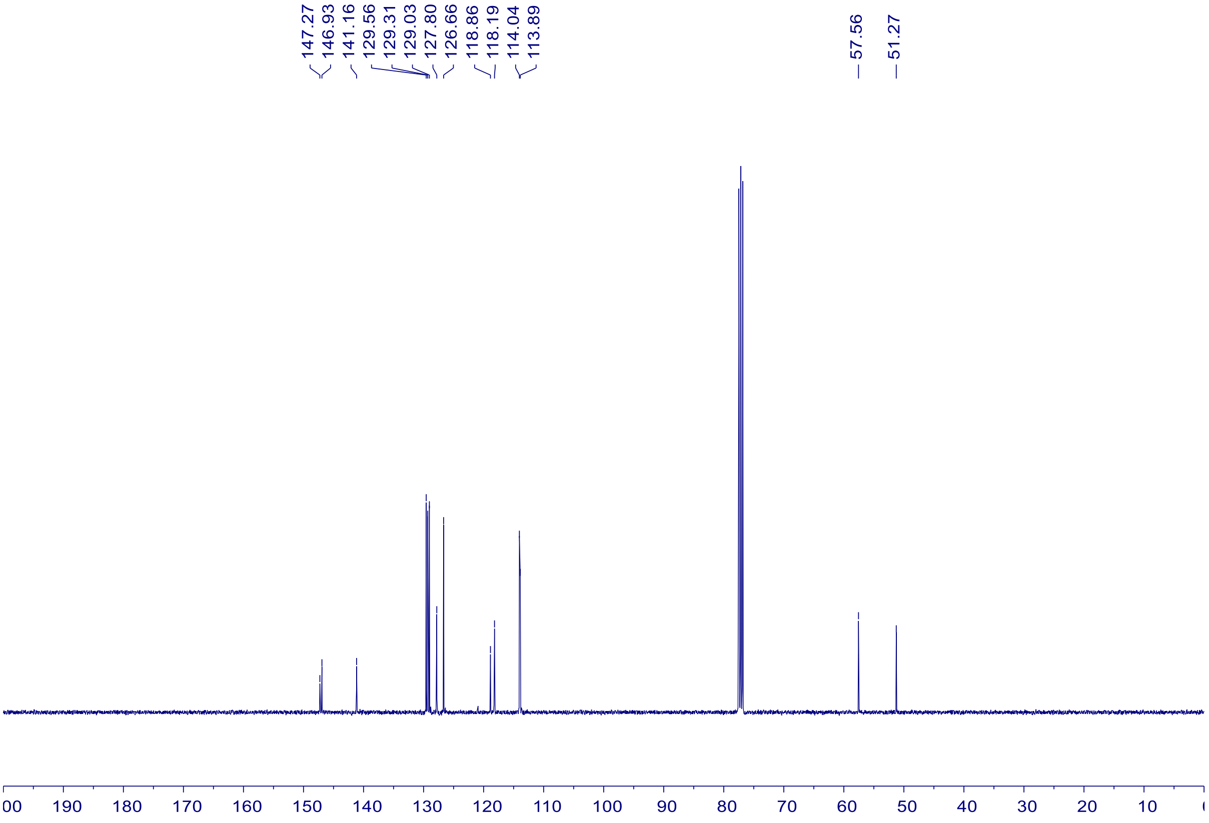

8 References

1. Zhang, G.; Yin, Z.; Zheng, S., *Org. Lett.*, **2016**, *18*, 300–303.
2. Kattamuri, P. V.; Yin, J.; Siriwongsup, S.; Kwon, D.-H.; Ess, D. H.; Li, Q.; Li, G.; Yousufuddin, M.; Richardson, P. F.; Sutton, S. C.; Kürti, L., *J. Am. Chem. Soc.* **2017**, *139*, 11184−11196.
3. Zang, Z.-L.; Karnakanti, S.; Zhao, S.; Hu, P.; Wang, Z.; Shao, P.-L.; He, Y., *Org. Lett.* **2017**, *19*, 1354–1357.
4. Crawford, S. M.; Lavery, C. B.; Stradiotto, M., *Chem. - A Eur. J.* **2013**, *19*, 16760–16771.
5. Ruffoni, A.; Juliá, F.; Svejstrup, T. D.; McMillan, A. J.; Douglas, J. J.; Leonori, D., *Nat. Chem.* **2019**, *11*, 426−433.
6. Purohit, G.; Rawat, D. S.; Reiser, O., *ChemCatChem* **2020**, *12*, 569–575.
7. Tegeder, P.; Freitag, M.; Chepiga, K. M.; Muratsugu, S.; Moeller, N.; Lamping, S.; Tada, M.; Glorius, F.; Ravoo, B. J., *Chem. - A Eur. J.* **2018**, *24*, 18682–18688.
8. Zhao, Y.; Feng, X.; Li, Y.; Zhang, W.; Yamamoto, Y.; Bao, M., *ChemistrySelect* **2020**, *5*, 7086–7092.
9. Voth, S.; Hollett, J. W.; McCubbin, J. A., *J. Org. Chem.* **2015**, *80*, 2545–2553.
10. Chen, X.; Zhou, X.-Y.; Wu, H.; Lei, Y.-Z.; Li, J.-H., *Synth. Commun.* **2018**, *48*, 2475–2484.
11. Panja, S.; Kundu, D.; Ahammed, S.; Ranu, B. C., *Tetrahedron Lett.* **2017**, *58*, 3457–3460.
12. Sorribes, I.; Liu, L.; Corma, A., *ACS Catal.* **2017**, *7*, 2698–2708.
13. Hyodo, K.; Hasegawa, G.; Maki, H.; Uchida, K., *Org. Lett.* **2019**, *21*, 8, 2818–2822.
14. Wang, M.; Gao, M.; Zheng, Q.-H., *Bioorg. Med. Chem. Lett.* **2014**, *24*, 4455–4459.
15. Guo, H.; Zheng, R.; Jiang, H.; Xu, Z.; Xia, A., *BMC Chemistry* **2019**, *13*, 130.
16. Chandna, N.; Kaur, F.; Kumar, S.; Jain, N., *Green Chem.* **2017**, *19*, 4268–4271.
17. Cheung, C. W.; Surry, D. S.; Buchwald, S. L., *Org. Lett.* **2013**, *15*, 3734–3737.
18. Nourry, A.; Zambon, A.; Davies, L.; Niculescu-Duvaz, I.; Dijkstra, H. P.; Ménard, D.; Gaulon, C.; Niculescu-Duvaz, D.; Suijkerbuijk, B. M. J. M.; Friedlos, F.; Manne, H. A.; Kirk, R.; Whittaker, S.; Marais, R.; Springer, C. J., *J. Med. Chem.* **2010**, *53*, 1964–1978.
19. Ribeiro, C. J. A.; Espadinha, M.; Machado, M.; Gut, J.; Gonçalves, L. M.; Rosenthal, P. J.; Prudêncio, M.; Moreira, R.; Santos, M. M. M., *Bioorg. Med. Chem.* **2016**, *24*, 1786–1792.
20. Kattamuri, P. V.; Yin, J.; Siriwongsup, S.; Kwon, D.-H.; Ess, D. H.; Li, Q.; Li, G.; Yousufuddin, M.; Richardson, P. F.; Sutton, S. C.; Kürti, L., *J. Am. Chem. Soc.* **2017**, *139*, 11184−11196.

21. Cristau, H. –J.; Cellier, P. P.; Spindler J. –F.; Taillefer, M., *Chem. Eur. J.* **2004**, *10*, 5607–5622.
22. Lang, Q.; Gu, G.; Cheng,Y.; Yin, Q.; Zhang, X., *ACS Catal*. **2018**, *8*, 4824–4828.
23. Shen, X.; Li, Y.; Wen, Z.; Cao, S.; Hou, X.; Gong, L., *Chem. Sci*. **2018**, *9*, 4562–4568.
24. Miyake, Y.; Nakajima, K.; Nishibayashi, Y., *J. Am. Chem. Soc.* **2012**, *134*, 3338−3341.
25. Brown, D. S.; Elliott, M. C.; Moody, C. J.; Mowlem, T. J.; Marino, J. P. J.; Padwa, A., *J. Org. Chem.* **1994**, *59*, 2447–2455.
26. Xie, C.; Song, J.; Wu, H.; Hu, Y.; Liu, Hu.; Zhang, Z.; Zhang, P.; Chen, B.; Han, B., *J. Am. Chem. Soc*. **2019**, *141*, 4002–4009.
27. Lefebvre, Q.; Porta, R.; Millet, A.; Jia, J.; Rueping, M., *Chem. Eur. J.* **2020**, *26*, 1363–1367.
28. Freifeld, I.; Armbrust, H.; Langer, P., *Synthesis* **2006**, *11*, 1807–1810.
29. Millet, A.; Lefebvre, Q.; Rueping, M., *Chem. Eur. J.* **2016**, *22*, 13464–13468.
30. Cho, B. T.; Kang, S. K., *Synlett* **2004**, *9*, 1484–1488.
31. Poisson, T.; Gembus, V.; Oudeyer, S.; Marsais, F.; Levacher, V., *J. Org. Chem.* **2009**, *74*, 3516–3519.
32. Fava, E.; Millet, A.; Nakajima, M.; Loescher, S.; Rueping, M., *Angew. Chem. Int. Ed.* **2016**, *55*, 6776–6779.
33. Pavlishchuk, V. V.; Addison, A. W., *Inorganica Chimica Acta* **2000**, *298*, 97–102.
34. Roth, H. G.; Romero, N. A.; Nicewicz, D. A., *Synlett* **2016,** *27*, 714–723.
35. Prier, C. K.; Rankic, D. A.; MacMillan, D. W. C., *Chem. Rev.* **2013**, *113*, 5322-5363.
36. Cismenia, M. A.; Yoon, T. P., *Chem. Sci*. 2015, *6*, 5426–5434.
